# Large Language Models for Patient Education in Cardiovascular Imaging: Prospective Observational Comparative Study

**DOI:** 10.2196/95883

**Published:** 2026-08-26

**Authors:** Ahmed Marey, Basudha Pal, Ayşenur Buz Yaşar, Shree Rath, Giulia Francese, Hossam M Ghorab, Julia Niemierko, Muhammad Shah Wali Jamal, Muhammad Umair

**Affiliations:** 1 Shaikh Khalifa Medical City Abu Dhabi, Abu Dhabi United Arab Emirates; 2 Johns Hopkins University Baltimore, MD United States; 3 Bolu Abant İzzet Baysal University Bolu, Bolu Turkey; 4 All India Institute of Medical Sciences Bhubaneswar Bhubaneshwar, Odisha India; 5 Centre Hospitalier Universitaire de Rouen Rouen, Normandy France; 6 Alexandria University Alexandria, Alexandria Egypt; 7 Gdańsk Medical University Gdansk, Pomerania Poland; 8 King Edward Medical University Lahore, Punjab Pakistan; 9 Columbia University Irving Medical Center New York, NY United States; 10 Johns Hopkins Medicine Baltimore, MD United States

**Keywords:** large language models, LLMs, DeepSeek, GPT-4o, GPT-o1, cardiovascular imaging, patient education, artificial intelligence, AI

## Abstract

**Background:**

Large language models (LLMs) are increasingly used to support digital health communication, yet their reliability in patient-facing cardiovascular imaging education remains uncertain. Cardiovascular imaging involves complex terminology and procedural details that many patients struggle to understand, creating a need for accurate, clear, and reassuring explanations. While prior evaluations of conversational AI have focused primarily on diagnostic reasoning or clinician-oriented tasks, few studies have systematically compared contemporary LLMs in their ability to communicate effectively with patients.

**Objective:**

This study aimed to compare the accuracy, clarity, completeness, and patient-centered communication quality of responses generated by 3 state-of-the-art conversational agents (DeepSeek, GPT-o1, and GPT-4o) when addressing real-world patient questions about cardiovascular imaging.

**Methods:**

A prospective methodological evaluation was conducted using 84 unique patient-centered questions curated from authoritative cardiovascular information sources and online patient forums. Each question was independently submitted to DeepSeek, GPT-o1, and GPT-4o in isolated sessions to avoid contextual contamination. Two cardiovascular radiologists scored each response across 4 domains (accuracy, clarity and appropriateness, completeness, and user engagement and reassurance) using a standardized 3-point rubric (total score range 4-12). Discrepancies were resolved through predefined adjudication procedures. Because the scores were ordinal, median domain and composite scores with IQRs were summarized and compared across the 3 models using the Kruskal-Wallis test, with ε^2^ as an effect size. Statistical significance was defined as an α value of .05.

**Results:**

Across the 84 patient questions, all 3 models produced largely accurate, clear, and complete responses, with comparably high scores across the accuracy, clarity, and completeness domains (median 3 of 3, IQR 3-3 in each). The only meaningful difference appeared in user engagement and reassurance. A “good” engagement rating was assigned to 96.4% (81/84) of DeepSeek responses and 98.8% (83/84) of GPT-o1 responses but only 53.6% (45/84) of GPT-4o responses (Kruskal-Wallis *P*<.001). Composite scores were correspondingly lower for GPT-4o (median 11, IQR 10-12) than for DeepSeek and GPT-o1 (both median 12, IQR 11-12; *P*<.001). No significant differences were observed across models for accuracy (*P*=.91), clarity (*P*=.06), or completeness (*P*=.65), and no unsafe statements were identified in any model.

**Conclusions:**

DeepSeek and GPT-o1 consistently delivered accurate, clear, and patient-centered explanations of cardiovascular imaging questions, whereas GPT-4o, despite comparable technical accuracy, provided less engaging and reassuring communication. These findings suggest that affective qualities rather than factual correctness represent the main differentiator among current LLMs in patient education tasks. As conversational agents become integrated into cardiovascular imaging workflows, attention to communication tone, emotional support, and health literacy alignment will be essential to ensure safe and effective patient use.

## Introduction

AI-driven conversational systems have emerged as influential tools within digital health, where they are increasingly used to support patient education, supplement clinical communication, and provide accessible explanations of complex medical procedures. Large language models (LLMs) such as the generative pretrained transformer series are built on the transformer architecture introduced by Vaswani et al [1], which enables the modeling of long-range contextual relationships in text. Subsequent advances in generative pretraining and few-shot learning have further strengthened the capacity of these systems to produce coherent, contextually aligned, and clinically relevant responses [2,3].

In radiology, LLMs have been explored for tasks including automated report generation, radiologic decision support, examination preparation, and patient-facing explanations of imaging results [4-8]. Studies demonstrate that GPT-4 significantly improves upon GPT-3.5 in accuracy and reasoning when tested on radiology board–style assessments [9,10]. These advances have encouraged interest in using LLMs to facilitate patient education, particularly when time constraints and health communication burdens limit the depth of clinician-patient discussions. Patients frequently seek supplemental information online, and conversational agents are well positioned to address questions about imaging procedures that are otherwise difficult to understand.

DeepSeek [11] represents another class of conversational AI systems built using a mixture-of-experts architecture designed to improve computational efficiency and flexibility [12]. Early evaluations suggest potential clinical utility in imaging-related assistance, workflow support, patient guidance, and documentation [13,14]. As open-source models become more capable, they may broaden access to AI-assisted education in regions with limited digital infrastructure or fewer health care specialists.

Cardiovascular diseases remain the leading cause of morbidity and mortality worldwide, and cardiovascular imaging is integral to diagnosis, risk stratification, and therapeutic planning [15-17]. Modalities such as echocardiography, coronary computed tomography angiography, cardiac magnetic resonance imaging, and nuclear imaging provide essential clinical information but are often difficult for patients to interpret. Low health literacy, limited patient familiarity with imaging terminology, and variable clinician communication styles contribute to gaps in understanding that can hinder informed decision-making. Prior research in digital health highlights the importance of accessible patient education tools, particularly in specialized domains where information needs are high and health literacy challenges are common [18-22].

Although conversational agents show promise as supplemental educational tools, concerns remain regarding variability in accuracy, potential for hallucinations, inconsistent depth of explanation, and differences in empathetic communication [19,23,24]. These limitations are especially relevant in cardiovascular imaging, where misinterpretation of procedural details, risks, or preparation steps may lead to confusion or anxiety. Despite increasing public reliance on AI-generated medical information, few studies have systematically evaluated how contemporary LLMs respond to patient-centered questions specifically related to cardiovascular imaging. Prior evaluations of LLMs in radiology and patient education have consistently demonstrated strong performance in factual accuracy, clinical coherence, and alignment with established guidelines [25-27]. Contemporary systems such as DeepSeek, GPT-o1, and GPT-4o, therefore, reflect a level of technical maturity that supports their potential use as adjunct patient education tools. However, as accuracy and completeness have become increasingly comparable across state-of-the-art models, emerging differences are more likely to arise in affective and communicative dimensions rather than technical correctness alone. Empathy, reassurance, and tone are central to effective patient education, particularly in high-stakes settings such as cardiovascular imaging, where patient anxiety and uncertainty are common.

Building on our prior evaluation of earlier-generation language models, which emphasized overall response reliability [26], the present study examined a much larger and more diverse set of real-world patient questions and focused on state-of-the-art architectures (GPT-o1 [OpenAI] [27], GPT-4o [Open AI] [28], and DeepSeek [11]). This study also incorporated a refined evaluation rubric with explicit emphasis on user engagement and reassurance and quantitatively assessed affective communication differences using categorical and composite analyses. By centering the evaluation on real patient questions and examining communication quality rather than exam-style knowledge, this study addressed an underexplored aspect of how LLMs function in everyday clinical communication.

Accordingly, this study aimed to (1) assess the accuracy, clarity, and completeness of responses generated by GPT-o1, GPT-4o, and DeepSeek to patient-oriented cardiovascular imaging questions; (2) evaluate the user engagement and reassurance conveyed in these responses, including their capacity to support understanding and reduce procedure-related anxiety; (3) compare performance across the 3 models using a standardized evaluation framework; and (4) consider their feasibility as adjunct patient education tools, particularly in settings with limited specialist availability or health literacy challenges.

## Methods

### Study Design

This prospective methodological study evaluated the performance of 3 LLMs in generating patient-oriented information about cardiovascular imaging. We followed the STROBE (Strengthening the Reporting of Observational Studies in Epidemiology) checklist for observational studies from the EQUATOR (Enhancing the Quality and Transparency of Health Research) network [29] and have provided it in Multimedia Appendix 1.

### Ethical Considerations

This study did not involve human participants, the collection or analysis of identifiable private patient information, or any clinical intervention. All inputs were publicly available patient education materials, and all outputs were AI-generated text. The study, therefore, did not meet the regulatory definition of human subject research, and prospective institutional review board or research ethics board approval was not sought. This determination is consistent with the US Department of Health and Human Services Common Rule (Title 45 of the Code of Federal Regulations §46.102(e) [30], under which an activity constitutes human subject research only when an investigator obtains information or biospecimens through interaction with living individuals or obtains identifiable private information, and with the approach taken in prior evaluations of conversational agents in health care communication [25,26]. Because no human participants were enrolled and no identifiable data were used, informed consent was not applicable.

### Question Selection and Data Acquisition

Patient-centered questions were compiled from established cardiovascular information resources, including the American College of Cardiology, the American Heart Association, and MedlinePlus. These sources provide standardized, guideline-aligned educational material for patients with cardiovascular diseases [15-17]. To ensure broad representation of real-world patient concerns, additional questions were gathered from publicly accessible cardiovascular support forums and online patient communities, reflecting common uncertainties expressed in digital health environments.

All collected questions underwent independent review by 3 authors with experience in cardiovascular imaging and patient communication. During this review, questions that were repetitive, ambiguous, subjective, or not directly related to cardiovascular imaging were excluded. The final dataset consisted of 84 unique patient-oriented questions. The use of structured, curated question sets follows established approaches in prior LLM evaluation studies examining accuracy and patient communication quality [18,20,25,31]. Minor edits were applied to improve grammar and readability while preserving original meaning. The included questions encompassed procedural preparation, imaging risks, diagnostic purpose, radiation exposure, and lifestyle considerations associated with cardiovascular imaging.

### Response Generation Procedure

**Figure 1:**
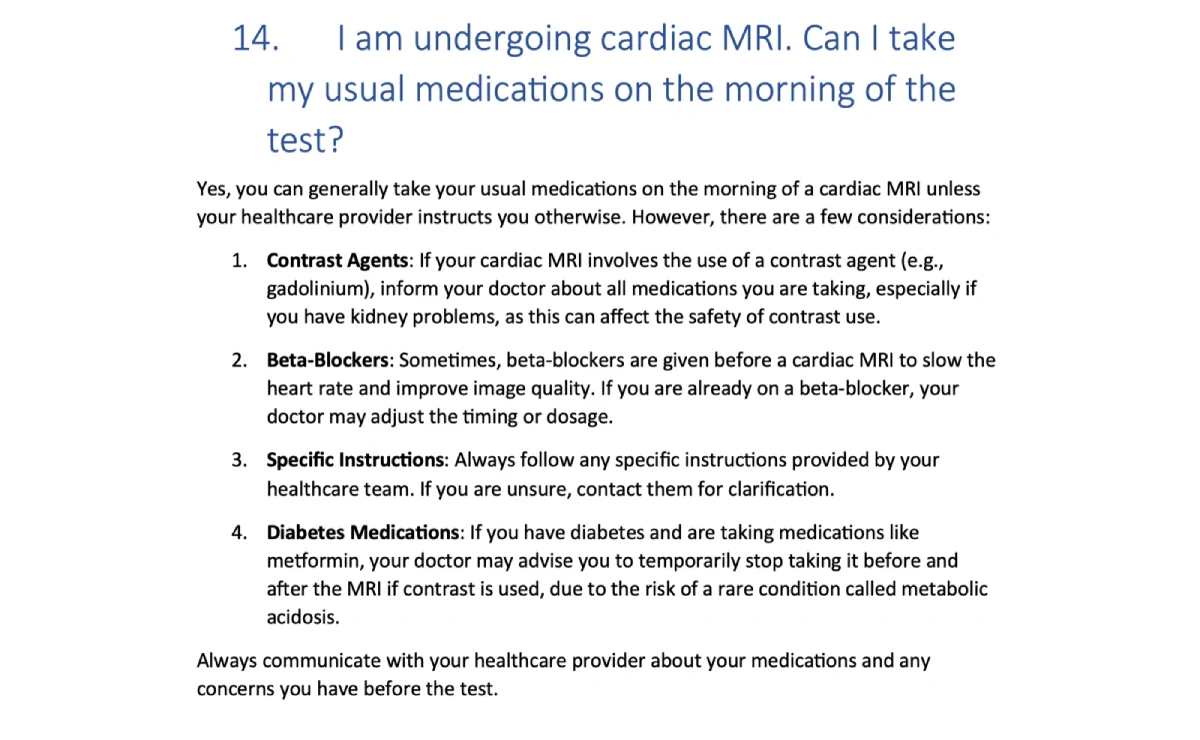
DeepSeek's response to question 14.

**Figure 2:**
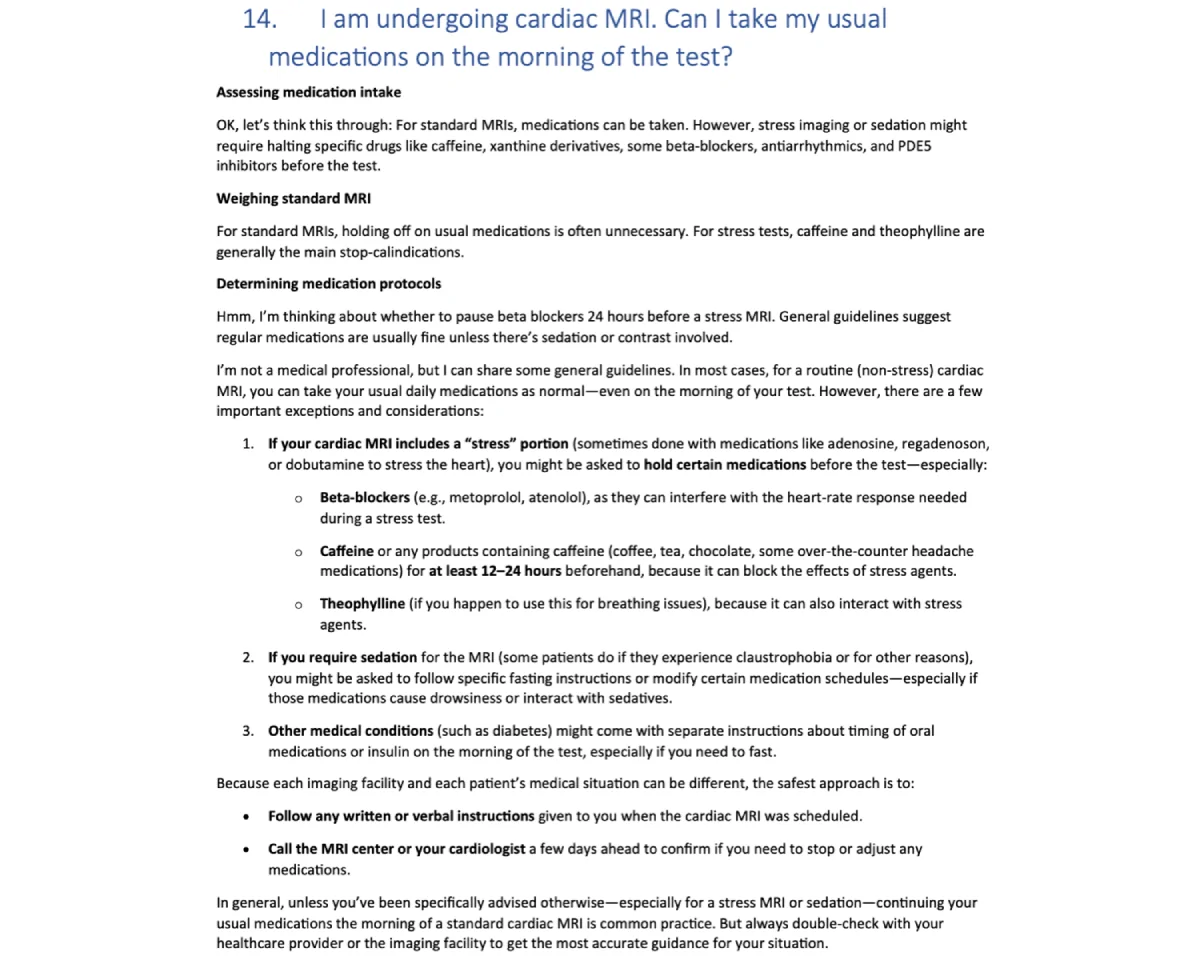
GPT-o1's response to question 14.

**Figure 3:**
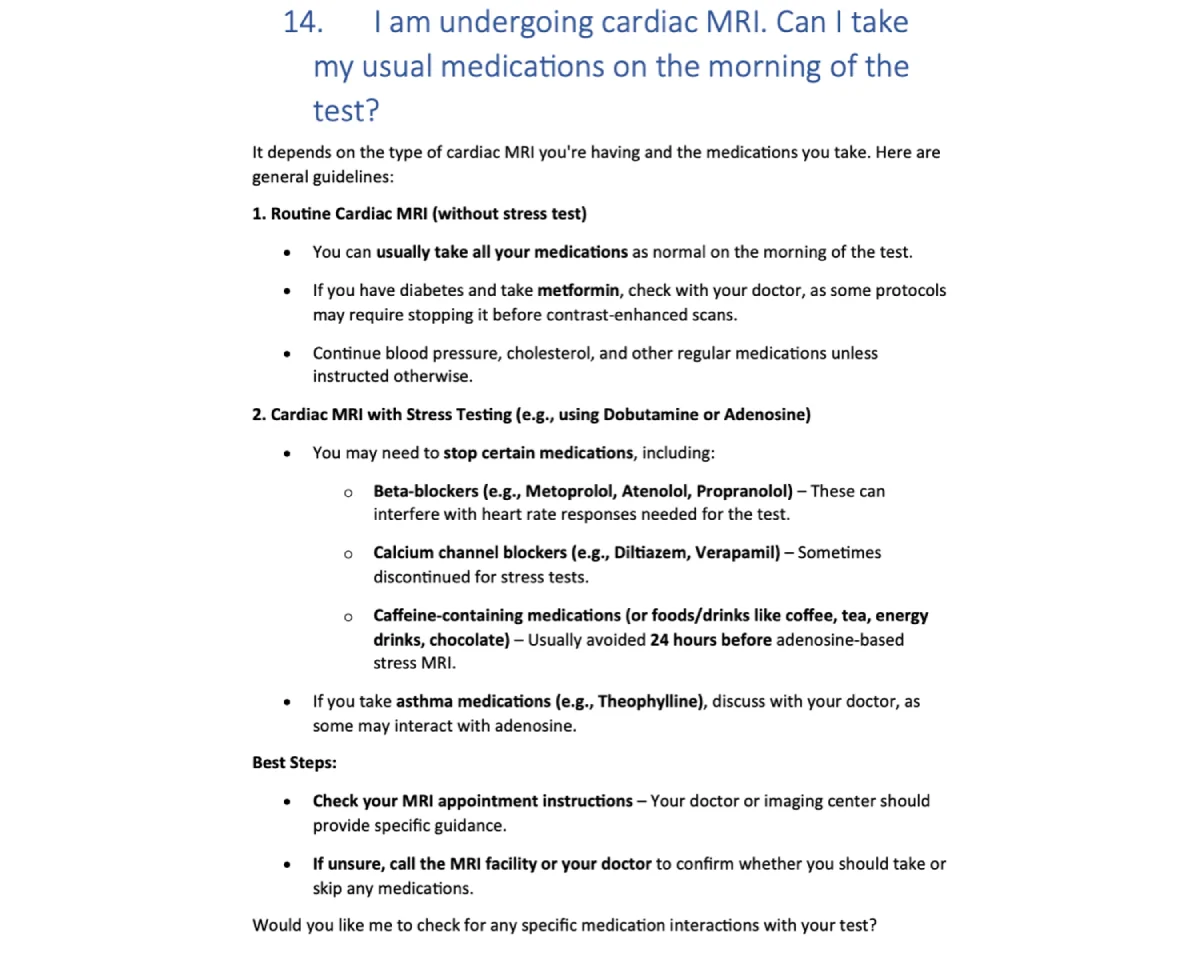
GPT-4o's response to question 14.

Each of the 84 questions was submitted to 3 LLMs: GPT-4o, GPT-o1, and DeepSeek. All responses were generated on May 9, 2025. To prevent contextual contamination across queries, each question was entered into a separate, newly initiated chat session, an approach consistent with single-turn evaluation methodologies used in previous radiology-focused LLM studies [8,20,32]. No follow-up prompts, clarifications, or feedback were provided. All questions were submitted in English. Each response was generated once and was not regenerated. Default platform settings were used for all models, and no generation parameters (eg, temperature or sampling settings) were manually modified by the investigators. Figures 1-3 show the different responses to one example question from the 3 models. The raw responses to all questions from each model can be found in Multimedia Appendices 2-4.

### Evaluation Framework

To evaluate the quality of AI-generated responses to patient inquiries regarding cardiovascular imaging, a structured assessment framework was developed based on established approaches used in prior analyses of conversational agents, medical question–answering systems, and radiology-focused LLM evaluations [25,26,31,32]. The framework consisted of 4 primary evaluation criteria: accuracy, clarity and appropriateness, completeness, and user engagement and reassurance.

**Figure 4:**
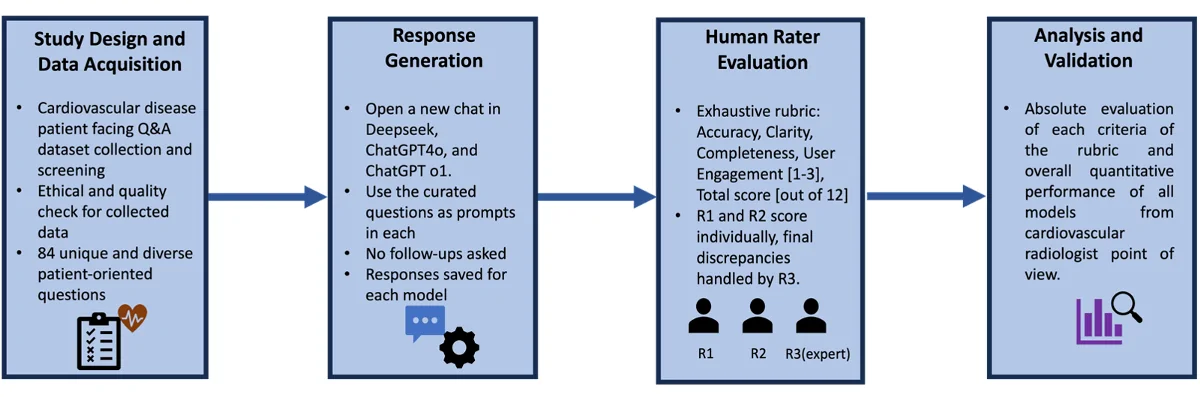
Methodological workflow for the comparative evaluation of AI-generated cardiovascular imaging information. This diagram illustrates the 4-stage pipeline designed to assess the clinical utility of large language models in patient education. Q&A: question and answer.

Two independent cardiovascular radiologists conducted the evaluations using this standardized scoring rubric. Using multiple expert reviewers and predefined scoring criteria aligns with recommended methodological practices for assessing communication quality and ensuring reproducibility in AI-mediated health information research [24,33]. Figure 4 illustrates the overall methodological workflow of the study, including question selection, response generation by the 3 LLMs, expert evaluation, and statistical analysis.

### Evaluation Criteria and Scoring System

#### Overview

Each AI-generated response was evaluated across the 4 domains (accuracy, clarity and appropriateness, completeness, and user engagement and reassurance) on a scale from 1 to 3, as described in Table 1. This multidimensional evaluation approach reflects prior frameworks used to assess patient communication, clinical accuracy, and usability in LLM outputs [25,26,31].

**Table 1:** Scoring rubric for the 4 evaluation domains. Each AI-generated response was rated on a scale from 1 to 3 on every domain.

Domain and score		Description
**Accuracy**		
	3 (excellent)	Fully accurate, up-to-date, aligned with established guidelines, and free from medical errors or misleading statements
	2 (moderate)	Mostly accurate, with minor inaccuracies or slightly outdated phrasing that do not substantially affect correctness
	1 (poor)	Contains significant inaccuracies, incorrect medical concepts, or misleading statements that could misinform patients
**Clarity and appropriateness**		
	3 (excellent)	Clear and well structured and uses simple language without sacrificing accuracy; free from excessive jargon or overly complex phrasing
	2 (moderate)	Mostly clear but contains some jargon, complex phrasing, or minor ambiguities
	1 (poor)	Poorly structured, contains excessive medical jargon, or an overly simplistic response that lacks necessary details
**Completeness**		
	3 (excellent)	Completely answers the question, addressing all relevant aspects comprehensively
	2 (moderate)	Partially answers the question, omitting minor details but still providing useful information
	1 (poor)	Incomplete response; omits key details necessary for understanding
**User engagement and reassurance**		
	3 (excellent)	Supportive, reassuring, and encourages informed patient engagement with clinicians
	2 (moderate)	Moderately engaging but lacks strong reassurance or appropriate motivational phrasing
	1 (poor)	Neutral, overly robotic, or anxiety-provoking language that may negatively affect patient confidence

#### Accuracy

Accuracy assesses whether the response is factually correct, consistent with contemporary cardiovascular imaging guidelines (such as those from the American Heart Association and American College of Cardiology), and free from medical errors or misleading statements. Accuracy has been a central outcome in prior LLM performance studies in radiology and clinical education [25,31,34]. Assessment of accuracy considered alignment with evidence-based medical practices, the presence of factual inaccuracies or outdated concepts, the correct use of medical terminology, and the avoidance of speculation or unverified claims.

#### Clarity and Appropriateness

This domain evaluates whether the response is logically structured and clearly written, uses accessible language, and is appropriate for a general patient audience. Clarity and readability are consistent with digital health literacy and patient education evaluation frameworks [18,21]. Assessment of clarity and appropriateness considered the use of patient-friendly language while maintaining medical accuracy, the logical structure and clarity of the explanation, and an appropriate level of detail without excessive complexity.

#### Completeness

Completeness assesses whether the response fully addresses the patient’s inquiry without omitting essential clinical or procedural information. Completeness has been a key quality indicator in prior evaluations of medical LLM outputs [25,31]. Assessment of completeness considered the inclusion of all essential aspects of the question and the avoidance of partial explanations that may leave patients uncertain.

#### User Engagement and Reassurance

This domain evaluates whether the response provides emotional support, fosters patient confidence, and encourages appropriate follow-up with health care professionals. This dimension is aligned with patient-centered communication evaluations in conversational AI research [19,22,25]. Assessment of user engagement and reassurance considered the encouragement of patient empowerment and of further discussion with a health care provider and the avoidance of unnecessary alarmism or overly cautious phrasing.

### Discrepancy Resolution Mechanism

To minimize evaluator subjectivity, a predefined discrepancy resolution procedure was implemented. This approach mirrors adjudication methodologies used in diagnostic performance research and multi-rater AI evaluation studies [24,35]. Disagreements were resolved through a structured process. The 2 cardiovascular radiologists first scored each response independently. Where scores differed by more than 2 points on any category, the evaluators discussed their rationale to reach consensus. For all other disagreements, an independent cardiovascular imaging expert served as the final arbitrator.

### Final Score Calculation

The composite score was calculated by summing the scores across the 4 evaluation domains (accuracy, clarity and appropriateness, completeness, and user engagement and reassurance), resulting in a total score ranging from 4 to 12. Composite scores were interpreted as follows: a score of 12 indicated excellent performance, reflecting responses that were fully accurate, clear, comprehensive, and patient centered; scores of 10 to 11 indicated good performance with only minor limitations; scores of 7 to 9 indicated adequate performance requiring some improvement; and scores of 4 to 6 indicated poor performance with substantial deficiencies across one or more evaluation domains.

### Statistical Analysis

For each response and domain, the value entered into the analysis was the single adjudicated score: in cases in which the 2 primary reviewers agreed, that score was used directly, and in cases in which they disagreed, the score assigned by the senior reviewer (R3) was used. The composite total score for each response was then obtained by summing the 4 adjudicated domain scores (each scored from 1-3), yielding a possible range of 4 to 12. Because the domains were measured on a 3-point ordinal scale, scores for each domain and for the composite total were summarized as medians with IQRs rather than as means with SDs. Differences in performance across the 3 LLMs were assessed using the Kruskal-Wallis test, a nonparametric method appropriate for comparing ordinal scores across 3 independent groups. Interrater reliability was assessed using the Cohen κ with quadratic weighting, which accounts for the ordinal structure of the 3-point scoring rubric and penalizes larger discrepancies more heavily than minor disagreements. Agreement was quantified between each primary reviewer (R1 and R2) and the adjudicating senior cardiovascular radiologist (R3). Agreement between R1 and R2 was not evaluated as the primary objective of the reliability analysis was to assess consistency relative to the final adjudicated reference standard rather than raw concordance between initial reviewers. The adjudicating radiologist (R3), as the most experienced reviewer, resolved all discrepancies and generated the final scores used for downstream analysis; therefore, agreement with R3 was considered the most clinically and methodologically relevant measure of scoring reliability. For each domain, and for the composite score, the Kruskal-Wallis *H* statistic, its df, the associated *P* value, and ε^2^ as an effect size estimate are reported. The distribution of ordinal ratings across score categories is also presented as counts and percentages for descriptive purposes only to show how responses were spread across the rating categories within each domain; these percentages were not subjected to separate inferential testing. All statistical analyses were performed using R (version 4.5.2; R Foundation for Statistical Computing), with statistical significance defined as an α value of .05.

## Results

### Overview

The evaluation sheets completed independently by the 2 primary cardiovascular radiologists, with adjudication by a third radiologist, can be found in Multimedia Appendices 5-10. Evaluations for all 3 models by the remaining reviewer are provided as separate worksheets in Multimedia Appendix 11.

Interrater reliability analysis (Table 2) demonstrated moderate agreement across all models based on commonly used interpretive benchmarks for the Cohen κ (eg, values of 0.41-0.60 indicating moderate agreement and 0.61-0.80 indicating substantial agreement). Notably, GPT-4o exhibited slightly higher quadratic weighted Cohen κ values than DeepSeek and GPT-o1. This finding likely reflects the relative uniformity and neutrality of GPT-4o’s responses, which may be easier to score consistently, rather than superior communication quality. In contrast, the more expressive and patient-centered responses generated by DeepSeek and GPT-o1 may introduce greater subjective variability between raters despite higher overall performance. Table 3 presents the descriptive statistics for all 4 scoring domains and the total composite score for each model. These differences were modest and should be interpreted cautiously as interrater reliability reflects consistency of scoring rather than intrinsic quality, clinical accuracy, or educational effectiveness of the model responses. Importantly, the observed agreement levels across all models indicate that the scoring rubric could be applied reproducibly by independent reviewers, supporting the robustness of the evaluation framework rather than implying clinically meaningful distinctions between models.

GPT-o1 demonstrated a consistently strong performance across all domains. Its responses were frequently rated as fully accurate, comprehensive, and clearly structured. Notably, it achieved the highest level of user engagement and reassurance among the 3 models, indicating a communication style that was patient-friendly and supportive. Overall, GPT-o1 produced balanced, high-quality outputs with strengths in clarity and patient-centered phrasing. DeepSeek showed a similarly favorable performance profile, with particularly strong clarity and readability. The model produced well-organized explanations that were easy for lay users to understand. Accuracy and completeness were comparable to those of GPT-o1, and its user engagement scores were also high, although marginally lower than those of GPT-o1. DeepSeek’s overall performance indicates a reliable ability to deliver clear and reassuring patient education content. GPT-4o demonstrated accuracy and completeness comparable to those of the other 2 models but differed meaningfully in its communication tone. While technically sound, its responses were more neutral and less patient oriented, resulting in lower scores in the user engagement and reassurance domain. This reduced patient-centeredness contributed to a lower total score relative to GPT-o1 and DeepSeek despite similar technical quality.

Overall, the descriptive statistics in Table 3 show that all 3 models are capable of producing accurate and complete responses. However, GPT-o1 and DeepSeek received higher ratings than GPT-4o in affective and engagement-related qualities, which may be particularly important in patient-facing educational contexts.

**Table 2:** Interrater agreement on the evaluations of the different models.

AI model	Cohen κ (R3 vs R1)^a^	Cohen κ (R3 vs R2)^a^	*P* value
DeepSeek	0.564	0.413	*<.001* ^b^
GPT-o1	0.519	0.582	*<.001* ^b^
GPT-4o	0.655	0.638	*<.001* ^b^

^a^ R1, R2, and R3 means raters 1, 2, and 3, respectively.

^b^Statistically significant at α=.05.

**Table 3:** Descriptive scores and Kruskal-Wallis comparisons of the 3 large language models across the 4 evaluation domains and the composite score^a^.

Domain (score range)	DeepSeek, median (IQR; range)	GPT-o1, median (IQR; range)	GPT-4o, median (IQR; range)	*H* statistic (*df*)	*P* value	ε^2^
Accuracy (1-3)	3 (3-3; 2-3)	3 (3-3; 2-3)	3 (3-3; 1-3)	0.20 (2)	.91^b^	0.001
Clarity and appropriateness (1-3)	3 (3-3; 1-3)	3 (3-3; 2-3)	3 (3-3; 1-3)	5.60 (2)	.06^b^	0.022
Completeness (1-3)	3 (3-3; 2-3)	3 (3-3; 2-3)	3 (3-3; 2-3)	0.85 (2)	.65^b^	0.003
User engagement and reassurance (1-3)	3 (3-3; 2-3)	3 (3-3; 2-3)	3 (2-3; 2-3)	76.64 (2)	*<.001* ^*c*^	0.305
Total score (4-12)	12 (11-12; 10-12)	12 (11-12; 10-12)	11 (10-12; 8-12)	17.37 (2)	*<.001* ^*c*^	0.069

^a^Between-model differences were assessed using the Kruskal-Wallis test; ε^2^ denotes the effect size. Statistical significance was defined as α=.05.

^b^Not statistically significant at α=.05.

^c^Statistically significant at α=.05.

### Comparative Classification Results

Table 4 presents the categorical distribution of scores across the 3 LLMs for descriptive purposes. For the accuracy domain, inaccurate responses were rare and limited to GPT-4o, whereas the proportion of fully accurate responses was similarly high across all 3 models.

For clarity and appropriateness, most responses from all models were rated as clear and concise, and unclear or confusing responses were rare across all 3 models. For completeness, most responses from every model were rated as comprehensive, with the remainder rated as partially comprehensive. The only domain with a meaningful difference was user engagement and reassurance. DeepSeek and GPT-o1 generated predominantly high-engagement responses, whereas GPT-4o produced a substantially lower proportion of high-engagement responses and a correspondingly higher proportion of moderate ones. This communication-related deficit contributed directly to the lower overall ratings of GPT-4o. Differences were also reflected in total score distributions. DeepSeek and GPT-o1 exhibited the highest proportion of perfect scores, whereas GPT-4o achieved a perfect score less often and showed a higher frequency of midrange scores, indicating that its responses, while technically sound, were less consistently reassuring and supportive for patients.

**Figure 5:**
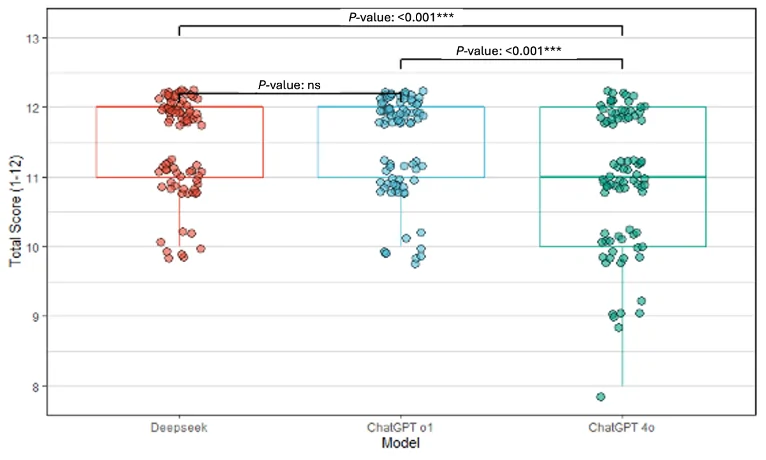
Box plots and raw distributions of total composite scores (4-12) across models. DeepSeek and GPT-o1 showed closely overlapping composite score distributions, whereas GPT-4o showed a lower and more variable distribution. The overall difference across the 3 models was significant on the Kruskal-Wallis test (*P*<.001). NS: not significant.

Figure 5 illustrates the distribution of total composite scores across models. While DeepSeek and GPT-o1 showed highly overlapping score distributions and did not differ significantly from one another, both models achieved higher total scores than GPT-4o. The broader spread and downward shift observed for GPT-4o align with its lower engagement and reassurance ratings, indicating greater variability and less consistently patient-centered communication.

**Table 4:** Evaluation of the responses generated by each large language model across 4 domains (accuracy, clarity and appropriateness, completeness, and user engagement and reassurance) using 3-point ordinal scales.

Term			Model, n (%)				
			DeepSeek (n=84)		GPT-o1 (n=84)		GPT-4o (n=84)
**Accuracy (1-3)**							
	Inaccurate	0 (0)		0 (0)		2 (2.4)	
	Partially accurate	15 (17.9)		13 (15.5)		11 (13.1)	
	Accurate	69 (82.1)		71 (84.5)		71 (84.5)	
**Clarity and appropriateness (1-3)**							
	Unclear or confusing	1 (1.2)		0 (0)		1 (1.2)	
	Somewhat clear	6 (7.1)		15 (17.9)		17 (20.2)	
	Clear and concise	77 (91.7)		69 (82.1)		66 (78.6)	
**Completeness (1-3)**							
	Incomprehensive	0 (0)		0 (0)		0 (0)	
	Partially comprehensive	18 (21.4)		14 (16.7)		14 (16.7)	
	Comprehensive	66 (78.6)		70 (83.3)		70 (83.3)	
**User engagement and reassurance (1-3)**							
	Poor	0 (0)		0 (0)		0 (0)	
	Moderate	3 (3.6)		1 (1.2)		39 (46.4)	
	Good	81 (96.4)		83 (98.8)		45 (53.6)	
**Total score (4-12; no model had a total score<8)**							
	8	0 (0)		0 (0)		1 (1.2)	
	9	0 (0)		0 (0)		6 (7.1)	
	10	8 (9.5)		9 (10.7)		18 (21.4)	
	11	28 (33.3)		25 (29.8)		29 (34.5)	
	12	48 (57.1)		50 (59.5)		30 (35.7)	

## Discussion

### Principal Findings

This study evaluated the quality of patient-facing cardiovascular imaging information generated by GPT-o1, GPT-4o, and DeepSeek across the domains of accuracy, clarity, completeness, and user engagement. Overall, all 3 models demonstrated similarly strong technical performance, with no statistically significant differences in accuracy, clarity, or completeness, indicating that contemporary LLMs are consistently capable of providing factually correct, clear, and comprehensive explanations of cardiovascular imaging for patients. The primary distinction between the models emerged in the user engagement and reassurance domain, where DeepSeek and GPT-o1 consistently generated more supportive, empathetic, and patient-centered responses than GPT-4o. Although the absolute differences in composite scores were modest, they were driven largely by these communication-related characteristics rather than by differences in technical correctness. These findings suggest that state-of-the-art LLMs have largely converged in their ability to deliver accurate educational content and that communication style, reassurance, and patient-centered language may now represent the principal factors differentiating their suitability for patient education. This distinction is particularly relevant in cardiovascular imaging, where patients often seek explanations of unfamiliar procedures while experiencing uncertainty or anxiety, making both the quality of information and the manner in which it is communicated important components of effective patient education.

### Accuracy, Clarity, and Completeness

The consistently strong performance observed across the accuracy, clarity, and completeness domains aligns with a growing body of literature demonstrating that modern LLMs can effectively generate medically appropriate educational content for patients. Prior evaluations across multiple medical specialties have reported that leading LLMs frequently provide responses that are accurate, comprehensive, and accessible to nonexpert audiences, supporting their potential utility as patient education resources [24,33]. Similar observations have been reported in studies evaluating patient-facing questions in radiology, oncology, cardiology, and surgical care, where LLM-generated responses often achieved high ratings for factual correctness and readability while maintaining language that could be understood by lay users [24,33].

The absence of meaningful differences between models in these technical domains suggests that all 3 systems were capable of conveying essential information regarding cardiovascular imaging procedures. This finding is encouraging because patient education represents one of the most promising and comparatively lower-risk applications of LLM technology within health care. Unlike diagnostic decision-making, patient education primarily involves translating established clinical knowledge into understandable explanations. The ability of multiple independent models to perform similarly in this setting may therefore reflect increasing maturity of LLMs for educational applications.

### User Engagement and Reassurance

Although technical performance was largely comparable across models, differences emerged in user engagement and reassurance. Responses perceived as more supportive, empathetic, and patient centered generally received higher overall evaluations despite containing information that was often similar in factual content. This observation is consistent with previous research indicating that patients value communication qualities such as empathy, emotional support, reassurance, and conversational tone in addition to factual accuracy [24,33].

Importantly, effective patient education extends beyond the transmission of correct information. Patients undergoing cardiovascular imaging procedures may experience anxiety related to diagnostic uncertainty, radiation exposure, contrast administration, procedural discomfort, or potential findings. In these contexts, responses that acknowledge concerns and provide reassurance may contribute meaningfully to patient understanding and satisfaction. Prior studies comparing LLM-generated and clinician-generated responses have similarly reported that communication style and perceived empathy often influence overall evaluations of response quality even when factual content is comparable [24,33].

These findings suggest that evaluation frameworks for patient-facing AI systems should not focus exclusively on factual correctness. Measures of engagement, reassurance, readability, and patient-centered communication may be equally important when assessing the real-world utility of LLM-generated educational content. Future model development efforts may therefore benefit from explicitly optimizing both informational quality and supportive communication.

### Implications for Patient Education

Taken together, the findings support the potential role of LLMs as adjunct tools for cardiovascular imaging education. Such systems may help patients obtain preliminary information, reinforce explanations provided by health care professionals, and improve access to educational resources outside clinical encounters. However, their greatest value is likely to arise when they complement rather than replace clinician-patient communication.

The results further suggest that future evaluations of patient-facing LLMs should incorporate broader measures of communication effectiveness, including patient comprehension, trust, anxiety reduction, and perceived usefulness. Although technical accuracy remains essential, educational success ultimately depends on whether information is communicated in a manner that patients find understandable, reassuring, and actionable.

### Hallucinations and Safety Considerations

Although no hallucinations or clinically concerning inaccuracies were identified in the responses evaluated in this study, hallucinations remain a recognized limitation of LLMs in health care and have been documented even in high-performing systems evaluated across clinical and biomedical domains [23,34,35]. Consequently, patient-facing deployment should incorporate appropriate safeguards to minimize the risk of incorrect or unsupported information. Proposed mitigation strategies include retrieval-augmented generation approaches that ground responses in curated evidence sources, structured prompting techniques, citation-aware generation, and ongoing human oversight [36-38]. Continued monitoring, domain-specific refinement, and transparent reporting of safety limitations will remain important as LLMs become increasingly integrated into health care communication workflows [39,40].

### Implications for Low- and Middle-Income Countries

The ability of LLMs to provide accessible educational information may have particular relevance in settings where access to health care professionals and patient education resources is limited. Prior work has highlighted the potential value of AI-based tools in health care systems facing shortages of trained specialists and educational resources [41]. However, the present study did not directly evaluate model performance across different languages, cultures, health care systems, or resource-constrained environments. Differences in digital literacy, internet access, and linguistic availability may substantially influence real-world utility. Consequently, further work is needed before conclusions regarding the applicability of these findings to low- and middle-income countries can be drawn.

### Limitations

This study has several limitations. First, LLM behavior evolves rapidly as models undergo continuous updates and retraining. These findings represent a snapshot of model behavior at a specific time point and should not be interpreted as immutable performance characteristics. Second, the analysis was limited to English-language questions, which may not generalize to multilingual or culturally adapted settings. Third, while this study evaluated patient-oriented communication, it did not assess clinical reasoning depth or medical decision-making accuracy, which are distinct dimensions of LLM performance. Fourth, even with a structured rubric, the scoring depended on expert judgment, which introduces subjectivity and may not capture all subtleties of communication quality. In particular, domains such as user engagement and reassurance represent inherently subjective constructs, and their evaluation reflects a clinical communication perspective rather than direct end user perception. Patient-based scoring or feedback was not incorporated in the present study, and future work should examine how patients themselves perceive engagement, reassurance, and the overall usefulness of LLM-generated explanations. Finally, despite discussing the potential relevance for low- and middle-income country settings, the present study did not directly evaluate model utility, accessibility, or safety in these environments, and thus, conclusions about their broader global applicability should be interpreted with caution.

### Conclusions

These findings carry several broader implications for the use of LLMs in patient education. As the models differed primarily in the affective and patient-centered quality of their communication rather than in clinical correctness, communication tone, empathy, and reassurance should be considered explicit design and evaluation targets for patient-facing AI systems. Realizing the potential of these tools at scale, particularly in settings where access to specialist education is limited, will require safeguards against hallucinations, strategies that promote supportive and comprehensible responses, and adaptation to diverse health literacy levels and languages. Prospective studies involving patients and evaluating real-world outcomes such as comprehension, anxiety reduction, and decision-making will be essential before these technologies can be safely integrated into routine cardiovascular imaging education.

## Appendix

Multimedia Appendix 1 STROBE checklist.

Multimedia Appendix 2 Raw responses generated by model 1 to all 84 patient-oriented questions.

Multimedia Appendix 3 Raw responses generated by model 2 to all 84 patient-oriented questions.

Multimedia Appendix 4 Raw responses generated by model 3 to all 84 patient-oriented questions.

Multimedia Appendix 5 Evaluation sheet for the DeepSeek responses completed by the first primary reviewer (R1).

Multimedia Appendix 6 Evaluation sheet for the GPT-4o responses completed by the first primary reviewer (R1).

Multimedia Appendix 7 Evaluation sheet for the GPT-o1 responses completed by the first primary reviewer (R1).

Multimedia Appendix 8 Evaluation sheet for the DeepSeek responses completed by the adjudicating reviewer (R3).

Multimedia Appendix 9 Evaluation sheet for the GPT-4o responses completed by the adjudicating reviewer (R3).

Multimedia Appendix 10 Evaluation sheet for the GPT-o1 responses completed by the adjudicating reviewer (R3).

Multimedia Appendix 11 Evaluation sheets for all 3 models completed by the second primary reviewer (R2), provided as separate worksheets.

## Abbreviations

EQUATOR: Enhancing the Quality and Transparency of Health Research
LLM: large language model
STROBE: Strengthening the Reporting of Observational Studies in Epidemiology

## Data Availability

All the detailed responses from the models and the ratings by reviewers are available as supplementary information.
