# Supplementary material for: Large Language Models for Patient Education in Cardiovascular Imaging: Prospective Observational Comparative Study
Source: JMIR Form Res. 2026 Aug 26;10:e95883. doi: 10.2196/95883 (PMC13559148; doi:10.2196/95883)
Supplement: Supplementary file 2 [file formative_v10i1e95883_app2.docx]

# 1. I am undergoing cardiac CT. What is a cardiac CT scan, and Why might I need one?

A cardiac CT (computed tomography) scan is a non-invasive imaging test that uses X-rays to create detailed pictures of your heart and its blood vessels. It provides a 3D view of the heart, allowing doctors to assess its structure, function, and blood flow. Here's a breakdown of what it involves and why you might need one:

**What is a Cardiac CT Scan?**

- **How it works**: A cardiac CT scan uses a rotating X-ray machine to take multiple images of your heart from different angles. These images are then combined by a computer to create a detailed 3D model.
- **Types of cardiac CT scans**:
  - **Calcium score scan**: Measures calcium deposits in the coronary arteries to assess the risk of coronary artery disease (CAD).
  - **CT coronary angiogram**: Uses a contrast dye to visualize blood flow through the coronary arteries and detect blockages or narrowing.
- **Duration**: The scan typically takes 10–15 minutes, though preparation (e.g., administering contrast dye) may take longer.

**Why Might You Need a Cardiac CT Scan?**

Your doctor may recommend a cardiac CT scan to:

1. **Evaluate chest pain**: Determine if chest pain is caused by reduced blood flow to the heart (angina) due to narrowed or blocked arteries.
2. **Assess coronary artery disease (CAD)**: Detect plaque buildup or blockages in the coronary arteries.
3. **Check heart structure and function**: Evaluate the size, shape, and function of the heart, including the chambers, valves, and major vessels.
4. **Plan for procedures**: Guide treatments like stent placement, bypass surgery, or valve repair.
5. **Monitor existing conditions**: Track the progression of heart disease or the effectiveness of treatments.
6. **Screen for heart disease**: Assess risk in individuals with risk factors like high cholesterol, diabetes, or a family history of heart disease.

**Benefits and Risks**

- **Benefits**: Non-invasive, quick, and provides highly detailed images.
- **Risks**: Minimal, but may include exposure to radiation (low dose) and potential allergic reactions to contrast dye.

If you have concerns or questions about the procedure, discuss them with your doctor to ensure it’s the right test for your situation.

# 2. I am undergoing cardiac MRI. What is a cardiac MRI, and why might I need one?

A cardiac MRI (Magnetic Resonance Imaging) is a non-invasive imaging test that uses powerful magnets and radio waves to create detailed pictures of your heart and surrounding structures. Unlike X-rays or CT scans, it does not use ionizing radiation. The images produced can show the heart's anatomy, function, blood flow, and tissue characteristics in great detail.

**Why Might You Need a Cardiac MRI?**

Your doctor may recommend a cardiac MRI to:

1. **Evaluate Heart Structure and Function**: Assess the size, shape, and function of the heart chambers, valves, and major blood vessels.
2. **Detect Heart Disease**: Identify conditions such as coronary artery disease, cardiomyopathies, or congenital heart defects.
3. **Assess Damage After a Heart Attack**: Determine the extent of damage to the heart muscle and scar tissue.
4. **Evaluate Tumors or Masses**: Detect and characterize tumors or abnormal growths in or around the heart.
5. **Monitor Heart Conditions**: Track the progression of diseases like heart failure or monitor the effects of treatments.
6. **Investigate Inflammation or Infection**: Diagnose myocarditis, pericarditis, or other inflammatory conditions.
7. **Plan for Surgery or Procedures**: Provide detailed information to guide treatment decisions, such as surgery or catheter-based interventions.

**What to Expect During the Test**

- You will lie on a table that slides into the MRI machine, which is a large, tunnel-like structure.
- The machine will create loud knocking sounds, so you may be given earplugs or headphones.
- You may receive a contrast dye (gadolinium) through an IV to enhance the images.
- The test typically takes 45–90 minutes, and you will need to remain still during the scan.

**Safety and Precautions**

- Cardiac MRI is generally safe, but inform your doctor if you have metal implants, pacemakers, or other devices, as these may not be compatible with the MRI machine.
- Let your doctor know if you are pregnant or have kidney problems, especially if contrast dye is used.

A cardiac MRI provides highly detailed information, helping your healthcare team make an accurate diagnosis and develop the best treatment plan for your condition. If you have concerns or questions, don’t hesitate to discuss them with your doctor.

# 3. I am undergoing heart CT. How would this test help diagnose my condition?

A heart CT (computed tomography) scan is a valuable diagnostic tool that provides detailed images of your heart and surrounding structures. It can help diagnose and evaluate various heart conditions in the following ways:

**1. Assessing Coronary Artery Disease (CAD)**

- A heart CT can detect **plaque buildup** in the coronary arteries, which supply blood to the heart muscle. This helps identify **narrowing or blockages** that could lead to chest pain (angina) or a heart attack.
- A **coronary calcium scan** (a type of heart CT) measures the amount of calcium in your coronary arteries, which is a marker of atherosclerosis (hardening of the arteries).

**2. Evaluating Heart Structure and Function**

- The scan provides detailed images of the heart's chambers, valves, and muscles. It can detect abnormalities such as:
  - **Enlarged heart** (cardiomegaly)
  - **Congenital heart defects** (structural problems present from birth)
  - **Heart valve issues** (e.g., stenosis or regurgitation)

**3. Detecting Blood Clots or Tumors**

- A heart CT can identify blood clots in the heart or nearby blood vessels, which could lead to a stroke or pulmonary embolism.
- It can also detect tumors or masses in or around the heart.

**4. Evaluating Pericardial Disease**

- The scan can reveal problems with the pericardium (the sac surrounding the heart), such as **pericarditis** (inflammation) or **pericardial effusion** (fluid buildup).

**5. Planning for Procedures**

- A heart CT can help doctors plan for surgeries or interventions, such as **coronary artery bypass grafting (CABG)** or **stent placement**, by providing a detailed map of the heart and blood vessels.

**6. Assessing Aortic Conditions**

- The scan can detect issues with the aorta, such as **aneurysms** (enlargement) or **dissections** (tears in the aortic wall), which are life-threatening if not treated promptly.

**7. Monitoring Known Conditions**

- If you have a known heart condition, a heart CT can help monitor its progression or assess the effectiveness of treatment.

**How the Test Works:**

- During the scan, you lie on a table that slides into a doughnut-shaped machine. A contrast dye may be injected into your bloodstream to highlight blood vessels and heart structures.
- The CT machine uses X-rays to create cross-sectional images of your heart, which are then reconstructed into 3D images for detailed analysis.

**What Your Doctor Can Learn:**

- The presence and severity of coronary artery disease.
- The condition of your heart valves and chambers.
- Blood flow through the heart and major vessels.
- Any structural abnormalities or signs of disease.

By providing this detailed information, a heart CT helps your doctor make an accurate diagnosis and develop an appropriate treatment plan tailored to your condition. If you have specific symptoms or concerns, discuss them with your doctor to better understand how this test applies to your situation.

# 4. I am undergoing heart MRI. How would this test help diagnose my condition?

A heart MRI, also known as a cardiac MRI, is a detailed imaging test that provides high-resolution pictures of your heart and surrounding structures. It can help diagnose and evaluate various heart conditions by offering detailed information about the heart's anatomy, function, and blood flow. Here’s how it can assist in diagnosing your condition:

**1. Assessing Heart Structure**

- It provides clear images of the heart's chambers, valves, and muscles, helping to identify abnormalities such as congenital heart defects, tumors, or scarring.

**2. Evaluating Heart Function**

- A cardiac MRI measures how well your heart is pumping (ejection fraction), detects issues with heart muscle movement, and identifies areas of weakened or damaged tissue.

**3. Detecting Heart Tissue Damage**

- It can identify scar tissue or inflammation in the heart muscle, which may indicate conditions like myocardial infarction (heart attack) or myocarditis (inflammation of the heart muscle).

**4. Evaluating Blood Flow**

- The test can assess blood flow through the heart and major vessels, helping to diagnose conditions like congenital heart disease, valve disorders, or blockages.

**5. Identifying Cardiomyopathy**

- It helps diagnose different types of cardiomyopathy (diseases of the heart muscle), such as hypertrophic or dilated cardiomyopathy, by showing changes in heart muscle thickness or size.

**6. Assessing Pericardial Disease**

- A cardiac MRI can detect issues with the pericardium (the sac around the heart), such as pericarditis or fluid buildup.

**7. Planning for Treatment**

- The detailed images can guide treatment decisions, such as the need for surgery, medications, or other interventions.

**8. Monitoring Progress**

- If you have a known heart condition, a cardiac MRI can monitor disease progression or the effectiveness of treatment over time.

**Conditions It Can Help Diagnose:**

- Coronary artery disease
- Heart failure
- Congenital heart defects
- Heart valve disease
- Cardiomyopathy
- Myocarditis or pericarditis
- Cardiac tumors
- Aortic disease

Your doctor will use the results of the MRI, along with your symptoms, medical history, and other tests, to make an accurate diagnosis and develop a treatment plan tailored to your condition. If you have specific concerns, discuss them with your healthcare provider for more personalized information.

# 5. I am undergoing heart CT. Which is considered more accurate CT or MRI?

Both CT (Computed Tomography) and MRI (Magnetic Resonance Imaging) are highly accurate imaging techniques, but they have different strengths and are used for different purposes when it comes to heart imaging.

**CT for Heart Imaging:**

- **Accuracy for Coronary Artery Disease (CAD):** CT is particularly accurate for evaluating coronary artery disease. A **CT coronary angiogram** is often used to assess blockages or narrowing in the coronary arteries.
- **Calcium Scoring:** CT can also measure calcium deposits in the coronary arteries, which is useful for assessing the risk of heart disease.
- **Speed:** CT scans are faster than MRI, making them more suitable for patients who may have difficulty holding their breath or staying still.
- **Radiation Exposure:** CT involves exposure to ionizing radiation, which is a consideration for some patients.

**MRI for Heart Imaging:**

- **Soft Tissue Detail:** MRI provides superior soft tissue contrast, making it excellent for evaluating heart muscle (myocardium), heart valves, and congenital heart defects.
- **Functional Assessment:** MRI can assess heart function, including how well the heart is pumping (ejection fraction), and can detect scar tissue or inflammation in the heart muscle.
- **No Radiation:** MRI does not use ionizing radiation, which can be beneficial for certain patients, especially those who need repeated imaging.
- **Longer Scan Time:** MRI scans take longer than CT scans, which can be challenging for some patients.

**Which is More Accurate?**

- **For Coronary Arteries:** CT is generally considered more accurate for imaging the coronary arteries and detecting blockages.
- **For Heart Muscle and Function:** MRI is more accurate for assessing heart muscle, valves, and overall heart function.

**Conclusion:**

The choice between CT and MRI depends on what your doctor is looking for. If the focus is on the coronary arteries, CT is typically preferred. If the focus is on heart muscle, valves, or functional assessment, MRI may be more appropriate. Your doctor will choose the best imaging modality based on your specific condition and needs.

# 6. I am undergoing heart MRI. Which is considered more accurate CT or MRI?

Both **CT (Computed Tomography)** and **MRI (Magnetic Resonance Imaging)** are highly advanced imaging techniques, but their accuracy depends on the specific clinical context and what needs to be evaluated in the heart.

**For Heart Imaging:**

- **MRI** is generally considered more accurate for assessing **soft tissue structures**, such as the heart muscle (myocardium), valves, and blood flow. It provides excellent detail without using ionizing radiation.
- **CT** is often better for visualizing **coronary arteries** and detecting **calcifications** or blockages, especially in coronary artery disease (CAD). It is faster and more widely available than MRI.

**Key Differences:**

1. **Soft Tissue Detail**:
   - MRI is superior for evaluating myocardial tissue, scarring (e.g., from a heart attack), inflammation, and congenital heart defects.
   - CT is less detailed for soft tissue but excellent for visualizing coronary artery anatomy.
2. **Radiation Exposure**:
   - MRI does not use ionizing radiation, making it safer for repeated imaging.
   - CT uses X-rays, which involve radiation exposure, though modern CT scanners use low-dose protocols.
3. **Speed and Accessibility**:
   - CT is faster and more widely available, making it better for emergency situations.
   - MRI takes longer but provides more detailed functional and structural information.
4. **Contrast Agents**:
   - Both may use contrast agents, but MRI contrast (gadolinium) is generally safer for patients with kidney issues compared to CT contrast (iodine-based).

**Which is More Accurate?**

- For **structural and functional heart assessment** (e.g., cardiomyopathy, heart failure, or congenital defects), **MRI** is typically more accurate.
- For **coronary artery disease** or **calcium scoring**, **CT** is often preferred.

Your doctor will choose the best imaging modality based on your specific condition and the information needed. If you're undergoing a heart MRI, it’s likely because your doctor needs detailed information about the heart’s structure, function, or tissue characteristics.

# 7. Why would my doctor recommend a cardiac CT instead of a cardiac MRI?

Your doctor might recommend a **cardiac CT (computed tomography)** scan instead of a **cardiac MRI (magnetic resonance imaging)** for several reasons, depending on your specific medical condition, the information needed, and practical considerations. Here are some key factors that could influence this decision:

**1. Purpose of the Imaging**

- **Cardiac CT** is often preferred for evaluating:
  - **Coronary artery disease (CAD):** CT is excellent for detecting calcium deposits (calcium scoring) and visualizing blockages in the coronary arteries (CT angiography).
  - **Anatomy of the heart and blood vessels:** It provides detailed images of the heart's structure, including the aorta and pulmonary arteries.
  - **Pre-surgical planning:** For procedures like valve replacement or ablation, CT can provide precise anatomical details.
- **Cardiac MRI** is better for assessing:
  - **Heart muscle function and tissue characterization:** It can evaluate heart muscle damage, inflammation, or scarring (e.g., from a heart attack or cardiomyopathy).
  - **Congenital heart disease:** MRI is often used for complex heart defects.
  - **Tumors or masses:** MRI provides superior soft tissue contrast.

**2. Speed and Accessibility**

- **Cardiac CT** is faster, often taking only a few minutes to complete. This makes it more suitable for patients who may have difficulty lying still for longer periods or who are in urgent need of diagnosis.
- **Cardiac MRI** takes longer (30–60 minutes or more) and requires the patient to remain still in a confined space, which can be challenging for some individuals.

**3. Patient-Specific Factors**

- **Kidney function:** CT scans typically require iodinated contrast dye, which can affect kidney function. If you have kidney issues, your doctor may prefer MRI, which uses gadolinium-based contrast (though this also has risks in severe kidney disease).
- **Metal implants or devices:** If you have certain metal implants (e.g., pacemakers, defibrillators), MRI may not be safe or feasible. CT is generally safe for patients with metal devices.
- **Claustrophobia:** Some patients find the enclosed MRI machine uncomfortable, whereas CT scanners are more open and less intimidating.

**4. Radiation Exposure**

- **Cardiac CT** involves exposure to ionizing radiation, which may be a concern for some patients, especially if repeated scans are needed.
- **Cardiac MRI** does not use radiation, making it a safer option for certain populations, such as younger patients or those requiring frequent imaging.

**5. Cost and Availability**

- **Cardiac CT** is often more widely available and less expensive than cardiac MRI, which may influence your doctor's recommendation depending on your healthcare system and insurance coverage.

**6. Specific Clinical Questions**

- If your doctor is primarily concerned about **coronary artery blockages**, CT is usually the first-line test.
- If the focus is on **heart muscle function, inflammation, or scarring**, MRI is typically preferred.

Ultimately, the choice between cardiac CT and MRI depends on your individual medical needs, the specific information your doctor is seeking, and practical considerations like availability and patient comfort. Your doctor will weigh these factors to determine the most appropriate test for you.

# 8. Why would my doctor recommend a cardiac MRI instead of a cardiac CT?

Your doctor might recommend a **cardiac MRI** instead of a **cardiac CT** for several reasons, depending on your specific medical condition, the information needed, and the strengths of each imaging modality. Here are some key factors that might influence this decision:

**1. Soft Tissue Detail**

- **Cardiac MRI** provides superior soft tissue contrast, making it ideal for evaluating the heart muscle, valves, and surrounding structures. It is particularly useful for assessing:
  - Myocardial scarring or fibrosis (e.g., from a prior heart attack or cardiomyopathy).
  - Inflammation (e.g., myocarditis).
  - Congenital heart disease.
  - Tumors or masses in the heart.
- **Cardiac CT** is better for visualizing coronary arteries and calcifications but lacks the same level of detail for soft tissue.

**2. No Radiation Exposure**

- **Cardiac MRI** does not use ionizing radiation, making it a safer option for patients who need repeated imaging or who are sensitive to radiation (e.g., younger patients or pregnant women).
- **Cardiac CT** involves exposure to radiation, which may be a concern for some patients.

**3. Assessment of Heart Function**

- **Cardiac MRI** is considered the gold standard for evaluating heart function, including:
  - Ejection fraction (how well the heart pumps blood).
  - Wall motion abnormalities.
  - Blood flow through the heart and vessels.
- While **cardiac CT** can also assess function, it is not as comprehensive as MRI for this purpose.

**4. Evaluation of Specific Conditions**

- **Cardiac MRI** is often preferred for diagnosing or monitoring:
  - Cardiomyopathies (e.g., hypertrophic, dilated, or restrictive cardiomyopathy).
  - Myocardial viability (to determine if damaged heart muscle can recover).
  - Complex congenital heart disease.
  - Pericardial diseases (e.g., constrictive pericarditis).
- **Cardiac CT** is typically used for evaluating coronary artery disease, plaque buildup, and stent or bypass graft patency.

**5. Contrast Agent Considerations**

- **Cardiac MRI** uses gadolinium-based contrast agents, which are generally safer for patients with kidney dysfunction compared to the iodine-based contrast used in **cardiac CT** (though gadolinium has its own risks in certain patients).

**6. Patient-Specific Factors**

- If you have a pacemaker or implantable cardioverter-defibrillator (ICD), **cardiac MRI** may not be recommended unless the device is MRI-compatible.
- If you have claustrophobia or difficulty lying still, **cardiac CT** might be preferred due to its faster imaging time.

**7. Clinical Question**

- If the primary concern is **coronary artery disease**, **cardiac CT** is often the first choice because it provides detailed images of the coronary arteries.
- If the focus is on **heart muscle or structural abnormalities**, **cardiac MRI** is typically more informative.

**Summary**

Your doctor will choose the imaging modality that best addresses your specific clinical needs, taking into account factors like the type of information required, your medical history, and any potential risks. If you're unsure why a cardiac MRI was recommended, don't hesitate to ask your doctor for clarification—they can explain how this choice aligns with your diagnosis or treatment plan.

# 9. How do I know if I’m a good candidate for a cardiac CT?

Determining if you are a good candidate for a cardiac CT (computed tomography) scan depends on several factors, including your medical history, symptoms, and risk factors for heart disease. Here are some key considerations:

**1. Symptoms or Conditions That May Warrant a Cardiac CT:**

- **Chest pain or discomfort:** If you have unexplained chest pain, a cardiac CT can help assess whether coronary artery disease (CAD) is the cause.
- **Abnormal stress test results:** If other tests (like an exercise stress test) suggest possible heart disease, a cardiac CT can provide more detailed information.
- **Intermediate risk of heart disease:** If you have risk factors (e.g., high cholesterol, high blood pressure, smoking, diabetes, or a family history of heart disease) but no clear symptoms, a cardiac CT can help assess your risk.
- **Evaluation of coronary artery anatomy:** If you need detailed imaging of your coronary arteries, such as before non-cardiac surgery or to evaluate congenital heart defects.

**2. Risk Factors for Heart Disease:**

- High cholesterol
- High blood pressure
- Diabetes
- Smoking
- Obesity
- Family history of early heart disease
- Sedentary lifestyle

**3. When a Cardiac CT May Not Be Suitable:**

- **Severe kidney disease:** The contrast dye used in cardiac CT can harm kidneys in people with severe kidney impairment.
- **Allergy to contrast dye:** If you have a known allergy to iodine-based contrast, alternative tests may be recommended.
- **High radiation exposure concerns:** While the radiation dose is relatively low, it may not be recommended if you’ve had multiple other imaging tests involving radiation.
- **Pregnancy:** Radiation exposure is generally avoided during pregnancy unless absolutely necessary.

**4. Age and Gender Considerations:**

- Cardiac CT is often more useful for individuals aged 40–70 with intermediate risk of heart disease.
- For younger individuals or those with very low risk, the test may not be necessary.
- Women with atypical symptoms of heart disease may benefit from cardiac CT to rule out CAD.

**5. Other Diagnostic Tests:**

- If you’ve already had a clear diagnosis from other tests (e.g., coronary angiography, stress test), a cardiac CT may not add significant value.
- Cardiac CT is often used when other tests are inconclusive or when a non-invasive option is preferred.

**Next Steps:**

If you think you might be a good candidate for a cardiac CT, consult your doctor or a cardiologist. They will evaluate your symptoms, medical history, and risk factors to determine if the test is appropriate for you. They may also consider alternative tests, such as an echocardiogram, stress test, or MRI, depending on your specific situation.

Let me know if you’d like more details!

# 10. How do I know if I’m a good candidate for a cardiac MRI?

Determining whether you are a good candidate for a cardiac MRI (magnetic resonance imaging) depends on several factors, including your medical history, symptoms, and the specific information your healthcare provider is seeking. Here are some key considerations:

**1. Medical Indications:**

- **Heart Conditions:** Cardiac MRI is often used to evaluate various heart conditions, such as:
  - Congenital heart disease
  - Cardiomyopathies (e.g., hypertrophic cardiomyopathy, dilated cardiomyopathy)
  - Myocarditis or pericarditis
  - Heart tumors
  - Valvular heart disease
  - Coronary artery disease (e.g., to assess myocardial viability after a heart attack)
  - Arrhythmias (e.g., to assess scar tissue in the heart)
- **Unexplained Symptoms:** If you have symptoms like chest pain, shortness of breath, or palpitations, and other tests (e.g., echocardiogram, stress test) have not provided a clear diagnosis, a cardiac MRI may be recommended.

**2. Previous Test Results:**

- If other imaging tests (e.g., echocardiogram, CT scan) have been inconclusive or if more detailed information is needed, a cardiac MRI might be the next step.

**3. Contraindications:**

- **Metal Implants or Devices:** Certain metal implants or devices (e.g., pacemakers, defibrillators, cochlear implants) may not be compatible with MRI. However, some newer devices are MRI-compatible, so this should be discussed with your doctor.
- **Claustrophobia:** If you have severe claustrophobia, you might find the MRI machine uncomfortable. In some cases, sedation can be used to help you relax.
- **Kidney Function:** If a contrast agent (gadolinium) is needed, your kidney function will be assessed, as gadolinium is not recommended for people with severe kidney impairment.

**4. Allergies:**

- If you have a history of allergies, particularly to contrast agents, this should be discussed with your doctor. Allergic reactions to gadolinium are rare but possible.

**5. Pregnancy:**

- If you are pregnant, the use of MRI should be carefully considered, especially during the first trimester. However, MRI is generally considered safer than CT scans or X-rays because it does not use ionizing radiation.

**6. Body Size:**

- The size of the MRI machine’s opening (bore) can be a limiting factor for some patients, particularly those who are very large or obese. Some facilities have larger bore MRI machines to accommodate larger patients.

**7. Ability to Lie Still:**

- A cardiac MRI requires you to lie still for an extended period (typically 30-60 minutes). If you have difficulty lying still due to pain or other conditions, this might affect the quality of the images.

**8. Cost and Accessibility:**

- Cardiac MRI is a specialized test and may not be available at all medical facilities. Additionally, it can be expensive, so insurance coverage and cost may be considerations.

**Next Steps:**

If you think you might be a candidate for a cardiac MRI, the best course of action is to discuss it with your healthcare provider. They will consider your medical history, symptoms, and the results of any previous tests to determine if a cardiac MRI is appropriate for you.

**Summary:**

- **Good Candidate:** If you have a heart condition that requires detailed imaging, unexplained symptoms, or inconclusive results from other tests.
- **Not a Good Candidate:** If you have incompatible metal implants, severe claustrophobia, or severe kidney impairment (if contrast is needed).

Always consult with your healthcare provider to make an informed decision.

# 11. Do I need to fast before my cardiac CT?

**Yes, you typically need to fast before a cardiac CT scan, especially if the scan involves contrast dye. Fasting helps ensure clear images and reduces the risk of complications, such as nausea or vomiting, which can occur if you have food in your stomach during the procedure.**

**General Fasting Guidelines:**

- **Duration: You are usually asked to fast for 2-4 hours before the scan. Your healthcare provider will give you specific instructions based on the type of cardiac CT and the contrast agent being used.**
- **Medications: You can usually take your regular medications with a small amount of water unless instructed otherwise.**
- **Avoid caffeine and smoking: These can affect your heart rate and may interfere with the test results.**

**Why Fasting is Important:**

1. **Contrast Dye: If contrast dye is used, fasting helps prevent nausea or aspiration (inhaling stomach contents) if you were to vomit.**
2. **Image Clarity: Food in your stomach can sometimes interfere with the quality of the images.**

**Always Follow Your Provider's Instructions:**

**Your healthcare provider will give you specific instructions tailored to your situation. If you have diabetes or other medical conditions, let them know, as they may adjust the fasting requirements or provide additional guidance.**

**If you're unsure, contact the facility performing the scan for clarification.**

# 12. Do I need to fast before my cardiac MRI?

Whether you need to fast before a cardiac MRI depends on the specific instructions from your healthcare provider or the imaging center. In many cases, fasting is not required for a standard cardiac MRI. However, there are some situations where fasting might be necessary:

1. **If contrast dye is used**: If your cardiac MRI involves the use of a contrast agent (gadolinium), you may be asked to fast for 2-4 hours beforehand to reduce the risk of nausea or vomiting.
2. **If sedation is required**: If you are anxious or unable to lie still during the scan, you might be given a sedative. In this case, fasting for a few hours before the procedure is usually recommended.
3. **Specific medical conditions**: If you have certain conditions, such as diabetes or gastrointestinal issues, your doctor may provide specific fasting instructions.

Always follow the instructions given by your healthcare provider or the imaging center. If you're unsure, contact them ahead of time to clarify whether fasting is necessary for your specific situation.

# 13. I am undergoing cardiac CT. Can I take my usual medications on the morning of the test?

Yes, you can generally take your usual medications on the morning of a cardiac CT scan, but there are some exceptions. Here are some key points to consider:

1. **Diabetes Medications**: If you take medications for diabetes, particularly metformin, you may need to adjust your dose or temporarily stop taking it. This is because the contrast dye used in the CT scan can affect kidney function, and metformin is cleared by the kidneys. Your healthcare provider will give you specific instructions.
2. **Beta-Blockers**: Sometimes, a beta-blocker is given before a cardiac CT to lower your heart rate, which helps improve the quality of the images. If your heart rate is naturally low or if you are already on a beta-blocker, your doctor will advise you on whether to take your usual dose.
3. **Other Medications**: Most other medications, including those for blood pressure, cholesterol, and other conditions, can usually be taken as normal. However, it's always best to confirm with your healthcare provider.
4. **Contrast Dye**: If you have a known allergy to contrast dye or have kidney issues, inform your healthcare provider. They may give you specific instructions or prescribe medications to reduce the risk of an allergic reaction.

Always consult with your healthcare provider or the imaging center for personalized advice based on your medical history and the specific requirements of the test.

# 14. I am undergoing cardiac MRI. Can I take my usual medications on the morning of the test?

Yes, you can generally take your usual medications on the morning of a cardiac MRI unless your healthcare provider instructs you otherwise. However, there are a few considerations:

1. **Contrast Agents**: If your cardiac MRI involves the use of a contrast agent (e.g., gadolinium), inform your doctor about all medications you are taking, especially if you have kidney problems, as this can affect the safety of contrast use.
2. **Beta-Blockers**: Sometimes, beta-blockers are given before a cardiac MRI to slow the heart rate and improve image quality. If you are already on a beta-blocker, your doctor may adjust the timing or dosage.
3. **Specific Instructions**: Always follow any specific instructions provided by your healthcare team. If you are unsure, contact them for clarification.
4. **Diabetes Medications**: If you have diabetes and are taking medications like metformin, your doctor may advise you to temporarily stop taking it before and after the MRI if contrast is used, due to the risk of a rare condition called metabolic acidosis.

Always communicate with your healthcare provider about your medications and any concerns you have before the test.

# 15. I am undergoing cardiac CT. Are there any dietary restrictions before the test?

Yes, there are typically dietary restrictions before a cardiac CT scan, especially if the test involves contrast dye. Here are some general guidelines:

1. **Fasting**: You may be asked to avoid eating or drinking for a few hours before the test, usually 2-4 hours. This helps ensure clear images and reduces the risk of nausea from the contrast dye.
2. **Caffeine and Stimulants**: Avoid caffeine (coffee, tea, energy drinks) and smoking for at least 24 hours before the test, as these can affect your heart rate and interfere with the results.
3. **Medications**: Inform your doctor about any medications you're taking. You may need to adjust or temporarily stop certain medications, such as beta-blockers or nitrates, if they affect your heart rate.
4. **Contrast Dye**: If your test involves contrast dye, let your healthcare provider know if you have any allergies, especially to iodine or shellfish, or if you have kidney problems.

Always follow the specific instructions provided by your healthcare team, as requirements may vary depending on the type of cardiac CT and your medical history.

# 16. I am undergoing cardiac MRI. Are there any dietary restrictions before the test?

When preparing for a cardiac MRI, dietary restrictions can vary depending on the specific protocols of the imaging center and whether contrast material will be used. Here are some general guidelines:

1. **Fasting**: Some centers may ask you to avoid eating or drinking for a few hours (typically 2-4 hours) before the test, especially if contrast dye is being used. This helps reduce the risk of nausea or vomiting.
2. **Caffeine and Stimulants**: You may be advised to avoid caffeine (coffee, tea, energy drinks) and other stimulants for 12-24 hours before the test, as they can affect heart rate and imaging results.
3. **Medications**: Continue taking your prescribed medications unless your doctor instructs otherwise. If you’re unsure, ask your healthcare provider.
4. **Contrast Agents**: If contrast dye is being used, inform your doctor if you have kidney problems or allergies to contrast materials. You may need blood tests to check kidney function beforehand.
5. **Hydration**: Staying hydrated is generally recommended, but follow any specific instructions from your healthcare provider.

Always confirm with your doctor or the imaging center for their specific requirements, as protocols can vary.

# 17. I am undergoing heart CT. Will I need blood tests or other preliminary checks beforehand?

Yes, before undergoing a heart CT scan, particularly a Coronary CT Angiogram (CTA), you may need some preliminary checks, including blood tests, to ensure your safety and the accuracy of the results. Here’s what you might expect:

**1. Blood Tests**

- **Kidney Function Test (Serum Creatinine):** A heart CT scan often involves the use of iodinated contrast dye, which is cleared by the kidneys. If your kidney function is impaired, the dye could cause complications. A blood test to check your creatinine levels helps assess kidney function.
- **Thyroid Function Test (if applicable):** The contrast dye can affect the thyroid, especially if you have a history of thyroid disorders.

**2. Other Preliminary Checks**

- **Medical History Review:** Your doctor will review your medical history, including any allergies (especially to contrast dye), heart conditions, or other health issues.
- **Medication Review:** Certain medications, like metformin (for diabetes), may need to be temporarily stopped before the scan due to the contrast dye.
- **Pregnancy Test (if applicable):** If you are of childbearing age, you may be asked to take a pregnancy test to rule out pregnancy, as radiation exposure can harm a developing fetus.
- **Heart Rate and Blood Pressure:** For a Coronary CTA, your heart rate needs to be slow and steady (usually below 65 beats per minute). You may be given medication (e.g., beta-blockers) to lower your heart rate if necessary.

**3. Fasting**

- You may be asked to fast for a few hours before the scan, especially if contrast dye will be used, to reduce the risk of nausea or vomiting.

**4. Allergy Precautions**

- If you have a known allergy to contrast dye, your doctor may prescribe medications (e.g., antihistamines or steroids) to reduce the risk of an allergic reaction.

Always follow your healthcare provider’s specific instructions, as requirements can vary depending on your individual health and the type of CT scan being performed.

# 18. I am undergoing heart MRI. Will I need blood tests or other preliminary checks beforehand?

When preparing for a heart MRI (magnetic resonance imaging), your healthcare provider may require certain preliminary checks to ensure your safety and optimize the results of the scan. Here's what you might expect:

**1. Blood Tests**

- **Kidney Function Test**: If the MRI involves the use of a contrast dye (gadolinium), your doctor may order a blood test to check your kidney function. This is because the dye is processed by the kidneys, and impaired kidney function could increase the risk of complications.
- **Other Blood Tests**: Depending on your medical history, additional blood tests (e.g., to check for anemia or infections) might be necessary.

**2. Medical History Review**

- Your doctor will review your medical history, including any allergies, prior reactions to contrast dye, or conditions like kidney disease.
- Inform them if you have any implanted medical devices (e.g., pacemakers, defibrillators, or metal implants), as these may affect your eligibility for an MRI.

**3. Pregnancy Screening**

- If you are or could be pregnant, let your doctor know. While MRI is generally considered safe during pregnancy, the use of contrast dye is typically avoided unless absolutely necessary.

**4. Allergy Assessment**

- If you have a history of allergies, especially to contrast agents, your doctor may take precautions or prescribe medications to reduce the risk of a reaction.

**5. Pre-MRI Instructions**

- You may be asked to avoid eating or drinking for a few hours before the scan, particularly if contrast dye will be used.
- Wear comfortable, metal-free clothing, and remove any jewelry or accessories.

**6. Other Tests**

- In some cases, your doctor may order additional tests, such as an ECG (electrocardiogram), to assess your heart's electrical activity before the MRI.

Always follow your healthcare provider's specific instructions, as requirements can vary based on your individual health and the reason for the MRI. If you have any concerns, discuss them with your doctor beforehand.

# 19. I am undergoing heart CT. What should I wear to the appointment?

When preparing for a heart CT scan, it's important to wear comfortable, loose-fitting clothing. Here are some specific recommendations:

1. **Avoid Metal**: Wear clothing without metal zippers, buttons, or snaps, as metal can interfere with the imaging process. Opt for sweatpants, a t-shirt, or a sweatshirt made of soft, stretchy fabric.
2. **Comfortable Clothing**: Choose something you can easily change out of if needed, as you may be asked to wear a hospital gown.
3. **Remove Jewelry**: Leave all jewelry, including piercings, at home, as metal objects need to be removed before the scan.
4. **Footwear**: Wear comfortable shoes that are easy to slip on and off, as you may need to remove them during the procedure.
5. **Bra Considerations**: If you wear a bra, choose one without an underwire or metal clasps, or be prepared to remove it.

The medical facility may provide a gown if your clothing is not suitable. Always follow any specific instructions given by your healthcare provider or the imaging center.

# 20. I am undergoing heart MRI. What should I wear to the appointment?

When preparing for a heart MRI, it's important to wear comfortable, loose-fitting clothing without metal components, as metal can interfere with the MRI machine. Here are some specific recommendations:

1. **Avoid Metal**: Do not wear clothing with zippers, snaps, buttons, or underwire bras, as these often contain metal.
2. **Comfortable Clothing**: Opt for sweatpants, yoga pants, or loose-fitting pants and a t-shirt or sweatshirt without metal.
3. **Remove Accessories**: Leave jewelry, watches, belts, and other accessories at home or be prepared to remove them before the scan.
4. **Footwear**: Wear socks and slip-on shoes without metal, as you may be asked to remove your shoes.
5. **Hospital Gown**: If your clothing is not suitable, you may be asked to change into a hospital gown.

Additionally, inform the MRI technologist if you have any implants, pacemakers, or metal in your body, as these can affect the procedure. They will guide you on what to do.

# 21. I am undergoing heart CT. Do I need to avoid strenuous activity or exercise before the test?

For a heart CT scan, particularly a coronary CT angiography (CTA), your healthcare provider may give you specific instructions to follow before the test. While strenuous activity or exercise is not typically a major concern, here are some general guidelines:

1. **Medications**: You may be asked to avoid certain medications, such as beta-blockers, unless prescribed for the test. These medications help slow your heart rate for clearer imaging.
2. **Caffeine and Stimulants**: Avoid caffeine (coffee, tea, energy drinks) and other stimulants for 12-24 hours before the test, as they can increase your heart rate.
3. **Fasting**: You might need to fast for a few hours before the scan, especially if contrast dye will be used.
4. **Strenuous Activity**: While moderate activity is usually fine, avoid intense exercise immediately before the test, as it can elevate your heart rate and affect the results.
5. **Clothing**: Wear comfortable clothing and remove any metal objects, as they can interfere with the imaging.

Always follow the specific instructions provided by your healthcare team, as requirements can vary depending on the type of CT scan and your individual health condition. If you're unsure, contact your doctor or the imaging center for clarification.

# 22. I am undergoing heart MRI. Do I need to avoid strenuous activity or exercise before the test?

Yes, it's generally recommended to avoid strenuous activity or exercise before a heart MRI. Here’s why:

1. **Heart Rate and Rhythm**: Strenuous activity can elevate your heart rate and potentially affect the MRI results, as the test aims to capture clear images of your heart at rest.
2. **Image Quality**: Excessive movement or an elevated heart rate can make it harder to obtain clear, high-quality images.
3. **Breathing Patterns**: Heavy exercise might affect your breathing, which can interfere with the MRI process, especially if breath-holding is required during the scan.

**General Guidelines:**

- **Light Activity**: Light activities, such as walking, are usually fine.
- **Avoid Heavy Exercise**: Avoid vigorous exercise for at least 24 hours before the test.
- **Follow Instructions**: Always follow any specific instructions provided by your healthcare provider or the imaging center.

If you have any concerns or questions, consult your doctor or the medical team performing the MRI. They can provide personalized advice based on your health condition.

# 23. I am undergoing heart CT. Should I arrive earlier than my scheduled time, and how long will the overall visit take?

Yes, it's generally a good idea to arrive earlier than your scheduled appointment time for a heart CT scan. Arriving 15-30 minutes early allows time for check-in, completing any necessary paperwork, and preparing for the procedure.

**How Long Will the Visit Take?**

- **Preparation**: This may include changing into a hospital gown, removing metal objects, and possibly receiving a contrast dye via an IV. This process can take 15-30 minutes.
- **Scan**: The actual CT scan typically takes only a few minutes, often less than 10 minutes.
- **Post-Scan**: If contrast dye was used, you might be monitored briefly to ensure there are no adverse reactions. This can add another 10-15 minutes.

**Total Time**

- **Without complications**: The entire visit usually takes about 30 minutes to an hour.
- **With complications or delays**: It could take longer, but this is less common.

Always follow any specific instructions provided by your healthcare provider, such as fasting or adjusting medications, to ensure the procedure goes smoothly.

# 24. I am undergoing heart MRI. Should I arrive earlier than my scheduled time, and how long will the overall visit take?

Yes, it’s generally a good idea to arrive earlier than your scheduled MRI appointment, typically around **30 minutes to an hour** in advance. This allows time for check-in, completing any necessary paperwork, and preparing for the procedure.

The **overall visit** can take anywhere from **1 to 2 hours**, depending on the specifics of your MRI. The scan itself usually lasts between **30 to 60 minutes**, but additional time may be needed for preparation, such as changing into a gown, removing metal objects, and discussing the procedure with the technologist. If contrast dye is used, this may also extend the time slightly.

Be sure to follow any pre-scan instructions provided by your healthcare provider, such as fasting or avoiding certain medications. If you have any concerns or questions, contact the imaging center ahead of time for clarification.

# 25. I am undergoing heart CT. Will an IV line for contrast dye be necessary, and how is it administered?

Yes, an IV line is typically necessary for a heart CT scan if contrast dye is used. The contrast dye enhances the visibility of your heart and blood vessels, allowing for more detailed images.

**How the IV Line is Administered:**

1. **Preparation**: A healthcare provider will insert a small IV catheter into a vein, usually in your arm.
2. **Contrast Injection**: During the scan, the contrast dye is injected through the IV line. You may feel a warm sensation or a metallic taste in your mouth, which is normal and temporary.
3. **Monitoring**: The medical team will monitor you for any reactions to the contrast dye, though serious reactions are rare.

**What to Expect:**

- The IV insertion is quick and may cause slight discomfort.
- The contrast dye is administered automatically during the scan.
- The entire process is closely supervised to ensure safety.

If you have concerns about kidney function or allergies, inform your healthcare provider beforehand, as these factors may affect the use of contrast dye.

# 26. I am undergoing heart MRI. Will an IV line for contrast dye be necessary, and how is it administered?

Yes, an IV line is typically necessary for a heart MRI if contrast dye (gadolinium-based) is required. The contrast dye helps enhance the visibility of blood vessels, heart structures, and any abnormalities. Here’s how it’s administered:

1. **IV Placement**: Before the MRI, a nurse or technician will insert an IV line, usually in your arm or hand.
2. **Contrast Injection**: During the MRI, the contrast dye is injected through the IV line. You might feel a cool sensation or a metallic taste briefly, which is normal.
3. **Imaging**: The MRI continues to capture detailed images of your heart with the contrast dye highlighting specific areas.

The procedure is generally safe, but inform the medical team if you have kidney issues or allergies, as these may affect the use of contrast dye.

# 27. I am undergoing heart CT. Can I drive myself to and from the scan, or do I need someone to accompany me?

Whether you can drive yourself to and from a heart CT scan depends on several factors, including the type of scan, whether contrast dye is used, and how you feel afterward. Here's what to consider:

1. **Type of Scan**: If it's a standard CT scan without contrast dye, you should be fine to drive yourself unless you feel unwell or anxious.
2. **Use of Contrast Dye**: If contrast dye is used, some people may feel lightheaded, nauseated, or experience other side effects afterward. In such cases, it's safer to have someone accompany you to drive you home.
3. **Sedation**: If sedation or medication is administered (which is rare for a standard heart CT), you will not be allowed to drive afterward and will need someone to take you home.
4. **Your Health Condition**: If you have symptoms like dizziness, chest pain, or fatigue, it's better to have someone drive you.
5. **Clinic/Hospital Policy**: Some facilities have policies requiring patients to have a companion after certain procedures. Check with your healthcare provider beforehand.

**Recommendation**: If contrast dye is used or you're unsure how you'll feel after the scan, arrange for someone to drive you. If it's a straightforward scan and you feel well, driving yourself is usually fine. Always confirm with your doctor or the imaging center.

# 28. I am undergoing heart MRI. Can I drive myself to and from the scan, or do I need someone to accompany me?

Whether you can drive yourself to and from a heart MRI depends on several factors, including whether you will receive sedation or contrast dye, and how you typically feel after medical procedures. Here’s a breakdown to help you decide:

1. **No Sedation or Contrast Dye**: If your MRI does not involve sedation or contrast dye, and you feel comfortable and alert, you should be able to drive yourself to and from the appointment.
2. **Contrast Dye**: If contrast dye is used, some people may feel mild side effects like dizziness, nausea, or fatigue. If you’ve had contrast dye before and didn’t experience any issues, you might still be fine to drive. However, if you’re unsure or have had adverse reactions in the past, it’s safer to have someone accompany you.
3. **Sedation**: If you are given a sedative (e.g., for anxiety or claustrophobia), you will not be allowed to drive afterward. Sedation can impair your reflexes and judgment, making it unsafe to operate a vehicle. In this case, you must arrange for someone to drive you home.
4. **Personal Comfort**: Even if sedation or contrast dye isn’t used, consider how you typically feel after medical procedures. If you tend to feel fatigued, anxious, or unwell, it’s better to have someone accompany you.

**Best Practice**: Check with your healthcare provider or the imaging center beforehand. They can give you specific guidelines based on your situation. If there’s any doubt, it’s always safer to have a friend or family member drive you

# 29. I am undergoing heart CT. Do I need to let my doctor know about allergies?

Yes, it's crucial to inform your doctor about any allergies before undergoing a heart CT scan. This includes allergies to:

1. **Iodine or Contrast Dye**: Many CT scans use a contrast dye containing iodine to enhance imaging. If you have a known allergy to iodine or contrast dye, your doctor may take precautions or use an alternative imaging method.
2. **Medications**: Inform your doctor about any allergies to medications, especially if you’ve had reactions to medications used during imaging procedures.
3. **Shellfish**: While the connection is rare, some people with shellfish allergies may also react to iodine-based contrast. Let your doctor know if you have this allergy.
4. **Other Allergies**: Mention any other allergies, as they could be relevant to medications or materials used during the procedure.

Your doctor may prescribe medications (like antihistamines or steroids) to reduce the risk of an allergic reaction or choose a different imaging method if necessary. Always provide a complete medical history to ensure your safety.

# 30. I am undergoing heart MRI. Do I need to let my doctor know about allergies?

Yes, it's crucial to inform your doctor about any allergies before undergoing a heart MRI. Here’s why:

1. **Contrast Dye**: Some MRI scans use a contrast dye (gadolinium-based) to enhance images. If you have a history of allergies, especially to contrast agents, iodine, or other substances, your doctor needs to know to assess the risk of an allergic reaction.
2. **Medications**: If you have severe allergies, your doctor might prescribe medications (e.g., antihistamines or steroids) to reduce the risk of a reaction before the scan.
3. **Other Allergies**: Inform your doctor about any other allergies, such as to metals (e.g., nickel) or latex, as these might be relevant to the MRI environment or equipment.

Always provide a complete medical history, including allergies, to ensure your safety during the procedure.

# 31. What exactly happens during a cardiac CT scan?

A cardiac CT (computed tomography) scan is a non-invasive imaging procedure used to visualize the heart and its blood vessels. Here's a step-by-step overview of what typically happens during the procedure:

**1. Preparation**

- **Fasting**: You may be asked to avoid eating or drinking for a few hours before the scan.
- **Medication Adjustment**: Inform your doctor about any medications you’re taking. You might need to temporarily stop certain medications, especially beta-blockers, to ensure accurate results.
- **Contrast Dye**: A contrast dye (iodine-based) is often used to enhance the visibility of the heart and blood vessels. You may be asked about any allergies, particularly to iodine or shellfish, to ensure you can safely receive the dye.
- **Clothing**: You’ll likely be asked to wear a hospital gown and remove any metal objects, such as jewelry, that could interfere with the imaging.

**2. During the Scan**

- **Positioning**: You’ll lie down on a narrow table that slides into the CT scanner, which is a large, doughnut-shaped machine.
- **Electrodes**: Electrodes may be placed on your chest to monitor your heart rate during the scan.
- **Breathing Instructions**: You’ll be given instructions on when to hold your breath, usually for about 10-15 seconds, to minimize movement and ensure clear images.
- **Contrast Injection**: If contrast dye is used, it will be injected into a vein in your arm. You might feel a warm sensation or a metallic taste in your mouth, which is normal.
- **Scanning**: The CT scanner will rotate around you, taking multiple X-ray images from different angles. The entire process usually takes about 10-15 minutes.

**3. After the Scan**

- **Monitoring**: You may be monitored for a short period to ensure there are no adverse reactions to the contrast dye.
- **Hydration**: Drinking plenty of fluids can help flush the contrast dye from your system.
- **Resume Normal Activities**: Most people can resume their normal activities immediately after the scan.

**4. Results**

- The images captured during the scan are reconstructed by a computer to create detailed cross-sectional views of the heart and blood vessels.
- A radiologist will analyze the images and send a report to your doctor, who will discuss the findings with you.

**Risks and Considerations**

- **Radiation Exposure**: Cardiac CT involves exposure to a small amount of radiation, though the benefits usually outweigh the risks.
- **Contrast Reactions**: Some people may experience allergic reactions to the contrast dye, though this is rare.
- **Kidney Function**: The contrast dye can affect kidney function, particularly in individuals with pre-existing kidney conditions.

A cardiac CT scan is a valuable tool for diagnosing and evaluating various heart conditions, including coronary artery disease, congenital heart defects, and other cardiovascular issues. Always follow your healthcare provider's instructions and discuss any concerns you may have before the procedure.

# 32. What exactly happens during a cardiac MRI scan?

A cardiac MRI (Magnetic Resonance Imaging) scan is a non-invasive imaging test that provides detailed pictures of the heart and surrounding structures using a powerful magnetic field, radio waves, and a computer. Here’s a step-by-step overview of what typically happens during a cardiac MRI scan:

**1. Preparation:**

- **Clothing:** You may be asked to change into a hospital gown and remove any metal objects (jewelry, watches, etc.) to avoid interference with the magnetic field.
- **Screening:** You’ll be screened for any metal implants or devices (e.g., pacemakers, cochlear implants) that could be affected by the MRI. Some implants are MRI-safe, but others may preclude you from having the scan.
- **Contrast Agent:** In some cases, a contrast agent (gadolinium-based) may be injected into a vein to enhance the images. This helps to highlight blood vessels, scar tissue, or other abnormalities.

**2. Positioning:**

- You’ll lie down on a sliding table that moves into the MRI machine. The machine is a large, cylindrical tube with openings at both ends.
- Electrodes may be placed on your chest to monitor your heart rate during the scan. This helps synchronize the imaging with your heartbeat (a technique called ECG-gating).

**3. Entering the MRI Machine:**

- The table will slide into the MRI machine, which is typically narrow and enclosed. If you’re uncomfortable in tight spaces, inform the technologist; they may offer a mild sedative or use an open MRI machine if available.
- You’ll need to remain as still as possible during the scan to ensure clear images.

**4. During the Scan:**

- **Magnetic Field and Radio Waves:** The MRI machine creates a strong magnetic field around you, and radio waves are directed at your body. These interact with the hydrogen atoms in your body, particularly in water and fat.
- **Image Acquisition:** The machine detects the signals emitted by the hydrogen atoms and uses them to create detailed cross-sectional images of your heart and surrounding structures.
- **Breathing Instructions:** You may be asked to hold your breath for short periods (10-20 seconds) to minimize motion artifacts and improve image quality.
- **Noises:** The MRI machine makes loud knocking or buzzing sounds during the scan. You’ll likely be given earplugs or headphones to protect your hearing and possibly listen to music.

**5. Duration:**

- A cardiac MRI typically takes between 30 minutes to 1.5 hours, depending on the complexity of the images needed.

**6. After the Scan:**

- Once the scan is complete, the table will slide out of the machine, and you can get dressed.
- If a contrast agent was used, you may be monitored briefly for any adverse reactions, though these are rare.
- You can usually resume normal activities immediately after the scan.

**7. Results:**

- The images will be analyzed by a radiologist or cardiologist, who will interpret the findings and send a report to your referring physician. Your doctor will discuss the results with you and determine any necessary next steps.

**Key Points:**

- **Safety:** MRI does not use ionizing radiation, making it a safe option for most people. However, it’s important to inform the technologist of any metal implants or conditions that could affect the scan.
- **Comfort:** The procedure is painless, but some people may find the confined space or loud noises uncomfortable.

Cardiac MRI is a powerful tool for diagnosing and evaluating a wide range of heart conditions, including congenital heart disease, cardiomyopathies, tumors, and scarring from heart attacks.

# 33. I am undergoing heart CT. Do I need to stay completely still or hold my breath at certain times?

Yes, during a heart CT scan, you will need to stay as still as possible and may be asked to hold your breath briefly at certain times. Here's why:

1. **Staying Still**: Movement can blur the images, making it harder for the radiologist to interpret the results accurately. Even small movements can affect the quality of the scan.
2. **Holding Your Breath**: You may be asked to hold your breath for 10-20 seconds during the scan. This helps reduce motion caused by breathing, which can also blur the images. The technician will guide you on when to hold your breath and when you can breathe normally.

The technician will give you clear instructions throughout the procedure, so follow their guidance closely. If you have any concerns or difficulties, such as trouble holding your breath, let them know beforehand so they can adjust the process if needed.

# 34. I am undergoing heart MRI. Do I need to stay completely still or hold my breath at certain times?

Yes, during a heart MRI, it's important to follow the instructions given by the technologist to ensure clear and accurate images. Here’s what you can generally expect:

1. **Staying Still**: You will need to remain as still as possible throughout the procedure. Any movement can blur the images and make them harder to interpret.
2. **Holding Your Breath**: At certain times, you may be asked to hold your breath for short periods, typically around 10-20 seconds. This helps to reduce motion artifacts caused by breathing, which can be particularly important when imaging the heart.
3. **Breathing Normally**: There will also be times when you can breathe normally, but you should still try to keep your body as still as possible.
4. **Communication**: The MRI machine is equipped with a communication system, so you can hear the technologist and they can hear you. They will guide you through the process, letting you know when to hold your breath and when you can relax.
5. **Comfort**: If you feel uncomfortable or anxious at any point, let the technologist know. They can provide reassurance or make adjustments to help you feel more at ease.

Following these instructions carefully will help ensure the best possible results from your heart MRI.

# 35. I am undergoing heart CT. Is the procedure painful or uncomfortable in any way?

A heart CT (computed tomography) scan is generally not painful, but some people may find certain aspects of the procedure slightly uncomfortable. Here’s what you can expect:

1. **Lying Still**: You’ll need to lie still on a table for the duration of the scan, which can take 10–30 minutes. Some people find this position uncomfortable, especially if they have back pain or difficulty lying flat.
2. **Contrast Dye**: If your scan involves a contrast dye (often used to highlight blood vessels and heart structures), you may feel:
   - A warm sensation or metallic taste in your mouth as the dye is injected.
   - A brief feeling of flushing or nausea, which usually passes quickly.
3. **IV Insertion**: If contrast dye is used, a small needle will be inserted into your vein to administer it. This may cause minor discomfort, similar to a standard blood draw.
4. **Breath-Holding**: You may be asked to hold your breath for short periods during the scan to ensure clear images. Some people find this slightly challenging.
5. **Noise**: The CT machine makes buzzing or clicking noises, which can be unsettling for some people, but earplugs or headphones may be provided to help.

Overall, the procedure is quick, non-invasive, and well-tolerated by most people. If you’re anxious, let the technologist know—they can help you feel more comfortable.

# 36. I am undergoing heart MRI. Is the procedure painful or uncomfortable in any way?

A heart MRI (Magnetic Resonance Imaging) is generally not painful, but there are some aspects of the procedure that might cause discomfort for certain individuals. Here’s what you can expect:

1. **Lying Still**: You’ll need to lie still on a narrow table for the duration of the scan, which can take 30 to 60 minutes or longer. Some people find this uncomfortable, especially if they have difficulty staying still for extended periods.
2. **Confined Space**: The MRI machine is a large, tube-like structure. If you’re claustrophobic, you might feel anxious or uncomfortable being inside the machine. Open MRI machines are available in some facilities and may be an option if you’re concerned about this.
3. **Noise**: The MRI machine makes loud knocking or buzzing sounds during the scan. You’ll be given earplugs or headphones to help reduce the noise, but some people still find it unsettling.
4. **Contrast Dye**: If your MRI requires a contrast dye (a special substance that helps highlight certain areas of the heart), it will be injected into a vein. You might feel a cool sensation or a slight pinch when the needle is inserted, but the dye itself is usually not painful.
5. **Breath-Holding**: During the scan, you may be asked to hold your breath for short periods to get clear images of your heart. This can be slightly uncomfortable but is usually manageable.
6. **Temperature**: The MRI room can be cool, and some people feel a bit cold lying still for so long. You may be offered a blanket to help with this.

If you’re particularly anxious about the procedure, let your healthcare team know. They may offer strategies to help you relax, such as playing music, providing a mild sedative, or explaining the process in more detail to ease your concerns.

Overall, while the MRI itself is not painful, the experience can be uncomfortable for some people due to the factors mentioned above. Most individuals tolerate the procedure well, and the benefits of obtaining detailed images of your heart usually outweigh the temporary discomfort

# 37. I am undergoing heart CT. Can I listen to music or communicate with the technician during the scan?

Yes, you can often listen to music during a heart CT scan, but communication with the technician may be limited. Here's what to expect:

1. **Listening to Music**: Many facilities allow you to listen to music through headphones to help you relax. You may need to use their equipment to avoid interference with the imaging process. Let the technician know beforehand if you'd like to listen to music.
2. **Communication During the Scan**: The technician will give you instructions before the scan begins, such as when to hold your breath. During the scan, you can communicate if necessary, but movement and talking should be minimized to ensure clear images. The technician will monitor you and can stop the scan if needed.

Always follow the specific instructions provided by your healthcare team to ensure the best results.

# 38. I am undergoing heart MRI. Can I listen to music or communicate with the technician during the scan?

Yes, you can often listen to music during a heart MRI, and you can communicate with the technician as needed. Here’s what you need to know:

1. **Listening to Music**: Many MRI facilities allow you to listen to music through headphones or earplugs to help you relax during the scan. The equipment used is MRI-safe, so it won’t interfere with the imaging process. You may be able to choose your own music or select from a provided playlist.
2. **Communication with the Technician**: You will be able to communicate with the MRI technician throughout the scan. They will provide you with a microphone or headset to talk to them, and they can hear you as well. It’s important to let them know if you feel uncomfortable, anxious, or experience any issues during the procedure.
3. **Stay Still**: While you can communicate and listen to music, it’s crucial to remain as still as possible during the scan to ensure clear images. The technician will guide you on when you need to hold your breath or stay still.
4. **Noise During the Scan**: MRI machines can be loud, producing knocking or buzzing sounds. The headphones or earplugs provided will help reduce this noise and make the experience more comfortable.

Always follow the specific instructions given by your healthcare provider or the MRI staff to ensure a smooth and safe procedure.

# 39. I am undergoing heart CT. What if I start feeling anxious or need to stop the test?

It's completely normal to feel anxious during a heart CT scan, especially if you're unfamiliar with the procedure or are concerned about the results. Here’s what you can do if you start feeling anxious or need to stop the test:

**1. Communicate with the Technologist**

- The technologist operating the CT scanner is there to assist you. If you feel anxious or uncomfortable at any point, let them know immediately. They can pause the procedure, provide reassurance, or take steps to make you feel more at ease.

**2. Use Relaxation Techniques**

- Before the scan, practice deep breathing or other relaxation techniques to help calm your nerves. During the test, focus on slow, steady breaths to reduce anxiety.

**3. Ask Questions**

- If you’re unsure about any part of the procedure, ask the technologist to explain what’s happening. Knowing what to expect can help reduce anxiety.

**4. Request a Break**

- If you feel overwhelmed, you can ask for a short break. The technologist can pause the scan (if possible) and give you time to regroup.

**5. Bring a Support Person**

- If allowed, having a friend or family member nearby can provide comfort and reassurance. Check with the facility beforehand to see if this is permitted.

**6. Discuss Sedation Options**

- If you’re extremely anxious, talk to your doctor before the test. They may prescribe a mild sedative to help you relax during the procedure.

**7. Remember You’re in Control**

- While the technologist will guide you through the process, you have the right to stop the test if you feel too uncomfortable. Let them know, and they will respect your decision.

**8. Focus on the Benefits**

- Remind yourself that the CT scan is an important diagnostic tool to assess your heart health. The procedure is quick, and the information gained can be crucial for your care.

**What Happens During a Heart CT Scan?**

- You’ll lie on a table that slides into the CT scanner.
- The scanner uses X-rays to create detailed images of your heart.
- You may be given a contrast dye through an IV to enhance the images.
- The technologist will guide you through breathing instructions to ensure clear images.

The entire process is usually quick (often less than 15 minutes), and the technologist will be in constant communication with you. If you feel anxious, don’t hesitate to speak up—they’re there to help!

# 40. I am undergoing heart MRI. What if I start feeling anxious or need to stop the test?

It's completely normal to feel anxious during an MRI, especially in a confined space like the MRI machine. Here’s what you can do if you start feeling anxious or need to stop the test:

1. **Communicate with the Technologist**: Before the test begins, you’ll be given a call button or squeeze ball to hold. If you feel anxious or uncomfortable at any point, press the button or squeeze the ball to alert the MRI technologist. They can hear you and will stop the scan to check on you.
2. **Breathing Techniques**: If you feel anxious, try slow, deep breathing. Inhale deeply through your nose, hold for a few seconds, and exhale slowly through your mouth. This can help calm your nerves.
3. **Earplugs or Headphones**: The MRI machine can be loud, which might contribute to anxiety. Ask for earplugs or headphones with music to help distract you and reduce noise.
4. **Ask for Updates**: If you’re feeling claustrophobic or anxious, ask the technologist to give you updates on how much longer the scan will take. Knowing there’s only a few minutes left can help you push through.
5. **Bring a Support Person**: If allowed, having a friend or family member nearby can provide comfort. They may not be able to stay in the room during the scan, but knowing they’re close by can help.
6. **Discuss Sedation Options**: If you’re extremely anxious, talk to your doctor beforehand. They may prescribe a mild sedative to help you relax during the procedure.
7. **Remember It’s Temporary**: The MRI scan is usually short (20-60 minutes), and you can stop it at any time if needed. Focus on the fact that it’s a temporary situation.
8. **Close Your Eyes**: If the confined space bothers you, keep your eyes closed before and during the scan. This can help reduce the feeling of being enclosed.
9. **Ask About Open MRI**: If you’re claustrophobic, ask your doctor if an open MRI machine is an option. These are less enclosed and may be more comfortable for some people.

Your comfort and safety are the top priorities, so don’t hesitate to communicate with the MRI staff. They’re there to help you through the process

# 41. I am undergoing Heart CT. Is there any special monitoring or sedation used during this scan?

During a Heart CT (computed tomography) scan, also known as a coronary CT angiography (CTA), special monitoring and sedation are generally not required. However, there are a few key points to be aware of:

1. **Monitoring**:
   - Your heart rate and rhythm may be monitored during the scan because a steady, slow heart rate is ideal for obtaining clear images. If your heart rate is too high, you might be given a medication (like a beta-blocker) to slow it down.
   - You will be connected to an ECG (electrocardiogram) machine to synchronize the CT scan with your heartbeats.
2. **Sedation**:
   - Sedation is typically not needed for a Heart CT scan. The procedure is non-invasive and usually takes only a few minutes.
   - You will need to lie still on the CT table, and you may be asked to hold your breath for short periods to minimize motion and ensure clear images.
3. **Contrast Dye**:
   - A contrast dye (iodine-based) is often injected into a vein to highlight the coronary arteries. You might feel a warm sensation or a metallic taste in your mouth when the dye is injected, but this is normal and temporary.
   - Inform the technologist if you have any allergies, especially to iodine or contrast dye, or if you have kidney problems, as the dye can affect kidney function.
4. **Preparation**:
   - You may be asked to avoid caffeine and smoking for a few hours before the scan, as these can increase your heart rate.
   - Follow any specific instructions given by your healthcare provider regarding medications or fasting.

If you have any concerns or specific medical conditions, discuss them with your healthcare provider or the radiology team before the scan. They will ensure you are well-prepared and comfortable throughout the procedure

# 42. I am undergoing Heart MRI. Is there any special monitoring or sedation used during this scan?

During a Heart MRI (also known as Cardiac MRI), special monitoring and sedation are generally not required for most patients. However, certain aspects of the procedure and individual patient needs may influence whether additional measures are taken:

**1. Monitoring:**

- **Standard Monitoring:** Your heart rate, blood pressure, and oxygen levels may be monitored during the scan to ensure your safety and to help synchronize the MRI images with your heartbeat.
- **ECG (Electrocardiogram) Monitoring:** Electrodes are often placed on your chest to monitor your heart rhythm. This helps the MRI machine capture images at the right moments in your cardiac cycle.

**2. Sedation:**

- **Typically Not Needed:** Most patients do not require sedation for a Cardiac MRI. The procedure is non-invasive and painless, though you will need to lie still for an extended period (usually 30-60 minutes).
- **Sedation for Anxiety or Claustrophobia:** If you experience severe anxiety or claustrophobia, your doctor may prescribe a mild sedative to help you relax. However, this is not common.
- **Sedation for Children or Special Cases:** In some cases, such as for young children or individuals who cannot remain still, sedation may be used to ensure the quality of the images.

**3. Contrast Agent:**

- A contrast dye (gadolinium-based) may be injected into your bloodstream to enhance the images. This is generally well-tolerated, but inform the medical team if you have kidney problems or allergies.

**4. Breathing Instructions:**

- You may be asked to hold your breath for short periods during the scan to reduce motion artifacts and improve image quality.

**5. Communication:**

- You will be in constant communication with the MRI technologist via an intercom. They will guide you through the process and ensure you are comfortable.

If you have specific concerns, such as claustrophobia or difficulty lying still, discuss them with your healthcare provider beforehand. They can tailor the procedure to your needs.

# 43. I am undergoing heart CT. Will I need to stay for observation once the scan is done?

After a heart CT scan, whether you need to stay for observation depends on several factors:

1. **Type of CT Scan**: If you had a standard CT scan without contrast dye, you can typically leave right after the scan. If contrast dye was used, you might need to stay briefly to ensure you don’t have an allergic reaction or other side effects.
2. **Your Health Condition**: If you have underlying health issues (e.g., kidney problems, allergies, or heart conditions), your doctor may want to monitor you for a short time after the scan.
3. **Symptoms During the Scan**: If you experienced any unusual symptoms (e.g., dizziness, chest pain, or difficulty breathing) during the scan, the medical team may want to observe you before you leave.
4. **Hospital/Clinic Policy**: Some facilities have standard protocols for observation after certain procedures.

In most cases, if you feel fine and no complications arise, you can leave shortly after the scan. Always follow the instructions provided by your healthcare team. If you’re unsure, ask the staff before the procedure.

# 44. I am undergoing heart MRI. Will I need to stay for observation once the scan is done?

After a heart MRI, it's uncommon to require extended observation unless there are specific medical concerns or complications. Typically, you can leave once the scan is complete and the medical team confirms you're feeling well. However, if you received sedation, contrast dye, or have a pre-existing condition, you might need to stay briefly for monitoring. Always follow the instructions provided by your healthcare team. If you're unsure, ask them directly about their post-scan protocol.

# 45. I am undergoing Heart CT. Can I drive myself home right afterward?

After undergoing a Heart CT (computed tomography) scan, whether you can drive yourself home depends on several factors:

1. **Use of Contrast Dye**: If your CT scan involved the use of a contrast dye, you might feel slightly lightheaded or experience other mild side effects. These effects are usually temporary but can affect your ability to drive safely.
2. **Sedation**: If you were given any sedatives or anti-anxiety medications before the procedure, you should not drive yourself home. These medications can impair your reflexes and judgment.
3. **Your Overall Health**: If you have any underlying health conditions or if the procedure has left you feeling unwell, it might be safer to have someone else drive you.
4. **Medical Advice**: Always follow the specific advice given by your healthcare provider. They will consider your individual circumstances and advise you accordingly.

In general, if no sedatives were used and you feel completely normal with no side effects, you might be able to drive yourself home. However, it's often recommended to have a friend or family member accompany you to the appointment, just in case. If you have any doubts, it's best to arrange for someone else to drive you.

# 46. I am undergoing Heart MRI. Can I drive myself home right afterward?

It's generally not recommended to drive yourself home immediately after a Heart MRI (Magnetic Resonance Imaging) if you were given a sedative or if you feel dizzy, fatigued, or unwell after the procedure. While a standard MRI itself doesn't typically require sedation, some patients may receive medication to help them relax, especially if they experience anxiety or claustrophobia during the scan.

Here are some considerations:

1. **Sedation or Anxiety Medication**: If you were given a sedative or anti-anxiety medication, you should not drive for at least 24 hours, as these medications can impair your ability to operate a vehicle safely.
2. **Contrast Dye**: If contrast dye was used during the MRI, it’s rare, but some people may feel lightheaded or experience side effects. If you feel unwell, it’s safer to have someone drive you.
3. **Fatigue or Discomfort**: Even without sedation, some people feel tired or uncomfortable after lying still for an extended period during the scan. If you feel this way, it’s better to arrange for a ride home.
4. **Medical Advice**: Always follow the specific instructions given by your healthcare provider or the imaging center. They will advise you based on your individual situation.

If you’re unsure, it’s best to arrange for someone to drive you home or use public transportation or a rideshare service. Safety should always come first!

# 47. I am undergoing heart CT. Will I feel any immediate side effects like nausea or dizziness?

During a heart CT scan, also known as a coronary CT angiography (CTA), most people do not experience immediate side effects like nausea or dizziness. However, there are a few factors that could potentially cause such symptoms:

1. **Contrast Dye**: If your scan involves the use of a contrast dye (which is common for heart CT scans), some people may experience a metallic taste, a warm sensation, or mild nausea. These sensations are usually brief and subside quickly.
2. **Anxiety or Claustrophobia**: If you feel anxious or claustrophobic during the scan, it could lead to dizziness or nausea. The CT machine itself is not as confining as an MRI, but some people may still feel uncomfortable.
3. **Breath-Holding**: You may be asked to hold your breath for short periods during the scan. This can sometimes cause lightheadedness or dizziness, especially if you are not used to it.
4. **Allergic Reaction**: In rare cases, some people may have an allergic reaction to the contrast dye, which could cause nausea, dizziness, or other symptoms. This is uncommon, but if you have a known allergy to contrast dye or iodine, you should inform your healthcare provider beforehand.
5. **Low Blood Sugar**: If you have been fasting before the scan (which is sometimes required), you might feel lightheaded or nauseous due to low blood sugar.

If you do experience any side effects, they are usually mild and temporary. The medical staff will be monitoring you throughout the procedure and can assist you if you feel unwell.

Always inform your healthcare provider of any concerns or medical conditions you have before the scan, as they can take steps to minimize any potential side effects.

# 48. I am undergoing heart MRI. Will I feel any immediate side effects like nausea or dizziness?

Undergoing a heart MRI (Magnetic Resonance Imaging) is generally a safe and non-invasive procedure. Most people do not experience immediate side effects like nausea or dizziness during or after the scan. However, there are a few factors to consider:

1. **Contrast Agent**: If your MRI involves the use of a contrast dye (gadolinium-based), some people might experience mild side effects such as nausea, headache, or dizziness. These side effects are usually temporary and resolve quickly.
2. **Claustrophobia**: If you are claustrophobic or anxious about being in a confined space, you might feel dizzy or lightheaded due to stress or anxiety. Let the technician know if you feel uncomfortable, as they can provide reassurance or offer strategies to help you relax.
3. **Loud Noises**: The MRI machine produces loud knocking or buzzing sounds, which can sometimes cause discomfort or dizziness in sensitive individuals. Earplugs or headphones are usually provided to minimize this.
4. **Lying Still**: Remaining still for an extended period might cause some people to feel lightheaded, especially if they are not used to lying flat for a long time.

If you experience any unusual or severe symptoms during or after the MRI, inform the medical staff immediately. They are trained to handle any concerns and ensure your safety throughout the procedure.

# 49. I did heart CT, for which I received contrast. Do I need to drink extra fluids or take other precautions?

After a heart CT scan with contrast, it's generally recommended to drink extra fluids to help your kidneys flush out the contrast material. Here are some precautions and steps you can take:

1. **Hydration**: Drink plenty of water throughout the day. This helps your kidneys process and eliminate the contrast agent more efficiently.
2. **Monitor for Reactions**: While rare, some people may experience allergic reactions or side effects from the contrast material. Symptoms can include itching, rash, or difficulty breathing. If you notice any unusual symptoms, contact your healthcare provider immediately.
3. **Kidney Function**: If you have pre-existing kidney issues, your doctor may have given you specific instructions. Follow those carefully, as contrast agents can sometimes affect kidney function.
4. **Medications**: If you are on medications, especially those that affect kidney function (like metformin for diabetes), follow your doctor's advice on whether to pause or adjust them.
5. **Rest**: Take it easy for the rest of the day, especially if you feel any discomfort or fatigue.

Always follow any specific instructions provided by your healthcare provider, as they will have the most accurate information tailored to your health condition. If you have any concerns or experience unusual symptoms, don't hesitate to reach out to your doctor

# 50. I did heart MRI, for which I received contrast. Do I need to drink extra fluids or take other precautions?

After receiving contrast for a heart MRI, it's generally recommended to drink extra fluids to help your kidneys flush out the contrast agent from your body. Here are some precautions and steps you can take:

1. **Hydration**: Drink plenty of water in the hours and days following the procedure. This helps your kidneys process and eliminate the contrast material more efficiently.
2. **Monitor for Reactions**: While serious reactions to contrast agents are rare, be aware of any symptoms such as rash, itching, swelling, dizziness, or difficulty breathing. If you experience any of these, seek medical attention immediately.
3. **Kidney Function**: If you have a history of kidney problems, inform your healthcare provider. They may take additional precautions or monitor your kidney function more closely.
4. **Follow Medical Advice**: Always follow any specific instructions given by your healthcare provider. They may have additional recommendations based on your medical history and the type of contrast used.
5. **Regular Activities**: You can generally resume your normal activities after the MRI, but if you feel unwell or have any concerns, contact your healthcare provider.

If you have any specific health conditions or concerns, it's always best to consult with your healthcare provider for personalized advice.

# 51. I did heart CT, for which I received contrast. How will my body get rid of the contrast?

The contrast agent used in a heart CT scan (often an iodine-based contrast) is typically eliminated from your body through your kidneys. Here's how the process works:

1. **Filtration by the Kidneys**: After the CT scan, the contrast agent circulates in your bloodstream. Your kidneys filter it out of the blood, similar to how they remove waste products.
2. **Excretion in Urine**: The filtered contrast agent is then excreted from your body through urine. Most of the contrast is eliminated within 24 hours, but it can take up to 48 hours for it to be completely cleared, depending on your kidney function and hydration levels.
3. **Hydration Helps**: Drinking plenty of water after the scan can help speed up the elimination process by supporting kidney function and increasing urine output.

If you have normal kidney function, the contrast agent will be efficiently removed without any issues. However, if you have impaired kidney function, your healthcare provider may take special precautions or monitor you more closely.

Let your doctor know if you have any concerns about kidney function or if you experience unusual symptoms after the scan.

# 52. I did heart MRI, for which I received contrast. How will my body get rid of the contrast?

The contrast agent used in heart MRI (magnetic resonance imaging) is typically a gadolinium-based contrast agent (GBCA). After the MRI, your body will eliminate the contrast agent primarily through your kidneys. Here's how the process works:

1. **Filtration by the Kidneys**: The gadolinium-based contrast agent is filtered out of your bloodstream by your kidneys. This is because the contrast agent is water-soluble and not metabolized by the body.
2. **Excretion in Urine**: Once filtered by the kidneys, the contrast agent is excreted in your urine. Most of the contrast agent is eliminated within 24 hours after the MRI, but it can take up to several days for it to be completely cleared from your body, depending on your kidney function.
3. **Role of Kidney Function**: If you have normal kidney function, the contrast agent will be efficiently cleared. However, if you have impaired kidney function, the elimination process may take longer. In rare cases, individuals with severe kidney impairment may be at risk for a condition called nephrogenic systemic fibrosis (NSF), which is why kidney function is often checked before administering gadolinium-based contrast agents.
4. **Hydration**: Drinking plenty of water after the MRI can help speed up the elimination process by promoting urine production and flushing out the contrast agent more quickly.

In summary, your body will eliminate the MRI contrast agent through your kidneys, and it will be excreted in your urine. Staying hydrated can help facilitate this process. If you have concerns about your kidney function or the contrast agent, it’s always a good idea to discuss them with your healthcare provider.

# 53. I am undergoing heart CT. Will I experience any pain or soreness following the procedure?

A heart CT (computed tomography) scan is generally a painless procedure. However, you might experience some mild discomfort or soreness afterward, depending on the specifics of the test. Here are some potential sources of discomfort:

1. **IV Contrast Injection**: If your CT scan involves contrast dye (often used to enhance images), you might feel a warm sensation or a metallic taste when the dye is injected. Some people experience mild soreness or bruising at the injection site afterward.
2. **Lying Still**: You’ll need to lie still on a hard table for the duration of the scan, which might cause some stiffness or soreness, especially if you have existing back or joint issues.
3. **Radiation Exposure**: While you won’t feel anything from the radiation itself, some people feel anxious about the exposure, which can cause stress-related discomfort.
4. **Allergic Reaction (Rare)**: If you have an allergic reaction to the contrast dye, you might experience itching, rash, or mild swelling. Severe reactions are rare but possible.

**What to Expect After the Procedure:**

- You can usually resume normal activities immediately after the scan.
- If contrast dye was used, you might be advised to drink plenty of water to help flush it out of your system.
- Any soreness or bruising at the injection site should resolve within a day or two.

If you experience significant pain, swelling, or other unusual symptoms after the procedure, contact your healthcare provider promptly

# 54. I am undergoing heart MRI. Will I experience any pain or soreness following the procedure?

No, you should not experience any pain or soreness following a heart MRI (magnetic resonance imaging). The procedure is non-invasive and does not involve any incisions or physical discomfort. However, there are a few things to keep in mind:

1. **Lying Still**: You may need to lie still for an extended period (typically 30–60 minutes), which could cause some stiffness or mild discomfort, especially if you have back or joint issues.
2. **Contrast Dye (if used)**: If your MRI involves a contrast dye, you might feel a cool sensation or a metallic taste when it’s injected, but this is not painful. Rarely, some people experience mild side effects like nausea or a headache.
3. **Claustrophobia**: If you’re uncomfortable in confined spaces, the MRI machine might cause anxiety, but this is not physical pain. Let your healthcare team know if you’re feeling anxious—they can provide support or sedation if needed.
4. **Post-Procedure**: After the MRI, you can resume normal activities immediately unless you were given sedation, in which case you’ll need someone to drive you home.

If you experience any unusual symptoms after the procedure, such as swelling, rash, or significant discomfort, contact your healthcare provider. However, these are rare. Most people feel completely fine afterward.

# 55. I did heart CT. How soon can I return to my normal activities, such as work or exercise?

The time it takes to return to normal activities after a heart CT scan depends on several factors, including the type of CT scan, your overall health, and whether any sedation or contrast dye was used. Here's a general guide:

1. **If no sedation or contrast dye was used**:
   - You can typically resume normal activities, including work and light exercise, immediately after the scan.
   - A heart CT scan is non-invasive, and most people feel fine afterward.
2. **If contrast dye was used**:
   - You may be advised to drink plenty of water to help flush the dye from your system.
   - Most people can return to normal activities the same day, but if you experience mild side effects like dizziness or nausea, rest until you feel better.
3. **If sedation was used**:
   - You’ll need someone to drive you home and should avoid strenuous activities for the rest of the day.
   - You can usually return to work and light activities the next day, but follow your doctor’s specific instructions.
4. **If the scan revealed a heart condition**:
   - Your doctor may recommend restrictions on exercise or work until further evaluation or treatment is completed.

Always follow the advice of your healthcare provider, as they know your specific situation best. If you’re unsure, contact your doctor for clarification.

# 56. I did heart MRI. How soon can I return to my normal activities, such as work or exercise?

The time it takes to return to normal activities after a heart MRI (magnetic resonance imaging) depends on several factors, including the reason for the MRI, your overall health, and whether you received contrast dye or sedation during the procedure. Here's a general guide:

**1. Without Sedation or Contrast Dye:**

- If your heart MRI was performed without sedation or contrast dye, you can typically return to normal activities immediately after the scan.
- You can resume work, exercise, and other daily routines right away, as there are no restrictions.

**2. With Contrast Dye:**

- If contrast dye was used, you may be advised to drink plenty of water to help flush it out of your system.
- Most people can return to normal activities immediately, but if you experience any unusual symptoms (e.g., rash, dizziness, or nausea), contact your healthcare provider.

**3. With Sedation:**

- If you were given sedation or anti-anxiety medication, you will need someone to drive you home, as the effects can impair your ability to operate machinery or make decisions.
- You may need to rest for the remainder of the day and avoid strenuous activities until the sedation wears off completely.
- Your doctor will provide specific instructions about when you can resume work, exercise, and other activities.

**4. Underlying Health Conditions:**

- If the MRI was performed to evaluate a heart condition (e.g., cardiomyopathy, myocarditis, or heart failure), your ability to return to normal activities may depend on the findings and your doctor's recommendations.
- Follow your doctor's advice regarding exercise, work, and other activities based on your specific condition.

**5. General Recommendations:**

- If you feel well and have no restrictions from your doctor, you can usually resume normal activities right away.
- If you have any concerns or experience unusual symptoms (e.g., chest pain, shortness of breath, or fatigue), contact your healthcare provider before resuming strenuous activities.

Always follow the specific instructions provided by your doctor or the imaging center, as individual circumstances may vary. If you're unsure, it's best to consult your healthcare provider for personalized advice.

# 57. I did heart CT. Are there any dietary restrictions after the scan?

After a heart CT scan, there are generally no specific dietary restrictions unless your doctor has advised otherwise. However, here are a few general considerations:

1. **Contrast Dye (if used):** If the CT scan involved a contrast dye, you might be advised to drink plenty of water to help flush the dye out of your system. Some people may experience mild side effects like nausea, so eating light, bland foods can help if you feel unwell.
2. **Underlying Conditions:** If you have a heart condition or other health issues, follow any dietary guidelines provided by your doctor, such as limiting salt, saturated fats, or cholesterol.
3. **Medications:** If you’re on medications, ensure you take them as prescribed, and ask your doctor if any dietary adjustments are needed.

Always consult your healthcare provider for personalized advice based on your specific health situation

# 58. I did heart MRI. Are there any dietary restrictions after the scan?

No, there are typically no dietary restrictions after a heart MRI (magnetic resonance imaging). An MRI is a non-invasive imaging procedure that uses magnetic fields and radio waves to create detailed images of your heart and surrounding structures. It does not involve radiation or contrast agents that would require dietary changes in most cases.

However, if a contrast agent (gadolinium-based) was used during the MRI, your healthcare provider might give you specific instructions, such as drinking plenty of water to help flush the contrast material from your body. Unless your doctor advises otherwise, you can resume your normal diet and activities immediately after the scan.

If you have any concerns or specific conditions, it’s always best to consult your doctor for personalized advice.

# 59. I did heart CT. Do I need a follow-up visit or phone call to discuss the results?

Yes, it's generally a good idea to have a follow-up visit or phone call to discuss the results of your heart CT scan. Here’s why:

1. **Interpretation of Results**: A heart CT scan can provide detailed images of your heart and blood vessels, but the results need to be interpreted by a healthcare professional, usually a cardiologist or radiologist. They can explain what the findings mean in the context of your overall health.
2. **Next Steps**: Depending on the results, your doctor may recommend further testing, lifestyle changes, medications, or other treatments. They can also address any concerns or questions you might have.
3. **Monitoring**: If the scan revealed any abnormalities or conditions that need monitoring, your doctor will want to discuss a plan for ongoing care.
4. **Peace of Mind**: Even if the results are normal, discussing them with your doctor can provide reassurance and clarify any next steps for maintaining heart health.

If your doctor hasn’t already scheduled a follow-up, it’s a good idea to contact their office to ask if one is needed. They’ll let you know whether an in-person visit or a phone call is appropriate.

# 60. I did heart CT. What symptoms after the test should prompt me to call my doctor?

After a heart CT scan (also known as a coronary CT angiography or calcium score scan), most people experience no significant side effects. However, there are certain symptoms that could indicate a complication or require medical attention. You should contact your doctor if you experience any of the following after the test:

**1. Allergic Reaction (if contrast dye was used):**

- **Symptoms:** Rash, itching, swelling (especially of the face, lips, or tongue), difficulty breathing, or dizziness.
- **Action:** Seek immediate medical attention if you experience these symptoms, as they could indicate an allergic reaction to the contrast dye.

**2. Kidney Issues (if contrast dye was used):**

- **Symptoms:** Decreased urination, swelling in the legs or feet, fatigue, or shortness of breath.
- **Action:** Contact your doctor, especially if you have a history of kidney problems, as contrast dye can affect kidney function.

**3. Pain or Discomfort:**

- **Symptoms:** Severe pain at the injection site (where the IV was placed), chest pain, or unusual discomfort in the arm or leg where the contrast was injected.
- **Action:** Call your doctor, as this could indicate a rare complication like contrast extravasation (leakage of contrast into surrounding tissue) or other issues.

**4. Infection:**

- **Symptoms:** Redness, warmth, swelling, or pus at the IV site, fever, or chills.
- **Action:** Contact your doctor, as these could be signs of an infection.

**5. Unusual Fatigue or Weakness:**

- **Symptoms:** Extreme tiredness, dizziness, or feeling faint.
- **Action:** Inform your doctor, especially if these symptoms persist or worsen.

**6. Shortness of Breath or Irregular Heartbeat:**

- **Symptoms:** Difficulty breathing, palpitations, or a rapid or irregular heartbeat.
- **Action:** Seek medical attention, as these could be related to heart issues.

**7. Persistent Nausea or Vomiting:**

- **Symptoms:** Ongoing nausea or vomiting after the procedure.
- **Action:** Contact your doctor, especially if you received contrast dye.

**8. Headache or Confusion:**

- **Symptoms:** Severe headache, confusion, or difficulty thinking clearly.
- **Action:** Seek medical attention, as these could be signs of a rare reaction or other underlying issues.

**When to Seek Immediate Emergency Care:**

If you experience **chest pain, severe shortness of breath, or signs of a severe allergic reaction (e.g., swelling of the face or throat, difficulty breathing)**, call emergency services or go to the nearest emergency room immediately.

**General Advice:**

- If you have any concerns or symptoms that feel unusual after the test, it’s always better to err on the side of caution and contact your healthcare provider.
- Make sure to follow any post-procedure instructions given by your doctor or the imaging center.

Let your doctor know about any pre-existing conditions (e.g., kidney disease, allergies) or medications you’re taking, as these can influence your risk of complications.

# 61. I did heart MRI. What symptoms after the test should prompt me to call my doctor?

After a heart MRI (cardiac MRI), most people do not experience significant side effects, as it is a non-invasive procedure. However, there are certain symptoms that could indicate a complication or require medical attention. Contact your doctor if you experience any of the following after the test:

1. **Allergic Reaction**: If you received a contrast dye during the MRI, watch for signs of an allergic reaction, such as:
   - Hives or rash
   - Itching
   - Swelling of the face, lips, tongue, or throat
   - Difficulty breathing or wheezing
2. **Pain or Discomfort**: While mild discomfort at the injection site (if contrast was used) is normal, severe pain, swelling, or redness could indicate an issue.
3. **Kidney Problems**: If you have a history of kidney issues and received contrast dye, watch for symptoms of kidney dysfunction, such as:
   - Decreased urination
   - Swelling in the legs or feet
   - Fatigue or confusion
4. **Dizziness or Lightheadedness**: If you feel unusually dizzy, faint, or lightheaded, it could be related to the procedure or an underlying condition.
5. **Chest Pain or Palpitations**: If you experience new or worsening chest pain, pressure, or irregular heartbeats, seek medical attention immediately, as this could indicate a heart-related issue.
6. **Fever or Infection**: If you develop a fever, chills, or signs of infection (e.g., redness, warmth, or pus at the injection site), contact your doctor.
7. **Unusual Symptoms**: Any other unusual or severe symptoms that concern you should be reported to your doctor.

If you experience severe symptoms like difficulty breathing, chest pain, or a severe allergic reaction, seek emergency medical care immediately.

Always follow any specific post-MRI instructions provided by your healthcare provider.

# 62. Can I travel (or fly) shortly after having a cardiac CT?

Yes, you can generally travel or fly shortly after having a cardiac CT scan, as it is a non-invasive procedure with no significant recovery time. However, there are a few considerations to keep in mind:

1. **Contrast Dye**: If your cardiac CT involved the use of a contrast dye, ensure you have no adverse reactions (e.g., allergic reaction or kidney issues). Most reactions, if any, occur shortly after the injection, so waiting a few hours post-scan is usually sufficient.
2. **Medical Condition**: If the CT scan was performed to evaluate a heart condition, your ability to travel may depend on the findings and your overall health. Consult your doctor to ensure it’s safe, especially if you have symptoms like chest pain, shortness of breath, or dizziness.
3. **Airline Restrictions**: Most airlines do not have restrictions related to medical imaging, but if you have a serious heart condition, check with the airline and your doctor before flying.
4. **Hydration**: If contrast dye was used, staying hydrated can help flush it out of your system, which may be especially important before a long flight.

Always consult your healthcare provider for personalized advice based on your specific situation.

# 63. Can I travel (or fly) shortly after having a cardiac MRI?

Yes, you can generally travel or fly shortly after having a cardiac MRI, as the procedure itself does not impose restrictions on travel. However, there are a few considerations to keep in mind:

1. **Your Health Condition**: The reason for the cardiac MRI is important. If you have a serious heart condition or are experiencing symptoms (e.g., chest pain, shortness of breath), you should consult your doctor before traveling. They may advise against flying or recommend specific precautions.
2. **Contrast Agent**: If your MRI involved a contrast agent (e.g., gadolinium), there are typically no restrictions on flying afterward. However, if you experienced side effects (e.g., allergic reactions or kidney issues), discuss travel plans with your doctor.
3. **Post-Procedure Symptoms**: If you feel unwell, dizzy, or fatigued after the MRI, it may be wise to rest before traveling.
4. **Airline Policies**: Most airlines do not have restrictions related to MRIs, but if you have a medical condition or require special assistance, inform the airline in advance.
5. **Doctor’s Advice**: Always follow your doctor’s recommendations based on your specific health situation.

If you feel well and have no complications, flying shortly after a cardiac MRI is usually safe. However, if you have any concerns, consult your healthcare provider.

# 64. Is there a radiation risk with cardiac CT, and how significant is it?

Yes, there is a radiation risk associated with cardiac CT scans, as they use ionizing radiation to create detailed images of the heart and surrounding structures. The significance of the radiation risk depends on several factors, including the type of CT scan, the dose of radiation used, the patient's age, and the frequency of exposure.

**Key Points:**

1. **Radiation Dose**:
   - The effective dose of radiation from a cardiac CT scan varies depending on the protocol and equipment used. On average, a cardiac CT scan can deliver a dose ranging from **1 to 10 millisieverts (mSv)**.
   - For comparison, the average annual background radiation exposure from natural sources is about **3 mSv**.
2. **Cancer Risk**:
   - The risk of developing cancer from radiation exposure is generally small but increases with higher doses and repeated exposures.
   - The lifetime risk of cancer from a single cardiac CT scan is estimated to be very low (e.g., less than 0.1% for most adults). However, this risk may be slightly higher for younger patients or those undergoing multiple scans.
3. **Factors Influencing Risk**:
   - **Age**: Younger patients are more sensitive to radiation because their cells are dividing more rapidly and they have a longer lifespan for potential effects to manifest.
   - **Gender**: Women may have a slightly higher risk of radiation-induced cancer from cardiac CT due to increased sensitivity of breast tissue.
   - **Scan Protocol**: Modern CT scanners and optimized protocols (e.g., low-dose techniques) can significantly reduce radiation exposure without compromising image quality.
4. **Benefits vs. Risks**:
   - The benefits of a cardiac CT scan, such as diagnosing coronary artery disease, evaluating heart function, or planning interventions, often outweigh the small radiation risk, especially in patients with significant symptoms or risk factors.
5. **Minimizing Risk**:
   - Use of **low-dose protocols** and advanced imaging techniques (e.g., iterative reconstruction).
   - Limiting scans to clinically necessary cases and avoiding repeat imaging when possible.
   - Shielding sensitive areas (e.g., breasts in women) when feasible.

**Conclusion:**

While there is a small radiation risk associated with cardiac CT scans, the risk is generally low and should be weighed against the potential diagnostic benefits. Advances in technology and imaging protocols continue to reduce radiation exposure, making cardiac CT a safer and more effective tool for cardiovascular evaluation. Always discuss the risks and benefits with your healthcare provider to make an informed decision.

# 65. Is there a radiation risk with cardiac MRI, and how significant is it?

Cardiac MRI (Magnetic Resonance Imaging) is generally considered a safe imaging technique because it does not use ionizing radiation, unlike X-rays or CT scans. Instead, it relies on strong magnetic fields and radio waves to create detailed images of the heart and surrounding structures. Therefore, there is **no radiation risk** associated with cardiac MRI.

**Key Points:**

1. **No Ionizing Radiation**: Cardiac MRI does not expose patients to ionizing radiation, making it a preferred option for repeated imaging, especially in vulnerable populations like children or pregnant women.
2. **Magnetic Fields and Radio Waves**: The procedure uses a combination of magnetic fields and radiofrequency pulses, which are not known to cause harmful effects when used within standard clinical guidelines.
3. **Contrast Agents**: In some cases, a gadolinium-based contrast agent may be used to enhance images. While generally safe, there are rare risks associated with these agents, such as allergic reactions or nephrogenic systemic fibrosis (NSF) in patients with severe kidney dysfunction. However, these risks are unrelated to radiation.

**When to Be Cautious:**

- Patients with certain implants (e.g., pacemakers, defibrillators) or metallic objects in their bodies should inform their healthcare provider, as these can interfere with the MRI's magnetic field.
- Pregnant women are typically advised to avoid MRI during the first trimester unless absolutely necessary, though this is a precaution rather than a radiation-related concern.

**Conclusion:**

Cardiac MRI is a safe and effective imaging tool with **no radiation risk**. Its ability to provide detailed, high-resolution images of the heart without exposing patients to ionizing radiation makes it a valuable diagnostic tool in cardiology. Always discuss any concerns with your healthcare provider to ensure the procedure is appropriate for your specific situation.

# 66. I have nails in my arm, can I do heart MRI?

If you have metal objects, such as nails, in your body, it is crucial to inform your healthcare provider and the MRI technologist before undergoing an MRI scan. Metal can interfere with the magnetic field of the MRI machine, potentially causing serious safety issues, such as movement of the metal objects, heating, or distortion of the images.

Here are some key points to consider:

1. **Type of Metal**: Not all metals are magnetic. If the nails are made of non-ferromagnetic materials (e.g., titanium, certain stainless steels), they may be safe for an MRI. However, ferromagnetic metals (e.g., iron, nickel, cobalt) can be dangerous.
2. **Location of the Nails**: If the nails are in your arm and the MRI is focused on your heart, the risk might be lower, but it still needs to be evaluated by a medical professional.
3. **Medical History**: Provide your complete medical history, including any surgeries, implants, or foreign objects in your body, to your healthcare provider.
4. **Alternative Imaging**: If an MRI is deemed unsafe, your doctor may recommend alternative imaging methods, such as a CT scan or ultrasound.

Always consult with your healthcare provider and the MRI team to assess the risks and determine the best course of action for your specific situation.

# 67. I have an inserted heart device. Can I do heart MRI?

**If you have an implanted heart device, such as a pacemaker or an implantable cardioverter-defibrillator (ICD), undergoing an MRI (Magnetic Resonance Imaging) scan can be complex and potentially risky. Traditional MRI machines use strong magnetic fields and radio waves, which can interfere with the function of these devices, potentially causing harm.**

**However, advancements in technology have led to the development of MRI-conditional devices, which are designed to be safe under specific MRI conditions. If your device is MRI-conditional, you may be able to undergo an MRI scan, but only under strict guidelines and with proper precautions.**

**Here’s what you should do:**

1. **Check Your Device Type: Confirm with your cardiologist whether your device is MRI-conditional. If it is, they will provide specific instructions and ensure the MRI is performed under the appropriate conditions.**
2. **Consult Your Cardiologist: Even if your device is MRI-conditional, your cardiologist will need to evaluate your individual case and determine if an MRI is safe for you. They may need to adjust your device settings before and after the scan.**
3. **Inform the MRI Technicians: Always inform the MRI facility about your implanted device. They will need to follow specific protocols to ensure your safety.**
4. **Non-MRI-Conditional Devices: If your device is not MRI-conditional, an MRI is generally not recommended. Alternative imaging methods, such as CT scans or ultrasound, may be considered instead.**

**In summary, whether you can undergo an MRI depends on the type of device you have. Always consult your cardiologist and the MRI facility to ensure your safety.**

# 68. I am pregnant, can I undergo heart CT?

Congratulations on your pregnancy! When it comes to medical imaging, especially during pregnancy, it's important to weigh the benefits and risks carefully.

A **CT scan of the heart** involves exposure to **ionizing radiation**, which can pose risks to the developing fetus, particularly in the first trimester when organs are forming. However, the risk depends on the radiation dose, the area being scanned, and the stage of pregnancy.

**Key Considerations:**

1. **Radiation Exposure**:
   - The fetus is most sensitive to radiation during the first trimester.
   - A CT scan of the heart typically focuses on the chest, so the abdomen (and fetus) may receive some scattered radiation, but the dose is usually low.
2. **Medical Necessity**:
   - If the CT scan is critical for diagnosing or managing a serious heart condition that could affect your health or the pregnancy, the benefits may outweigh the risks.
   - Your doctor may explore alternative imaging methods, such as **echocardiography** (ultrasound of the heart) or **MRI** (which does not use ionizing radiation), if they can provide the necessary information.
3. **Shielding**:
   - If a CT scan is deemed necessary, the radiology team can use lead shielding to minimize radiation exposure to the abdomen and pelvis.

**What Should You Do?**

- **Consult your healthcare provider**: Discuss the necessity of the CT scan and whether there are safer alternatives.
- **Involve a radiologist**: They can help determine the safest approach and calculate the potential radiation dose to the fetus.
- **Weigh risks and benefits**: Ensure that the scan is absolutely necessary for your health and that no safer alternative is available.

In summary, while a heart CT scan is generally avoided during pregnancy unless absolutely necessary, your medical team will guide you based on your specific situation. Always prioritize open communication with your healthcare providers.

# 69. I am pregnant, can I undergo heart MRI?

Congratulations on your pregnancy! When it comes to undergoing a heart MRI (magnetic resonance imaging) during pregnancy, there are several factors to consider:

1. **Safety of MRI in Pregnancy**: MRI is generally considered safe during pregnancy, especially after the first trimester. Unlike CT scans or X-rays, MRI does not use ionizing radiation, which can be harmful to the developing fetus. Instead, MRI uses magnetic fields and radio waves to create detailed images of the body.
2. **Contrast Agents**: If the MRI requires the use of a contrast agent (such as gadolinium), this should be carefully considered. Gadolinium-based contrast agents can cross the placenta and enter the fetal bloodstream, and their safety during pregnancy is not fully established. The use of contrast agents is generally avoided unless absolutely necessary.
3. **Medical Necessity**: The decision to undergo an MRI during pregnancy should be based on the medical necessity of the procedure. If the information obtained from the MRI is critical for your health or the management of your pregnancy, your healthcare provider may recommend proceeding with the MRI.
4. **First Trimester Caution**: While MRI is generally considered safe after the first trimester, there is limited data on its effects during the first trimester, which is a critical period of fetal development. If possible, non-urgent MRIs are usually postponed until after the first trimester.
5. **Consult Your Healthcare Provider**: It is essential to discuss the risks and benefits of undergoing an MRI with your healthcare provider, including your obstetrician and the radiologist. They will consider your specific medical situation and determine whether the MRI is necessary and safe for you and your baby.

In summary, while MRI is generally considered safe during pregnancy, especially after the first trimester, the decision should be made in consultation with your healthcare provider, taking into account the necessity of the procedure and the potential risks and benefits.

# 70. I have kidney problems, can I do heart CT?

If you have kidney problems, it's important to be cautious about undergoing a heart CT scan, particularly if the scan involves the use of contrast dye (contrast material). The contrast dye used in some CT scans can potentially worsen kidney function, especially in individuals who already have kidney issues. This condition is known as contrast-induced nephropathy (CIN).

Here are some key points to consider:

1. **Consult Your Doctor**: Always discuss your kidney condition with your healthcare provider before undergoing any imaging tests that involve contrast dye. They can assess your kidney function and determine if the benefits of the CT scan outweigh the risks.
2. **Kidney Function Tests**: Your doctor may order blood tests to evaluate your kidney function (e.g., serum creatinine and estimated glomerular filtration rate (eGFR)) before proceeding with the scan.
3. **Alternative Imaging**: If the risk of using contrast dye is too high, your doctor might recommend alternative imaging methods that do not require contrast, such as a non-contrast CT, MRI, or echocardiogram.
4. **Hydration**: If contrast dye is deemed necessary, your doctor may recommend hydration before and after the procedure to help protect your kidneys.
5. **Medication Adjustments**: In some cases, medications that could affect kidney function (e.g., certain diabetes medications like metformin) may need to be temporarily stopped before the procedure.

Always follow your healthcare provider's advice and ensure they are fully aware of your kidney condition and any other health issues you may have.

# 71. I have kidney problems, can I do heart MRI?

If you have kidney problems, it's important to discuss this with your healthcare provider before undergoing a heart MRI, especially if the MRI involves the use of a contrast agent. Here’s why:

1. **Contrast Agents and Kidney Function**: Some MRI scans use a contrast agent (usually gadolinium-based) to enhance the images. If your kidney function is impaired, your body may have difficulty clearing the contrast agent, which could lead to a rare but serious condition called **nephrogenic systemic fibrosis (NSF)**. This condition is associated with the use of gadolinium-based contrast agents in people with severe kidney impairment.
2. **Assessment of Kidney Function**: Before an MRI with contrast, your healthcare provider may check your kidney function through blood tests (e.g., serum creatinine and estimated glomerular filtration rate, or eGFR). If your kidney function is significantly reduced, they may avoid using contrast or take special precautions.
3. **Alternative Imaging**: If contrast is deemed too risky, your doctor might consider alternative imaging methods, such as a non-contrast MRI, echocardiography, or other tests that do not require contrast agents.
4. **Hydration and Precautions**: In some cases, if contrast is necessary, your doctor may recommend hydration before and after the procedure or use a lower-risk gadolinium agent to minimize potential complications.

Always inform your healthcare team about your kidney condition and any other health issues before undergoing any imaging procedure. They will weigh the risks and benefits and determine the safest approach for you.

# 72. If I’m claustrophobic, can I do heart MRI?

If you're claustrophobic, undergoing a heart MRI can be challenging due to the confined space of the MRI machine. However, there are strategies to help you manage the experience:

1. **Open MRI Machines**: Some facilities offer open MRI machines, which are less enclosed and may be more comfortable for claustrophobic patients. However, these may not always provide the same image quality as traditional MRI machines, especially for heart imaging.
2. **Sedation**: Your doctor may prescribe a mild sedative to help you relax during the procedure. This is a common option for patients with claustrophobia.
3. **Communication**: Inform the MRI technologist about your claustrophobia. They can provide reassurance, explain the process, and may allow you to take breaks if needed.
4. **Distraction Techniques**: Some facilities offer headphones with music or guided imagery to help distract you during the scan.
5. **Practice or Visualization**: Some patients find it helpful to practice relaxation techniques or visualize the process beforehand to reduce anxiety.
6. **Alternative Imaging**: In some cases, your doctor may consider alternative imaging methods, such as a CT scan, though these may not provide the same level of detail as an MRI for certain heart conditions.

Always discuss your concerns with your healthcare provider. They can help determine the best approach for your specific situation and ensure your comfort and safety during the procedure.

# 73. My doctor is suggesting heart CT, could a cardiac ultrasound (echo) or stress test replace the CT?

When your doctor suggests a heart CT (computed tomography) scan, they are likely looking for detailed images of your heart's structure, including the coronary arteries, to assess for blockages, plaque buildup, or other abnormalities. Whether a cardiac ultrasound (echocardiogram) or a stress test can replace a heart CT depends on the specific clinical question your doctor is trying to answer.

**1. Cardiac Ultrasound (Echocardiogram)**

- **What it does**: An echocardiogram uses sound waves to create images of the heart's structure and function. It provides information about the heart's chambers, valves, and pumping ability.
- **When it might replace a CT**: If the primary concern is evaluating heart function, valve function, or the presence of fluid around the heart, an echocardiogram might be sufficient. However, it does not provide detailed images of the coronary arteries, so it cannot replace a CT if the goal is to assess for coronary artery disease (CAD).

**2. Stress Test**

- **What it does**: A stress test evaluates how well your heart performs under physical stress (usually exercise or medication-induced). It can detect reduced blood flow to the heart muscle, which may indicate blockages in the coronary arteries.
- **When it might replace a CT**: A stress test is useful for assessing symptoms like chest pain or shortness of breath, especially if coronary artery disease is suspected. However, it is less specific than a CT for identifying the exact location and severity of blockages. If the CT is intended to provide detailed anatomical information, a stress test alone may not be sufficient.

**3. Heart CT (Coronary CT Angiography)**

- **What it does**: A heart CT provides detailed images of the coronary arteries, allowing doctors to see blockages, plaque buildup, and other structural issues.
- **When it is necessary**: If your doctor is specifically looking for detailed information about the coronary arteries, a heart CT is often the best option. It is particularly useful for evaluating chest pain in patients with an intermediate risk of coronary artery disease.

**Key Considerations:**

- **Symptoms and Risk Factors**: If you have symptoms like chest pain, shortness of breath, or a high risk of coronary artery disease, a heart CT may be the most appropriate test.
- **Previous Test Results**: If you’ve already had an echocardiogram or stress test that was inconclusive, a heart CT might be the next step.
- **Radiation Exposure**: A heart CT involves radiation exposure, which is not the case with an echocardiogram. If radiation exposure is a concern, discuss this with your doctor.

**Conclusion:**

While an echocardiogram or stress test can provide valuable information, they may not fully replace a heart CT if detailed images of the coronary arteries are needed. It’s important to discuss your specific situation with your doctor to determine the most appropriate test for your condition.

# 74. What is I can't undergo heart MRI, are there any alternatives?

If you cannot undergo a heart MRI (magnetic resonance imaging), there are several alternative imaging techniques that your doctor might consider, depending on your specific condition and the information needed. Here are some common alternatives:

1. **Echocardiogram (Echo)**:
   - **Transthoracic Echocardiogram (TTE)**: This is a non-invasive ultrasound of the heart that provides detailed images of the heart's structure and function.
   - **Transesophageal Echocardiogram (TEE)**: This involves passing a specialized probe down the esophagus to get closer and more detailed images of the heart.
2. **CT Scan (Computed Tomography)**:
   - **Cardiac CT**: This can provide detailed images of the heart and coronary arteries. It is particularly useful for assessing coronary artery disease and congenital heart disease.
   - **CT Angiography (CTA)**: This involves injecting a contrast dye to visualize the blood vessels and assess blockages.
3. **Nuclear Imaging**:
   - **Myocardial Perfusion Imaging (MPI)**: This test uses a small amount of radioactive material to assess blood flow to the heart muscle.
   - **PET Scan (Positron Emission Tomography)**: This can provide detailed information about blood flow and metabolic activity in the heart.
4. **X-ray**:
   - **Chest X-ray**: While not as detailed as other imaging techniques, it can provide information about the size and shape of the heart and the condition of the lungs.
5. **Electrocardiogram (ECG or EKG)**:
   - This test measures the electrical activity of the heart and can help diagnose various heart conditions, although it does not provide images.
6. **Cardiac Catheterization**:
   - This invasive procedure involves inserting a catheter into a blood vessel and guiding it to the heart to measure pressures and inject contrast dye for imaging (angiography).
7. **Stress Test**:
   - This can be done with or without imaging (like echocardiography or nuclear imaging) to assess how the heart performs under stress, such as exercise or medication-induced stress.

Each of these alternatives has its own advantages and limitations, and the choice of test will depend on the specific clinical question your doctor is trying to answer. It's important to discuss your options with your healthcare provider to determine the most appropriate test for your situation.

# 75. I am supposed to do Heart CT. However, I have allergy to contrast dye—are there alternative options?

If you have an allergy to contrast dye and need a Heart CT (commonly a Coronary CT Angiography), there are several alternative options and strategies that can be considered:

**1. Pre-Medication for Contrast Allergy:**

- If your allergy to contrast dye is mild to moderate, your doctor may recommend pre-medication with antihistamines and corticosteroids to reduce the risk of an allergic reaction. This allows you to proceed with the contrast-enhanced CT scan safely.

**2. Non-Contrast CT Scan:**

- A non-contrast CT scan can still provide valuable information about the heart, such as detecting calcium deposits in the coronary arteries (calcium score). However, it may not provide as detailed information about blood flow or blockages as a contrast-enhanced scan.

**3. Alternative Imaging Modalities:**

- **Cardiac MRI:** Magnetic Resonance Imaging (MRI) can assess heart function, structure, and blood flow without the need for iodine-based contrast. In some cases, a gadolinium-based contrast agent may be used, which is different from the iodine-based contrast used in CT scans and may be better tolerated.
- **Stress Echocardiography:** This ultrasound-based test evaluates heart function and blood flow during physical or pharmacological stress. It does not require contrast dye.
- **Nuclear Stress Test:** This test uses a small amount of radioactive tracer to assess blood flow to the heart. It is an alternative for evaluating coronary artery disease.
- **Invasive Coronary Angiography:** If non-invasive tests are inconclusive, a traditional coronary angiogram (catheterization) may be performed. While this also uses contrast dye, the procedure is done in a controlled environment where any allergic reaction can be managed immediately.

**4. Use of Alternative Contrast Agents:**

- In some cases, non-iodinated contrast agents or lower-osmolality contrast agents may be used, which are less likely to cause allergic reactions.

**5. Discuss with Your Doctor:**

- It’s important to discuss your allergy history and concerns with your cardiologist or radiologist. They can tailor the imaging approach based on your specific condition, the severity of your allergy, and the diagnostic information needed.

**6. Allergy Testing:**

- If your allergy to contrast dye is not well-defined, you may consider allergy testing to confirm the specific type of contrast agent you are allergic to. This can help guide the choice of alternative contrast agents.

Always inform your healthcare provider about your allergy before any imaging procedure to ensure your safety and the best diagnostic approach.

# 76. Since there's no radiation risk in heart MRI, why is my doctor recommending heart CT?

Your doctor may recommend a heart CT scan over an MRI for several reasons, even though MRI does not involve ionizing radiation. Here are some possible reasons:

1. **Specific Clinical Question**: CT scans are particularly good at visualizing coronary arteries and detecting calcifications or blockages. If your doctor is specifically concerned about coronary artery disease, a CT angiogram might be more appropriate.
2. **Speed and Availability**: CT scans are generally faster than MRI scans. If time is a critical factor, a CT scan might be preferred. Additionally, CT scanners are more widely available than MRI machines.
3. **Patient Factors**: Some patients may have contraindications for MRI, such as certain types of metal implants, pacemakers, or severe claustrophobia. In such cases, a CT scan might be a safer or more feasible option.
4. **Image Quality**: In some cases, CT might provide better image quality for the specific area of interest, especially when looking at fine details in the coronary arteries.
5. **Cost**: CT scans are often less expensive than MRI scans, which might be a consideration depending on your healthcare system and insurance coverage.
6. **Follow-Up**: If you have had previous CT scans, your doctor might recommend another CT for consistency in comparing images over time.

It's important to discuss with your doctor why they are recommending a CT scan in your particular case. They can provide you with the specific reasons based on your medical history, current condition, and the diagnostic information they need.

# 77. I did heart CT, When and how will I receive my results?

The timing and method for receiving your heart CT (computed tomography) results can vary depending on the healthcare facility and the urgency of your situation. Here's a general guideline:

**1. Timing:**

- **Routine Cases:** Results are typically available within a few days to a week.
- **Urgent Cases:** If your scan was done in an emergency or urgent care setting, results may be available within hours or sooner, depending on the situation.

**2. How You’ll Receive Results:**

- **Follow-Up Appointment:** Your doctor may schedule a follow-up appointment to discuss the results with you.
- **Online Portal:** Many healthcare providers use online patient portals where you can access your results as soon as they’re available.
- **Phone Call:** In some cases, your doctor or a nurse may call you to discuss the results, especially if further action is needed.
- **Mail:** Some facilities may send results via mail, though this is less common now.

**3. Next Steps:**

- If the results indicate any concerns, your doctor will discuss the next steps, which may include further testing, treatment, or lifestyle changes.
- If the results are normal, your doctor may still want to discuss any ongoing symptoms or concerns.

**4. What to Do While Waiting:**

- If you haven’t heard back within the expected timeframe, don’t hesitate to contact your doctor’s office or the imaging center for an update.
- Avoid self-diagnosing or worrying excessively while waiting, as this can lead to unnecessary stress.

Always follow up with your healthcare provider to ensure you fully understand your results and any recommended next steps.

# 78. I did heart MRI, when and how will I receive my results?

The timing and process for receiving your heart MRI results can vary depending on the healthcare facility and your location. Here's a general guide:

1. **Timing**:
   - Results are typically available within a few days to a week after the MRI.
   - In urgent cases, results may be available sooner, sometimes even the same day.
2. **How You’ll Receive Results**:
   - **Follow-Up Appointment**: Your doctor may schedule a follow-up appointment to discuss the results with you.
   - **Online Portal**: Many healthcare providers use patient portals where you can access your results directly.
   - **Phone Call**: Your doctor or a member of their team may call you to explain the results.
   - **Mail**: In some cases, results may be mailed to you, though this is less common.
3. **Next Steps**:
   - If the results are normal, your doctor may simply inform you and recommend any necessary follow-up care.
   - If abnormalities are found, your doctor will discuss further testing, treatment options, or referrals to specialists.

**What You Can Do**:

- Contact the facility where you had the MRI or your referring doctor’s office to ask about the expected timeline.
- If you have access to a patient portal, check there for updates.

Always follow up if you haven’t received your results within the expected timeframe.

# 79. Will I need additional testing after heart CT?

Whether you'll need additional testing after a heart CT scan depends on the results of the scan and your overall health condition. Here are some scenarios where further testing might be recommended:

1. **Abnormal Findings**: If the CT scan reveals abnormalities such as blockages, plaque buildup, or other issues, your doctor may recommend additional tests like a stress test, echocardiogram, or cardiac catheterization to gather more information.
2. **Symptoms Persist**: If you continue to experience symptoms like chest pain, shortness of breath, or dizziness, further testing may be needed to determine the cause.
3. **Risk Factors**: If you have risk factors for heart disease (e.g., high cholesterol, diabetes, smoking, or a family history of heart disease), your doctor might suggest additional tests to monitor your heart health.
4. **Inconclusive Results**: Sometimes, a heart CT scan may not provide clear answers, and further imaging or diagnostic tests may be required.
5. **Pre-Surgical Evaluation**: If you're being evaluated for surgery or a procedure, additional tests might be needed to ensure your heart is healthy enough for the intervention.

Your doctor will review the CT scan results, consider your symptoms and medical history, and decide if further testing is necessary. Always follow your healthcare provider's recommendations for the best course of action.

# 80. I am undergoing heart MRI. Can I obtain copies of the images or medical reports for my personal records?

Yes, you can typically obtain copies of your heart MRI images and medical reports for your personal records. Here’s how you can usually go about it:

1. **Request from the Hospital or Imaging Center**: Contact the hospital or imaging center where the MRI was performed. They often have a medical records department that handles such requests.
2. **Authorization Form**: You may need to fill out an authorization form to release your medical records. This is to ensure your privacy and compliance with healthcare regulations like HIPAA in the United States.
3. **Format**: Specify whether you want digital copies (e.g., on a CD or DVD) or printed images and reports. Digital copies are often more convenient and can be easily shared with other healthcare providers.
4. **Fees**: Be aware that there may be a fee for copying and transferring your records. This varies by institution.
5. **Timing**: Ask about the turnaround time. Some places can provide the records immediately, while others may take a few days.
6. **Patient Portals**: Many healthcare providers offer online patient portals where you can access your medical records, including imaging results, directly.
7. **Consult Your Doctor**: It’s also a good idea to discuss this with your doctor. They can help you understand the images and reports and may facilitate the process of obtaining them.

Remember, having your medical records can be very useful for second opinions, ongoing care, or personal reference.

# 81. If my heart CT turns to be normal, does that guarantee I’m free from heart disease?

A normal heart CT scan is a positive sign, but it doesn't guarantee that you are entirely free from heart disease. Here's why:

1. **Scope of the CT Scan**: A heart CT scan, such as a coronary CT angiography, primarily evaluates the coronary arteries for blockages or plaque buildup. If the scan is normal, it suggests no significant coronary artery disease at the time of the scan. However, it doesn't assess other aspects of heart health, such as heart rhythm disorders, heart valve issues, or heart muscle abnormalities.
2. **Early-Stage Disease**: Some heart conditions, like very early-stage atherosclerosis or small areas of inflammation, might not be detectable on a CT scan.
3. **Functional Issues**: A CT scan is an anatomical test and doesn't evaluate how well your heart functions under stress or during physical activity. Conditions like ischemia (reduced blood flow to the heart muscle) might only appear during exertion and could be missed on a resting CT scan.
4. **Other Heart Conditions**: Heart diseases such as arrhythmias, heart failure, or congenital heart defects may not be identified by a CT scan alone.
5. **Risk Factors**: Even with a normal CT scan, if you have risk factors like high blood pressure, high cholesterol, diabetes, smoking, or a family history of heart disease, you could still be at risk for developing heart disease in the future.

To fully assess your heart health, your doctor may recommend additional tests, such as an electrocardiogram (ECG), stress test, echocardiogram, or blood tests, depending on your symptoms and risk factors. Regular check-ups and managing risk factors are key to preventing heart disease. Always consult your doctor for personalized advice.

# 82. If my heart MRI turns to be normal, does that guarantee I’m free from heart disease?

A normal heart MRI is a good sign, but it doesn't guarantee you're completely free from heart disease. Here's why:

1. **Limitations of MRI**: While MRI is excellent for assessing heart structure, function, and tissue characteristics, it may not detect all types of heart disease. For example, early-stage coronary artery disease (plaque buildup in arteries) might not be visible unless specialized techniques like stress perfusion imaging are used.
2. **Other Types of Heart Disease**: Some conditions, like certain arrhythmias (irregular heart rhythms) or microvascular disease (issues with small blood vessels), may not be easily detected by a standard MRI.
3. **Risk Factors and Symptoms**: Even with a normal MRI, if you have risk factors (e.g., high blood pressure, high cholesterol, diabetes, smoking, or a family history of heart disease) or symptoms (e.g., chest pain, shortness of breath, or fatigue), further evaluation might be needed.
4. **Dynamic Nature of Heart Disease**: Heart disease can develop over time. A normal MRI today doesn't rule out the possibility of future issues, especially if risk factors are present.

If you have concerns about heart disease, it's important to discuss your symptoms, risk factors, and test results with your doctor. They may recommend additional tests or monitoring based on your individual situation.

# 83. My doctor says that my heart CT is positive, how certain is that?

A "positive" result on a heart CT scan typically means that the scan has detected some form of abnormality, such as plaque buildup in the coronary arteries, which could indicate coronary artery disease (CAD). However, the term "positive" can vary depending on the context and what the scan was specifically looking for.

**Key Points to Consider:**

1. **Type of CT Scan**:
   - If you had a **Coronary CT Angiogram (CTA)**, a positive result usually means there is evidence of narrowing or blockages in the coronary arteries.
   - If you had a **Calcium Score Scan**, a positive result indicates the presence of calcium deposits in the coronary arteries, which is a sign of atherosclerosis (plaque buildup).
2. **Degree of Certainty**:
   - A heart CT scan is generally very accurate in detecting coronary artery disease, but it is not 100% definitive. False positives or false negatives can occur, though they are relatively rare with modern imaging technology.
   - The certainty of the result also depends on the quality of the scan and the expertise of the radiologist interpreting it.
3. **Next Steps**:
   - Your doctor will likely recommend further tests or treatments based on the CT results. This could include stress tests, more advanced imaging, or even invasive procedures like a coronary angiogram.
   - Lifestyle changes, medications, or interventions (like stents or bypass surgery) may be discussed depending on the severity of the findings.
4. **Clinical Correlation**:
   - The CT result is just one piece of the puzzle. Your doctor will consider your symptoms, risk factors (e.g., high cholesterol, diabetes, smoking, family history), and other test results to determine the best course of action.

**Conclusion:**

A positive heart CT scan is a significant finding that suggests the presence of coronary artery disease, but it is not the final word. Your doctor will use this information, along with other clinical data, to guide your treatment plan. It’s important to discuss the results in detail with your doctor to understand what they mean for your specific situation and what steps you should take next.

# 84. My doctor says that my heart MRI is positive, how certain is that?

When your doctor says your heart MRI is "positive," it typically means that the imaging has revealed findings consistent with a specific condition or abnormality. However, the certainty of the diagnosis depends on several factors:

1. **Nature of the Findings**: The MRI may show clear evidence of a condition (e.g., scar tissue, inflammation, or structural abnormalities), which can make the diagnosis highly certain. Other findings might be less definitive and require further testing or correlation with symptoms.
2. **Clinical Context**: The interpretation of the MRI is often combined with your symptoms, medical history, and other test results (e.g., blood tests, ECG, or echocardiogram). This context can increase the certainty of the diagnosis.
3. **Radiologist's Expertise**: The accuracy of the interpretation depends on the experience and skill of the radiologist and your doctor in analyzing MRI results.
4. **Follow-Up Testing**: In some cases, additional tests (e.g., a biopsy, stress test, or advanced imaging) may be needed to confirm the diagnosis.

If you're unsure about the certainty of the diagnosis or what the results mean for your health, it’s important to ask your doctor for clarification. They can explain the specific findings, the level of confidence in the diagnosis, and the next steps in your care.
