# Supplementary material for: Large Language Models for Patient Education in Cardiovascular Imaging: Prospective Observational Comparative Study
Source: JMIR Form Res. 2026 Aug 26;10:e95883. doi: 10.2196/95883 (PMC13559148; doi:10.2196/95883)
Supplement: Supplementary file 3 [file formative_v10i1e95883_app3.docx]

# 1. I am undergoing cardiac CT. What is a cardiac CT scan, and Why might I need one?

A **cardiac CT scan** (computed tomography scan of the heart) is a specialized imaging test that uses X-rays and sophisticated computer processing to capture highly detailed cross-sectional images of your heart and its blood vessels. Here’s a more detailed look at what it is and why you might need one:

**What Is a Cardiac CT Scan?**

1. **Imaging Technology**: A cardiac CT uses an X-ray machine that rotates around you, taking multiple pictures (slices) of your heart from different angles. A computer then assembles these slices into detailed 2D or 3D images.
2. **Possible Use of Contrast**: Often, a contrast dye (iodine-based) is injected into a vein during the test. The contrast dye highlights your coronary arteries and other blood vessels, enabling your medical team to see blockages or narrowed areas more clearly.
   - *Note*: In certain situations (such as a “calcium scoring” test), no contrast is needed. Instead, the scan measures calcium deposits in the coronary arteries, an indicator of plaque buildup.
3. **Noninvasive but Uses Radiation**: While it is a noninvasive test—no incisions or catheters are involved—it does expose you to a small amount of ionizing radiation. Modern CT scanners and techniques help minimize the dose as much as possible.

**Why Might You Need a Cardiac CT Scan?**

1. **Check for Coronary Artery Disease (CAD)**
   - **Evaluate Blockages or Narrowing**: If you have risk factors such as high blood pressure, high cholesterol, smoking history, diabetes, or a family history of heart disease, a cardiac CT can help reveal plaque buildup or narrowing in the coronary arteries.
   - **Calcium Scoring**: A “coronary calcium scan” measures calcium deposits in the artery walls. Higher calcium scores can indicate a higher risk of future heart issues, even before you show symptoms.
2. **Investigate Unexplained Symptoms**
   - **Chest Pain or Shortness of Breath**: If you experience ongoing chest pain or shortness of breath, and other initial tests (like an ECG or stress test) are inconclusive, a cardiac CT can clarify whether your heart’s structure or vessels are the culprit.
3. **Pre-Surgical or Pre-Procedure Planning**
   - **Heart Surgery Planning**: If you are being evaluated for bypass surgery or a valve procedure, a cardiac CT can give your surgical team a detailed road map of your heart’s anatomy.
   - **Transcatheter Procedures**: For certain catheter-based treatments (e.g., transcatheter aortic valve replacement, TAVR), a CT can help measure key structures precisely.
4. **Assess Heart Muscle and Structures**
   - **Structural Abnormalities**: A cardiac CT can detect problems with the heart muscle (such as hypertrophy or thinning), congenital heart defects, aneurysms, and certain issues with heart valves.
5. **Follow-Up on Known Conditions**
   - **Monitoring**: If you have a known condition like stents or bypass grafts from a prior procedure, your cardiologist may use a cardiac CT to track changes over time and ensure everything remains open and functional.

**Key Takeaways**

- A **cardiac CT** provides **detailed images** of your heart’s anatomy and blood vessels.
- It is **noninvasive** but involves **X-ray radiation** and, frequently, **intravenous contrast**.
- Reasons for a cardiac CT scan vary: from clarifying unexplained symptoms and assessing your **heart disease risk** to planning or evaluating **heart-related procedures**.
- If you have risk factors for heart disease (e.g., high blood pressure, high cholesterol, smoking, diabetes) or unexplained chest symptoms, this test may help your doctor diagnose or rule out potentially serious heart conditions.

If you have any concerns or questions about the procedure—such as how to prepare, what it entails, or your specific risks—be sure to discuss them with your healthcare provider. They can explain why a cardiac CT scan is right for you and how it fits into your overall care plan.

# 2. I am undergoing cardiac MRI. What is a cardiac MRI, and why might I need one?

A **cardiac MRI** (magnetic resonance imaging of the heart) is a noninvasive, highly detailed imaging test that uses powerful magnets and radio waves—rather than X-rays—to create clear and precise pictures of your heart and surrounding blood vessels. It is considered one of the gold-standard imaging techniques for evaluating the structure and function of the heart.

Below is an overview of *what a cardiac MRI is* and *why you might need one*:

**What is a Cardiac MRI?**

1. **Imaging Method**
   - Uses a strong magnetic field and radio waves to produce detailed cross-sectional images.
   - Does not use ionizing radiation (unlike a CT scan), making it safer in terms of radiation exposure.
2. **Detail and Accuracy**
   - Provides high-resolution images of the heart’s muscles, valves, chambers, and surrounding vessels.
   - Can identify subtle changes such as scarring or inflammation in the heart muscle.
3. **Functional Assessment**
   - Allows doctors to measure how effectively your heart is pumping blood (ejection fraction).
   - Can show blood flow patterns, valve function, and the movement of the heart walls in real-time.

**Why Might You Need One?**

1. **Evaluation of Heart Structure and Function**
   - To assess the size and shape of your heart’s chambers.
   - To measure cardiac output and ejection fraction.
   - To evaluate heart muscle abnormalities, such as cardiomyopathies.
2. **Diagnosis After a Heart Attack or Other Damage**
   - Can detect areas of scar tissue or reduced blood flow.
   - Helps determine how much damage a heart attack may have caused.
3. **Investigation of Congenital Heart Conditions**
   - In children and adults with congenital (present-at-birth) heart problems, an MRI provides a clear roadmap of structural issues to guide treatment or surgical plans.
4. **Assessing Cardiomyopathies and Inflammatory Diseases**
   - Identifies diseases that thicken the heart muscle (hypertrophic cardiomyopathy) or weaken it (dilated cardiomyopathy).
   - Detects inflammation (myocarditis) or infiltration (e.g., sarcoidosis, amyloidosis).
5. **Valvular Heart Disease**
   - Evaluates the function of heart valves (e.g., aortic, mitral valves) to check for leaks or narrowing (regurgitation or stenosis).
6. **Planning and Monitoring Treatment**
   - Helps decide whether certain procedures (such as catheter-based or surgical interventions) might be required.
   - Monitors heart function over time to see how well treatments or medications are working.

**What to Expect During a Cardiac MRI**

- **Preparation:** You’ll typically lie down on a table that slides into the MRI scanner. You may be given contrast dye (gadolinium) intravenously to highlight blood vessels or damaged tissue.
- **Noise and Comfort:** The machine can be loud (it makes repeated tapping, thumping noises). Often, you’ll be given earplugs or headphones.
- **Breath-Holding:** You may be asked to hold your breath briefly to get sharper images.
- **Duration:** A standard cardiac MRI can take anywhere from 30 to 60 minutes or longer, depending on how many images are needed.

A **cardiac MRI** is a powerful tool to give your medical team detailed insights into how your heart is working. If your healthcare provider recommends one, it is generally to investigate or confirm a diagnosis, monitor the progression of a known condition, or help plan the best treatment strategy for your heart health. If you have concerns about the test or any part of the preparation process, it’s always best to discuss them with your doctor or cardiologist ahead of time.

# 3. I am undergoing heart CT. How would this test help diagnose my condition?

I’m not a medical professional, but I can share general information about how a heart (cardiac) CT scan is commonly used and what it can reveal. A heart CT, sometimes called a cardiac CT or coronary CT, uses X-rays and computer technology to create detailed images of your heart and blood vessels. Here are a few ways it can help with diagnosis:

1. **Identifying Coronary Artery Disease (CAD):**
   - A CT coronary angiogram (CTA) can visualize the coronary arteries that supply blood to your heart. It helps detect any blockages or narrowing (stenosis) caused by plaque buildup.
   - It can also detect calcifications (calcium deposits in the arteries), often reported as a “calcium score.” A high calcium score may indicate an increased risk of heart disease.
2. **Evaluating Heart Structure and Function:**
   - A CT scan can provide detailed images of the heart’s chambers, valves, and surrounding structures. This can help detect abnormalities such as aneurysms (ballooning of a blood vessel), congenital heart defects, or problems with the heart valves.
3. **Assessing Heart Muscle and Surrounding Tissues:**
   - Heart CT can also look at the thickness of the heart muscle (useful if there’s concern about hypertrophic cardiomyopathy or other cardiomyopathies) and detect any masses or tumors in or around the heart.
4. **Guiding Further Treatment:**
   - The results from a heart CT can help doctors determine if you need additional procedures (e.g., cardiac catheterization, stent placement) or if medications and lifestyle modifications might be enough to manage your condition.

**Why Heart CT Over Other Tests?**

- **Noninvasive:** Unlike cardiac catheterization (which involves inserting a catheter into your arteries), a cardiac CT doesn’t require invasive procedures, though it does involve the use of contrast dye in many cases (CT angiogram).
- **Speed and Detail:** Modern CT scanners are fast and can capture detailed images of the heart and blood vessels in a single breath-hold, making the process relatively quick.

**What to Expect**

- You’ll typically lie on a table that slides into the CT machine.
- You may be given a contrast dye through an IV to help highlight blood vessels.
- Sometimes a beta-blocker is administered (if appropriate) to slow down your heart rate for clearer images.
- The scanner will take images in short bursts while you hold your breath.

**Important Note:** The specific benefit of a heart CT scan for *your* condition depends on why your doctor recommended it—whether it’s to check for coronary artery disease, structural issues, or something else. It’s always a good idea to discuss the results with your cardiologist or healthcare provider. They can interpret the findings in the context of your symptoms, medical history, and any other tests you’ve had, and then decide on the most appropriate next steps or treatment options.

If you have questions about how this test specifically applies to *your* situation—especially concerning risks, benefits, or alternatives—your healthcare provider is the best source of personalized information.

# 4. I am undergoing heart MRI. How would this test help diagnose my condition?

A cardiac MRI (Magnetic Resonance Imaging) is a powerful, noninvasive test that uses a magnetic field and radio waves (rather than X-rays) to create detailed images of your heart. These images can reveal information that helps physicians assess your heart’s structure, function, blood flow, and even the tissue composition of the heart muscle. Here’s how it typically contributes to diagnosis:

1. **Detailed Structural Views**
   - **Heart chambers and walls**: A cardiac MRI provides clear images of the thickness and shape of each of the heart’s chambers. This level of detail helps identify any abnormal thickening, thinning, or problems with the heart’s muscular walls.
   - **Valves**: It can show if the heart valves are opening and closing properly or if there is any leakage (regurgitation) or narrowing (stenosis).
2. **Assessment of Heart Function**
   - **Ejection fraction**: Cardiac MRI is considered the gold standard for measuring how well the heart pumps blood (the ejection fraction). Accurately knowing how much blood is being pumped out with each heartbeat can guide treatment decisions.
   - **Cardiac output and volumes**: The test gives precise measurements of the volume of blood in the ventricles, both before and after pumping, which helps detect conditions like heart failure or cardiomyopathies.
3. **Tissue Characterization**
   - **Scarring or fibrosis**: MRI can detect areas of scarring in the heart muscle caused by past heart attacks or inflammation. The ability to differentiate healthy from damaged tissue helps doctors pinpoint the extent and age of any damage.
   - **Inflammation or infiltration**: Certain conditions (e.g., myocarditis, sarcoidosis, amyloidosis) can be diagnosed by looking at how tissues take up and release contrast agents, revealing signs of inflammation or abnormal proteins in the heart muscle.
4. **Blood Flow & Vascular Assessment**
   - **Blood vessels**: Cardiac MRI can assess blood flow patterns in the major vessels, which is especially helpful in diagnosing congenital heart defects or vascular abnormalities.
   - **Stress imaging**: Sometimes, doctors perform stress cardiac MRI—often using medications rather than exercise—to see how blood flow to the heart muscle changes under stress, helping identify areas at risk for ischemia (reduced blood flow).
5. **Identification of Congenital Abnormalities**
   - **Congenital heart disease**: It provides high-resolution images that help detect holes in the heart (such as atrial or ventricular septal defects) or abnormal connections between vessels, even in very complex congenital issues.
6. **Planning and Monitoring Treatment**
   - **Surgical or procedural planning**: If surgery or a procedure is needed, the detailed 3D images from an MRI guide surgeons on the best approach.
   - **Tracking disease progression**: By repeating MRIs over time, doctors can see if the heart’s structure or function is improving, stabilizing, or worsening.

**What This Means for You**

- **Precision in Diagnosis**: By combining the structural, functional, and tissue-level details, your medical team can get a comprehensive picture of your heart’s health.
- **Less Radiation**: MRI does not use ionizing radiation (unlike CT scans), so it’s safer in that regard, especially when multiple follow-up scans may be needed.
- **Potential Next Steps**: Depending on what the MRI shows, your care team may recommend changes to medical therapy, interventional procedures, or, in some cases, surgery.

If you have additional questions—such as why MRI might be preferred over an ultrasound (echocardiogram) or a CT scan in your specific case—be sure to discuss these with your cardiologist. They can explain exactly how the test’s findings will guide your treatment plan based on your unique condition.

# 5. I am undergoing heart CT. Which is considered more accurate CT or MRI?

**Short Answer**
It depends on what exactly you’re trying to diagnose or evaluate. In general:

- **CT (especially coronary CT angiography)** is often preferred for detailed imaging of the coronary arteries (to check for blockages or narrowing) and has high accuracy for detecting coronary artery disease.
- **MRI (cardiac MRI)** is typically the gold standard for detailed evaluation of heart function (ventricular size, ejection fraction), tissue characterization (looking for scar tissue or inflammation), and specific heart muscle diseases.

**How They Differ in Accuracy**

1. **Coronary Artery Assessment**
   - **CT**: When looking for coronary artery disease (blockages), a **coronary CT angiogram** is highly sensitive and specific, meaning it’s very good at ruling out and detecting significant blockages.
   - **MRI**: While MRI can provide some information about blood flow and coronary arteries with advanced techniques, it is generally **not** the first-line test for detailed imaging of coronary artery narrowing or plaque.
2. **Heart Function and Tissue Characterization**
   - **CT**: Can give a snapshot of heart anatomy and function (how well the ventricles are pumping), but it typically involves radiation and might not be repeated frequently unless absolutely necessary.
   - **MRI**: Considered the **gold standard** for many aspects of heart function—measuring ejection fraction, cardiac volumes, and looking for fibrosis or scar tissue in the heart muscle. MRI excels at providing detailed tissue characterization (e.g., detecting areas of myocarditis or damage from a prior heart attack).
3. **Valvular Heart Disease**
   - **CT**: Good for anatomical detail and assessing calcification in valves (e.g., aortic valve).
   - **MRI**: Provides a precise measurement of flow across valves, regurgitation fraction, and function over time without radiation.
4. **Radiation and Safety**
   - **CT**: Uses X-rays, so there is exposure to ionizing radiation, although modern scanners try to limit the dose.
   - **MRI**: Uses magnetic fields and radio waves (no ionizing radiation), but can be more challenging for people with certain implants or devices.

**Which One Is “More Accurate”?**

**Accuracy** really depends on what you need to find:

- If your doctor wants to rule out or confirm **coronary artery blockages**, a cardiac **CT** scan (coronary CT angiography) is often very accurate and less invasive than a traditional catheter angiogram.
- If the question is about **heart muscle disease**, **inflammation**, or detailed functional assessment, a **cardiac MRI** is usually the best test for accuracy.

**Bottom Line**

No single test is universally “more accurate” for all heart conditions. **CT** is often best for *coronary artery* imaging, while **MRI** excels at *functional and tissue-level* evaluation of the heart. In many cases, **both** imaging tests can be complementary. Your healthcare provider will recommend the one that best addresses your specific diagnostic question.

# 6. I am undergoing heart MRI. Which is considered more accurate CT or MRI?

I’m not a medical professional, but I can share some general information that might help. In cardiology, both CT (Computed Tomography) scans and MRI (Magnetic Resonance Imaging) are widely used for visualizing the heart, but they each excel in different areas:

**1. Heart structure and function (MRI often called “the gold standard”):**

- **Cardiac MRI** is considered the best imaging modality for evaluating heart muscle structure, function, and tissue characterization (for example, detecting scarring, inflammation, or fibrosis).
- It provides very detailed information about how well the heart is pumping, measurements of chamber size and wall thickness, and can detect subtle heart muscle diseases.
- There is *no ionizing radiation* in MRI, though it can be more time-consuming and expensive, and some people find the enclosed MRI space uncomfortable.

**2. Coronary arteries (CT often favored for speed and detail):**

- **Cardiac CT** (especially Coronary CT Angiography) is excellent for visualizing the coronary arteries themselves. It shows calcifications and blockages quickly and with high accuracy.
- It’s less time-consuming and widely available. However, CT uses ionizing radiation and sometimes requires an iodine-based contrast agent, which may not be suitable for all patients.
- While newer MRI techniques can look at coronary arteries, CT is still the more common and practical choice if the main question is about blockages or narrowing in the coronary arteries.

**Which is “more accurate”?**

- It really depends on *what* your doctors are looking for. If it’s about **heart muscle health, function, or detailed tissue characterization**, MRI is generally considered the gold standard and “more accurate” for those purposes.
- If it’s about **coronary artery blockages**, CT angiography is often the first-line imaging method because it’s faster and gives clear information about the arteries.

Because you’re scheduled for an MRI, there’s a good chance your cardiologist wants detailed information about your heart’s structure and function, or to evaluate specific tissue characteristics—areas where MRI shines. If you have further questions about why MRI was chosen for you, it’s always best to discuss with your cardiologist, who can explain the reasoning based on your specific situation.

# 7. Why would my doctor recommend a cardiac CT instead of a cardiac MRI?

Doctors may recommend a cardiac CT (computed tomography) instead of a cardiac MRI (magnetic resonance imaging) for a variety of reasons, typically relating to what specific information they need and the patient’s overall situation. Some common factors include:

1. **Visualizing the Coronary Arteries**
   - **Coronary CT angiography (CTA)** is particularly good at imaging the coronary arteries to detect blockages or plaque buildup. While MRI can provide excellent soft-tissue details, it generally is not the go-to test for detailed visualization of the coronary arteries.
   - CT scans are especially useful in ruling out or identifying significant coronary artery disease quickly.
2. **Speed and Availability**
   - A cardiac CT can be performed relatively quickly, which can be crucial in urgent or emergency scenarios.
   - MRI scans typically take longer (often 30–60 minutes or more), and the specialized equipment required may not be as readily available, especially in emergency settings.
3. **Detection of Calcifications**
   - A **Coronary Calcium Score** via CT is a common, noninvasive screening tool to assess the amount of calcium in the coronary arteries, which is an indicator of atherosclerotic plaque.
   - MRI does not detect calcium as well as CT does.
4. **Patient Factors/Contraindications to MRI**
   - Some people have implantable devices (e.g., pacemakers, defibrillators, certain types of metal implants) that might be contraindications for an MRI due to the strong magnetic fields involved.
   - Patients with severe claustrophobia or an inability to remain still for longer periods may tolerate the shorter CT scan better.
5. **Precision in Certain Structural Evaluations**
   - For certain structural heart issues—like evaluating cardiac valves, heart muscle function, or scarring—MRI might be preferred. However, for quickly assessing complex anatomy (such as the aorta or pulmonary veins), a high-resolution CT might be the simpler choice.

In short, the decision often comes down to the specific clinical question, the need for rapid and detailed coronary artery assessment, patient suitability for MRI, and practical considerations such as scan time and local availability. If your doctor suspects or wants to rule out significant coronary artery disease, or there’s a reason why you cannot undergo an MRI, a cardiac CT might be the preferred test.

# 8. Why would my doctor recommend a cardiac MRI instead of a cardiac CT?

Reasoned about cardiac MRI vs. CT for a few seconds

**Understanding the imaging options**

I'm comparing cardiac MRI and CT options, focusing on tissue characterization, radiation avoidance, and functional details. It's interesting to see how low kidney function or heart inflammation impacts these choices.

**Deciding on MRI over CT**

I’m gathering info on choosing MRI for tissue details, avoiding radiation, and assessing heart function, especially when examining valve dynamics and soft tissues over coronary artery disease.

Cardiac MRI (magnetic resonance imaging) and cardiac CT (computed tomography) are both valuable tools for evaluating the heart, but they each have distinct strengths and limitations. A doctor’s recommendation for a cardiac MRI over a cardiac CT often comes down to the specific information they need, as well as individual patient factors. Here are some of the most common reasons:

**1. Avoiding Radiation Exposure**

- **CT scans** use X-rays to produce images, which involves exposure to ionizing radiation.
- **MRI** does not use ionizing radiation, making it preferable for patients who require multiple follow-up scans over time (e.g., in young patients or those who have had many imaging studies) or who are otherwise considered high-risk for radiation.

**2. Superior Soft Tissue Characterization**

- **MRI** excels at differentiating between various types of soft tissue, including muscles, blood vessels, and abnormal tissue within the heart muscle.
- This detailed tissue characterization is particularly useful in diagnosing conditions like **myocarditis**, **cardiomyopathies** (e.g., hypertrophic, dilated, or infiltrative types), and in determining the presence of **scar tissue** or **fibrosis** after a heart attack.

**3. Detailed Assessment of Cardiac Function and Structures**

- **MRI** provides highly accurate measurements of ventricular volumes, ejection fraction, and wall motion. This can be critical in planning treatments such as valve interventions or in assessing the impact of heart failure.
- It also offers excellent visualization of the heart valves and blood flow without needing additional contrast agents in many cases.

**4. Specific Clinical Scenarios**

- **Congenital Heart Disease**: MRI is often preferred for complex anatomical and functional assessments in patients with congenital heart anomalies.
- **Viability Studies**: MRI (particularly with late gadolinium enhancement) can show whether areas of the heart muscle have viable tissue or scar tissue, aiding decisions about revascularization or other interventions.
- **Myocarditis or Inflammatory Conditions**: MRI can detect subtle edema or inflammation in the heart muscle that may not be evident on other imaging modalities.

**5. Certain Patient Factors**

- **Allergy or Contraindications to Iodinated Contrast**: Cardiac CT often requires iodinated contrast for clear imaging of coronary arteries and vessels. Patients who have severe allergies to iodinated contrast might benefit from MRI (which uses gadolinium-based contrast, if contrast is needed at all).
- **Need for Detailed Cardiac Tissue Information**: If your doctor suspects an infiltrative disease (e.g., sarcoidosis, amyloidosis) or wants to clarify ambiguous findings from other tests, MRI’s detailed soft tissue resolution can be crucial.

**6. When Speed or Coronary Artery Visualization Is Not the Priority**

- **CT** is often the first-line imaging modality to quickly visualize the **coronary arteries** (especially for detecting blockages) and for **cardiac calcium scoring** because it is fast and widely available.
- **MRI** typically takes longer and may be more challenging for patients who have difficulty staying still or holding their breath, but it gives more comprehensive tissue information.

**Summary**

In short, your doctor might recommend a **cardiac MRI** instead of a **cardiac CT** if the clinical situation requires detailed tissue characterization, if you need repeated imaging and want to avoid radiation, or if there’s a suspicion of specific conditions (like myocarditis, infiltrative cardiomyopathies, or complex structural heart disease) where MRI provides superior detail. Always discuss with your healthcare team about the benefits, risks, and any preparatory steps needed for the imaging study they recommend.

# 9. How do I know if I’m a good candidate for a cardiac CT?

I’m not a medical professional, but I can share general information to help you understand what factors often determine whether someone is a good candidate for a cardiac CT (also known as a heart CT scan). Ultimately, you should seek individualized advice from your healthcare provider, as they will consider your specific medical history and risk factors.

Here are some common considerations:

**1. Assessing Risk Factors**

Your doctor will typically look at your overall risk for coronary artery disease (CAD). Common risk factors include:

- High blood pressure (hypertension)
- Elevated cholesterol levels
- Diabetes
- Smoking history
- Obesity
- Family history of heart disease (especially close relatives diagnosed at a young age)
- Age (men over 45 and women over 55 often have a higher risk)

If you have multiple risk factors or moderate to high risk for CAD, your provider may discuss the possibility of a cardiac CT to evaluate the extent of plaque buildup in your coronary arteries.

**2. Symptoms Suggesting Heart Disease**

A cardiac CT might be recommended if you have signs or symptoms that could suggest coronary artery disease, such as:

- Chest pain or tightness (angina)
- Shortness of breath, especially on exertion
- Unexplained fatigue

Often, when symptoms are not conclusive—if you have borderline findings on tests like an EKG or stress test—your doctor may consider a cardiac CT to get a more detailed anatomical view of the coronary arteries.

**3. Clarifying Ambiguous Test Results**

A cardiac CT scan may be used when other diagnostic tests (e.g., a stress echocardiogram or nuclear stress test) have provided inconclusive or conflicting information. Because a cardiac CT can visualize the coronary arteries non-invasively, it can sometimes help avoid more invasive procedures like a cardiac catheterization.

**4. Coronary Calcium Scoring (Heart Scan)**

Some individuals without overt symptoms but with risk factors may undergo a “coronary calcium score” scan. This specific type of cardiac CT looks for calcified plaque in the coronary arteries and provides a calcium score.

- If your risk profile is borderline—meaning you’re not clearly low or clearly high risk—your doctor might recommend a calcium score to help refine your overall risk of heart disease.
- If your calcium score is high, it indicates more plaque buildup, suggesting a higher risk for future cardiac events, and might prompt more aggressive management of risk factors.

**5. Evaluation Before Certain Surgeries**

If you need heart surgery (like valve surgery) or a procedure that could be complicated by undiagnosed coronary artery disease, a cardiac CT might be used to assess your heart and coronary arteries beforehand.

**6. Understanding Potential Limitations**

While cardiac CT is a powerful tool, it isn’t the right test for every scenario. Factors include:

- **Radiation exposure:** Although modern CT technology aims to minimize radiation, it’s still higher than a simple chest X-ray.
- **Heart rate control:** A stable, relatively low heart rate (<65–70 bpm) is usually needed for the clearest images. People with higher heart rates may need medication (like beta blockers) to slow the heart temporarily if a diagnostic-quality scan is necessary.
- **Existing kidney issues:** The contrast dye used during some CT scans can pose risks if you have significantly impaired kidney function.

**7. Summary of Who May Benefit**

- Individuals with moderate or uncertain risk for heart disease who need clearer imaging.
- People with symptoms suggestive of CAD that are unexplained by simpler tests.
- Patients with inconclusive or intermediate findings on prior tests (stress tests, EKGs, etc.).
- Certain patients requiring surgical intervention where precise coronary artery imaging is crucial.
- Those who might benefit from a coronary calcium scan to clarify their risk profile.

**Important Next Steps**

1. **Consult Your Physician**: Always discuss personal risk factors (family history, lifestyle, etc.) and concerns with your healthcare provider.
2. **Review Other Tests**: Your doctor might order preliminary tests (like blood work or an EKG) first.
3. **Discuss Pros and Cons**: Talk about the benefits of a cardiac CT (detailed artery images) versus potential drawbacks (radiation, need for IV contrast if performing a CT angiography).
4. **Preparation**: If your heart rate is high, you may be asked to take medication to lower it. Fasting may be required for some types of scans.

**Bottom line:**
A cardiac CT can provide very useful information for patients with uncertain risk profiles or indeterminate test results. However, it’s best to rely on your physician’s recommendation, since your overall heart health, medical history, and risk factors will determine if you’re a good candidate.

# 10. How do I know if I’m a good candidate for a cardiac MRI?

I’m not a medical professional, but I can share some general information that might be helpful. Cardiac MRI (Magnetic Resonance Imaging) is typically used by healthcare providers to get detailed images of the heart’s structure and function. Whether you’re a good candidate often depends on your specific symptoms, medical history, and the diagnostic questions your doctor is trying to answer. Below are some common reasons for and considerations about having a cardiac MRI:

**1. Common Reasons to Consider a Cardiac MRI**

1. **Assessment of Heart Structure and Function**
   - Evaluating the size of the heart’s chambers, the thickness of the heart muscle walls, and blood flow patterns.
   - Checking for heart muscle damage (for example, after a heart attack or in myocarditis).
   - Evaluating cardiac function in conditions such as heart failure or cardiomyopathy.
2. **Checking for Congenital Heart Defects**
   - A cardiac MRI can provide a detailed look at complex congenital heart anomalies in both children and adults.
3. **Evaluating Heart Valve Issues**
   - Getting precise measurements of valve structure, blood flow through the valves, or valve leaks/narrowing.
4. **Detecting Scarring or Inflammation**
   - Visualizing areas of scarred heart tissue (e.g., from past heart attacks) or actively inflamed areas (e.g., in myocarditis).
5. **Further Assessment After Other Tests**
   - Sometimes, if a test such as an echocardiogram or cardiac CT scan shows something unusual, a cardiac MRI might help clarify the findings.

**2. Considerations That Affect Suitability**

1. **Safety Around Metal or Implants**
   - MRI uses powerful magnets. Certain types of metal implants (e.g., pacemakers, older stents, some joint replacements) may be problematic or require special MRI-compatible devices.
   - Always inform your doctor or MRI technician about any implants or metal in your body.
2. **Claustrophobia or Comfort in Enclosed Spaces**
   - MRI machines can feel confining. If you have severe claustrophobia, you might need medication to relax or seek a facility with an “open” MRI (though those are less common for cardiac imaging).
3. **Kidney Function**
   - Contrast agents used in MRI (like gadolinium) are processed by the kidneys. If your kidney function is significantly impaired, your doctor may decide against using certain contrasts or may use a different imaging test.
4. **Allergies**
   - Some people are allergic to contrast agents. Your healthcare provider will review allergies or past reactions to imaging dyes.
5. **Pregnancy**
   - MRI without contrast is generally considered safe in pregnancy, but guidelines vary depending on the trimester. MRI with contrast typically requires more caution.
6. **Physiological Stability**
   - You need to lie still for the duration of the scan. People who have severe difficulty lying down or holding still (e.g., due to back pain, breathing issues) may require special accommodations or sedation.

**3. Deciding if You’re a Good Candidate**

1. **Speak with Your Healthcare Provider**
   - The single most important step is consulting a doctor (cardiologist or primary care) who knows your medical history. They can help you decide whether the detailed information from a cardiac MRI is necessary or if another imaging method—like an echocardiogram, CT scan, or nuclear stress test—would be better.
2. **Review Your Medical Records and History**
   - If you have known heart issues (e.g., prior heart attacks, arrhythmias, unusual symptoms like chest pain or shortness of breath), your doctor might want a more detailed look at the heart muscle or valves using MRI.
3. **Assess the Risk-Benefit Balance**
   - Like all diagnostic tests, a cardiac MRI has potential benefits (better diagnosis, clearer pictures) and certain drawbacks (cost, time, issues with claustrophobia or implants). Your healthcare provider will weigh these before recommending it.

**4. Next Steps**

- **Consult a Cardiologist**
  - They can interpret any existing test results (like ECG, stress tests, echocardiograms) and decide if a cardiac MRI will add helpful information.
- **Communicate Any Concerns**
  - If you have implants, metal fragments, kidney issues, or anxiety about enclosed spaces, tell your doctor. They can plan accommodations or alternative tests.
- **Follow Pre-Scan Instructions**
  - If you proceed with a cardiac MRI, follow instructions about fasting, medication adjustments, or removing jewelry and accessories.

**Important Note**

This information is general and should not replace personalized medical advice. If you suspect you need a cardiac MRI or have heart-related concerns, it’s best to speak with a qualified healthcare professional who can evaluate your individual situation.

# 11. Do I need to fast before my cardiac CT?

**Figuring out fasting needs**

OK, let me see. For a cardiac CT, you might need to fast a few hours to reduce aspiration risk and help with sedation. No food for 4 hours, and no caffeine to keep a steady heart rate.

**Figuring out fasting guidelines**

For a standard CT with contrast, fasting for at least 2 hours if sedation is used is common. Avoid caffeine and smoking for 12 hours pre-test. Other guidelines recommend either 2 hours of fasting or avoiding solids for 4 hours.

Whether or not you need to fast (avoid eating and/or drinking) before a cardiac CT scan can vary depending on the specific protocol at your imaging center and whether or not you will receive IV contrast. However, here are some general guidelines that many facilities follow:

1. **Avoid heavy meals for at least 4 hours before the scan.**
   - Many centers recommend not eating a large meal shortly before your CT to help reduce the risk of nausea (especially if IV contrast is given) and to ensure that your heart rate remains stable.
2. **Avoid caffeinated beverages and nicotine for 12–24 hours prior.**
   - Caffeine (coffee, tea, soda, energy drinks) and nicotine can raise your heart rate, which can make it harder to get clear images of your heart. It’s often suggested to skip these for about a day before your test if possible.
3. **Light meal or clear liquids may be allowed.**
   - Some facilities allow a light meal up to a couple of hours before the exam; however, each center has its own protocols. You may be instructed to drink water freely but avoid other beverages.
4. **Follow any beta-blocker instructions.**
   - If your doctor or the radiology department has instructed you to take a beta-blocker (to lower heart rate for clearer images), be sure to follow directions precisely, including timing of doses and any meal restrictions.
5. **Confirm with your healthcare provider or imaging center.**
   - Practices and guidelines differ, so the most accurate instructions will come from the facility performing the scan. If you have diabetes or other medical conditions, be sure to mention this so staff can tailor instructions accordingly.

In short, **yes**, you are often advised to fast (at least from heavy meals) and avoid stimulants like caffeine before a cardiac CT—though exactly how long you must fast can differ. Always verify with your imaging center or doctor for the specific instructions they want you to follow.

# 12. Do I need to fast before my cardiac MRI?

Whether you need to fast before a cardiac MRI typically depends on the specifics of your test:

1. **Routine cardiac MRI (without sedation or certain stress agents):**
   - Usually, no fasting is required. You can eat and drink normally before most cardiac MRI scans.
2. **If sedation or anesthesia is planned:**
   - Your care team may ask you not to eat or drink for a certain number of hours beforehand (often 4–6 hours). This helps prevent complications during sedation.
3. **If a medication-based “stress test” is part of the MRI:**
   - Your doctor might ask you to avoid caffeine for 24 hours before the test and possibly not eat for a few hours beforehand.
   - This is because certain stress agents are less effective if you have caffeine in your system, and fasting may be recommended for patient comfort or for accurate test results.

Since recommendations can vary, the best step is to confirm with the imaging center or your cardiologist. They will give you the most accurate instructions based on your individual case—particularly if there are special considerations like sedation or stress agents. Otherwise, for a standard cardiac MRI, there is generally **no need to fast.**

# 13. I am undergoing cardiac CT. Can I take my usual medications on the morning of the test?

**Navigating medication rules**

OK, taking medications with water generally works fine for a cardio CT. Still, some meds might hit pause on results, so follow specific instructions.

I’m not a medical professional, but I can share general information that is usually given to patients before a cardiac CT. In many cases, you can continue taking your usual medications on the morning of the test. However, there are some exceptions and important considerations:

1. **Beta-blockers or other heart-rate controlling medications**
   - For many cardiac CT scans (especially coronary CT angiography), having a lower heart rate can help produce clearer images. Sometimes your doctor may adjust your dose of beta-blockers or ask you to take them at a specific time.
2. **Diabetes medications**
   - If you have diabetes and take insulin or other blood sugar–lowering medications, your doctor or the imaging facility may have special instructions—especially if you are required to fast for a certain period before the scan.
3. **Avoiding caffeine and stimulants**
   - Often you are asked to avoid caffeine (coffee, tea, energy drinks, chocolate) or other stimulants (decongestants in cold medicines) for at least 12–24 hours before a cardiac CT, as they can raise your heart rate, which may reduce image quality.
4. **Fasting requirements**
   - Some cardiac CT protocols require you to arrive with an empty stomach, so you might be asked not to eat or drink (other than small sips of water to take essential medications) for a few hours before the test.

Because recommendations can differ based on the type of cardiac CT you’re having, it’s always best to **contact the imaging center or your doctor’s office** for specific instructions. They can give you guidance tailored to your medical history and the exact test you’ll be undergoing.

# 14. I am undergoing cardiac MRI. Can I take my usual medications on the morning of the test?

**Assessing medication intake**

OK, let’s think this through: For standard MRIs, medications can be taken. However, stress imaging or sedation might require halting specific drugs like caffeine, xanthine derivatives, some beta-blockers, antiarrhythmics, and PDE5 inhibitors before the test.

**Weighing standard MRI**

For standard MRIs, holding off on usual medications is often unnecessary. For stress tests, caffeine and theophylline are generally the main stop-calindications.

**Determining medication protocols**

Hmm, I’m thinking about whether to pause beta blockers 24 hours before a stress MRI. General guidelines suggest regular medications are usually fine unless there’s sedation or contrast involved.

I’m not a medical professional, but I can share some general guidelines. In most cases, for a routine (non-stress) cardiac MRI, you can take your usual daily medications as normal—even on the morning of your test. However, there are a few important exceptions and considerations:

1. **If your cardiac MRI includes a “stress” portion** (sometimes done with medications like adenosine, regadenoson, or dobutamine to stress the heart), you might be asked to **hold certain medications** before the test—especially:
   - **Beta-blockers** (e.g., metoprolol, atenolol), as they can interfere with the heart-rate response needed during a stress test.
   - **Caffeine** or any products containing caffeine (coffee, tea, chocolate, some over-the-counter headache medications) for **at least 12–24 hours** beforehand, because it can block the effects of stress agents.
   - **Theophylline** (if you happen to use this for breathing issues), because it can also interact with stress agents.
2. **If you require sedation** for the MRI (some patients do if they experience claustrophobia or for other reasons), you might be asked to follow specific fasting instructions or modify certain medication schedules—especially if those medications cause drowsiness or interact with sedatives.
3. **Other medical conditions** (such as diabetes) might come with separate instructions about timing of oral medications or insulin on the morning of the test, especially if you need to fast.

Because each imaging facility and each patient’s medical situation can be different, the safest approach is to:

- **Follow any written or verbal instructions** given to you when the cardiac MRI was scheduled.
- **Call the MRI center or your cardiologist** a few days ahead to confirm if you need to stop or adjust any medications.

In general, unless you’ve been specifically advised otherwise—especially for a stress MRI or sedation—continuing your usual medications the morning of a standard cardiac MRI is common practice. But always double-check with your healthcare provider or the imaging facility to get the most accurate guidance for your situation.

# 15. I am undergoing cardiac CT. Are there any dietary restrictions before the test?

**Preparing for a cardiac CT scan**

I'm piecing together the instructions for a cardiac CT scan. Typically, avoiding caffeine and heavy food is advised. Also, skipping certain medications like PDE5 inhibitors is recommended to stabilize the heart rate.

**Confirming guidelines**

Okay, let's clarify the essential steps: avoid eating or drinking for a few hours before the test, steer clear of caffeine to prevent heart rate spikes, and skip intense exercises in the lead-up.

I’m not a medical professional, but I can share general guidelines commonly recommended for patients undergoing a cardiac CT. Always follow the specific instructions given by your healthcare provider, since exact requirements can vary from one facility to another. However, here are some typical considerations:

1. **Fasting**
   - Many centers request that you avoid eating solid food for about 2–4 hours prior to the scan, especially if you are receiving IV contrast. This helps reduce the chance of nausea and can improve imaging quality.
2. **Avoiding Caffeine and Stimulants**
   - It’s often advised to avoid caffeine (coffee, tea, soda, energy drinks, chocolate) for at least 12–24 hours before a cardiac CT. Caffeine can increase your heart rate or cause irregular beats, making it more challenging to get clear images of the heart.
   - In addition to caffeine, you may be instructed to avoid nicotine (including smoking, vaping, or nicotine patches) and other stimulants before the test.
3. **Medications**
   - Your doctor may have specific instructions about blood pressure or heart-rate controlling medications (e.g., beta-blockers). In some cases, you’ll continue to take your usual medications; in others, the radiology or cardiology team may modify the timing or dosage. If you’re uncertain, clarify with your healthcare provider.
4. **Hydration**
   - While fasting typically means no solid food, you are often encouraged to stay hydrated with water unless instructed otherwise. However, always confirm with the imaging facility if sipping small amounts of water is permitted before the exam.
5. **Alcohol**
   - Generally, it’s wise to avoid alcohol for at least 24 hours before your test, since alcohol may affect heart rate and blood pressure.
6. **Individual Facility Policies**
   - Preparation instructions can vary from one center to another, depending on the type of CT scanner, the use of contrast, and your personal medical history.

To be safe, always follow the specific guidelines your doctor or the imaging center provides. If you are unsure about any dietary or medication instructions, reach out to your healthcare provider before the test date.

# 16. I am undergoing cardiac MRI. Are there any dietary restrictions before the test?

**Assessing dietary needs**

I'm thinking about the lack of dietary restrictions for standard MRI but noting the need to avoid caffeine for 12-24 hours before a stress MRI with a vasodilator.

**Evaluating dietary needs**

For standard cardiac MRI, no specific diet is required. For those using sedation, fasting might be necessary. Pharmacological stress tests indicate avoiding caffeine for 12 hours beforehand.

Whether or not there are dietary restrictions before a cardiac MRI often depends on the specific type of scan you’re having and whether you will be receiving any form of sedation or undergoing a pharmacologic stress test. In most cases, a standard (non-stress) cardiac MRI does not have significant dietary restrictions. However, here are some general guidelines:

1. **If you are NOT having a pharmacological (stress) MRI or sedation:**
   - Typically, you can eat and drink as you normally would.
   - You may be asked to avoid heavy meals immediately before the test to minimize discomfort from lying still.
2. **If you are having a pharmacologic (stress) MRI** (e.g., using adenosine, regadenoson, or dobutamine):
   - **Caffeine:** You will usually be asked to avoid all caffeinated products (coffee, tea, chocolate, soda, certain medications) for at least 12–24 hours prior to the test. Caffeine can interfere with the stress agent (especially adenosine or regadenoson), reducing its effectiveness or causing inaccurate results.
   - **Meals:** Some centers suggest not having a large meal just before the stress MRI so that you’re comfortable during the test; however, strict fasting is usually not required unless instructed otherwise.
3. **If you will receive sedation** (which is sometimes used for patient comfort if claustrophobia or difficulty lying still is a concern):
   - Many facilities will ask you not to eat or drink for a certain period (often 4–6 hours) before your appointment to reduce the risk of aspiration if sedation is given.

Because practices vary, **always confirm with your radiology or cardiology team** regarding the specific instructions for your situation. They’ll be able to provide the most accurate guidance based on the exact type of MRI you’re scheduled to have and your personal medical history.

# 17. I am undergoing heart CT. Will I need blood tests or other preliminary checks beforehand?

**Working through typical preparations**

For a heart CT, it's common to undergo kidney function tests, possibly monitor the heart rate, ensure no contrast allergies, and likely have an ECG.

I’m not a medical professional, but I can share general information about what is commonly done before a cardiac CT (heart CT). Whether you need any tests beforehand can vary based on your health status, the type of CT scan, and the specific instructions from your doctor or imaging center. Here are a few things that are often considered:

1. **Kidney Function (Creatinine or eGFR blood test)**
   - If you’re scheduled for a cardiac CT *with contrast* (often called a CT angiogram, or CTA), the radiology team may check your kidney function via a simple blood test (creatinine and/or estimated Glomerular Filtration Rate, eGFR). This is to make sure your kidneys can safely clear the contrast dye used during the scan.
2. **Allergies or History of Contrast Reactions**
   - If you’ve had any previous reaction to contrast dye (iodine-based contrast), you should let your healthcare provider know. In some cases, they may do an allergy test or prescribe pre-medications (such as antihistamines or steroids) if you are at higher risk for an allergic reaction.
3. **Heart Rate and Blood Pressure Check**
   - For many cardiac CT exams—especially coronary CT angiograms—getting a clear image of the heart’s blood vessels is easier when your heart rate is steady (often under ~60 bpm). Sometimes a beta-blocker or other medication is given before or during the scan to slow the heart rate. Your medical team will check your blood pressure and heart rate to decide if any pre-medication is needed.
4. **Electrocardiogram (ECG)**
   - An ECG tracing during the scan (or just before it) helps time the imaging with your heartbeat. Sometimes your doctor might do a quick ECG beforehand to ensure there are no rhythm issues that need to be managed.
5. **Other Blood Tests (Less Common)**
   - Other than kidney function tests, additional labs (like thyroid function) aren’t always necessary for a typical heart CT. However, if you have a known thyroid issue or other significant health conditions, your doctor may want to check related labs.
6. **Pregnancy Test (for women of childbearing age)**
   - If there’s any chance you could be pregnant, a urine or blood pregnancy test is usually done before the scan, as radiation exposure is generally avoided during pregnancy unless absolutely necessary.
7. **Fasting Instructions**
   - You’ll often be asked to avoid caffeine, nicotine, or sometimes all food and drink for a few hours before the exam. This helps minimize factors (like an elevated heart rate from caffeine) that might reduce scan quality.

**Next Steps:**

- **Ask your doctor or radiology center** for specific instructions. They’ll clarify exactly which tests (if any) they need from you based on your medical history and the type of heart CT they plan to perform.
- If you have any **underlying conditions** (like kidney disease, allergies to contrast, or certain heart rhythm issues), it’s especially important to discuss those beforehand so that the medical team can take any necessary precautions.

Remember, your healthcare provider is the best person to give you advice tailored to your situation. This information is for general guidance, so be sure to verify the steps required for your particular test.

# 18. I am undergoing heart MRI. Will I need blood tests or other preliminary checks beforehand?

**Clarifying MRI requirements**

I’m thinking through the need for blood tests to assess kidney function before an MRI with contrast. Sedation is rare and usually limited to claustrophobic patients only.

I’m not a medical professional, but I can share general information that may help you prepare for a heart MRI. Requirements can vary depending on your specific situation and the protocols of the imaging center or hospital conducting the test. Therefore, it’s always best to follow the advice of your cardiologist or radiologist. That said, here are some commonly encountered scenarios:

**1. Kidney Function Tests (Blood Tests):**

- If your doctor plans to use a gadolinium-based contrast agent during the MRI, you might be asked to have a blood test (often checking serum creatinine or estimated glomerular filtration rate, eGFR) to ensure your kidneys can safely filter the contrast.
- This is typically required if there are concerns about kidney function, if you have a history of kidney problems, or if you are older or have other risk factors.

**2. Metal or Device Checks:**

- You will be asked about any metal implants, pacemakers, implantable cardioverter defibrillators (ICDs), or other metallic devices (including certain stents, surgical clips, etc.) in your body.
- You may need to provide documentation about the type of device or implant to confirm it is MRI-compatible. This usually doesn’t require a blood test but is an important safety screening.

**3. Allergies and Medication Review:**

- If you have known allergies (especially to contrast agents) or a history of allergic reactions, let your care team know.
- They might recommend pre-medication (e.g., with antihistamines or steroids) in rare cases of known contrast allergies.

**4. Other Preliminary Checks:**

- Some centers may measure vital signs (blood pressure, heart rate) before the scan.
- If you are claustrophobic or anxious about the MRI, discuss this beforehand with your doctor. They may prescribe a mild sedative in some cases, which may come with its own guidelines.

**Summary:**

- A routine, non-contrast heart MRI often does not require blood tests. However, if contrast is used or if there’s any suspicion of kidney issues, a blood test to check kidney function is standard practice.
- You’ll also go through a questionnaire regarding implants, metal in the body, or allergies.
- Always confirm the specific requirements with your medical team or the imaging center, since practices vary.

If you have more specific concerns—such as known kidney issues, metal implants, or severe allergies—be sure to discuss these thoroughly with your doctor or the MRI facility well in advance. They can give you the most accurate guidance tailored to your situation.

# 19. I am undergoing heart CT. What should I wear to the appointment?

**Choosing appropriate attire**

For a heart CT scan, opting for comfortable, metal-free clothing is ideal. Avoid metal snaps, zippers, and jewelry to ensure clear imaging.

When preparing for a heart CT scan, it is generally best to wear comfortable, loose-fitting clothing that does not contain metal. Below are a few tips on what to wear and how to prepare:

1. **Avoid Metal**
   - Metal clasps, underwire bras, zippers, or snaps can create artifacts (unwanted marks) on the images.
   - If your clothing has metal, you may be asked to change into a hospital gown before the scan.
2. **Choose Comfortable, Loose-Fitting Clothes**
   - Wear a shirt or top that you can easily remove if necessary.
   - If you need an IV line for contrast, it’s easier if your sleeves can be rolled up or if your shirt can be unbuttoned.
3. **Limit Jewelry and Accessories**
   - Remove necklaces, watches, earrings, or any other metal items before the scan to avoid image interference.
4. **Be Prepared to Change if Needed**
   - Even if you dress carefully, your radiology department might give you a gown.
   - Dress in layers if you get cold easily—most facilities provide blankets, but it can help to have a sweater or jacket without metal parts.
5. **Follow Any Additional Instructions from Your Healthcare Team**
   - Depending on your specific situation, your doctor might have additional guidelines about eating or drinking before the scan (especially if you are receiving contrast material).
   - Let the technologist know if you have any implanted medical devices (pacemakers, stents, etc.) or other medical conditions that might affect the imaging process.

By keeping metal to a minimum and wearing comfortable clothing, you can help ensure your CT scan goes smoothly and reduce the time spent changing or removing items. If you have any doubts or special considerations (like a pacemaker, or known allergies to contrast materials), reach out to the imaging center or your doctor before your appointment.

# 20. I am undergoing heart MRI. What should I wear to the appointment?

**Choosing suitable attire**

I’m thinking through comfortable, loose-fitting clothing with no metal for a heart MRI appointment, like shorts or sweatpants, and avoiding jewelry or metallic items.

When preparing for a heart MRI, it’s best to dress in a way that minimizes or eliminates metal and allows you to lie comfortably and still during the scan. Here are some general guidelines:

1. **Wear comfortable, loose-fitting clothing**
   - Avoid clothing with zippers, snaps, or buttons containing metal.
   - Sweatpants or athletic pants without metal drawstring tips or zippers are a good choice.
2. **Skip underwire bras**
   - If you wear a bra, choose one without an underwire or metal fasteners. Some people opt for a sports bra with no metal components.
3. **Remove all metal accessories**
   - Jewelry, watches, belts with metal buckles, hair clips, and bobby pins must be taken off before the MRI.
   - You will usually be asked to store these items in a secure locker before your scan.
4. **Check with your MRI facility about changing into a gown**
   - Some facilities provide a hospital gown for you to wear during the scan.
   - Ask ahead of time if you need to bring an extra set of clothes or if you can wear your own clothes if they meet the metal-free requirement.
5. **Avoid metallic makeup or lotions**
   - Certain cosmetics (especially glitter or metallics) or lotions can contain trace amounts of metal. It’s best to go without them on the day of the MRI to avoid interference with the images.
6. **Follow facility instructions**
   - Every MRI center has slightly different guidelines and forms you might need to fill out (e.g., for medical implants or devices).
   - If you have a pacemaker, stent, or other implanted device, discuss this ahead of time with your care team. They will have special instructions or precautions.

In summary, wear simple, metal-free, comfortable clothing, remove all accessories, and follow your MRI facility’s instructions to ensure your heart MRI goes smoothly and safely. If you have any concerns about what is permissible, call the radiology department before your appointment for clarification.

# 21. I am undergoing heart CT. Do I need to avoid strenuous activity or exercise before the test?

**Considering CT scan prep**

I’m gathering info on avoiding strenuous activity or exercise before a heart CT scan, focusing on caffeine and smoking restrictions, and avoiding exercise for 12 to 24 hours to ensure optimal imaging quality.

**Modifying lifestyle**

I'm detailing lifestyle adjustments before a heart CT scan: avoiding strenuous exercise, excessive caffeine, or smoking. The aim is to ensure a stable heart rate, particularly if a beta-blocker is used during the test.

**Consulting guidelines**

Now I'm noting that heart CT scan guidelines can vary by facility. Specific instructions from the American Heart Association or consultation with the testing center are recommended for optimal preparation.

I’m not a medical professional, but in general, most facilities that perform cardiac CT (including CT angiograms) recommend avoiding strenuous exercise for several hours—sometimes up to 24 hours—before your scan. The main reason is that intense exercise can raise your heart rate and/or blood pressure, which can make it harder to get clear images of the heart during the test.

**Why does it matter?**

- A stable, lower heart rate helps produce clearer, more detailed images.
- If your heart rate is too high, you might need medication (often a beta-blocker) to slow it down before the scan, or the images might not turn out as clearly as desired.

**Typical pre-test guidelines for a cardiac CT might include**:

1. **Avoid caffeine** (coffee, tea, soda, energy drinks, chocolate) for about 12–24 hours before the test. Caffeine can raise your heart rate and sometimes cause irregular beats.
2. **Avoid nicotine** for several hours beforehand for the same reason.
3. **Skip strenuous exercise** or heavy physical activity (running, intense cardio, heavy lifting) for several hours—often recommended 12–24 hours prior.
4. **Follow your facility’s fasting instructions** if you’re told not to eat or drink for a certain number of hours before the scan.
5. **Take (or hold) medications** exactly as instructed. Some centers provide a beta-blocker or ask that you take your regular heart medications in a specific way.

Because instructions can vary depending on the exact type of cardiac CT, your medical history, and the facility’s protocols, it’s always best to confirm the specific guidelines with your doctor or the imaging center performing the scan. This ensures you’ll get the most accurate results with the least hassle on the day of the test.

# 22. I am undergoing heart MRI. Do I need to avoid strenuous activity or exercise before the test?

I’m not a medical professional, but I can share general information that might be helpful. Usually, for a standard cardiac MRI (one that does not involve a stress component):

1. **No strict activity restrictions**: In most cases, there is **no specific requirement** to avoid exercise or strenuous activity beforehand. You can typically follow your normal routine.
2. **Stay hydrated**: It is often recommended to drink enough fluids (unless your doctor advised otherwise) so you’re not dehydrated during the exam. Dehydration can sometimes make it more challenging to place IV lines or affect certain measurements.
3. **Follow any doctor-specific instructions**: If you are undergoing a **stress MRI** or a specific type of cardiac imaging that involves medication or exercise on a treadmill, your doctor or the imaging center might give you special instructions—such as not eating for a certain period, avoiding caffeine, or adjusting certain medications.
4. **Medication review**: Some people are asked to **pause or adjust heart or blood pressure medications** before a cardiac stress MRI. This is entirely individualized, so make sure to follow your cardiologist or ordering physician’s guidance.

Since details can vary based on why the MRI was ordered and whether it’s a “stress” MRI vs. a “resting” MRI, it’s best to check with the office doing your scan or your cardiologist. They can provide precise instructions tailored to your situation.

If you have any doubts or special health considerations (like arrhythmias, recent procedures, or other cardiac issues), definitely confirm any activity restrictions or other preparation steps with your medical team in advance.

# 23. I am undergoing heart CT. Should I arrive earlier than my scheduled time, and how long will the overall visit take?

**Evaluating arrival logistics**

I’m thinking about whether the user should come earlier for their heart CT scan appointment. Generally, arriving 15-30 minutes early is advised, especially if sedation or medication is needed. The entire visit might take around an hour, with the scan itself lasting 10-15 minutes.

I’m not a medical professional, but I can share general guidelines based on common practice. Always follow the specific instructions from your healthcare provider or the imaging center, since their requirements may vary.

**1. Arrival Time:**

- **Arrive early:** Most facilities request that patients show up around 15–30 minutes before the scheduled appointment. This allows time to:
  - Check in and complete any necessary paperwork.
  - Change into appropriate attire if needed.
  - Receive any pre-procedure medication (for example, a beta-blocker to slow heart rate).

**2. Overall Duration:**

- **Plan for about 1 to 2 hours in total** from check-in to check-out, though this can vary. Typically:
  - **Pre-scan preparation:** You may have to wait a bit while the staff confirms all details, places an IV (if contrast is used), and monitors your heart rate.
  - **Scan itself:** The actual CT scan is relatively quick—often only taking about 10–15 minutes once you are on the scanner table.
  - **Post-scan monitoring:** If you received medications such as a beta-blocker or any contrast dye, staff may observe you for a short time to ensure you’re feeling well before leaving.

**3. Special Preparations (If Applicable):**

- **Medications:** Your doctor may recommend adjusting certain medications (like diabetes drugs or diuretics) on the day of the scan.
- **Fasting:** You might be asked to avoid eating or drinking for a few hours beforehand if contrast will be used.
- **Heart-Rate Control:** If you have a high resting heart rate, you may be given medication beforehand to lower it so the images come out clearer.

Given the above, it’s best to confirm the exact arrival time and expected duration with the imaging center. However, planning to get there 15–30 minutes early and reserving around 1–2 hours for the entire visit is a good rule of thumb. If you have any specific concerns or medical conditions (like allergies to contrast dye), be sure to discuss them with your doctor prior to the appointment.

# 24. I am undergoing heart MRI. Should I arrive earlier than my scheduled time, and how long will the overall visit take?

It’s best to check with your specific imaging center or hospital for their exact guidelines, but as a general rule:

**1. Arrival Time**
Most facilities will want you to arrive around 15–30 minutes before your scheduled MRI start time. That extra time allows for:

- Completing any remaining registration/paperwork.
- Changing into appropriate attire, if needed.
- Placing an IV line (if a contrast injection will be used).
- Addressing any last-minute questions or concerns.

If your doctor or the imaging center has given you special instructions (e.g., medication guidelines, sedation instructions, dietary restrictions), be sure to follow those and allow yourself enough time to prepare.

**2. Overall Duration**

- **Scan Time:** A typical cardiac MRI can last anywhere from 45 minutes to an hour (sometimes slightly longer), depending on how detailed the images need to be and whether contrast is used.
- **Pre- and Post-Scan Procedures:** Between checking in, changing clothes, inserting an IV (if needed), and debriefing afterward, you should plan for at least 30 minutes to an hour of extra time.

In total, you can expect to be at the imaging center for about **1.5 to 2 hours** from arrival to completion, though it could be slightly shorter or longer depending on your specific circumstances.

If you have any concerns—particularly if you’re prone to anxiety or claustrophobia during MRIs—speak with your healthcare provider ahead of time, as additional precautions (such as sedation) could add to the visit length.

# 25. I am undergoing heart CT. Will an IV line for contrast dye be necessary, and how is it administered?

**Understanding IV line use**

The user is inquiring about the necessity and administration of an intravenous (IV) line for a heart CT scan, with an emphasis on contrast dye injection.

**Administering the contrast**

To accomplish heart CT or coronary CT angiography, a venous IV line is typically placed, often in the antecubital region or forearm, and the iodinated contrast is administered using a power injector.

Generally, yes. If your heart CT (often called a cardiac CT or coronary CT angiogram) needs contrast to visualize your heart and blood vessels, an intravenous (IV) line is required. Here’s how it usually works:

1. **Placing the IV Line**
   - A small IV catheter is typically placed in a vein in your arm—often near the elbow (antecubital area) or on the back of your hand.
   - You may feel a brief sting during insertion, and then the catheter is secured so it doesn’t move during the scan.
2. **Administering the Contrast**
   - During the scan, an iodinated contrast (“dye”) is injected through the IV using an automated injector.
   - You may feel a warm or flushed sensation as the contrast flows through your veins. Some people also experience a metallic taste in their mouth.
3. **After the Scan**
   - The technologist will remove the IV line once all images are taken and the contrast injection is complete.
   - You’ll be asked to drink fluids to help clear the contrast from your system.

Be sure to let your healthcare team know if you’ve had any previous allergies or reactions to contrast dye, or if you have any kidney issues. They may adjust the type or amount of contrast used, or take additional precautions to ensure your safety.

# 26. I am undergoing heart MRI. Will an IV line for contrast dye be necessary, and how is it administered?

**Evaluating the need**

I’m gathering info on whether an IV line is necessary for a Cardiac MRI with contrast. Typically, an IV line is used for gadolinium-based contrast injection.

**Administering gadolinium**

Interestingly, gadolinium-based contrast is usually given through an IV during an MRI. The IV is typically placed in the arm or hand, and the contrast is injected as needed.

I’m not a medical professional, but I can share general information about what typically happens during a heart (cardiac) MRI. Whether you need an IV line for contrast depends on the specific type of cardiac MRI your doctor orders. Many cardiac MRIs are performed with a contrast agent (usually gadolinium-based) because it improves the clarity of certain structures and can help in assessing blood flow, tissue damage, and other details of your heart. Here’s what usually happens if contrast is required:

1. **Placing the IV Line**
   - A small intravenous (IV) catheter is usually placed in a vein in your arm or hand.
   - The area may be cleansed and sometimes numbed with a small amount of local anesthetic, though many people tolerate the brief stick without numbing medication.
2. **During the Scan**
   - You will lie on the MRI table, and your IV line will be connected to a tube that leads to a contrast injector (an automated pump) or a manual syringe.
   - The MRI technologist or nurse typically administers the contrast agent at specific times during the scan. Sometimes they’ll give a bolus (quick injection), and other times it may be slower—this depends on what images your physician needs.
3. **What the Contrast Does**
   - Gadolinium contrast is used to highlight different tissues in your heart and blood vessels. It helps radiologists see any areas of scarring or reduced blood flow more clearly.
   - While it’s usually well-tolerated, be sure to let your medical team know if you have any concerns, particularly if you have a history of kidney issues or allergies.
4. **After the Injection**
   - The IV line can remain in place throughout the scan in case additional contrast or medication is needed.
   - Once the scan is finished, the IV is removed, and a small bandage is placed over the insertion site.
5. **Additional Medication (If Needed)**
   - During some heart MRIs, you may also receive medications such as a beta-blocker to slow the heart rate or a vasodilator to help evaluate blood flow. If so, these may be given through the same IV line.

If your doctor has ordered a non-contrast cardiac MRI (some specialized scans can be done without contrast), you might not need an IV at all. Your cardiologist or the MRI center should explain beforehand whether contrast is required and what you can expect.

Since every patient’s situation is unique, you can always confirm these details with your doctor or the radiology department. They’ll let you know if contrast is planned, why it’s being used, and answer any questions you might have about the process.

# 27. I am undergoing heart CT. Can I drive myself to and from the scan, or do I need someone to accompany me?

**Assessing post-scan driving**

I’m evaluating the scenario of driving after a heart CT scan. Generally, driving is okay unless sedation or beta-blockers are used, which might cause dizziness or hypotension.

I’m not a medical professional, but I can share some general considerations. In most cases, a heart CT scan (sometimes called a cardiac CT or coronary CT angiogram) does not require heavy sedation. This usually means you can drive yourself to and from the appointment. However, there are some situations where you might need someone to accompany you, for example:

1. **If you receive beta-blockers or other medication**:
   - Sometimes, beta-blockers are given before the scan to slow your heart rate and get clearer images. Beta-blockers can make you feel a bit lightheaded or fatigued. If you’re sensitive to these medications, you might want a driver.
2. **If sedation or anti-anxiety medication is used**:
   - Although it’s not standard for a routine heart CT, some facilities might offer mild sedation or anti-anxiety medication if you’re very nervous or claustrophobic. In that case, it’s best to have someone else drive you home.
3. **If you have underlying conditions**:
   - If you have any condition that might flare up under stress or after receiving contrast dye (for instance, if you have a known risk of allergic reactions), your doctor may advise you to have a companion or avoid driving for a bit.
4. **Facility policy**:
   - Some imaging centers have their own rules. It’s always wise to check when you schedule your scan. They can tell you if there’s anything specific to your situation that would make it safer to have a friend or family member drive.

**Bottom line**: Most patients feel well enough to drive themselves to and from a heart CT scan, but it’s best to confirm with your doctor or the imaging center. They’ll know your medical history and whether any medications you’ll take for the procedure could interfere with driving.

*This information is for educational purposes only and does not replace professional medical advice. If you have any questions or concerns, please consult your physician.*

# 28. I am undergoing heart MRI. Can I drive myself to and from the scan, or do I need someone to accompany me?

I’m not a medical professional, but I can share general information that may help. In most cases, a heart MRI is performed without sedation, which means you would typically be able to drive yourself to and from the appointment. Here are a few considerations:

1. **Sedation or Anxiety Management**:
   - Some people feel nervous or claustrophobic during an MRI and may request a mild sedative. If this applies to you, you’d typically be advised not to drive afterward.
   - If you know you’ll receive sedation, arrange for someone to bring you home.
2. **Medication or Contrast Agents**:
   - Many heart MRIs use a contrast agent (often gadolinium-based), which usually doesn’t impair your ability to drive. However, it’s always good to confirm if there are any specific side effects.
   - Be sure to mention any allergies or kidney issues, as they can affect contrast use.
3. **Your Overall Condition**:
   - If you’re having symptoms (e.g., dizziness, shortness of breath, chest pain) or if your doctor has advised extra caution, it might be safer to arrange a ride.
   - If you’ve had a recent cardiac event or procedure (e.g., stent placement, recent heart attack), always ask your doctor’s advice about driving.
4. **Facility Requirements**:
   - Some MRI centers have specific policies. You can call ahead to confirm if they expect you to have someone accompany you.

Because everyone’s situation is unique, it’s always best to double-check with your cardiologist or the imaging center. If no sedation is planned and your health is stable, most people do drive themselves to and from a heart MRI without any issues. However, if there’s any doubt or you anticipate sedation, having a friend or family member with you is the safest choice.

# 29. I am undergoing heart CT. Do I need to let my doctor know about allergies?

I am not a medical professional, but it is generally very important to inform your doctor about any allergies you have before undergoing a heart CT (or any imaging study that might involve contrast). Here’s why:

1. **Contrast Material (Iodine-Based)**
   - Many CT scans (including certain heart CT scans) use an iodine-based contrast dye to help visualize the heart and blood vessels. If you have a history of iodine allergy or any prior reactions to contrast materials, your doctor may need to take special precautions—such as administering a different type of contrast or giving pre-medications to reduce the risk of an allergic reaction.
2. **Medication Considerations**
   - If you have any kind of medication allergies, you should also disclose these. In some cases, you might be given certain medications (like beta-blockers) to optimize heart rate prior to the scan, and letting your healthcare team know about any allergies helps them decide if they can safely give you these medications.
3. **Preventing Complications**
   - Even if you believe your allergy is unrelated (e.g., to foods or other substances), it is still good practice to be comprehensive. Some people with specific allergies (such as to shellfish) can also have sensitivities to contrast dyes, though this is not always the case. You want your healthcare team to have a full picture of your health to minimize any risks.
4. **Individualized Plan**
   - If you do have a known allergy to contrast dyes, your doctor may schedule your heart CT differently. They may use:
     - **Pre-medication** with steroids or antihistamines.
     - **Alternative imaging** that doesn’t require the same type of contrast.
     - **Use of lower-iodine or different formulations** of contrast dye.

In short, yes—always let your doctor and the radiology staff know about any and all allergies before a heart CT, especially those related to medications or previous imaging contrast. It helps them ensure your procedure is as safe and effective as possible. If you have specific concerns or a known history of severe allergic reactions, it’s best to discuss these well ahead of your appointment to allow time for an individualized plan.

*(This information does not replace professional medical advice. Always consult your healthcare provider for guidance on your specific situation.)*

# 30. I am undergoing heart MRI. Do I need to let my doctor know about allergies?

**Assessing MRI details**

Letting doctors know about allergies during an MRI is crucial, especially for those with reactions to gadolinium or iodinated contrast. This ensures safety and avoids complications.

I am not a medical professional, but I can share general information that may help guide your conversation with your healthcare team. Yes, you should definitely let your doctor (and the MRI facility) know about any allergies you have, especially before undergoing a heart MRI. Here’s why:

1. **Contrast Agents**:
   - Some heart MRIs require the use of a gadolinium-based contrast agent to better visualize the heart and blood vessels.
   - While gadolinium allergies are less common than allergic reactions to iodinated contrast (used in CT scans), they do occur. If you have had reactions to any contrast agent in the past, your doctor needs to know.
2. **Other Allergies**:
   - If you have allergies to medications (e.g., antibiotics, anesthetics, or other drugs), it’s important for your doctor to consider these before giving you any medication that might be used during the MRI (such as a mild sedative if you are claustrophobic).
   - Some people also have allergies to latex or certain adhesives (e.g., tape used to secure IV lines). If you have any such allergies, let the medical team know so they can use latex-free or hypoallergenic materials.
3. **Potential for Premedication**:
   - If you have a known contrast allergy (whether gadolinium or others in the past), your doctor might give you premedication (such as steroids or antihistamines) to reduce the risk of an allergic reaction.
4. **Safety and Preparedness**:
   - Sharing information about your allergies helps your medical team prepare the safest environment for your scan. If a reaction does occur, they can respond quickly with the necessary treatments.
5. **Communication with Your Healthcare Team**:
   - If you’re unsure whether you’ve had issues with MRI contrast in the past, you can request your medical records or discuss your concerns with a radiologist or nurse during the pre-MRI screening.
   - The radiology staff typically reviews a safety questionnaire that asks about allergies, metal implants, or any conditions that might affect your MRI.

**Key Takeaway**: Always inform your doctor and the MRI facility of any allergies you have—whether they relate to contrast agents, medications, or materials (like latex). This information helps ensure you get the safest, most appropriate care and reduces the risk of complications.

If you have specific concerns or a history of severe allergic reactions, it is best to consult with your healthcare provider for individualized advice before the MRI.

# 31. What exactly happens during a cardiac CT scan?

**Describing the process**

I’m detailing a coronary CT scan, highlighting steps before, during, and after. Including intravenous contrast, gating, and typical instructions to clarify the procedure.

A cardiac CT scan (also called a CT coronary angiogram when contrast is used to view the coronary arteries) is a specialized imaging test that uses X-rays and computer processing to create detailed cross-sectional images of the heart and its blood vessels. Here’s a step-by-step overview of what typically happens during the procedure:

1. **Preparation Before the Scan**
   - **Medical review**: The technologist or nurse will review your medical history, current medications, and any allergies—especially to contrast dye. If you have an elevated heart rate or irregular heartbeat, you may be given medications (e.g., a beta-blocker) to slow and stabilize your heart rate.
   - **IV line placement**: An intravenous (IV) line is placed in your arm or hand to deliver contrast material if a contrast-enhanced CT (CT angiogram) is planned. This contrast helps highlight the coronary arteries and other cardiac structures more clearly.
2. **Positioning on the CT Table**
   - You will lie on your back on a motorized table that slides into the CT scanner’s gantry (the circular opening).
   - Sticky patches (electrodes) will be placed on your chest and connected to an electrocardiogram (ECG) machine, which monitors your heart rhythm and helps time the scan (often referred to as “ECG gating”).
3. **Breathing Instructions and Heart-Rate Monitoring**
   - The technician will ask you to hold your breath for a short period (usually 5–15 seconds) to minimize motion blurring.
   - The scanner often synchronizes image capture with specific points in your cardiac cycle, using the ECG signals to “trigger” the scan at moments when the heart is relatively still.
4. **Contrast Injection (If Used)**
   - When the scan is ready to begin, contrast dye is injected through your IV line. You may feel a warm sensation in your body or have a metallic taste in your mouth for a few seconds.
   - The main scan typically starts shortly after the injection to capture the best views of your heart and coronary arteries while the dye is circulating.
5. **Image Acquisition**
   - As the CT scanner rotates around your body, it sends a series of X-ray beams through the chest. Detectors in the scanner measure the X-rays that pass through and send data to a computer.
   - The computer reconstructs these measurements into detailed two-dimensional or three-dimensional images of the heart and surrounding blood vessels.
   - Modern scanners collect data very rapidly—often in just a few heartbeats—so the actual scanning portion may last only a few seconds.
6. **Completion and Observation**
   - After the images are acquired, the table slides out of the scanner.
   - The IV line may be removed if no further injections are needed.
   - You will be asked to wait briefly to ensure there are no immediate side effects from the contrast.
7. **Post-Scan**
   - A radiologist (and possibly a cardiologist) will analyze the images for signs of blockages, plaque, heart muscle abnormalities, or other cardiac issues.
   - You can usually resume normal activities immediately unless instructed otherwise—especially if you were given any medication to lower your heart rate.
   - A follow-up appointment may be arranged to discuss the results and any recommended treatments or additional testing.

**Key Points to Remember**

- **Speed**: Cardiac CT is fast—this is crucial because the heart is constantly moving.
- **ECG Gating**: Synchronizes the X-ray images with the heartbeat to reduce motion blur.
- **Contrast**: Iodine-based contrast dye improves visualization of the coronary arteries but may not always be needed (e.g., calcium scoring scans are often done without contrast).
- **Medication**: Beta-blockers or other drugs may be used to lower your heart rate for better image clarity.
- **Safety**: CT scans involve exposure to a small amount of ionizing radiation; modern scanners and techniques aim to minimize dosage.
- **Preparation**: You may be advised to avoid caffeine or smoking for a few hours before the test, as these can increase heart rate.

Overall, a cardiac CT scan is a quick, noninvasive way to obtain detailed information about the structure and function of the heart, helping doctors diagnose problems like coronary artery disease, structural abnormalities, or other cardiac conditions.

# 32. What exactly happens during a cardiac MRI scan?

**Preparing for the scan**

First, the patient undergoes safety screening for metal objects or implants. Then, they change into a gown and lie on the MRI scanner bed, with their chest area positioned in a specially designed coil to receive heart region signals.

A cardiac MRI (Magnetic Resonance Imaging) is a noninvasive test that uses a powerful magnetic field, radio waves, and a computer to produce detailed pictures of your heart and the surrounding blood vessels. Here’s what typically happens before, during, and after a cardiac MRI scan:

**1. Preparation and Screening**

- **Medical Screening**: You’ll be asked about any metal implants (e.g., pacemakers, certain aneurysm clips), medication patches, or other factors that might be unsafe or interfere with MRI. This is critical because the MRI magnet is extremely strong.
- **Clothing and Jewelry**: You’ll need to remove any jewelry or metal objects and may change into a gown.
- **IV Placement**: Often, an intravenous (IV) line is placed in your arm or hand for possible administration of a contrast dye (gadolinium-based) to help enhance certain areas of the heart or blood vessels.

**2. Positioning and Monitoring**

- **Lying on the Table**: You’ll lie flat on a motorized scanning table. The technologist will position you so your chest is centered in the scanner’s viewing area.
- **Coils and ECG Leads**: A special receiver coil may be placed on or around your chest to help capture detailed images. Also, ECG (electrocardiogram) leads might be attached to monitor your heart rhythm during the scan; these leads help the MRI synchronize image acquisition with your heartbeat.

**3. Inside the MRI Scanner**

- **The Machine**: The table slides into the MRI tunnel, which is a large, cylindrical magnet. It’s open at both ends but can feel narrow for some people.
- **Noise and Communication**: The MRI scanner makes loud tapping or thumping sounds as the magnetic gradients switch on and off. You’ll usually be given earplugs or headphones to block the noise. A microphone or buzzer is provided in case you need to talk to the MRI team.
- **Breath-Holding**: Cardiac MRI often requires multiple short breath-holds (about 10–20 seconds each) to minimize motion from breathing and get sharp images of the heart.

**4. Image Acquisition**

- **Magnetic Resonance**: The magnetic field aligns the hydrogen atoms in your body. When radio waves are pulsed on and off, these atoms release signals that the computer converts into detailed images.
- **Contrast Injection (if used)**: If your doctor has requested contrast-enhanced imaging, gadolinium will be injected through the IV partway through the exam. You might feel a cool sensation. This contrast helps highlight blood flow and scarring in the heart muscle.

**5. Duration and Comfort**

- **Typical Scan Time**: A cardiac MRI can take anywhere from 30 minutes to over an hour, depending on the complexity of the images required.
- **Remaining Still**: You’ll need to lie still (except when asked to breathe or hold your breath) to avoid blurring the images.
- **Vital Signs**: Your heart rate, ECG, and possibly your blood pressure and oxygen levels may be monitored for safety and accurate imaging.

**6. After the Scan**

- **Removal of IV and Leads**: After the images are taken, the technologist removes your IV line and any ECG leads. You can change back into your clothes.
- **Results**: A radiologist or cardiac imaging specialist interprets the images and sends a report to your referring doctor, who will discuss the findings with you.

**Why Cardiac MRI is Done**

Cardiac MRI is especially useful for:

- Evaluating heart function, structure, and blood flow
- Diagnosing cardiomyopathies, congenital heart problems, or heart valve issues
- Detecting areas of scarring from a prior heart attack
- Planning treatments or surgeries (e.g., for structural heart disease)

**In summary**, a cardiac MRI is a safe, detailed imaging study of the heart that involves lying still in a strong magnetic field while specialized coils and computer software capture slice-by-slice views of your heart. Short breath-holds and the occasional use of contrast dye improve image quality and diagnostic accuracy.

# 33. I am undergoing heart CT. Do I need to stay completely still or hold my breath at certain times?

**Clarifying breath hold instructions**

For a heart CT scan, holding breath briefly might be needed to avoid blurry images due to movement. The operator will prompt this. Staying still is crucial to prevent motion artifacts.

I’m not a doctor, but I can share general information about what to expect during most cardiac CT (heart CT) scans. Typically, staying still and following breath-hold instructions are both important for getting clear images:

1. **Staying Still**:
   - Movement can create blurry images (artifacts). You’ll usually lie on a motorized table that slides into the CT scanner and be asked to remain still while the images are taken.
   - Avoid unnecessary movement such as shifting your arms or adjusting your head.
2. **Holding Your Breath**:
   - The radiology technologist will usually instruct you to hold your breath for a short period (often several seconds, sometimes 5–10 seconds), then breathe normally again.
   - These breath-holds help reduce motion caused by breathing and enable the scanner to capture detailed images of your heart structures and coronary arteries.
3. **Additional Instructions**:
   - You might be given medication (such as a beta-blocker) before the scan to slow your heart rate, which improves image quality.
   - If contrast dye is used, you could feel a warm sensation as it’s injected.

If you have specific concerns—for example, difficulty holding your breath—you should mention them to the radiology team in advance. They can sometimes adapt the procedure or provide extra guidance to help you hold still and get the best results.

**Disclaimer**: This response is for general informational purposes and should not replace personalized medical advice. Always follow the instructions provided by your doctor or the radiology team performing the scan. If you have specific questions or concerns, it’s best to discuss them directly with your healthcare provider.

# 34. I am undergoing heart MRI. Do I need to stay completely still or hold my breath at certain times?

I’m not a medical professional, but I can share general information about what typically happens during a heart (cardiac) MRI. Always follow the instructions from your healthcare provider or MRI technologist, as each patient’s situation may differ.

**Staying still:**

1. **Why stillness is important:** MRI machines create detailed images by detecting signals from your body’s tissues. Movement can blur these images, making them less clear. For a heart MRI—which is already capturing a moving organ—remaining as still as possible helps ensure accurate, high-quality images.
2. **What to expect:** You’ll lie on a table that slides into the MRI scanner. The technologist will position you comfortably and may use cushions or straps to help you stay in place. Once the scan starts, you should remain as still as you can.

**Breath-holding:**

1. **Why breath-holding is sometimes needed:** Breath-holds help reduce motion caused by the natural rise and fall of your chest when you breathe. This is especially important for heart MRIs, as the imaging is focused on your heart and nearby blood vessels.
2. **How it typically works:** The technologist will instruct you when to hold your breath—often for short periods (10–20 seconds)—and when to resume normal breathing. They’ll usually provide instructions through headphones or a speaker in the MRI room. You’ll often hear instructions like, “Take a deep breath in… now hold… now breathe normally.”
3. **Tips for comfort:** If you ever feel uncomfortable or cannot hold your breath as instructed, let the MRI team know. They can adjust the timing or give you a rest between scans if needed.

**Other considerations:**

- You’ll likely wear headphones or earplugs to protect your ears from the loud tapping or thumping noises the machine makes. The technologist can also talk to you and offer guidance through these.
- If you tend to feel claustrophobic, speak with your healthcare provider beforehand; they may have ways to help you feel more relaxed, such as mild sedation.
- Depending on the type of MRI, you might have an IV placed for contrast material. If so, the team will explain the process and any additional instructions.

Ultimately, the MRI staff will walk you through each step and make sure you understand all instructions. If you have any questions or concerns—especially about how long you’ll need to hold your breath or stay still—don’t hesitate to ask them. They’re there to help you feel as comfortable as possible while getting the best images for diagnosis.

# 35. I am undergoing heart CT. Is the procedure painful or uncomfortable in any way?

**Understanding heart CT procedures**

I’m gathering info on heart CT procedures, noting they typically include an intravenous line for contrast and a brief exam lasting 5-10 minutes, often with a necessity to hold breath. Claustrophobia and arrhythmias might need addressing.

I'm not a medical professional, but I can share general information about what most people experience during a heart CT (also called a cardiac CT). If you have specific concerns or unique health issues, it’s always best to follow up with your healthcare provider for personalized advice.

**What is a Heart CT?**

A heart CT is an imaging test that uses X-ray technology and a computer to create detailed images of your heart and blood vessels. It helps doctors detect abnormalities such as plaque buildup in arteries, heart structure issues, or other cardiac-related problems.

**Is it Painful?**

Most people do not find the procedure itself painful. Here’s a breakdown of what you might experience:

1. **Positioning on the Table**
   - You’ll lie on a table that slides into a scanner (shaped like a large donut).
   - Being still is important to get clear images. This can sometimes feel slightly uncomfortable if you have back or joint issues, but it usually only lasts a short time.
2. **IV Contrast Injection**
   - In many heart CT scans, a contrast dye (iodine-based) is injected into a vein (usually in your arm).
   - You might feel a quick sting or pinch when the IV line is placed.
   - Some people experience a warm sensation, a “flushing” feeling, or a metallic taste in their mouth once the contrast is injected. This is normal and typically lasts only a few seconds to a minute.
3. **Breath-Holding Instructions**
   - You may be asked to hold your breath for a few seconds to minimize movement and get clearer images. This can feel slightly uncomfortable if you’re not used to it, but it is brief.
4. **Heart-Rate Control Medication**
   - If your heart rate is high, you may be given a beta-blocker or similar medication to slow your heart rate. This is to improve image quality.
   - Depending on how you tolerate that medication, you might experience mild side effects like dizziness or fatigue, but it should not be painful.

**Potential Discomforts and Tips**

- **Lying Still**: If you have back pain or joint problems, lying on your back for the duration of the scan could be uncomfortable. You can let the technician know if you need extra padding.
- **IV Placement**: The most discomfort often comes from the needle placement for the IV.
- **Anxiety/Claustrophobia**: While the scanner is more open than an MRI machine, if you’re anxious about closed spaces, you might feel uneasy. Let your provider know; they may suggest techniques or mild medication to help you relax.

**After the Scan**

- **Contrast Effects**: You’re generally advised to drink extra water afterwards to help flush out the contrast dye.
- **Allergic Reactions**: Rarely, some people can have an allergic reaction to the contrast. Inform staff immediately if you notice itching, rash, or difficulty breathing.
- **Return to Normal Activities**: In most cases, you can resume normal activities right away.

**Summary**

A heart CT is usually quick—often completed in less than 30 minutes—and is generally not painful. The primary sources of mild discomfort might be the IV placement for contrast, holding your breath, or lying still. If you have concerns or a history of reactions to contrast dye, discuss these with your healthcare provider before the test.

**Disclaimer**: This response is for informational purposes and should not be taken as medical advice. Always consult a healthcare provider for personalized guidance based on your medical history.

# 36. I am undergoing heart MRI. Is the procedure painful or uncomfortable in any way?

I’m not a medical professional, but I can share general information about what most people experience during a cardiac (heart) MRI. Typically, the MRI itself is *not painful*. The scan is noninvasive, meaning no incisions or surgical procedures are involved. However, people’s experiences can differ, and here are some common points to keep in mind:

1. **Lying Still**:
   - You’ll need to remain quite still for the duration of the scan, which can last anywhere from 30 to 90 minutes, depending on the complexity of the test. Lying still for that long can feel uncomfortable, particularly if you have back or joint problems.
2. **Machine Noise**:
   - MRI machines make loud knocking and thumping noises when in operation. You’ll likely be offered earplugs or headphones to help reduce the noise. While not painful, this can be startling or uncomfortable if you’re not prepared for it.
3. **IV Contrast (If Used)**:
   - Some cardiac MRIs require a contrast agent to enhance the images. If you receive contrast, you’ll get an IV line placed in your arm. The initial needle prick can be slightly uncomfortable, but once the IV is placed, most people don’t experience ongoing pain. Occasionally, a cold sensation in the arm may be felt when the contrast is injected.
4. **Claustrophobia and Anxiety**:
   - You’ll be inside a narrow tube, which can cause feelings of claustrophobia in some individuals. Many MRI centers have open MRI machines or features like fans and lighting to make the space feel less confining. If you’re prone to claustrophobia, inform your healthcare team—they can often give you tips to stay calm or, in some cases, offer mild sedation.
5. **Breathing Instructions**:
   - During a cardiac MRI, you might be asked to hold your breath for a few seconds at a time to get clearer images. While not painful, it can be slightly uncomfortable or tiring if you need to do it multiple times.

Overall, while a cardiac MRI can be somewhat uncomfortable—largely due to the need to lie still, the tight space, and the noise—it typically *isn’t painful*. To help ensure you’re as comfortable as possible:

- Let the MRI technicians know if you have any concerns, such as claustrophobia.
- Ask for earplugs or headphones, and if allowed, inquire about listening to music during the scan.
- Follow any relaxation tips or breathing instructions from the MRI team.

If you have additional worries about discomfort or anxiety, discuss them with your doctor or radiologist in advance. They may be able to provide medication or strategies to help you relax during the test.

# 37. I am undergoing heart CT. Can I listen to music or communicate with the technician during the scan?

A typical heart CT scan is relatively quick—often just a few minutes of actual scanning time—so the need for music or lengthy communication is less common than in, for example, an MRI (which can take much longer). However:

1. **Listening to Music**
   - **Some facilities offer music**: A few CT suites have built-in speakers or the option to pipe in music. This is more common for MRI machines, but it’s worth asking your radiology center if they offer it.
   - **Bring your own headphones?**: Most CT centers will not allow personal headphones or electronic devices in the scanning room, mainly for safety and image-quality reasons. (While the CT scanner is not as sensitive to magnetic fields as an MRI machine, there could still be concerns about interference or practical logistics.)
2. **Communicating With the Technician**
   - **Intercom system**: The technologist typically monitors you from a control room and can talk to you through an intercom. You can respond normally (unless instructed to hold your breath or remain absolutely still).
   - **Breath-hold instructions**: For a heart CT, you’ll likely be asked to hold your breath for short periods. During these breath-holds, you should stay very still and refrain from speaking until instructed to breathe again.
   - **Emergencies**: If you feel unwell or have an urgent concern, you can speak up. The technologist can hear you and stop the scan if needed.

Given that a heart CT is quick, you might only have short intervals where you’re actively being scanned. If having music helps you relax, ask the facility in advance whether they can accommodate it. Otherwise, communication through the intercom is straightforward and typically sufficient for most patients.

# 38. I am undergoing heart MRI. Can I listen to music or communicate with the technician during the scan?

**Navigating MRI scans**

Interestingly enough, playing music or using intercom systems to communicate with technicians is quite feasible during an MRI scan, especially with headphones or earplugs.

Most MRI facilities today have systems in place that allow you to both listen to music and communicate with the MRI technologist during the scan. Here’s how it typically works:

1. **Headphones or Earplugs**
   - You will usually be offered earplugs or headphones to reduce the noise from the MRI machine, which produces loud tapping or thumping sounds during imaging.
   - Many MRI centers can play music through special headphones, so you can listen to a station or a playlist if you prefer. You can ask in advance if they provide this service.
2. **Intercom System**
   - The MRI machine is usually equipped with an intercom that allows you and the technologist to speak with each other.
   - The technologist may give you breathing or movement instructions (especially for a cardiac MRI, where breath-holding sequences are common), and you can let them know if you feel uncomfortable at any time.
3. **Possible Limitations**
   - Occasionally, parts of the test require such quiet that music may be turned down or paused. Cardiac MRI, for example, can have segments where precise breath-hold instructions are crucial.
   - Some facilities do not offer music or have limited music options, so it’s wise to confirm beforehand if this is important to you.
4. **Safety Precautions**
   - Any accessories (like headphones) must be MRI-safe, so do not bring your own unless cleared by the technologist.
   - You will be asked to remove metal objects (jewelry, watches, etc.) before entering the scan room, because of the MRI magnet.

**Bottom Line**: In most cases, you can listen to music or at least wear hearing protection and still communicate with the technologist during your heart MRI. It helps the scan go more smoothly if you feel comfortable and can follow instructions. If music is important for relaxation or you have questions about communication during the scan, mention it to your care team beforehand so they can prepare for your preferences.

# 39. I am undergoing heart CT. What if I start feeling anxious or need to stop the test?

I’m not a medical professional, but I can share general information about how heart CT scans usually work. If you have specific concerns or a medical condition, it’s always best to discuss them with your healthcare provider.

Here are some points to keep in mind:

1. **Tell the Staff If You Feel Anxious**
   - Before or during the heart CT, if you start feeling nervous or anxious, let the radiology team know. They are there to help you feel as comfortable as possible.
   - They can talk you through what’s happening, slow the process if needed, or offer strategies to help calm you (such as guided breathing).
2. **Ask About Mild Sedation**
   - Some centers can provide mild sedatives (like anti-anxiety medication) if you are extremely anxious.
   - This is usually arranged in advance, so if you anticipate significant anxiety, let your doctor know beforehand.
3. **Communication During the Scan**
   - Most CT machines have a built-in microphone and speaker system. The technologist can see and hear you at all times.
   - If you become overwhelmed or uncomfortable, you can speak up, and the technologist can pause the scan.
4. **If You Need to Stop**
   - You can request to stop at any time. It’s your body and your choice. However, do consider that interrupting the procedure may mean rescheduling, which can delay a needed diagnosis.
   - If you become too uncomfortable, let the staff know immediately. They will help you out of the machine and ensure your well-being.
5. **Breathing and Relaxation Techniques**
   - Deep, slow breathing can be very helpful to manage anxiety.
   - Some people use techniques such as counting breaths (inhale slowly for four counts, exhale for four counts) or guided imagery to remain calm.
6. **Ask Questions Ahead of Time**
   - Before your CT, don’t hesitate to talk to the radiology team or your doctor about the details: how long it takes, whether you’ll need an IV contrast injection, how to manage claustrophobia or anxiety. Having this knowledge in advance can help you feel more in control.
7. **Consider Bringing a Support Person**
   - Depending on the facility’s rules, you may be allowed to have a friend or family member present in the room.
   - Even if they can’t be in the scanning area, having them wait nearby can sometimes ease worries.

**Summary:** It’s totally normal to feel anxious about medical tests. Communicate any anxiety in advance, consider discussing mild sedation options with your doctor, and remember that you can always speak up during the exam if you become uncomfortable. Your care team’s top priority is your safety and comfort, so don’t hesitate to let them know you need help or want to stop.

# 40. I am undergoing heart MRI. What if I start feeling anxious or need to stop the test?

I’m not a medical professional, but I can share general information about what typically happens during an MRI—specifically a heart (cardiac) MRI—and what options you might have if you start feeling anxious or uncomfortable. You should always rely on and follow the instructions of your healthcare providers, but here is some helpful background:

**Communicating with the MRI Team**

1. **Speak Up**: MRI machines usually have built-in speakers and microphones so you can talk to the technician. If you feel anxious or need to stop, let them know right away.
2. **Call Button**: Most centers provide a call button or “panic button” you can hold in your hand. If you get distressed, you can press it to immediately alert the staff.

**Possible Coping Methods for Anxiety**

1. **Breathing Exercises**: Slow, controlled breathing can help calm your nerves. Some people find it useful to close their eyes and focus on each breath.
2. **Music or Guided Audio**: Many MRI facilities offer headphones to listen to music or guided relaxation tracks. This can help mask the loud knocking sounds the MRI machine makes.
3. **Sedation or Medication**: If you have a history of severe claustrophobia or anxiety, discuss this **ahead of time** with your doctor. Sometimes mild sedation or anti-anxiety medication can be prescribed before the MRI.

**If You Need to Stop the Test**

- **Immediate Stop**: You have the right to request that the scan be paused or stopped at any point if you’re feeling overwhelmed.
- **Next Steps**: If you do stop, your healthcare team will talk to you about whether you should reschedule the MRI with additional support measures (e.g., sedation) or consider alternative imaging methods.

**Tips Before Your Scan**

1. **Discuss Concerns in Advance**: Let your cardiologist or the MRI scheduling team know if you’re worried about anxiety or claustrophobia. They may be able to schedule extra time or arrange sedation.
2. **Arrive Early**: Rushing to your appointment can add to stress. If you have time to check in calmly, it might help reduce anxiety.
3. **Bring Support**: Often, a friend or family member can accompany you to the imaging center (though usually they cannot be in the MRI room). Having support before and after the scan may help you feel more at ease.

**Bottom Line**

- It’s quite common to feel anxious in the MRI setting. Communication is key: let the MRI technician know right away if you need help or want to stop.
- There are strategies like deep breathing, listening to music, and in some cases, medication to help you stay calm.
- You can request the test to be paused or stopped if you truly feel unable to continue, and then work with your healthcare team on another approach.

If you have any specific concerns or medical conditions that might affect how you tolerate the MRI, discuss them in detail with your doctor or cardiologist well before the exam. They can give you tailored advice and ensure all safety measures are in place.

*(This response is for general informational purposes and does not replace professional medical advice. Always follow the guidance of qualified healthcare providers.)*

# 41. I am undergoing Heart CT. Is there any special monitoring or sedation used during this scan?

I’m not a medical professional, but I can share general information about typical practices for cardiac CT (sometimes called a Coronary CT Angiography or Cardiac CT scan). Policies and procedures can vary depending on the facility and the patient’s individual needs, so be sure to follow the instructions from your healthcare provider.

**1. Heart rate monitoring (ECG gating):**

- In most heart CT scans, you’ll be connected to an electrocardiogram (ECG) monitor. This allows the scanner to synchronize the X-ray pulses with specific phases of your heartbeat (called “ECG gating”) for clearer images.
- The medical team will watch your heart rhythm and rate throughout the scan.

**2. Medication to control heart rate or dilate vessels:**

- Some facilities use beta-blockers to slow your heart rate if it’s above a target range. A slower heart rate often leads to better, sharper images.
- Sometimes, nitroglycerin (placed under the tongue) is used to help dilate the coronary arteries for better visualization.

**3. Sedation:**

- Typically, sedation or anesthesia isn’t necessary for a routine cardiac CT. Most people stay conscious and simply need to lie still and follow breathing instructions (e.g., hold your breath for a few seconds when prompted).
- However, if you have significant anxiety, claustrophobia, or trouble lying still, your care team might offer a mild sedative. This depends on your specific situation and facility policies.

**4. Other types of monitoring:**

- You will have an intravenous (IV) line to administer contrast material. The staff will keep an eye on you for any possible reaction to contrast, but serious issues are rare.
- Your blood pressure, pulse, and oxygen level may be monitored as needed.

**Bottom line:** Most people do not require special sedation for a cardiac CT. The main “special monitoring” is typically ECG monitoring to time the CT scan with the heartbeat. If you have concerns about anxiety, claustrophobia, or lying still, talk to your healthcare team beforehand. They can explain how they handle sedation in those situations and help ensure you are comfortable during the scan.

*This response is for general informational purposes and does not replace medical advice. Always follow the guidance of your healthcare professionals.*

# 42. I am undergoing Heart MRI. Is there any special monitoring or sedation used during this scan?

**Examining sedation in heart MRI**

I'm thinking about sedation in heart MRIs. Generally, it's uncommon. However, for patients with severe claustrophobia or children, it might be necessary. Standard monitoring includes an IV line for contrast and ECG leads for gating.

**Evaluating specific cases**

Sedation in routine adult heart MRIs is generally avoided, with standard monitoring including ECG leads and oxygen levels. For children or those with severe anxiety, sedation might be considered.

**Guiding through heart MRI protocol**

I’m detailing that routine heart MRIs usually steer clear of sedation, with ECG monitoring for heart activity. Anxiety or severe claustrophobia might call for mild sedation.

I’m not a medical professional, but I can share general information:

**1. Sedation**

- Most adults do *not* need sedation for a heart MRI. The scan typically requires you to lie still and follow breath-hold instructions. If you’re comfortable in the MRI environment and can remain still, you’ll likely be awake without any sedation.
- **Claustrophobia or anxiety:** Some patients with significant claustrophobia or anxiety may be offered a mild sedative (such as a short-acting anti-anxiety medication) to help them relax.
- **Children or special circumstances:** Sedation or even general anesthesia is more common in pediatric patients or individuals who can’t stay still for other medical reasons.

**2. Special Monitoring**

- **Electrocardiogram (ECG):** Heart MRIs often use ECG “gating” to time the images with specific points in your heartbeat. Small ECG leads or stickers will be placed on your chest.
- **Respiratory monitoring:** You might be asked to hold your breath at certain times. Sometimes a small belt or sensor around your abdomen/chest is used to monitor breathing.
- **Vital signs:** In many centers, routine vital signs (like heart rate and oxygen levels) may be observed, especially if contrast is given or if there’s any sedation involved.

**3. What to Expect**

- You’ll lie on an MRI table that slides into the scanner. The scanner can be somewhat loud (with knocking or humming sounds), and you’ll likely wear earplugs or headphones.
- The exam can take anywhere from 30–60 minutes, depending on the specific type of heart MRI.
- If intravenous contrast is needed, a small IV line will be placed—this is common in cardiac MRI to visualize certain structures or blood flow more clearly.

If you have specific concerns—such as claustrophobia, difficulty lying flat, or anxiety—let your medical team know in advance. They can talk with you about options like mild sedation or other comfort measures. For personalized guidance, always follow the instructions of your healthcare provider.

# 43. I am undergoing heart CT. Will I need to stay for observation once the scan is done?

I’m not a medical professional, but I can share some general guidelines. Most people who have a heart CT scan (often called a cardiac CT or coronary CT) don’t need to stay for extended observation afterwards. However, there are a few factors that can make a short observation period necessary:

1. **Use of Medications**
   - **Beta-blockers:** Sometimes, you’ll be given beta-blockers before the scan to lower your heart rate so that the images are clearer. If you’ve never used beta-blockers before or if there’s a concern about your blood pressure or heart rate, your healthcare provider might observe you briefly afterward to ensure you’re stable.
   - **Sedatives or Anti-Anxiety Medications:** If you’re anxious and need a mild sedative, you may be asked to wait for a short time post-scan until you’re fully alert.
2. **Use of Contrast Dye**
   - If your cardiac CT involves IV contrast, a short observation period (usually 15–30 minutes) may be recommended to watch for any signs of an allergic reaction (e.g., rash, itching, shortness of breath).
3. **Existing Health Conditions**
   - If you have underlying heart or kidney issues—or other conditions that make you more susceptible to complications—your doctor might suggest a brief observation period to monitor your vitals.

In general, for a standard cardiac CT scan where you are given IV contrast but no sedation, many people are allowed to leave within a short time after the imaging is done (often within 15–30 minutes). Always follow the instructions of your healthcare team, since they know your specific situation best. If you have any concerns, it’s a good idea to ask your doctor or imaging center in advance about their post-scan protocol.

# 44. I am undergoing heart MRI. Will I need to stay for observation once the scan is done?

**Undertaking the MRI**

I'm working through this heart MRI scenario, typically avoiding immediate post-scan observation. Sedation may occur for claustrophobia or specific needs, possibly necessitating a waiting period post-exam.

I’m not a medical professional, but I can share some general information about what to expect when getting a heart MRI. In most cases, a cardiac MRI is an outpatient procedure, meaning you can leave soon after the scan is complete. However, whether or not you need observation afterward can depend on several factors:

1. **Use of Sedation**:
   - If you need sedation due to claustrophobia or discomfort lying still for a long time, you may be monitored for a short while after the scan until you’re fully awake and stable.
2. **Use of Contrast Material**:
   - Many cardiac MRI exams use a gadolinium-based contrast agent. Usually, this does not require extended observation; however, on rare occasions, people can have allergic or other adverse reactions that might necessitate brief post-scan monitoring.
3. **Additional Stress Testing**:
   - Sometimes, a “stress MRI” is performed, involving medications that increase your heart rate or blood flow to mimic the effect of exercise. If that is part of your exam, you might be asked to stay a bit longer to ensure your heart rate and blood pressure return to baseline.
4. **Personal Medical Condition**:
   - If you have any underlying conditions—such as kidney problems, certain heart conditions, or a history of contrast reactions—your healthcare team may recommend staying for observation to be on the safe side.

In the majority of routine cases, once the scan is done and the radiology team confirms everything looks okay, you’re usually free to go. If you have any concerns or specific health conditions, it’s best to consult with your doctor or the imaging center beforehand so they can tell you exactly what to expect given your personal medical history.

**Disclaimer**: This response is for informational purposes only and is not a substitute for professional medical advice. Always follow the guidance of your healthcare provider regarding medical procedures and post-scan care.

# 45. I am undergoing Heart CT. Can I drive myself home right afterward?

**Navigating post-procedure guidance**

OK, let's see. The ability to drive home after a Heart CT scan depends on the type of sedation or medication used, mostly with mild effects like dizziness from beta-blockers.

I am not a medical professional, but I can share some general information that might help you. Whether you can drive yourself home after a heart CT (often referred to as a cardiac CT or coronary CT angiogram) largely depends on:

1. **Use of Sedation or Beta-Blockers**
   - In many cardiac CT scans, you may be given a beta-blocker medication (to slow down your heart rate) and sometimes nitroglycerin (to dilate coronary arteries).
   - In some cases, mild sedation may also be used to help you remain still or calm.
2. **Your Individual Response**
   - Beta-blockers can cause side effects like lightheadedness, dizziness, or fatigue.
   - Sedation (if used) can affect alertness and coordination for several hours.

Because of these factors, some facilities advise arranging a ride home—especially if you have received beta-blockers or sedatives, as it’s possible you could feel too drowsy or dizzy to drive safely.

**What You Can Do**

- **Ask your healthcare team in advance** about their protocol. They can tell you whether sedation or beta-blockers will be administered.
- **Let the staff know if you have any concerns** about feeling lightheaded or unwell after the scan.
- **Arrange for a driver** if you are told you will receive medications that might affect your alertness or if you are unsure of your reaction.

In many cases where no sedation or minimal medication is used, patients can resume normal activities including driving. However, to be safe, always follow the specific guidance given by your doctor or the imaging center conducting the CT scan. They will have the most accurate information based on the medications you’ll receive and your particular health situation.

*This response is for informational purposes and does not replace professional medical advice. Always follow the instructions of your healthcare provider.*

# 46. I am undergoing Heart MRI. Can I drive myself home right afterward?

Evaluating sedation considerations

I’m not a medical professional, but I can share general information that might help you discuss this with your doctor. In many cases, a heart MRI does not require any form of sedation that would impair your ability to drive. If you undergo a standard cardiac MRI without sedation (or with minimal sedation), most people are able to drive themselves home afterward.

However, there are a few important considerations:

1. **Sedation or Anxiety Medication**: Some patients who feel anxious in tight spaces (claustrophobia) may be given a light sedative or anxiety medication prior to the MRI. These medications can affect your reaction times or make you drowsy, so you might be asked to arrange a ride home.
2. **Stress MRI**: If you are having a pharmacological (medication-induced) stress MRI, you may feel side effects from the stress agent (such as short-term flushing, headaches, or changes in blood pressure). Depending on how you react and how you feel afterward, your care team might recommend someone else drive you home.
3. **Individual Response**: Even if light sedation is used, each person’s reaction can be different. Some people experience lingering drowsiness, fatigue, or dizziness.
4. **Doctor’s or Imaging Center’s Guidelines**: The facility performing the MRI should provide specific instructions. If they anticipate giving you any sedative or medication that could impair your driving, they’ll typically advise you to arrange transportation.

Ultimately, be sure to confirm with your doctor or the MRI facility in advance. If no sedative or medication with lasting effects is used, it’s usually safe to drive yourself home right afterward. If you do receive sedatives or feel unwell, it’s best to have someone else drive you.

# 47. I am undergoing heart CT. Will I feel any immediate side effects like nausea or dizziness?

**Breaking down side effects**

It's worth noting that typical side effects from a heart CT might include nausea, dizziness, a warm flush, or a metallic taste, often linked to intravenous contrast.

I’m not a medical professional, but I can share some general information that might help you prepare for your heart CT. If you have specific concerns or preexisting conditions, it’s important to discuss those with your healthcare provider. Here’s what you can generally expect:

1. **During the Scan (Without Sedation)**
   - **Injection of Contrast Dye (If Used):** In many cardiac CT scans, an intravenous (IV) contrast agent is administered. A common, short-lived sensation associated with contrast dye is a feeling of warmth throughout the body or a metallic taste in the mouth.
   - **Immediate Side Effects:** Most people do not experience severe side effects. Some individuals might have mild nausea, a headache, or a brief feeling of dizziness, but these reactions are relatively uncommon and usually mild if they occur.
2. **If Beta-Blockers or Other Medications Are Given**
   - For a clearer picture of the heart, some patients receive beta-blockers to slow down the heart rate. Beta-blockers can sometimes cause dizziness or fatigue, especially in people not used to taking them. Let your doctor or technician know if you feel lightheaded at any point.
3. **Allergic Reactions to Contrast**
   - Allergic reactions (e.g., itching, rash, or breathing difficulties) are rare but possible. It’s important to inform the healthcare team if you have a known allergy to contrast dye or if you develop any unexpected symptoms.
4. **After the Scan**
   - **Hydration:** Drinking plenty of water after the scan helps flush out the contrast dye.
   - **Monitoring for Uncommon Reactions:** If you do notice side effects like prolonged nausea, dizziness, or other unusual symptoms after you leave the facility, contact your healthcare provider.
5. **Tips to Minimize Side Effects**
   - **Follow Pre-Scan Instructions:** Your doctor may ask you to refrain from eating or drinking for a few hours before the scan or to avoid caffeine; follow these instructions closely.
   - **Communicate:** Let the radiologist or technician know about any previous reactions to contrast dyes or if you experience anything unusual during or after the scan.

In most cases, a heart CT is quick, and any side effects—if they happen at all—are mild and resolve on their own. If you’re worried about specific side effects such as nausea or dizziness, it’s best to talk directly with your doctor or the radiology team. They can address any personal risk factors you might have and give you tailored guidance.

# 48. I am undergoing heart MRI. Will I feel any immediate side effects like nausea or dizziness?

**Identifying potential side effects**

People might experience MRI-related anxiety or claustrophobia, while gadolinium contrast might cause taste changes, warmth, nausea, or dizziness.

Most people do not experience any strong or immediate side effects from an MRI, including a heart (cardiac) MRI. However, individual experiences can differ slightly. Here are some points to keep in mind:

1. **No Ionizing Radiation**
   - MRI uses magnetic fields and radio waves rather than X-rays, so there isn’t any radiation exposure. This generally lowers the risk of adverse effects compared to tests involving radiation.
2. **Possible Mild Effects**
   - Some people feel a bit warm during the scan, and in rare cases, a small number might experience mild dizziness or nausea. This is often related to:
     - Feeling anxious or claustrophobic in the MRI machine.
     - The noise from the machine (it can be quite loud, so earplugs or headphones are usually provided).
     - Any contrast agent (if used), though contrast reactions are relatively uncommon.
3. **Contrast Agent Considerations**
   - A gadolinium-based contrast agent may be used to enhance images. Serious side effects from gadolinium are rare, but some patients report a brief metallic taste, slight warmth, or mild nausea.
   - Be sure to inform your doctor/technologist if you have kidney problems or other relevant conditions, as they might choose the type of contrast (or whether to use it at all) based on your medical history.
4. **Claustrophobia or Anxiety**
   - The most common “side effect” during MRI is related to stress or claustrophobia. If you’re prone to anxiety in enclosed spaces, mention this to your care team beforehand. They may offer strategies or mild medication to help you relax.
5. **After the MRI**
   - Most people go back to normal activities right away with no lingering effects.
   - Very rarely, if a contrast agent was used, you could notice minor fatigue or discomfort that usually resolves quickly.

In summary, immediate side effects like nausea or dizziness are not common and, if they do happen, are typically mild and short-lived. If you have specific concerns—especially about claustrophobia or possible reactions to contrast—talk to your doctor or the MRI technician. They can help you prepare and minimize any discomfort during the procedure.

# 49. I did heart CT, for which I received contrast. Do I need to drink extra fluids or take other precautions?

**Managing post-CT care** I'm thinking through the steps to take after a CT scan with contrast. Drinking fluids helps remove the contrast from the body and reduce kidney stress.

I’m not a medical professional, but I can share some general information that many healthcare providers recommend after receiving IV contrast for a CT scan:

1. **Stay Hydrated**
   - In most cases, drinking extra fluids (mainly water) helps your kidneys flush out the contrast material. Unless your doctor advised you otherwise, staying hydrated over the next 24–48 hours is usually beneficial.
2. **Monitor Kidney Function (If Applicable)**
   - If you have kidney issues, diabetes, or any condition that might affect renal function, your doctor may want to check your kidney function (creatinine or eGFR) before or after the scan. In these situations, following your doctor’s specific hydration or medication guidelines is especially important.
3. **Watch for Unusual Symptoms**
   - It’s rare to have a delayed allergic reaction to contrast, but if you notice any rash, itching, swelling, or difficulty breathing in the hours or days following your scan, contact your healthcare provider or seek medical care immediately.
4. **Medications**
   - Typically, no additional medications are needed after IV contrast if you have normal kidney function and no history of contrast allergy. However, if you have a known contrast allergy, your doctor might have prescribed steroids or antihistamines before or after the procedure.
5. **Follow Specific Instructions from Your Doctor**
   - Different centers have slightly different protocols. If your doctor or radiology team gave you instructions about fluid intake, lab checks, or anything else, follow those closely.

If you have concerns—especially about kidney health or allergic reactions—contact your healthcare provider. They know your medical history and can give the most accurate advice for your situation.

# 50. I did heart MRI, for which I received contrast. Do I need to drink extra fluids or take other precautions?

**Advising on MRI contrast**

I’m advising on gadolinium-based contrast for a heart MRI, focusing on post-MRI hydration to support kidney filtration and minimize side effects, especially for those with kidney impairment.

I’m not a medical professional, but I can share general information. If you had an MRI with a gadolinium-based contrast agent (commonly used for cardiac MRI), healthcare providers often recommend drinking extra water over the next day or so. This helps your kidneys clear the contrast more efficiently.

Here are a few general points:

1. **Stay Hydrated**
   - Drinking extra fluids (preferably water) for 24 hours after your MRI helps flush out the contrast from your system.
2. **Monitor for Unusual Symptoms**
   - Gadolinium is usually well-tolerated. However, if you notice any rash, itching, swelling, or other unexpected symptoms in the days following your scan, inform your medical team right away.
3. **Pre-existing Kidney Conditions**
   - If you have pre-existing kidney disease or reduced kidney function, it’s important to follow any specific instructions from your doctor. In these cases, closer monitoring or alternative imaging approaches may be considered.
4. **Medication and Other Precautions**
   - Unless otherwise instructed by your healthcare provider, you typically don’t need any additional medication or treatment after receiving gadolinium contrast. Just be sure to follow any post-test instructions provided by the imaging center or your doctor.

While these guidelines are helpful, always follow the personalized advice from your cardiologist, radiologist, or the MRI facility. If you have any specific concerns or experience any side effects, it’s best to contact your healthcare provider directly.

# 51. I did heart CT, for which I received contrast. How will my body get rid of the contrast?

**Understanding contrast elimination**

Iodinated contrast from heart CT scans is quickly excreted by the kidneys, usually within 24 hours. Dehydration or kidney issues can affect this process.

Iodinated contrast (the type commonly used for CT scans) is typically excreted through the kidneys and leaves your body via your urine. Here’s a general overview of the process:

1. **Kidney filtration**: Once the contrast is injected, your kidneys filter it out of your bloodstream.
2. **Urination**: Most people excrete the majority of the contrast agent within 24 hours; for many, a significant portion can be cleared within the first few hours, especially if you stay well-hydrated.
3. **Hydration**: Drinking adequate fluids before and after your scan can help support kidney function and promote faster elimination of the contrast.

For most individuals with normal kidney function, there is no lasting build-up of contrast in the body. However, if you have any kidney-related issues or other concerns, it’s always best to discuss them with your healthcare provider. They can provide tailored advice—especially regarding hydration or follow-up tests—to make sure everything goes smoothly.

*(This response is for general information and should not be taken as medical advice. Always consult with your doctor for guidance specific to your health situation.)*

# 52. I did heart MRI, for which I received contrast. How will my body get rid of the contrast?

**Tracking gadolinium clearance**

OK, let's see—gadolinium-based contrast agents in heart MRIs are mainly excreted by the kidneys through urine, typically within 24 hours. Compromised kidney function can lead to gadolinium retention or NSF risk, emphasizing the importance of hydration.

**Comparing contrast agents**

It's interesting to see that gadolinium-based MRI contrasts typically pass through the kidneys and are excreted in urine. Normal kidney function lets them clear out pretty quickly, often within 24 to 48 hours.

Most MRI contrast agents are gadolinium-based and are cleared from your bloodstream primarily by your kidneys. In other words, once the contrast agent is injected, it circulates through your body and then gets filtered out by the kidneys and excreted in the urine. For people with normal kidney function, most of the contrast typically leaves the body within about 24 hours.

Here are a few key points:

1. **Route of Elimination**
   - Gadolinium-based agents are processed by the kidneys and exit your body when you urinate.
2. **Time Frame**
   - Most is gone within 24 hours, though some sources say 24–48 hours. Staying well-hydrated can help your body clear it more efficiently.
3. **Kidney Function**
   - If you have normal kidney function, there’s usually no special precaution needed aside from staying hydrated.
   - If you have known kidney problems, your doctor may check your kidney function (e.g., creatinine/GFR) before giving gadolinium. In such cases, they may weigh the benefits versus risks more carefully.
4. **Aftercare**
   - You can help the process by drinking plenty of water (unless your doctor has placed you on fluid restrictions).
   - Let your healthcare provider know if you notice any unusual symptoms after the MRI.

Most individuals clear the contrast agent without any problems, and no extra steps are needed besides maintaining adequate hydration. If you’re concerned or have specific health issues, it’s always good to check back with your doctor for personalized guidance.

# 53. I am undergoing heart CT. Will I experience any pain or soreness following the procedure?

I am not a medical professional, but I can share general information that might help you feel more prepared. A heart CT (often called a cardiac CT or coronary CT) is typically a quick, noninvasive imaging procedure. Most people do not experience pain or soreness afterward. Here are a few points to consider:

1. **During the Procedure**
   - If you receive IV contrast (sometimes referred to as “dye”), you may briefly feel a warm sensation when it’s injected. Some individuals also mention a slight metallic taste in their mouth.
   - If you need medication to slow your heart rate (beta-blockers) or a mild sedative, you might feel drowsy or relaxed. These effects usually wear off quickly.
2. **After the Procedure**
   - Most patients do not have any lingering pain or soreness.
   - The IV site on your arm may feel slightly tender where the needle was placed, but this is usually minimal and temporary.
   - In rare cases, people might experience mild side effects from the contrast, such as nausea, but these typically resolve soon after the procedure.
3. **Returning to Normal Activities**
   - You can usually resume your normal activities right after a heart CT, unless your doctor gives specific instructions to wait (for example, if you received any medication that can affect driving).
4. **When to Contact Your Healthcare Provider**
   - If you notice significant swelling or redness at the IV site, or experience unusual symptoms such as worsening pain, shortness of breath, or a rash, you should contact your doctor promptly. While uncommon, these signs could indicate infection or an allergic reaction to the contrast.

Overall, most patients report that a heart CT is straightforward and painless, with minimal after-effects. If you have concerns specific to your health situation, it’s always best to address them directly with your healthcare team. They can provide the most accurate guidance based on your medical history.

# 54. I am undergoing heart MRI. Will I experience any pain or soreness following the procedure?

**Understanding MRI effects**

I'm explaining that a heart MRI typically brings no pain, despite the potential for minor soreness from the IV line. Potential side effects include mild bruising and an initial needle prick.

I’m not a medical professional, but I can share general information about what people typically experience with a heart MRI. An MRI (Magnetic Resonance Imaging) scan is usually a painless procedure that uses a powerful magnet and radio waves to create detailed images of the heart. Here are a few points that might help:

1. **No Direct Pain from the MRI Itself:**
   The MRI process itself does not cause pain. You’ll lie still on a bed that slides into a cylindrical scanner. The machine makes loud knocking or thumping noises, but you’ll usually be given earplugs or headphones for comfort. Many people describe the process as slightly uncomfortable just because of the confined space and the noise, but not painful.
2. **Possibility of an IV Injection:**
   In some heart MRI exams, a contrast agent (often gadolinium-based) is administered through an IV line to help capture clearer images of your heart. You might feel a brief sting when the IV is inserted. Occasionally, people experience minor bruising or soreness at the IV site afterward, but it’s generally mild.
3. **Positioning or Lying Still:**
   Since MRI scans can be lengthy, you might have to lie very still for 30–60 minutes or more, depending on the complexity of the exam. If you have any pre-existing back or joint issues, sometimes staying in one position can cause stiffness or mild soreness. Let your MRI technician know if you’re uncomfortable so they can help you adjust positions or add extra padding.
4. **After the Scan:**
   - Most people can resume normal activities immediately after the MRI.
   - The majority report no post-procedure pain or soreness specifically related to the scan itself.
   - If you received contrast, you might want to stay hydrated to help your body clear it, but that doesn’t typically cause soreness or pain.

If you have specific concerns—especially if you have claustrophobia, anxiety, or any underlying conditions—talk with your medical team before the scan. They may offer options like light sedation or additional comfort measures. Always follow the advice of your healthcare provider, since they’ll have the most accurate information about your personal health needs and can give guidance tailored to you.

# 55. I did heart CT. How soon can I return to my normal activities, such as work or exercise?

**Understanding post-procedure norms**

I'm thinking through post-procedure guidelines for a heart CT scan, generally involving no significant downtime. Immediate resumption of normal, non-sedentary activities is typically permissible.

I’m not a medical professional, but I can share general guidelines based on common practice. In most cases, a heart CT (often a CT angiogram) is a fairly quick and minimally invasive procedure. Here’s what people typically experience:

1. **Immediate Recovery:**
   - If you had **no sedation** (or only very mild sedation), you can usually resume normal activities—including returning to work or light exercise—almost right away. Most people feel back to normal by the time they leave the imaging center.
2. **If Sedation Was Used:**
   - If you received a sedative or stronger medication (sometimes used to help lower the heart rate for better imaging), you may be advised to **rest for the remainder of the day** and avoid driving. Usually by the next day, you can safely go back to your usual routine.
3. **Contrast Dye Considerations:**
   - Heart CTs often involve IV contrast. This usually doesn’t interfere with your ability to exercise or work, but it’s a good idea to **stay well-hydrated** for 24 hours afterward to help your kidneys clear the contrast dye.
4. **Beta-Blockers or Other Medications:**
   - If you were given beta-blockers to slow your heart rate during the test, you might feel more tired than usual for a few hours. This typically wears off by the next day.
   - Check with your doctor regarding any medication adjustments if you regularly take beta-blockers or other heart medications.
5. **Any Discomfort or Side Effects:**
   - Most people feel no lingering discomfort. If you notice any unusual symptoms (like shortness of breath, swelling, rash, or pain at the IV site), contact your healthcare provider.

In general, **most individuals resume normal activities (work, errands, light exercise) the same day** unless your provider gave specific instructions otherwise. If you have special considerations (e.g., pre-existing kidney issues, heart conditions requiring specific follow-up, or complicated medical history), it’s wise to confirm with your doctor.

**Disclaimer:** The information provided here is for general educational purposes and does not replace medical advice. Always follow the guidance of your healthcare team regarding post-procedure care and activity level.

# 56. I did heart MRI. How soon can I return to my normal activities, such as work or exercise?

**Evaluating MRI outcomes**

User asks about post-heart MRI activities. Clarifying, MRI usually doesn't involve downtime unless sedation or contrast is used, which may require specific post-procedural care.

I’m not a medical professional, but I can share general information that may help you understand what to expect after a heart MRI. In most cases, people are able to return to their normal activities (work, exercise, and daily tasks) very soon after the test. Here are a few key points to keep in mind:

1. **No Downtime for Most Routine MRIs:**
   - A cardiac MRI (heart MRI) is a non-invasive imaging technique. Most of the time, if you were not sedated, you can go right back to your usual activities as soon as the scan is over.
   - If you received a contrast agent (often gadolinium), it typically doesn’t require any special recovery period. Drinking water can help your body clear the contrast faster, but that’s typically the only extra step needed.
2. **Consider Sedation or Medications:**
   - If you were given a sedative to help you stay still during the scan, you might feel drowsy for a few hours. In this case, it’s best to avoid strenuous activities (including exercise) for the remainder of the day and follow any specific instructions from the staff.
   - If a stress MRI was performed (where certain medications are used to stimulate or mimic exercise for imaging), you could have lingering mild effects such as feeling flushed or lightheaded, though this normally resolves fairly quickly.
3. **Individual Differences and Underlying Conditions:**
   - Everyone’s recovery can be different, depending on their overall health. If you have a heart condition or other health concerns, it’s important to follow your healthcare provider’s advice for returning to activities and to note any specific instructions they might give based on the reason for your heart MRI.
   - If your provider needed you to avoid certain activities before the test (for instance, stopping certain workouts or adjusting medications), they will guide you on how and when to resume.
4. **Follow Your Provider’s Recommendations:**
   - If your cardiologist or radiology team has given you specific post-scan guidelines—especially related to your heart condition—be sure to follow those instructions for the safest outcome.
   - If you have questions about exercise intensity or how soon to return to work (particularly if your job is physically demanding), it’s a good idea to touch base with your healthcare provider directly.

In general, you can typically resume your usual daily routine relatively quickly after a heart MRI. However, if there were any special instructions or sedation involved, make sure to follow up with your medical team for the most accurate, personalized guidance.

# 57. I did heart CT. Are there any dietary restrictions after the scan?

**Clarifying dietary restrictions**

I'm explaining that typically, post-heart CT scan, there are no special dietary restrictions unless sedation or medications are used. Drinking fluids to flush out contrast is generally recommended.

I’m not a doctor, but in most cases, there are no strict dietary restrictions after a heart CT scan (especially if you received IV contrast). Typically, once the scan is over, you can resume your usual diet.

However, there are a few general tips that many healthcare providers recommend:

1. **Stay well-hydrated**
   - If you were given an iodine-based contrast dye, drinking extra fluids (water) can help flush the contrast from your system.
2. **Check about medications**
   - If you’re on metformin for diabetes, for example, your doctor may give you specific instructions about when to resume it after contrast administration.
3. **Resume normal eating**
   - Unless your radiologist or cardiologist gives you specific instructions, you can return to your normal diet immediately.

If you have any lingering concerns—especially regarding medication use or if you experience any unusual symptoms (e.g., allergic reaction, nausea)—it’s always best to consult your healthcare provider for personalized guidance.

# 58. I did heart MRI. Are there any dietary restrictions after the scan?

Alright, let me see. If the MRI was without contrast, no dietary tweaks are needed. For a gadolinium-based contrast, just stay hydrated. Note any sedation to avoid driving.

**Understanding dietary freedom**

Post-MRI, regular eating habits are feasible. If gadolinium was used, keeping hydrated is essential. Always touch base with a healthcare provider for any unique needs.

I’m not a medical professional, but generally speaking, most people can return to their regular diet immediately after a heart MRI. Here are a few considerations, however, based on common practices:

1. **If No Contrast Was Used:**
   - No specific dietary restrictions. You can usually eat and drink as normal following the scan.
2. **If Gadolinium (MRI Contrast) Was Used:**
   - You can still eat and drink as usual afterward. However, staying well-hydrated (drinking water) helps flush the contrast out of your system more quickly.
3. **If You Received Sedation:**
   - Follow your doctor’s or the imaging center’s guidelines. Some might ask you to avoid heavy meals right away or to wait a certain period before driving or operating machinery.
4. **If You Had a Stress MRI (Using Medication Like Adenosine or Dobutamine):**
   - Generally, no specific dietary restrictions after the scan, but you may be advised to continue drinking water.
   - If you were asked to avoid caffeine or certain foods before the test, you can usually resume them afterward unless your doctor says otherwise.

To be absolutely certain, it’s always best to follow any specific instructions given by your cardiologist or the imaging facility, as individual recommendations can vary. If you have any concerns or notice any unusual symptoms after your MRI—especially if contrast or sedation was involved—contact your healthcare provider for personalized advice.

# 59. I did heart CT. Do I need a follow-up visit or phone call to discuss the results?

**Discussing next steps**

I’m recommending a follow-up visit or phone call to discuss the "heart CT" results. This approach ensures a comprehensive review and consistent with typical imaging procedures.

**Interpreting imaging outcomes**

OK, let me see. Normal or abnormal results require a follow-up with a healthcare professional to understand their significance, especially if no contact has occurred yet.

I’m not a doctor, but in most cases, it’s important to discuss the results of a heart CT (or any imaging test) with a healthcare professional—especially if you haven’t been contacted with clear information about what the scan showed. Here are a few reasons why a follow-up is usually recommended:

1. **Understanding the Findings**
   A heart CT often measures things like coronary calcium scores or looks for signs of blockages in the arteries. Even if the results are normal, it helps to understand what “normal” means in your specific situation. If there’s anything unusual, your doctor will explain what that means and how it could affect your health moving forward.
2. **Next Steps in Care**
   - If the scan is completely normal, your doctor might recommend continuing routine check-ups or preventive measures.
   - If there are findings that need more evaluation (like plaque buildup or other abnormalities), your doctor may discuss additional tests, lifestyle changes, or treatment options.
3. **Medication or Lifestyle Adjustments**
   Even borderline results (e.g., mild plaque or slightly elevated calcium scores) can lead to advice on diet, exercise, or possibly starting medication to lower cholesterol or manage blood pressure.
4. **Peace of Mind**
   Having a conversation—whether by phone, video call, or in-person—lets you ask questions directly. It can be reassuring to have a professional interpretation rather than just relying on a written report.

**Practical Tip:** If you haven’t heard from your healthcare provider’s office, don’t hesitate to call them and ask about scheduling a follow-up or phone call. Some clinics have a process of automatically contacting patients if the results are abnormal, but others recommend you reach out to discuss **any** results.

**In summary:** It’s generally advisable to have at least a phone call or a quick appointment to discuss your heart CT results. That way, you’ll get clear guidance about what your results mean for you and whether any changes to your care plan are necessary. If you’re unsure, reach out to your doctor’s office to confirm next steps.

# 60. I did heart CT. What symptoms after the test should prompt me to call my doctor?

**Noting symptoms**

I'm thinking through potential symptoms after a heart CT scan, like allergic reactions or sedation side effects. If chest pain or unusual symptoms emerge, a doctor should be contacted.

I’m not a medical professional, but I can share general information that might help you make an informed decision about when to follow up with your doctor. If you’ve recently had a heart CT (also known as a cardiac CT scan), you typically can resume your usual activities immediately afterward. However, certain symptoms or side effects can sometimes occur—especially if you received intravenous contrast dye. Here are a few issues to watch out for:

**1. Signs of an Allergic Reaction**

- **Skin reactions:** Hives, itching, rash, or redness that appears soon after the test
- **Swelling:** Especially of the face, lips, tongue, or throat
- **Breathing difficulties:** Wheezing, shortness of breath, or feeling of tightness in the chest

These can indicate an allergic reaction to the contrast dye. If any of these symptoms occur, contact your doctor promptly.

**2. Unusual or Worsening Pain**

- **Chest pain or pressure:** A heart CT itself typically doesn’t cause chest pain afterward, so if you notice any persistent or worsening discomfort in your chest, get medical help.
- **Injection site pain:** Mild tenderness or a small bruise at the IV site (where contrast was injected) can happen, but if pain at the injection site increases, becomes red, swollen, or warm to the touch, you may have an infection or inflammation.

**3. Kidney-Related Issues**

- **Changes in urination:** If you notice a significant decrease in urine output, darker urine, or difficulty urinating, especially if you have a history of kidney problems, let your doctor know. Contrast dye can sometimes affect kidney function.

**4. General Symptoms of Concern**

- **Dizziness or fainting:** Feeling lightheaded or as if you might pass out could indicate an issue with blood pressure or a reaction to medication (e.g., beta-blockers given to slow your heart rate during the scan).
- **Nausea or vomiting that doesn’t resolve:** While mild nausea can sometimes happen, persistent nausea or vomiting might warrant a call to your doctor.

**When in Doubt, Call**

If you’re uncertain about any symptom—especially if it’s severe, worsening, or simply out of the ordinary—it’s always a good idea to check in with your healthcare provider. They’ll be able to determine if what you’re experiencing is routine or if further evaluation is needed.

**Disclaimer:** This information is for general educational purposes and does not replace medical advice from your healthcare provider. If you are experiencing concerning symptoms or have questions specific to your health situation, please reach out to your doctor for personalized guidance.

# 61. I did heart MRI. What symptoms after the test should prompt me to call my doctor?

**Educating on post-heart MRI care**

I'm thinking through post-heart MRI care, emphasizing non-medical advice. Noticing symptoms like chest pain or severe headache could indicate complications, especially if contrast or sedation was involved.

I am not a medical professional, but I can share general information about what is typically advised after a cardiac (heart) MRI. If you have any concerns or are experiencing symptoms that are severe or worsening, please contact a qualified healthcare provider or emergency services promptly.

Here are some post-heart MRI considerations and the signs that usually prompt a call to your doctor:

**1. Allergic Reactions (If Contrast Was Used)**

- **What to watch for**:
  - Hives, rash, or itching
  - Swelling of the face, lips, or throat
  - Difficulty breathing or wheezing
  - Unusual dizziness or fainting
- **Why it matters**: Many heart MRIs use a gadolinium-based contrast agent. While severe allergic reactions are uncommon, they can happen.

**2. Injection Site Problems**

- **What to watch for**:
  - Redness, warmth, swelling, or pain at the IV site
  - Discharge or persistent bleeding
- **Why it matters**: These may signal an infection or inflammation at the IV insertion site.

**3. Unusual or Worsening Cardiac Symptoms**

- **What to watch for**:
  - Chest pain or pressure
  - Significant shortness of breath
  - Irregular heartbeat or palpitations that are new or intensifying
- **Why it matters**: Although an MRI itself doesn’t typically cause these problems, any underlying heart condition could flare or produce new symptoms. If you experience them after a test, it’s important to get an evaluation.

**4. Persistent Nausea, Vomiting, or Headache**

- **What to watch for**:
  - Nausea, vomiting that doesn’t improve
  - Severe or persistent headache
- **Why it matters**: These could be related to contrast side effects, stress/anxiety from the procedure, or other underlying issues.

**5. Dizziness or Fainting**

- **What to watch for**:
  - Feeling lightheaded, unsteady, or having episodes of near-fainting or fainting
- **Why it matters**: Although mild dizziness can sometimes occur after lying still for a long time in the MRI machine, persistent or severe dizziness may indicate something more significant.

**When to Seek Help**

- If any symptoms are severe or worsening.
- If you are worried at all about how you are feeling, especially with cardiac or breathing issues—better to be cautious and seek medical advice.
- If you have any existing heart conditions and develop new symptoms, contact your provider as soon as possible.

**Takeaways**

- **Stay hydrated**: If you received contrast, drinking water helps flush it out of your system.
- **Follow your doctor’s instructions**: They may have given you specific guidelines depending on your health and the test results.
- **Listen to your body**: If something feels “off,” it’s safer to check in with your healthcare team.

**Disclaimer**: The information here is for general educational purposes. You should contact a qualified healthcare provider for any specific concerns, especially if you’re experiencing troubling or severe symptoms.

# 62. Can I travel (or fly) shortly after having a cardiac CT?

**Assessing travel risks**

It's interesting to see that flying post-cardiac CT scan usually poses no restrictions, except if sedation was used or there are unusual heart conditions.

I’m not a medical professional, so you’ll want to confirm with your healthcare provider for advice specific to you. In general, however, most people can travel or fly shortly after having a cardiac CT scan under normal circumstances. Here are some points to keep in mind:

1. **No Residual Radiation Concern:** A CT scan uses X-rays, but there’s no lingering radiation in your body afterward. Once the scan is complete, you aren’t radioactive or posing any risk to people around you.
2. **Use of Contrast Dye:** If you received intravenous contrast dye, your body will naturally eliminate it over time, usually through the kidneys. Most people have no issues with flying after a contrast-enhanced study, but:
   - If you have a history of kidney problems or contrast allergies, be sure to talk to your doctor. They may suggest drinking extra fluids (if medically appropriate) to help clear the contrast or advise you to wait for a short period to monitor for any reaction.
3. **Sedation or Medications:** If you had any sedation or medications to help you relax during the scan, you should:
   - Wait until the sedative’s effects wear off, which could be a few hours.
   - Ensure you’re fully alert and capable of managing airport security, boarding, and any in-flight needs.
4. **Underlying Heart Condition:** If the scan was done due to specific cardiac symptoms (e.g., chest pain, shortness of breath), your doctor might want to evaluate the results before giving you the green light to travel. Make sure you discuss your upcoming travel plans and overall condition with them to confirm there’s no medical reason to delay your flight.
5. **Timing and Follow-Up:**
   - Occasionally, healthcare providers want to review the scan results in person or monitor you for a short time if there’s a possibility of an unusual finding or side effect.
   - If you’re scheduled to fly very soon after the scan, coordinate with your medical team to ensure you receive any important results or post-scan instructions before leaving.

In short, most people can safely travel right away after a cardiac CT, but do check with your physician if you have any concerns about sedation, kidney function, potential allergic reactions, or the condition of your heart itself. Your doctor’s guidance should override any general advice.

# 63. Can I travel (or fly) shortly after having a cardiac MRI?

**Assessing safety**

I've assumed a non-invasive cardiac MRI. After sedation, grogginess might occur. Generally, a flight post-MRI is safe.

**Understanding potential impacts**

If sedation was involved or a stress test was part of the MRI, rest or mild side effects might be needed. Generally, travel post-MRI shouldn't pose medical concerns.

I’m not a medical professional, but in most cases, having a cardiac MRI does not prevent you from traveling—whether by plane or any other means—soon afterward. Here are a few general points to consider:

1. **Non-Invasive Nature of MRI:**
   - A cardiac MRI is typically a non-invasive diagnostic test that uses magnetic fields to create detailed images of your heart. It does not involve radiation, and it does not usually require a lengthy recovery period.
2. **Possible Use of Contrast Agent (Gadolinium):**
   - Sometimes a contrast agent is administered via IV during a cardiac MRI to enhance image quality. Afterward, most people can resume normal activities, including travel, without restrictions.
   - If you have any known kidney issues or allergies, your radiologist or cardiologist will discuss these with you beforehand, but even then, travel is rarely restricted solely because of the contrast.
3. **Medication-Induced Stress for Cardiac MRI:**
   - In some cardiac MRIs, medications that speed up your heart rate or dilate blood vessels (stress agents) might be given to simulate exercise conditions. These can occasionally cause mild side effects (like flushing, shortness of breath, or dizziness).
   - If you experience side effects, it’s advisable to wait until you feel back to normal before traveling, especially if you will be driving yourself to the airport or need to manage luggage. Usually, these effects wear off quickly.
4. **Sedation Concerns:**
   - Most cardiac MRI scans do not require sedation. However, if you did receive sedation (for claustrophobia or other reasons), you may need a few hours before driving or operating machinery. If you’re flying immediately after, ensure you have someone to assist you if you’re still groggy.
5. **Individual Health Factors:**
   - Your doctor might have specific considerations related to the condition being evaluated (e.g., if you have unstable cardiac symptoms). In such cases, they may advise you to delay travel or flying for reasons related to your heart health, rather than the MRI itself.

**Bottom Line**: In the vast majority of situations, you can fly or travel by other means as soon as your cardiac MRI is completed. The main caveat is whether sedation or medication-induced stress was used and how you feel afterward. Always follow the guidance of your cardiologist or the radiology team if they give specific precautions based on your overall health.

If you have any lingering questions or concerns—especially related to recent cardiac issues—it's best to double-check with your healthcare provider before heading to the airport. They’ll give you tailored advice based on your particular cardiac condition and the details of your test.

# 64. Is there a radiation risk with cardiac CT, and how significant is it?

**Evaluating radiation risk**

I’m assessing the radiation risk of cardiac CT scans. Doses usually range from 1 to 10 mSv, depending on the technology used. Advanced methods can significantly reduce exposure.

Cardiac CT does involve exposure to ionizing radiation, but in most cases the overall risk from this exposure is relatively low—especially compared to the value of accurately diagnosing or ruling out significant heart disease. Here are the key points to keep in mind:

**1. Typical radiation doses:**

- A non-contrast CT for calcium scoring might involve about 1–3 millisieverts (mSv) of radiation.
- A contrast-enhanced cardiac CT angiogram (which looks at the coronary arteries) can vary from ~1 mSv (using the most advanced, low-dose techniques) up to ~5–10 mSv or more, depending on the scanner and protocol used.

For perspective, the average person in the United States receives about 3–6 mSv of background radiation per year from natural and environmental sources.

**2. How significant is the risk?**

- The lifetime risk of developing a radiation-related cancer from a single cardiac CT is considered small—roughly estimated in the range of a few additional cases per 10,000 to 100,000 people.
- In the context of someone who may have a significant risk of heart disease, the diagnostic benefits (e.g., detecting blockages or narrowing in the coronary arteries) can substantially outweigh this small additional risk.

**3. Technological advancements and dose reduction:**

- Modern CT scanners have dose-saving features such as prospective ECG gating (also known as “step-and-shoot” techniques), iterative reconstruction, and optimized scanning protocols. These can significantly reduce radiation dose compared to older methods.
- Radiologists and cardiologists follow the “ALARA” principle—keeping radiation “As Low As Reasonably Achievable”—tailoring each exam to the patient's specific needs.

**4. Balancing risk and benefit:**

- Cardiac CT is often performed when noninvasive imaging is crucial for diagnosis or when it can help avoid more invasive procedures.
- If you have concerns, speak with your doctor or radiologist; they can explain why cardiac CT is recommended and whether there are alternative tests that do not use ionizing radiation (for example, cardiac MRI).

Overall, while cardiac CT does expose you to a measurable amount of radiation, the risk of harm from that exposure is generally low—especially when balanced against the critical diagnostic information the test can provide.

# 65. Is there a radiation risk with cardiac MRI, and how significant is it?

Magnetic resonance imaging (MRI) does **not** use ionizing radiation. Instead, it relies on strong magnetic fields and radiofrequency waves to produce images. As a result, a cardiac MRI has essentially **no radiation risk** in the sense of ionizing radiation (the type associated with X-rays, CT scans, or nuclear medicine studies).

Here are a few points to keep in mind:

1. **No Ionizing Radiation**
   - MRI uses radiofrequency (RF) waves, which are non-ionizing. Therefore, the potential risks associated with ionizing radiation—such as radiation-induced cancer—do not apply.
2. **Heat and RF Energy**
   - While there is no ionizing radiation, the RF energy from MRI can cause heating in tissues. MRI machines have built-in safety limits to keep this heating within safe ranges.
3. **Contrast Agents**
   - Some MRI studies, including cardiac MRI, might use gadolinium-based contrast agents to improve image clarity. These agents do not involve radiation but come with their own considerations (e.g., rare risk of allergic reactions or nephrogenic systemic fibrosis in patients with severely reduced kidney function).
4. **Pacemakers and Other Implants**
   - If you have certain cardiac devices (e.g., pacemakers, defibrillators), you should let your healthcare team know. Modern “MRI-conditional” devices are designed to be safe in an MRI environment, but older devices may pose risks.

Overall, from a **radiation standpoint**, cardiac MRI is extremely safe because it does **not** involve ionizing radiation at all. The main safety considerations center around the magnetic field, potential heating effects, and the presence of any implanted devices.

# 66. I have nails in my arm, can I do heart MRI?

I’m not a medical professional, but I can provide some general information that may help guide your next steps. If you have any concerns about your specific situation, it’s very important to speak directly with your doctor or a radiologist.

**1. Why metal in the body matters for MRI**
Magnetic Resonance Imaging (MRI) uses a powerful magnetic field to produce detailed images of the body. Metals that are ferromagnetic (e.g., iron or steel) can be attracted strongly to the magnet. This can pose a risk of movement, heating, or image distortion during an MRI.

**2. Types of metal**

- **MRI-safe/compatible metals:** Some implants are made with non-ferromagnetic materials such as titanium or certain alloys that are generally considered safe in MRI scanners. Many modern surgical implants (e.g., rods, screws, plates) are made with titanium or stainless steel alloys specifically designed to be MRI-compatible.
- **Older or unknown materials:** If the “nails” in your arm were placed some time ago and/or you aren’t sure what metal they contain, it’s crucial to find out exactly what material was used.

**3. Verification process**

- **Documentation or implant card:** If the nails or screws were placed by a surgeon, there should be medical records or implant information available. Often, patients receive implant cards specifying if the hardware is MRI-compatible.
- **Communicate with the MRI facility:** Before scheduling an MRI, tell the radiology team exactly what kind of metal is in your arm. They might request your medical records or imaging (like X-rays) to confirm the type of metal.
- **Safety screening:** MRI facilities conduct a screening process before scans. They will ask detailed questions about implants, devices, or any metal in your body. In certain cases, they may do additional imaging or tests to confirm if the implant is ferromagnetic.

**4. Possible outcomes**

- **Safe to proceed:** If the nails are determined to be MRI-safe, you’ll likely be cleared for the scan.
- **Conditional scan:** Sometimes, scans are done under specific conditions (e.g., a lower magnetic strength, certain positioning, or different protocols).
- **Alternative imaging:** If there is a high risk or if the nails cannot be verified as MRI-safe, doctors might recommend a different imaging modality, such as a CT scan or an ultrasound, depending on what needs to be examined.

**What to do next**

1. **Contact your surgeon or doctor** for records about the nails placed in your arm, including material details.
2. **Communicate with the MRI center/radiologist**; let them know you have metal in your arm. They may want to review your records or perform additional imaging to ensure it’s safe.
3. **Follow professional advice**; if there’s any question about the safety of the metal, alternatives (like CT or certain nuclear imaging methods) may be considered for your heart exam.

Again, the best course of action is to discuss all these specifics with a healthcare professional. They can give you personalized guidance based on the exact type of implants in your arm and your overall medical situation.

# 67. I have an inserted heart device. Can I do heart MRI?

**Considering conditions**

I notice the user asks about MRI safety with heart devices like pacemakers or ICDs. I recommend consulting a medical professional for precise advice.

**Navigating MRI queries**

I note the complexity of MRI safety with heart devices, emphasizing "MRI-safe" or "conditional" labels, and caution about older or incompatible devices. Consult a healthcare professional or refer to the device manufacturer for guidance.

I’m not a medical professional, but I can share some general information that might help you have a more informed discussion with your doctor.

**1. Device type matters**

- **Pacemakers and ICDs (Implantable Cardioverter-Defibrillators):**
  Some older models are **not** compatible with MRI because of the risk that the strong magnetic field can interfere with electronic components or heat up the leads.
  Newer models are often labeled **“MRI-conditional”** or **“MR-safe.”** If your device is one of these, it means you might be able to safely undergo an MRI *under specific conditions*—for example, at certain magnetic field strengths or with the device temporarily reprogrammed by a specialist.
- **Other implants (stents, heart valves, clips, etc.):**
  Many stents and modern heart valve replacements are made of materials that are less likely to pose a risk during MRI. However, the safety varies by brand, type, and the date of implantation.

**2. Checking device specifics**

Each device has a model number and manufacturer. Your cardiologist or electrophysiologist can help verify:

- Whether your device is MRI-compatible or MRI-conditional.
- Specific guidelines from the device manufacturer (like maximum field strength, scanning duration, monitoring requirements, etc.).

**3. MRI safety protocols**

If your device is **MRI-conditional**:

1. **Pre-scan check:** A specialist (often an electrophysiologist) may need to reprogram the device into a safe mode.
2. **Monitoring during the scan:** Your heart rhythm may need close monitoring while you’re in the MRI scanner.
3. **Post-scan check:** The device is usually reprogrammed back to normal settings immediately after the MRI, and a thorough check ensures the device is functioning properly.

**4. Potential risks**

- **Device malfunction:** If the device is not certified for MRI, or if the guidelines aren’t followed, there’s a risk the device could malfunction during or after the scan.
- **Heating of leads:** The leads (wires connected to the device) can sometimes heat up if they’re not designed for the MRI environment.

**5. Next steps**

- **Speak with your cardiologist/electrophysiologist:** They have your complete medical history and device details.
- **Gather device information:** If you have a card or paperwork showing your device’s model number and MRI-compatibility, bring it to your appointment.
- **Radiology consultation:** The radiology department at the facility where you would have the MRI often has a protocol for scanning patients with heart devices.

**Disclaimer:** This information is for general knowledge and does not replace medical advice. Please consult your cardiologist or the device manufacturer’s guidelines to determine whether an MRI is safe in your specific case.

# 68. I am pregnant, can I undergo heart CT?

**Weighing diagnostic options**

Pregnant women should minimize ionizing radiation exposure, using safer alternatives like MRI or ultrasound for imaging. Consult healthcare providers for detailed advice.

I’m not a medical professional, but I can share some general information that may be helpful. If you are pregnant and there is a need to evaluate your heart, your doctor will carefully consider whether a heart CT scan is the best option. Here are a few key points to keep in mind:

**1. Radiation Exposure**

- A CT scan uses ionizing radiation. In general, healthcare providers try to avoid exposing the fetus to radiation unless the benefits significantly outweigh the risks.
- In most cases, if there is an urgent, life-threatening concern, the medical team may decide that the benefit of diagnosing and treating a potential heart problem outweighs the small risk from radiation.

**2. Possible Alternatives**

- If it is not an emergency, doctors might consider alternative imaging methods that do not involve ionizing radiation, such as:
  - **Echocardiography (ultrasound of the heart)** – Often the first imaging choice for heart issues during pregnancy.
  - **MRI (Magnetic Resonance Imaging)** – Does not use ionizing radiation and can provide detailed images of the heart, although availability and the specific information needed might limit its use.

**3. Protective Measures**

- If a CT scan is deemed necessary, there are ways to minimize radiation exposure. Special shielding can help protect both you and your fetus. Protocols can be adjusted to use the lowest radiation dose that still provides diagnostic-quality images.

**4. Timing of the Scan**

- If the heart CT can be delayed and it is safe to do so, your doctor might choose to wait until after delivery—or at least until later in your pregnancy—when the fetus is less vulnerable. However, this depends entirely on the urgency of your heart condition.

**5. Consultation and Decision-Making**

- Ultimately, your healthcare provider (often a cardiologist and/or a maternal-fetal medicine specialist) will balance the medical necessity of the test with the potential risks.
- Ask your care team about all possible alternative tests, the radiation dose you would receive, and how they plan to protect your fetus if a CT scan is necessary.

**Important:** Only a qualified healthcare provider who understands your medical history and specific situation can give you a definitive recommendation. If you are pregnant and concerned about your heart health, please speak directly with your obstetrician and cardiologist. They can discuss the risks, benefits, and alternatives in detail to help you make the most informed decision for both you and your baby.

# 69. I am pregnant, can I undergo heart MRI?

**Assessing MRI safety**

I’m engaging with the question of MRI safety during pregnancy. Generally, MRI is safe beyond the first trimester, but avoiding gadolinium-based contrast is crucial due to potential placental crossing.

**Consulting professionals**

OK, let me see. The user should consult her doctor for MRI safety, avoiding gadolinium if possible. Non-contrast MRI is preferred, and sedation is rarely needed, typically just for immobile patients.

I’m not a medical professional, but I can share general information about MRI during pregnancy that may help you discuss this further with your healthcare providers:

**1. MRI is generally considered safe**
Magnetic resonance imaging (MRI) does not use ionizing radiation (unlike X-rays or CT scans), so there is no radiation exposure to worry about. Large studies and clinical experience suggest that MRI—especially without contrast—is generally safe during all stages of pregnancy. However, because the first trimester is a critical time of fetal development, many healthcare providers prefer to use MRI only if it is absolutely necessary during that period.

**2. Use of contrast agents (like gadolinium) is usually limited**
Gadolinium-based contrast agents can cross the placenta, and their effects on the fetus are not fully understood. Most guidelines recommend avoiding or limiting gadolinium during pregnancy unless it is critical for diagnostic accuracy. If your doctors determine that contrast is important for diagnosing a serious condition, they will discuss the risks and benefits with you.

**3. Timing and necessity are key**

- If there is a pressing cardiac concern (for example, to investigate abnormal heart function, suspected structural heart problems, or to clarify other imaging findings), an MRI might be recommended, even during pregnancy.
- In many heart-related issues, doctors may also rely on (or start with) less invasive tests like an echocardiogram (an ultrasound of the heart), which is often sufficient to diagnose common heart problems without any exposure to magnetic fields or contrast.

**4. Discuss with your obstetrician and cardiologist**
Your healthcare team (including your obstetrician/midwife and cardiologist) will weigh the urgency of the MRI against any potential unknown risks. They’ll consider:

- **How critical** the MRI findings are to making a treatment decision.
- **Whether alternative imaging (e.g., echocardiogram) could provide sufficient information.**
- **The stage of pregnancy** (some doctors may be more comfortable scheduling MRI after the first trimester if the condition allows for safe delay).

**Key Takeaways**

- **MRI itself (without contrast)** is generally viewed as safe during pregnancy when medically necessary.
- **Avoiding gadolinium contrast** is preferred unless there’s a compelling reason to use it.
- **Always inform all healthcare providers** about your pregnancy before scheduling imaging.
- **Echocardiogram** is often the first-line imaging for heart issues during pregnancy, but if MRI is needed, it can be performed with appropriate precautions.

Because every situation is unique, it’s best to have an open discussion with your cardiologist and obstetrician. They know your specific medical history and can guide you to the safest and most effective imaging choice.

# 70. I have kidney problems, can I do heart CT?

**Navigating medical questions**

I’m not a medical professional, but I can share some general information that may help guide your discussion with your healthcare team. Whether or not you can safely undergo a heart CT (also called a cardiac CT or coronary CT scan) often depends on:

1. **Type of CT Scan**
   - **Coronary CT angiography (CTA)** uses iodine-based contrast to visualize the coronary arteries. Iodinated contrast can, in some individuals, affect kidney function (often referred to as contrast-induced nephropathy).
   - **Coronary calcium scoring (non-contrast CT)** does not typically use any contrast. This type of CT evaluates calcium deposits in the coronary arteries and does not pose the same kidney-related risks as scans requiring contrast.
2. **Level of Kidney Function**
   - Doctors often measure kidney function using the estimated Glomerular Filtration Rate (eGFR). If your eGFR is below a certain threshold (often <30 mL/min/1.73 m² is considered significant impairment), many providers will carefully weigh the risks and benefits of using intravenous contrast.
   - If your kidney problems are mild to moderate, your care team may still proceed with a contrast-based heart CT, sometimes with extra precautions.
3. **Preventive Measures**
   - If your medical team deems a contrast-based cardiac CT necessary, they may give intravenous fluids before, during, and after the scan to help protect your kidneys.
   - They may opt for a lower volume or a special type of contrast agent (iso-osmolar or low-osmolar contrast) that is potentially less harmful to the kidneys.
4. **Alternative Imaging Options**
   - **Stress Test (Nuclear or Echo):** A stress test with nuclear imaging or an echocardiogram may help assess blood flow to the heart without exposing you to iodinated contrast.
   - **MRI (Cardiac MRI):** Depending on what information is needed, a cardiac MRI might be an alternative. However, an MRI with gadolinium-based contrast also has potential kidney considerations (for example, the risk of nephrogenic systemic fibrosis in very advanced kidney disease).
5. **Risk vs. Benefit Discussion**
   - In some scenarios—especially if there is a strong suspicion of significant coronary artery disease—getting the detailed anatomical information from a contrast-enhanced CT scan can be critical to guide treatment. Your doctors will weigh this benefit against the risk to your kidney function.
   - If the benefit of accurate heart imaging to diagnose/treat potential heart disease outweighs the kidney risk, your medical team may recommend steps to minimize that risk and proceed.
6. **Working with Your Healthcare Provider**
   - Ultimately, your cardiologist, nephrologist, and radiologist should work together to evaluate your kidney function, assess your overall risk, and decide whether a contrast-based heart CT is the best option for you.
   - Make sure you share detailed information about your renal condition (such as your most recent lab results, eGFR, and any history of contrast-induced nephropathy) with all involved specialists.

**Key Takeaways**

- **Yes, it may still be possible** to do a heart CT if you have kidney problems, but this depends on the severity of your kidney issues and whether the scan requires contrast.
- **Non-contrast scans (like calcium scoring)** pose less risk to kidney function.
- **If contrast is needed**, your medical team may use protective measures (e.g., hydration) or lower-risk contrast agents.
- **Discuss risks and benefits thoroughly** with your healthcare provider, including alternative imaging options if you have significantly reduced kidney function.

**Disclaimer:** This information is for general educational purposes and should not replace personalized medical advice. If you have kidney problems and are considering a heart CT, please consult with your nephrologist and cardiologist to determine the safest and most appropriate approach for you.

# 71. I have kidney problems, can I do heart MRI?

**Evaluating MRI options**

I'm considering whether a heart MRI is feasible with kidney problems. I'm weighing gadolinium-based contrast risks and exploring alternative methods like non-contrast MRI or different contrast agents.

I am not a medical professional, but I can share general information that may help you discuss this issue with your healthcare provider:

1. **Non-contrast MRI**
   - If a heart (cardiac) MRI is done **without** contrast, kidney function typically is **not** a limiting factor. Non-contrast MRI relies on the natural signals from your body’s tissues, so it doesn’t involve injecting any agent that needs to be cleared by the kidneys.
2. **Contrast MRI and Kidney Health**
   - When doctors want more detailed information, they often order **contrast-enhanced MRI** using a gadolinium-based contrast agent.
   - In people with **reduced kidney function**—especially advanced kidney disease (e.g., an eGFR below 30 ml/min)—certain gadolinium-based contrasts can increase the risk of a rare condition called **Nephrogenic Systemic Fibrosis (NSF)**.
   - However, newer “macrocyclic” gadolinium agents carry a **much lower** risk of NSF even for people with reduced kidney function. Your doctor will consider your level of kidney function (your eGFR) to decide whether it is safe to use contrast and which type is safest.
3. **Possible Alternatives**
   - If the risk of using contrast is too high, some centers may use **non-contrast MRI protocols**, specialized **T1/T2 mapping** techniques, or **other imaging methods** such as echocardiography or nuclear imaging.
   - In some cases, a different type of imaging (like **CT or echocardiogram**) might be recommended if gadolinium MRI is deemed risky and a non-contrast MRI cannot provide sufficient diagnostic detail.
4. **Talk to Your Doctor**
   - Discuss your **latest kidney function tests (eGFR)** with your physician or nephrologist. They can help determine if:
     - You can have a regular contrast MRI safely,
     - You need to use specific low-risk gadolinium agents, or
     - You should have an alternative imaging test or a non-contrast MRI study.
5. **Hydration and Monitoring**
   - If you do undergo a contrast MRI, your medical team may recommend **extra hydration** (if your heart and kidney conditions allow) to help flush out the contrast. They will also **monitor your kidney function** around the time of the scan to ensure your body handles it well.

**Key Takeaway**

- **Yes**, many people with kidney problems **can** still have a heart MRI.
- The **most important factor** is whether the MRI requires contrast and what your **kidney function (eGFR)** currently is.
- Always share your medical history with your radiologist and cardiologist; they will choose the safest and most appropriate imaging strategy for you.

**Disclaimer:** This information is for educational purposes and should not replace specific medical advice. Always consult a qualified healthcare professional for guidance on your individual situation.

# 72. If I’m claustrophobic, can I do heart MRI?

**Addressing claustrophobia in MRI**

OK, let me see. Claustrophobic patients can manage anxiety with open MRI machines, sedation, or anti-anxiety meds. Technological progress and advanced options make these techniques more effective than before.

I’m not a medical professional, but I can share some general information that may be helpful. Having claustrophobia does not necessarily mean you *cannot* have a heart MRI. However, cardiac MRIs typically use “closed” MRI machines, which can feel tight or confining. Many people with claustrophobia are able to manage, especially if the healthcare team takes extra steps to help ease anxiety. Below are some considerations and options to discuss with your doctor or imaging center:

**1. Talk to your doctor and care team in advance**

- Let them know about your claustrophobia well before the appointment. They can tailor the experience to your needs or discuss other imaging options if needed.

**2. Mild sedation or anti-anxiety medication**

- Some patients with claustrophobia find it helpful to take a mild sedative or anti-anxiety medication under medical supervision. This can help you relax during the scan.

**3. Wider-bore or “open” MRI machines**

- Although cardiac MRI is often done in a closed-bore scanner (because of the image quality and speed required), some facilities have wider-bore or “short bore” MRI machines that are more open than older models. This design can help reduce the sensation of being in a tight space.
- Completely open MRI machines are *less* commonly used for heart scans, because image quality may not be as high. Still, it’s worth asking about what types of machines are available.

**4. Use of calming techniques**

- Simple practices such as controlled breathing, guided imagery, or listening to music (if the facility allows you to wear headphones) can help distract from the confined space.

**5. Having someone nearby**

- In many centers, you can ask if a friend or family member can be present in the room (following safety rules). Knowing someone is close by can help lessen anxiety.

**6. Plan for possible alternative imaging**

- In some cases, if a cardiac MRI is not absolutely essential or not feasible because of severe claustrophobia, your doctor might suggest another imaging modality (like a CT scan or nuclear imaging). It depends on the specific reason for the exam.

If you’re concerned about tolerating the procedure, the most important step is to communicate your anxiety early on with your healthcare provider. They can explain which strategies are available at their facility—whether it’s medication, a specific type of MRI scanner, or special accommodations—and help you choose the best option.

# 73. My doctor is suggesting heart CT, could a cardiac ultrasound (echo) or stress test replace the CT?

**Weighing diagnostic options**

Cardiac CT scans are typically used for visualizing coronary arteries and detecting blockages, whereas echocardiograms assess structural issues like ejection fraction and valve function. Stress tests evaluate blood flow during exertion.

**Evaluating diagnostic tools**

I’m examining if stress tests or echocardiograms could replace heart CT scans. It depends on the specific diagnostic needs, and consulting a healthcare professional is crucial for guidance.

I’m not a medical professional, but I can share some general information that may help you have a more informed discussion with your doctor. Whether an echocardiogram (cardiac ultrasound) or a stress test could replace a heart CT really depends on why the CT was recommended in the first place. Each test has its own strengths, weaknesses, and specific purposes:

**1. What a heart CT does**

- **Visualizes coronary arteries**: A cardiac CT (especially a CT angiogram) can show detailed images of the coronary arteries, helping detect plaque buildup or blockages.
- **Calcium scoring**: A calcium scoring CT measures calcified plaque in the coronary arteries to estimate heart disease risk.
- **Structural details**: It can show structural details of the heart and surrounding vessels with high resolution.

If your doctor is primarily concerned about coronary artery disease (blockages in your arteries) or wants a calcium score to gauge heart disease risk, a CT scan is often the test of choice.

**2. What an echocardiogram (ultrasound) does**

- **Checks heart valves and pumping function**: An echo is good for evaluating valve function, checking for valve leaks or narrowing, and measuring ejection fraction (how well the heart pumps).
- **Detects structural abnormalities**: Good for seeing the size and shape of the chambers of the heart and spotting some congenital defects or other structural issues.

However, a standard echo **cannot** reliably visualize the inside of coronary arteries. So it won’t tell you much about plaque buildup or blockages. It’s typically more about function (how well the heart is pumping) and checking valves.

**3. What a stress test does**

- **Looks for signs of ischemia (reduced blood flow)**: A stress test—whether done on a treadmill or using imaging (nuclear stress test, stress echo)—checks how well your heart performs under stress. It can detect areas of the heart muscle that might not be getting enough blood flow.
- **Functional assessment**: Helpful if you have symptoms like chest pain or shortness of breath that might only show up during exertion.

A normal stress test reduces the likelihood of significant blockages, but it’s not a direct visualization of coronary arteries. In some cases, stress tests can miss less severe narrowing or plaque.

**4. Why a doctor might choose one test over another**

- **Suspected plaque in coronary arteries**: A CT angiogram is excellent at directly showing blockages or narrowing in the coronary arteries. Neither a standard echo nor a basic stress test can do this as precisely.
- **Valve or pumping function concerns**: An echo is usually the first-line test because it’s non-invasive, quick, and gives good structural information about the heart’s function and valves.
- **Symptoms under exertion**: A stress test is useful if your doctor suspects angina (chest pain due to coronary artery disease) or wants to see how your heart performs when it’s working harder.

**Could an echo or stress test “replace” a CT?**

- **If the primary goal is to detect or measure plaque/blockages in the coronary arteries**, a cardiac CT (especially a CT angiogram) is typically more definitive. An echo or stress test alone usually **cannot** provide the same level of detail regarding arterial plaque.
- **If the concern is about the heart’s pumping function, valve problems, or exercise capacity**, an echo or stress test might be sufficient for diagnosis, and a CT might not be needed.

**Key Takeaways**

1. **Different tests answer different questions** about heart health.
2. **A cardiac CT is often chosen for detailed imaging of the coronary arteries** to look for plaque or blockages, or for a calcium scoring check.
3. **An echo** excels at evaluating valve function, chamber sizes, and overall pump function.
4. **A stress test** helps identify issues that appear under physical stress, such as ischemia.

Your doctor’s recommendation likely comes from their specific concerns about your heart health and what they need to rule out or confirm. It’s best to **ask your doctor why they think a CT scan is necessary** in your situation—whether it’s for calcium scoring, detailed artery imaging, or something else. If you still have questions about alternatives, your doctor can explain if and why an echo or stress test would (or wouldn’t) give them the information they need.

**Disclaimer**

This response is for general informational purposes and should not be taken as medical advice. Always follow up with your healthcare provider for personalized guidance. They know your health history and can give recommendations tailored to your specific situation.

# 74. What is I can't undergo heart MRI, are there any alternatives?

**Surveying alternatives**

I'm mapping out non-MRI imaging options for heart examinations, such as echocardiogram, TTE, TEE, nuclear imaging, and coronary CT.

I’m not a medical professional, but I can share some general information. If you are unable to undergo a heart MRI (often called a Cardiac MRI or CMR), there are several alternative imaging and diagnostic tests that doctors can use to evaluate your heart. Which test is most appropriate depends on the specific clinical question your doctor is trying to answer (e.g., looking at heart structure, function, blood flow, valve issues, or tissue viability). Here are some common alternatives:

**1. Echocardiogram**

- **What it is**: An ultrasound-based test (transthoracic echo or TTE).
- **What it can show**: Heart muscle function, valve structure and function, and overall chamber sizes. It can also measure blood flow across valves and can sometimes detect areas of weak heart muscle.
- **Pros**:
  - Widely available.
  - Noninvasive and does not use radiation.
  - Often provides immediate results in real-time.
- **Cons**:
  - Image quality can vary based on patient’s anatomy or body habitus.
  - May not provide all the detailed tissue characterization that MRI can.

**Transesophageal Echocardiogram (TEE)**

- **What it is**: A specialized form of echo where the ultrasound probe is inserted into the esophagus to get clearer pictures of the heart.
- **Pros**:
  - Can offer more detailed images of valves, heart chambers, and certain structures (like the atria or the aorta) than a standard echo.
- **Cons**:
  - More invasive (requires mild sedation).
  - Not always necessary unless specific structures (e.g., valves, septum, or clots in the atria) need more detailed evaluation.

**2. Cardiac CT (Computed Tomography)**

- **What it is**: Uses X-rays and computer processing to create detailed images of the heart and blood vessels. Often done with contrast dye (CT angiography) to look at coronary arteries.
- **What it can show**:
  - Heart anatomy and coronary arteries (to check for blockages or narrowing).
  - Calcium scoring (to assess the amount of calcium in your coronary arteries).
  - Certain congenital or structural issues of the heart.
- **Pros**:
  - Fast test, widely available.
  - Excellent for looking at coronary artery disease and anatomical detail.
- **Cons**:
  - Uses ionizing radiation (which doctors try to minimize).
  - Requires IV contrast dye in many cases, so patients with certain kidney problems or contrast allergies may not be candidates.

**3. Nuclear Cardiology (Myocardial Perfusion Scans, PET, or SPECT)**

- **What it is**: Imaging techniques (e.g., SPECT or PET scans) using radioactive tracers to evaluate blood flow to the heart muscle, often combined with a stress test.
- **What it can show**:
  - Areas of the heart that aren’t getting enough blood flow (ischemia).
  - Areas of scarred or infarcted tissue.
- **Pros**:
  - Very good at detecting reduced blood flow and tissue viability.
  - Can be done with pharmacological or exercise stress to see how the heart works under strain.
- **Cons**:
  - Involves exposure to small amounts of radioactive tracer.
  - Not as widely available as standard echo or CT in some regions.

**4. Stress Echocardiography**

- **What it is**: An echocardiogram done while the heart is stressed, either by exercise or by medication that simulates exercise.
- **What it can show**: Changes in heart function under stress, which can help identify regions with poor blood supply or other functional abnormalities.
- **Pros**:
  - Noninvasive.
  - No radiation.
- **Cons**:
  - May not provide the same level of detail for tissue characterization as MRI.
  - Image quality is still dependent on ultrasound “windows.”

**5. Cardiac Catheterization (Invasive)**

- **What it is**: A procedure where a catheter is threaded through an artery or vein (usually from the wrist or groin) into the heart to directly visualize the coronary arteries via X-ray and contrast dye.
- **What it can show**: Blockages or narrowing in coronary arteries, direct measurements of intracardiac pressures.
- **Pros**:
  - “Gold standard” for diagnosing coronary artery blockages.
  - Interventions (like stents) can be placed if blockages are found.
- **Cons**:
  - Invasive.
  - Requires contrast and radiation.
  - Generally not used unless there’s a strong suspicion of significant coronary disease or a need for intervention.

**Factors to Discuss with Your Healthcare Provider**

1. **Reason for imaging**: Clarify why a cardiac MRI was initially recommended. Was it for diagnosing scar tissue after a heart attack, assessing valve problems, identifying cardiomyopathies, or something else? The reason guides which alternative imaging is best.
2. **Implants or devices**: If you have a pacemaker, ICD, or other devices that make MRI challenging, some newer devices are MRI-safe or “MRI-conditional.” Ask your doctor if there’s a possibility to do an MRI under special conditions.
3. **Allergies or kidney function**: If you have issues with contrast dyes, that can limit the use of contrast-based CT or invasive angiography.
4. **Claustrophobia or comfort**: If claustrophobia is the only issue, some centers offer open MRI machines or sedation options. Alternatively, an equivalent imaging test may be easier for you to undergo.
5. **Radiation considerations**: If you’ve had multiple imaging tests or have concerns about radiation, you and your doctor may choose an option with little to no radiation (like an echocardiogram).

**Summary**

- If cardiac MRI is contraindicated or not feasible for you, there are multiple other tests—echocardiograms (including transesophageal), stress echocardiograms, cardiac CT scans, and nuclear cardiac imaging—that can be used to evaluate heart structure and function.
- The choice of test depends on the specific information your healthcare team needs. Always discuss with a cardiologist or imaging specialist to weigh the pros and cons, considering your medical history and the particular questions that need answering.

**Disclaimer**: This information is for general educational purposes and does not replace professional medical advice. For personal guidance or a definitive plan, you should consult your cardiologist or another qualified health professional.

# 75. I am supposed to do Heart CT. However, I have allergy to contrast dye—are there alternative options?

**Evaluating alternatives**

OK, let's think this through: Since the patient's allergic to iodine-based contrast, I'm planning further with pre-med steroid regimens for a CTA, MRI, stress echocardiography, or nuclear stress test.

**Crafting alternative plans**

Considering minimal contrast or noncontrast CT; stress test with nuclear imaging or echocardiogram; or cardiac MRI with gadolinium for coronary calcium scoring and plaque burden.

**Identifying solutions**

OK, let me see. I’m exploring noncontrast coronary artery scans, nuclear stress tests, cardiac MRIs, and pre-medication for mild allergies. A glimpse of these options offers a promising progression in management.

I’m not a medical professional, but I can share some general information that might help you have a more informed discussion with your healthcare provider. If you need a heart (cardiac) CT but are allergic to the iodinated contrast dye typically used, there are a few possible alternatives or workarounds to consider:

**1. Pre-Medication Protocols**

For some patients with known contrast allergies, doctors may still use iodinated contrast under controlled conditions by:

- **Pre-medicating** with corticosteroids (like prednisone) and/or antihistamines (like diphenhydramine) hours before the scan.
- **Choosing a low- or iso-osmolar contrast agent,** which tends to be less allergenic than older, high-osmolar agents.

Whether pre-medication is an option depends on the severity of your past reaction and your overall health. If your allergy is severe (e.g., a history of anaphylaxis), your doctor may recommend avoiding iodinated contrast entirely.

**2. Non-Contrast Cardiac CT (Calcium Scoring)**

- A **Coronary Artery Calcium (CAC) Score** can be obtained with a non-contrast CT scan.
- This test measures the amount of calcified plaque in your coronary arteries.
- While it doesn’t show “soft” plaque or give detailed information about specific blockages, it does help assess overall risk of coronary artery disease.

**3. Cardiac MRI (CMR)**

- A **Cardiac MRI** can provide detailed images of the heart’s structure and function.
- Often, cardiac MRI uses a different type of contrast (gadolinium-based), and this may be safer if you only have issues with iodinated contrast.
- You’d need to confirm with your doctor that you don’t have other contraindications to MRI (such as certain implants or kidney issues).

**4. Nuclear Stress Test (Myocardial Perfusion Imaging)**

- A **Stress test with nuclear imaging (SPECT or PET)** uses a radioactive tracer (not iodinated dye) to evaluate blood flow to the heart muscle at rest and under stress.
- It can help identify areas of reduced blood flow or damage but doesn’t directly visualize the coronary arteries like a CT angiogram would.

**5. Stress Echocardiography**

- **Stress echocardiography** (often with exercise or medication-induced stress) uses ultrasound to check the heart’s function and blood flow.
- No contrast dye is required (though sometimes specialized echo contrast may be used, which is typically a different kind of agent than CT contrast).

**6. Other Considerations**

- **Allergy Testing**: If it’s unclear which specific part of the contrast you’re allergic to, your physician might consult an allergist for testing, especially if a CT with iodinated contrast is medically necessary.
- **Severity of Allergy**: The severity and type of reaction you’ve had before are critical in deciding if it’s safe to try a pre-medication approach or better to switch to a dye-free or different-modality test.

**Next Steps**

1. **Discuss thoroughly with your cardiologist or ordering physician**: They’ll consider the diagnostic benefits of a contrast CT against the risk of your allergy.
2. **Consult an allergist**: If there is a possibility of pre-medication, an allergist can help develop a safe protocol.
3. **Explore alternative imaging**: If contrast is simply too risky, talk about MRI, nuclear stress tests, or a non-contrast CT approach (like a calcium score), depending on what specific information your doctors need.

Remember, each patient’s situation is different, so it’s important to speak with your healthcare team. They’ll help you find the safest and most effective imaging strategy for your specific condition and medical history.

# 76. Since there's no radiation risk in heart MRI, why is my doctor recommending heart CT?

**Assessing diagnostic options**

The doctor may opt for a coronary artery CT scan over an MRI due to its superior imaging of coronary arteries and detailed evaluation of coronary artery calcifications, crucial for diagnosing coronary artery disease.

There are a few reasons why your doctor might recommend a heart CT (computed tomography) even though an MRI (magnetic resonance imaging) does not involve ionizing radiation. In many scenarios, a heart CT can provide faster, clearer, or more specific information, especially about the coronary arteries, than an MRI. Here are the main factors that could be influencing your doctor’s decision:

1. **Evaluation of the Coronary Arteries**
   - **Coronary Artery Calcium Score (CACS):** A CT scan is the gold standard for detecting and quantifying calcium in the coronary arteries, which helps estimate your risk for future heart disease. MRI is not typically used for calcium scoring.
   - **Coronary CT Angiography (CTA):** When doctors need detailed visualization of the coronary arteries to look for blockages or narrowing, CT angiography often provides a clearer and faster image. MRI can image blood vessels, but the spatial resolution and speed of a CT can be superior for coronary imaging in many cases.
2. **Specific Clinical Questions**
   - Sometimes, the particular heart issue your doctor suspects (for example, plaque buildup, coronary anomalies, or assessing stents/bypass grafts) is best addressed by CT, which offers a more detailed look at the arteries themselves.
   - Certain heart structures or complications—such as calcified plaques—might be far easier to see on a CT than on an MRI.
3. **Practical Considerations**
   - **Time and Availability:** A CT scan generally takes just a few minutes of actual scan time, whereas an MRI might be longer and is not always as widely available.
   - **Patient Comfort:** Some patients have difficulty tolerating an MRI due to claustrophobia, longer exam duration, or the noise of the scanner.
   - **Cost and Insurance Coverage:** In some healthcare settings, a heart CT scan may be more straightforward and more commonly approved for certain diagnostic pathways.
4. **Balancing the Radiation Risks**
   - Modern cardiac CT scans use advanced dose-reduction techniques, meaning that, while they do involve ionizing radiation, the dose is kept as low as reasonably achievable.
   - In many cases, the diagnostic benefit (i.e., obtaining crucial information about your heart or coronary arteries) significantly outweighs the relatively small risk from the radiation exposure.

**Bottom Line**
Even though an MRI does not use ionizing radiation, a CT might be recommended because it can deliver superior or more direct information regarding the coronary arteries and certain heart structures. If you have concerns about radiation, talk to your doctor about how they weigh the risks and benefits for your specific situation and whether there are alternative diagnostic options.

# 77. I did heart CT, When and how will I receive my results?

**Assessing medical test stages**

OK, let me see. The user is curious about the timeline and process for receiving heart CT results, emphasizing the importance of consulting healthcare professionals for detailed interpretations.

**Mapping the process**

I'm detailing how heart CT results are read by radiologists, sent to the ordering physician, and typically shared via follow-up appointments or patient portals within a few days to weeks.

**Tracking result delivery**

Results from a heart CT typically arrive within a few days to a couple of weeks, often relayed by the ordering physician through phone calls or patient portals.

I’m not a medical professional, but I can share general information about what usually happens after a heart CT (computed tomography) scan:

1. **Radiologist Review**: After your scan, a radiologist (or cardiac imaging specialist) will interpret the images and create a report. This can happen quickly in some facilities (within hours or a day or two) or take a bit longer, depending on their workload.
2. **Communication With Your Doctor**: Once the report is ready, it’s usually sent to the doctor who ordered your test—often a cardiologist or primary care physician. They’ll review the findings and decide how best to share the results with you.
3. **Receiving the Results**:
   - **Phone Call or Email**: Your doctor’s office might call or email you to discuss the results, especially if anything needs prompt attention.
   - **Follow-Up Appointment**: If it’s part of a larger diagnostic workup or if your doctor wants to talk through the details, they may schedule a follow-up appointment.
   - **Patient Portal**: In many healthcare systems, the report may also appear in your electronic health record portal, where you can log in and see results directly (though some portals wait until the doctor has reviewed it before posting).
4. **Typical Timeframe**: In general, you might receive results within a few days to a week, though sometimes it can take longer. If you haven’t heard back after a reasonable time (about a week or two), it’s a good idea to call your doctor’s office and ask if the results are in.
5. **Next Steps**: Depending on the findings, your doctor may:
   - Give you the “all clear.”
   - Suggest more tests (e.g., stress test, echocardiogram, etc.).
   - Discuss treatment or lifestyle recommendations.

If you have specific concerns about the timing or the process, the best step is to contact the office that scheduled or performed your heart CT, or reach out to the doctor who ordered it. They can give you the most accurate information about when and how your individual results will be delivered.

*(This response is for general informational purposes and does not replace professional medical advice. For specific guidance about your situation, please consult your healthcare provider.)*

# 78. I did heart MRI, when and how will I receive my results?

I’m not a medical professional, but I can offer some general guidelines on what to expect after having a cardiac (heart) MRI:

1. **Radiologist Review**
   - First, a radiologist (a doctor who specializes in interpreting imaging) will review the MRI images. This usually takes anywhere from 24 hours to a few days. In some cases, especially if the scan was done urgently or if there is something time-sensitive in the images, the interpretation can be faster.
2. **Report Preparation**
   - After reviewing the images, the radiologist will write a detailed report describing any findings. If you had the MRI performed at a large hospital or imaging center, this process can be quite efficient. However, in smaller facilities or if there is a high volume of scans, it can take several days to complete and finalize the report.
3. **Transmission of Results**
   - The radiologist’s final report is sent to the doctor who ordered the MRI—often a cardiologist or primary care provider. They will then review the results in the context of your overall health and any other tests you might have had.
4. **Receiving Your Results**
   - **Doctor’s Office or Portal**: Your doctor will usually call you or schedule an appointment to discuss the findings. If your healthcare system has an online patient portal, you might be able to view the final report there. Results can appear in the portal as soon as they’re finalized by the radiologist, sometimes before your doctor has a chance to call you, so don’t be alarmed if you see medical jargon you don’t fully understand.
   - **Follow-up Appointment**: Often, doctors prefer to share results during a follow-up appointment so they can explain the findings and outline the next steps for management or treatment, if necessary.
5. **Typical Timeframe**
   - In many places, you can expect to hear back within about **1–2 weeks** for non-urgent scans. If the MRI is part of an urgent investigation (e.g., checking for serious cardiac issues), your doctor might contact you sooner—sometimes within a few days.
6. **What You Can Do**
   - If you haven’t heard back within the timeframe your doctor’s office estimated, it’s reasonable to call and ask about the status of your results.
   - Check your patient portal (if available) regularly to see if a report has been posted.

**Key Takeaways**

- Your cardiac MRI images will be reviewed by a radiologist, who then sends the report to your referring doctor.
- You’ll usually receive results either via a phone call, during a scheduled follow-up appointment, or through a secure online patient portal.
- Most people get an update within **a week or two**, although urgent results can come back faster.
- If you haven’t been contacted when you expect, don’t hesitate to reach out to your doctor’s office for an update.

If you have concerns or experience any unusual symptoms in the meantime, it’s always best to contact your healthcare provider promptly.

# 79. Will I need additional testing after heart CT?

I’m not a medical professional, so I can’t diagnose or give specific medical advice, but I can offer general information. Whether you need additional testing after a heart CT (often called a cardiac CT) depends on several factors, including:

1. **Why the CT was ordered**: If it was done to evaluate specific symptoms—like chest pain—or to screen for coronary artery disease, your doctor may or may not require more tests based on the findings.
2. **What the CT scan shows**:
   - If it shows **no significant issues**—clear coronary arteries, no signs of plaque or abnormal structures—further testing might not be necessary.
   - If it shows **some abnormalities**—like mild plaque, potential blockages, or other structural concerns—you might need follow-up testing, such as a stress test, echocardiogram, or cardiac catheterization, for a closer look.
3. **Personal risk factors**: People with risk factors like high blood pressure, high cholesterol, diabetes, smoking history, or a strong family history of heart disease might need more thorough follow-up, even if the CT scan is relatively normal. Your healthcare provider may want to keep a closer eye on your heart health.
4. **Symptom persistence or progression**: If you continue to have unexplained symptoms—chest pain, shortness of breath, or palpitations—additional tests may be recommended, regardless of the CT findings.

**Examples of Possible Follow-up Tests**

- **Stress test (exercise or pharmacological)**: Assesses how your heart performs under stress and checks for exercise-induced issues.
- **Echocardiogram**: An ultrasound of the heart, which can look at valve function and heart muscle movement.
- **Cardiac MRI**: A more detailed imaging test to evaluate the structure and function of the heart.
- **Cardiac catheterization (angiogram)**: A more invasive test to directly visualize your coronary arteries and possibly treat blockages with stents if needed.

**Next Steps**

- **Discuss results with your doctor**: They know your medical history and can interpret the CT in the context of your overall health.
- **Ask about lifestyle and preventive measures**: Even if no additional tests are needed, you can discuss steps to maintain a healthy heart—diet, exercise, and controlling any risk factors like high blood pressure or high cholesterol.
- **Stay alert for new or worsening symptoms**: Let your medical provider know if anything changes.

Ultimately, only your cardiologist or healthcare team can tell you if you need more tests after your heart CT. If you’re unsure about the next steps, it’s always a good idea to schedule a follow-up appointment to review the results in detail.

# 80. I am undergoing heart MRI. Can I obtain copies of the images or medical reports for my personal records?

Yes. As a patient, you typically have the legal right to obtain copies of your imaging studies (like MRIs, CTs, X-rays) as well as your official medical reports. In the United States, this right is covered by HIPAA (Health Insurance Portability and Accountability Act). Other countries have similar regulations ensuring patient access to medical records.

Here are some practical steps:

1. **Ask Your Healthcare Provider or Radiology Center**
   - During or after your scan, let the facility staff (technologist, nurse, front desk) know that you would like copies of your images and the radiologist’s report. They might direct you to the imaging library or medical records department.
2. **Submit a Written Request (if needed)**
   - Some institutions require a formal, written request or completion of a release-of-information form. You can usually obtain the form from the hospital or practice’s website, or you can ask the staff at the medical records department.
3. **Check How the Images Are Provided**
   - Many centers now provide images in a digital format on a CD, DVD, or USB drive. Some have secure online portals where you can view or download the images and reports directly.
   - If you want hard copies of the report or film copies of the images, specify this in your request. Be aware that there may be a fee for producing film copies.
4. **Inquire About Fees**
   - Under most laws, you cannot be charged a fee to simply view your records, but facilities can charge reasonable fees for copying and mailing or for digital media. Always ask about cost up front.
5. **Check Turnaround Time**
   - The time it takes to receive copies may vary from a couple of days to a few weeks. Clarify how long it will take when you make your request.
6. **Retaining the Records**
   - Once you receive the copies, it is wise to keep them in a secure location. Make backup copies (digital or physical) so you have them in the event they are needed by other healthcare providers.

If you run into any problems obtaining your records, your facility’s patient advocacy department (sometimes called a patient ombudsman) can often help. In most areas, there are clear regulations in place obligating healthcare providers to release medical records to patients within a specific timeframe.

# 81. If my heart CT turns to be normal, does that guarantee I’m free from heart disease?

Noticing CT limitations

I am not a medical professional, so it’s always best to discuss any health concerns with your doctor. However, in general:

- **A normal heart CT (computed tomography) scan is reassuring** because it often shows that there are no major blockages or significant calcifications in the coronary arteries and no obvious structural abnormalities of the heart or large blood vessels.
- That said, **no single test can completely guarantee the absence of all heart disease**. Some forms of heart disease may not show up on a CT scan, especially early microvascular disease or subtle functional issues. Additionally, electrical conduction problems, certain types of valvular heart disease, or issues that only manifest under physical stress may not be fully captured on a standard CT.
- **Risk factors matter.** Even if your heart CT is normal, you should continue to manage cardiovascular risk factors (e.g., high blood pressure, high cholesterol, diabetes, smoking, obesity, and family history) through healthy lifestyle changes and periodic evaluations.
- **Other tests might be needed** if you have concerning symptoms or significant risk factors. For example, a stress test (exercise or pharmacologic), an echocardiogram, or advanced imaging like a cardiac MRI might be used to evaluate heart function, detect subtle structural problems, or diagnose conditions that are not always visible on a CT scan.

In summary, a normal heart CT is a positive sign but does **not** provide an absolute guarantee that all forms of heart disease are ruled out. If you have ongoing symptoms or risk factors, talk with your healthcare provider about whether additional testing or ongoing monitoring is appropriate.

# 82. If my heart MRI turns to be normal, does that guarantee I’m free from heart disease?

I’m not a medical professional, so I can’t give specific medical advice. However, in general, a normal heart MRI is reassuring but it does not completely rule out all forms of heart disease. Here are a few key points to consider:

1. **Different Tests Look for Different Things**
   - A cardiac MRI can provide detailed images of the heart’s structure and function (including the heart muscle, valves, and surrounding tissue).
   - However, **not all types of heart disease are fully captured** by MRI. For example, coronary artery disease (blockages in the arteries) is often more thoroughly evaluated by other imaging tests, such as a **coronary angiogram**, cardiac CT scan, or stress tests.
2. **Disease Progression Can Vary**
   - Some heart conditions may be in very early stages or might be intermittent in nature (e.g., arrhythmias). These might not be fully apparent on a single MRI study.
   - A normal MRI only reflects the state of your heart at the time the images were taken.
3. **Clinical Picture Matters**
   - Doctors interpret MRI results alongside **other tests** (electrocardiograms, echocardiograms, blood tests, stress tests) and your overall clinical picture (symptoms, risk factors, family history).
   - If you have significant risk factors (e.g., high blood pressure, high cholesterol, diabetes, smoking history, family history of heart disease), your doctor may still recommend further evaluation.
4. **Follow-Up**
   - If symptoms persist or new symptoms develop, your cardiologist may suggest **additional tests** or **repeat imaging** in the future.
   - Medicine is about probability, not absolute certainty. A normal test lowers the likelihood of significant heart disease but does not eliminate every possibility.

In short, a normal heart MRI is an excellent sign and suggests you do not have major structural problems with your heart. It significantly reduces the chances of certain heart conditions. However, it **does not 100% guarantee** you are free from every possible form of heart disease. If you continue to experience cardiac-related symptoms or have concerns, it’s important to follow up with your doctor for a comprehensive evaluation.

# 83. My doctor says that my heart CT is positive, how certain is that?

**Assessing test accuracy**

OK, let's break down the reliability of a positive heart CT result. It depends on specificity, sensitivity, and predictive values. More details are needed to offer a definitive assessment.

**Navigating false positives**

It's interesting to see that addressing false positives, reliability, and sensitivity is crucial. Discussing next steps with a doctor and considering confirmatory tests is advised.

I’m not a medical professional, but I can share general information that might help clarify how “certain” a positive heart CT (often referring to a cardiac CT angiogram or coronary calcium scan) might be. The certainty of any diagnostic test result depends on several factors:

**1. The Type of Heart CT**

- **Coronary CT Angiography (CCTA)**: A detailed imaging test that looks at the coronary arteries. It’s often used to detect blockages or narrowing in the arteries.
- **Coronary Artery Calcium (CAC) Scan**: A simpler form of CT that measures calcium deposits in the coronary arteries and generates a “calcium score.”

Each test has its own sensitivity (probability of detecting disease if present) and specificity (probability of a negative result if disease is absent). A “positive” result in:

- **CCTA** could mean there is a visible plaque or narrowing in your coronary arteries.
- **CAC scan** indicates a nonzero calcium score (or a certain threshold), suggesting some degree of coronary artery disease.

**2. Sensitivity and Specificity**

- **Sensitivity**: Many heart CT tests, especially CCTA, have high sensitivity (often in the range of 90–95% or higher) for detecting significant coronary artery disease. This means it rarely misses disease (few false negatives).
- **Specificity**: This can vary more (commonly between 65–90% for CCTA, for example), meaning there is a chance of false positives—situations where the test suggests disease when there may be little or no clinically significant disease.

**3. Pre-Test Probability and Positive Predictive Value**

A key piece of the “certainty” puzzle is your **pre-test probability**—basically how likely you were to have heart disease before the test, based on factors like:

- Age
- Family history of heart disease
- Smoking history
- Cholesterol levels
- Presence of diabetes or high blood pressure
- Symptoms (chest pain, shortness of breath, etc.)

If your pre-test probability is high (say you have multiple risk factors and concerning symptoms), a positive test result is more likely to be truly “positive.” Conversely, if your risk is very low and you have no symptoms, the odds of a false positive increase.

**4. What Does “Positive” Mean?**

- **For a CCTA**: A positive result may mean doctors have seen plaques or narrowing that need further evaluation, possibly with stress testing, invasive angiography, or a more detailed risk assessment.
- **For a Calcium Scan**: A positive result typically means a calcium score above zero. The higher your score, the higher your risk of significant coronary artery disease. However, the exact implications depend on your overall clinical picture.

**5. Next Steps if Your CT Is Positive**

1. **Review the Findings with Your Doctor**: Ask for specifics about the extent of any blockages or the severity of calcification.
2. **Discuss Additional Tests**: Depending on the result and your clinical situation, your doctor might recommend:
   - A **stress test** (exercise or pharmacological).
   - An **invasive coronary angiogram** (though not always necessary unless the CT findings are significant or you have concerning symptoms).
   - **Blood tests** to check cholesterol, inflammatory markers, etc.
3. **Lifestyle and Medication**:
   - If there’s evidence of coronary artery disease, your doctor might discuss cholesterol-lowering medications (such as statins), blood pressure control, dietary changes, exercise, and other preventive measures.

**6. Key Takeaways**

- A positive heart CT often has high sensitivity, meaning it’s quite good at picking up signs of disease. Therefore, a truly diseased artery is usually detected.
- However, **specificity** can be more moderate, so false positives are possible—particularly in lower-risk individuals.
- Interpretation of a “positive” result is influenced by your overall risk profile (age, risk factors, symptoms).
- Always discuss results in detail with your cardiologist or primary care physician, who can clarify whether the finding is significant and what further steps, if any, are needed.

**In Summary**

A “positive” result on a heart CT is typically a good indicator that further evaluation or risk-reduction measures may be warranted—but it does not always mean you have advanced coronary artery disease that will necessarily cause symptoms or heart attacks. The true certainty depends on the test’s accuracy (sensitivity/specificity), your personal risk factors, and how the images correlate with any clinical signs or symptoms you might have.

If you have concerns about how reliable your specific test results are—or what the next best step might be—your best move is to have a detailed conversation with your doctor (or even seek a second opinion from a cardiologist) to interpret the findings in the context of your overall health.

**Disclaimer**: This information is for general educational purposes and is not a substitute for a professional medical evaluation. Always follow the advice of your healthcare provider for personal medical concerns.

# 84. My doctor says that my heart MRI is positive, how certain is that?

**Identifying medical implications**

OK, let me see. The user's doctor reported a positive heart MRI. This suggests an abnormal condition could be present, making the likelihood of an issue more probable.

I’m not a medical professional, so please consider this information as general guidance and not personalized medical advice. If your doctor said that your heart MRI came back “positive,” it typically means that the images or measurements suggest an abnormality or finding that may point to a specific heart condition. However, the term “positive” can be interpreted differently depending on the context and the exact imaging findings.

Here are a few general points to consider when thinking about how certain a “positive” MRI result might be:

1. **Test Sensitivity and Specificity**
   - **Sensitivity** refers to a test’s ability to correctly identify those with a particular condition (true positives).
   - **Specificity** refers to a test’s ability to correctly identify those without the condition (true negatives).
   - If an MRI has high sensitivity and specificity for a particular heart problem, a “positive” finding can be more reliable. However, no test is 100% accurate, and there is always a risk of false positives (the test says there’s an abnormality when there isn’t one) and false negatives (the test misses an actual abnormality).
2. **Clinical Correlation**
   - Doctors usually rely on more than just an imaging test. They’ll also look at symptoms, physical exam findings, blood tests, and other imaging or diagnostic procedures when evaluating heart conditions.
   - If your symptoms, exam findings, and other tests are all pointing in the same direction as the MRI result, that increases the likelihood that the MRI is accurate in detecting a real issue.
3. **Quality of the Imaging and Interpretation**
   - The accuracy also depends on the imaging center’s equipment quality and how experienced the radiologist or cardiologist is in interpreting cardiac MRIs.
   - Sometimes, if an MRI is borderline or unclear, your doctor may recommend a second imaging study or a different type of test (e.g., cardiac CT, stress test, echocardiogram) for confirmation.
4. **Confirmation with Additional Testing**
   - Depending on the suspected heart condition, your doctor may order follow-up diagnostic procedures to confirm the finding. For instance, they may suggest a cardiac catheterization, stress MRI, or an echocardiogram.
5. **Communication with Your Healthcare Team**
   - The best step is to speak with your doctor about what “positive” means in your specific case and how certain they are based on the imaging and your overall clinical picture. Ask about the possibility of false positives, what follow-up tests might be needed, and how they arrived at this conclusion.

**Key Takeaway**: A “positive” heart MRI indicates that the imaging showed something your doctor believes is significant. The certainty of this result depends on the test’s accuracy, your clinical context, and your doctor’s interpretation. If you have concerns or need more clarity, discussing them directly with your medical team is the best course of action. They can provide details tailored to your individual situation and advise on any further tests or steps needed to confirm the diagnosis.
