# Supplementary material for: Large Language Models for Patient Education in Cardiovascular Imaging: Prospective Observational Comparative Study
Source: JMIR Form Res. 2026 Aug 26;10:e95883. doi: 10.2196/95883 (PMC13559148; doi:10.2196/95883)
Supplement: Supplementary file 4 [file formative_v10i1e95883_app4.docx]

# 1. I am undergoing cardiac CT. What is a cardiac CT scan, and Why might I need one?

**What is a Cardiac CT Scan?**

A **Cardiac CT (Computed Tomography) Scan** is a specialized imaging test that uses X-rays and advanced computer technology to create detailed 3D images of the heart, coronary arteries, and surrounding structures. It is a **non-invasive** procedure that helps in evaluating heart conditions and blood vessels without the need for catheterization.

During the scan, a contrast dye (usually iodine-based) is often injected into a vein to enhance visualization of the coronary arteries, allowing doctors to assess for blockages, calcium buildup, or other abnormalities.

**Why Might You Need a Cardiac CT?**

A cardiac CT is typically recommended for several reasons, including:

1. **Coronary Artery Disease (CAD) Assessment**
   - To check for narrowing or blockages in the coronary arteries (atherosclerosis).
   - If you have symptoms like chest pain or shortness of breath but an unclear diagnosis.
2. **Calcium Scoring (Coronary Artery Calcification Test)**
   - Helps assess the amount of calcium buildup in the coronary arteries, which can indicate heart disease risk.
   - Often used in individuals with risk factors (high cholesterol, high blood pressure, diabetes, smoking, family history).
3. **Pre-Surgical or Pre-Procedural Evaluation**
   - Before a heart surgery or procedures like **coronary artery bypass grafting (CABG)** or **valve replacement**.
   - To assess the anatomy of the heart before interventions such as transcatheter aortic valve replacement (TAVR).
4. **Congenital Heart Disease Evaluation**
   - If there are suspected structural abnormalities in the heart, especially in younger patients or those with congenital heart disease.
5. **Aortic Disease Evaluation**
   - To detect **aortic aneurysms** (widening of the aorta) or **aortic dissections** (tears in the aortic wall).
6. **Evaluation of Heart Tumors or Masses**
   - If a suspected tumor or mass is found on echocardiography, a CT scan can provide more detail.
7. **Post-Intervention Assessment**
   - To check the patency (openness) of coronary stents or bypass grafts after previous procedures.

**What to Expect During a Cardiac CT?**

- You may be given a **beta-blocker** to slow your heart rate for better image quality.
- A contrast dye may be injected to highlight blood vessels.
- You will lie on a scanning table while the CT machine takes images.
- The procedure is **painless** and usually takes **10-15 minutes**.
- Afterward, you may be asked to drink plenty of fluids to help flush out the contrast dye.

**Are There Any Risks?**

- **Radiation exposure** (low dose, but should be considered, especially if repeated scans are needed).
- **Allergic reaction** to the contrast dye (rare, but possible).
- **Kidney concerns** in people with pre-existing kidney disease (contrast dye may worsen function in some cases).

Would you like more details on how to prepare for your scan?

# 2. I am undergoing cardiac MRI. What is a cardiac MRI, and why might I need one?

A **Cardiac MRI (Magnetic Resonance Imaging)** is a non-invasive imaging test that provides detailed images of the heart's structure, function, and blood flow using strong magnetic fields and radio waves. It does not use ionizing radiation, making it safer compared to some other imaging techniques like CT scans.

**Why Might You Need a Cardiac MRI?**

Your doctor may order a **cardiac MRI** to evaluate various aspects of your heart, including:

1. **Assessment of Heart Structure and Function**
   - Evaluates heart muscle (myocardium), chambers, and valves.
   - Measures ventricular function (ejection fraction, wall motion abnormalities).
2. **Detection of Cardiomyopathies**
   - Identifies hypertrophic, dilated, or restrictive cardiomyopathies.
   - Detects fibrosis, scarring, or inflammation of the heart muscle.
3. **Coronary Artery Disease & Ischemia**
   - Evaluates myocardial perfusion (blood supply to the heart muscle).
   - Detects previous heart attacks (myocardial infarctions) and areas of scarring.
4. **Congenital Heart Disease**
   - Useful for diagnosing and monitoring congenital heart defects.
5. **Heart Valve Diseases**
   - Assesses valve function, stenosis (narrowing), or regurgitation (leakage).
6. **Myocarditis & Pericarditis**
   - Detects inflammation or infections affecting the heart or its surrounding sac (pericardium).
7. **Aortic Disease & Aortic Stenosis**
   - Evaluates aortic aneurysms, dissections, or congenital abnormalities.
8. **Cardiac Tumors or Masses**
   - Identifies any abnormal growths or thrombi in the heart.
9. **Evaluation of Extracellular Volume (ECV) and T1 Mapping**
   - Used in research and advanced cardiac imaging to assess diffuse myocardial fibrosis, as in **your meta-analysis project** on aortic stenosis.

**How to Prepare for a Cardiac MRI**

- **Inform Your Doctor** if you have any metal implants, pacemakers, or claustrophobia.
- **Avoid Caffeine** (if stress MRI is needed).
- **Wear Comfortable Clothing** (without metal zippers or buttons).
- **Follow Fasting Instructions**, if required for contrast-enhanced studies.

# 3. I am undergoing heart CT. How would this test help diagnose my condition?

A **heart CT (cardiac computed tomography)** is a non-invasive imaging test that provides detailed images of your heart and blood vessels. Depending on the specific type of cardiac CT you are undergoing, it can help diagnose various heart conditions, including:

**1. Coronary Artery Disease (CAD)**

- A **coronary CT angiography (CTA)** can detect narrowing or blockages in the coronary arteries caused by **atherosclerosis (plaque buildup)**.
- It is useful for evaluating symptoms such as chest pain and ruling out **coronary artery disease**.

**2. Calcium Scoring (Coronary Artery Calcium Scan)**

- Measures the amount of **calcified plaque** in your coronary arteries.
- Higher calcium scores indicate an increased **risk of heart disease or future heart attacks**.

**3. Structural Heart Diseases**

- Identifies congenital or acquired abnormalities in heart structure, including:
  - **Aortic stenosis or regurgitation** (narrowing or leaking of the aortic valve).
  - **Mitral valve disease**.
  - **Congenital heart defects**.

**4. Aortic and Pulmonary Conditions**

- Detects **aortic aneurysms** (abnormal dilation of the aorta) and **aortic dissections** (life-threatening tears in the aortic wall).
- Assesses **pulmonary embolism** (blood clots in the lungs) if a CT pulmonary angiogram is done.

**5. Heart Function and Myocardial Tissue Evaluation**

- Some cardiac CT scans, especially with **contrast**, help assess:
  - **Left ventricular function** (pumping ability).
  - **Myocardial fibrosis or scarring**, which may be seen in **heart failure or myocarditis**.
  - Extracellular volume (ECV) and T1 mapping (if part of research or specialized protocols) to assess **myocardial tissue characteristics**.

**6. Pre-Procedural Planning**

- If you are undergoing **transcatheter aortic valve replacement (TAVR)** or **coronary interventions**, CT helps plan the procedure.
- It aids in preoperative evaluation for **coronary bypass surgery**.

**How Will It Help Diagnose Your Condition?**

The usefulness of the test depends on **why your doctor ordered it**. Based on your background (radiology research and cardiovascular imaging interest), you might also be aware that:

- If you have symptoms of **chest pain or shortness of breath**, it can check for coronary artery disease or structural problems.
- If you have risk factors (high cholesterol, high blood pressure, smoking, diabetes), calcium scoring can assess your heart disease risk.
- If your concern is related to **aortic stenosis**, myocardial ECV/T1 measurements (if available) might help assess myocardial remodeling.

Would you like help interpreting your results once you get them?

# 4. I am undergoing heart MRI. How would this test help diagnose my condition?

A **cardiac MRI (CMR)** is a highly detailed imaging test that provides comprehensive information about your heart’s structure and function. It helps diagnose and assess various heart conditions based on the sequences and parameters used. Depending on your symptoms and suspected condition, CMR can provide insights in several ways:

**1. Assessment of Heart Structure & Function**

- Measures **left and right ventricular function**, including ejection fraction (EF).
- Detects **wall motion abnormalities** that may indicate ischemia or cardiomyopathy.
- Evaluates **heart chamber sizes** to diagnose dilation or hypertrophy.

**2. Myocardial Tissue Characterization (T1 & T2 Mapping, ECV, LGE)**

- **Late Gadolinium Enhancement (LGE)**: Identifies myocardial scarring or fibrosis (e.g., past heart attacks, cardiomyopathies, myocarditis).
- **T1 and T2 Mapping**: Helps in detecting diffuse fibrosis, inflammation, or edema (e.g., myocarditis, amyloidosis).
- **Extracellular Volume (ECV) Quantification**: Measures fibrosis, which can be useful in assessing conditions like **aortic stenosis**, heart failure, or infiltrative diseases.

**3. Evaluating Ischemic and Non-Ischemic Heart Disease**

- **Ischemic Heart Disease (Coronary Artery Disease)**
  - Detects previous heart attacks (infarction).
  - Assesses for viable myocardium to determine if revascularization (e.g., stenting, bypass surgery) may help.
- **Non-Ischemic Cardiomyopathies**
  - Differentiates between **dilated cardiomyopathy (DCM), hypertrophic cardiomyopathy (HCM), arrhythmogenic cardiomyopathy, and restrictive cardiomyopathy**.
  - Helps in **amyloidosis, sarcoidosis, Fabry disease**, and other infiltrative conditions.

**4. Valvular Heart Disease**

- Evaluates valve function, regurgitation, or stenosis with precise measurements.
- Can complement echocardiography in complex cases.

**5. Congenital Heart Disease**

- Detects structural defects like atrial or ventricular septal defects.
- Assesses shunt physiology and complex congenital anomalies.

**6. Aortic Disease**

- Identifies **aortic aneurysms, dissections, and coarctation**.

**7. Pericardial Disease**

- Diagnoses **pericarditis, pericardial effusion, and constrictive pericarditis**.

If you have specific symptoms or concerns, I can help interpret how MRI findings might relate to your case. Let me know what you’re looking for!

# 5. I am undergoing heart CT. Which is considered more accurate CT or MRI?

The choice between **CT (Computed Tomography) and MRI (Magnetic Resonance Imaging)** for heart imaging depends on the specific clinical question being addressed. Each modality has its strengths and limitations:

**CT (Cardiac CT)**

✅ **More accurate for:**

- **Coronary artery disease (CAD)**: CT coronary angiography (CTCA) is highly accurate for detecting coronary artery stenosis and plaques.
- **Calcium scoring**: Detects coronary artery calcification, helping in cardiovascular risk stratification.
- **Structural heart evaluation**: Good for assessing congenital heart defects, aortic diseases, and heart valve anatomy.
- **Speed**: Faster than MRI, making it ideal for emergency settings (e.g., suspected coronary artery blockage).

❌ **Limitations:**

- Uses **ionizing radiation** (though dose is optimized in modern scanners).
- Requires contrast (iodine-based), which may be problematic for patients with kidney issues or allergies.
- Provides limited functional information compared to MRI.

**MRI (Cardiac MRI)**

✅ **More accurate for:**

- **Myocardial tissue characterization**: Superior in assessing myocardial infarction, fibrosis, edema, and inflammation (e.g., myocarditis, cardiomyopathies).
- **Viability assessment**: Helps differentiate viable vs. non-viable myocardium before revascularization.
- **Valvular and congenital heart disease**: Provides detailed functional and anatomical assessment.
- **No radiation exposure**, making it safer for repeated imaging.

❌ **Limitations:**

- Longer scan time (may be uncomfortable for some patients).
- Requires breath-holding, which may be difficult for certain patients.
- Not suitable for patients with metal implants (e.g., pacemakers, some stents).

**Which is more accurate?**

- **For coronary artery disease** → **CT is better** (CT coronary angiography is highly sensitive for detecting stenosis).
- **For heart function and tissue characterization** → **MRI is better** (detailed assessment of myocardial fibrosis, inflammation, and viability).

**What is your indication for the heart CT?** If you’re undergoing **CT for coronary artery evaluation**, it is one of the most accurate and non-invasive methods. If your concern is **cardiomyopathy, myocarditis, or viability assessment**, MRI might be more informative.

# 6. I am undergoing heart MRI. Which is considered more accurate CT or MRI?

When it comes to **cardiac imaging**, both **MRI (Cardiac MRI - CMR)** and **CT (Cardiac CT - CCTA)** have their strengths and are used for different purposes. However, in many cases, **Cardiac MRI is considered more accurate** than CT for certain applications, especially in **functional and tissue characterization**.

**Comparison of Accuracy: MRI vs. CT**

| **Feature** | **Cardiac MRI (CMR)** | **Cardiac CT (CCTA)** |
| --- | --- | --- |
| **Anatomy & Morphology** | Excellent, especially for soft tissues | Excellent for coronary arteries and calcifications |
| **Tissue Characterization** | **Gold standard** (differentiates myocardial infarction, fibrosis, edema, etc.) | Limited ability for tissue characterization |
| **Function (Ejection Fraction, Wall Motion, Volume, etc.)** | **Most accurate** for assessing heart function | Good, but less accurate than MRI |
| **Coronary Artery Imaging** | Not optimal for coronary arteries | **Best modality** for detecting coronary artery disease (CAD) |
| **Myocardial Viability** | **Best modality** for viability and scar assessment | Can assess viability using delayed contrast but is not as accurate as MRI |
| **Perfusion (Ischemia assessment)** | **Can assess ischemia non-invasively** | Less commonly used for perfusion analysis |
| **Radiation Exposure** | No radiation | **Uses ionizing radiation** |
| **Speed & Availability** | Takes longer, may require breath-holding | Faster, widely available |

**Which is More Accurate for Your Case?**

- **If you are undergoing a heart MRI**, it is likely for **functional assessment, myocardial tissue characterization, or viability assessment**.
- If the goal is to **evaluate coronary artery disease**, **CT is more accurate**.
- If the goal is to assess **heart function, myocardial tissue, inflammation, fibrosis, edema, or perfusion**, **MRI is more accurate**.

Let me know why you're undergoing a **heart MRI**, and I can give you a more specific answer! 🚑💙

# 7. Why would my doctor recommend a cardiac CT instead of a cardiac MRI?

Your doctor might recommend a **cardiac CT** instead of a **cardiac MRI** for several reasons, depending on your specific clinical situation and the type of information needed. Here are some key reasons why a cardiac CT might be preferred:

**1. Evaluating Coronary Arteries (Coronary Artery Disease - CAD)**

- **Coronary CT Angiography (CCTA)** is excellent for detecting and ruling out coronary artery disease (CAD).
- It provides a **high-resolution** view of the coronary arteries and can detect **plaques, stenosis (narrowing), and calcium deposits** better than MRI.
- A **coronary calcium score (CAC)** via CT can also help assess your risk of future heart disease.

**2. Speed and Accessibility**

- Cardiac CT is **faster** than MRI, often taking just **5–10 minutes** compared to 30–60 minutes for MRI.
- It is more widely available in many hospitals and clinics.

**3. Patients with Metal Implants or Claustrophobia**

- **MRI is contraindicated** in patients with certain metal implants (e.g., older pacemakers, defibrillators, or metallic clips).
- Patients with **claustrophobia** may tolerate CT better since it's quicker and less enclosed.

**4. Superior for Specific Structural and Vascular Abnormalities**

- CT is better for evaluating **coronary artery anomalies, aortic disease (e.g., aortic dissection, aneurysms), and pericardial calcifications**.
- It is also useful in planning for **TAVR (transcatheter aortic valve replacement)** and **coronary bypass graft assessment**.

**5. Emergency Situations**

- In **acute chest pain or suspected pulmonary embolism (PE) or aortic dissection**, cardiac CT provides a **rapid diagnosis**.
- It is often the preferred test in the emergency department setting.

**6. Patients with Poor MRI Tolerance**

- MRI requires patients to stay still for long periods, making it challenging for those with **arrhythmias, severe heart failure, or an inability to hold their breath**.
- CT is less affected by motion artifacts.

**When is Cardiac MRI Preferred?**

Cardiac MRI is better for:

- Assessing **myocardial tissue characterization** (e.g., myocarditis, cardiomyopathies, infarct detection, fibrosis, edema).
- Evaluating **cardiac function** in greater detail.
- Diagnosing **complex congenital heart disease**.

**Bottom Line**

Your doctor likely recommended a **cardiac CT** if they need **detailed coronary artery imaging, quick results, or if MRI is contraindicated** in your case. However, if they need **more information on heart muscle function and tissue characterization**, a cardiac MRI might be a better choice.

Would you like more details on your specific condition?

# 8. Why would my doctor recommend a cardiac MRI instead of a cardiac CT?

Your doctor may recommend a **cardiac MRI (CMR) instead of a cardiac CT (CCT)** for several reasons, depending on the clinical scenario, the type of information needed, and your individual health factors. Here are some key reasons:

**1. Soft Tissue Characterization**

- **Cardiac MRI** is superior for evaluating **myocardial tissue characteristics**, such as edema (inflammation), fibrosis, and scarring. This is crucial for diagnosing **myocarditis, cardiomyopathies, or myocardial infarction** without the use of contrast agents in some cases.
- **Cardiac CT**, on the other hand, is excellent for **coronary artery evaluation** but does not provide detailed tissue characterization.

**2. No Ionizing Radiation**

- **CMR** does not use radiation, making it a better option for **younger patients or those requiring multiple follow-ups**.
- **CCT** exposes the patient to ionizing radiation, which is a concern, particularly in younger individuals or those with cumulative radiation exposure.

**3. Functional and Flow Assessment**

- **CMR** can provide highly accurate measurements of **ventricular function (ejection fraction, volumes), valvular function, and blood flow dynamics** using techniques like phase-contrast imaging.
- **CCT** can estimate these parameters but is less precise for functional assessment compared to MRI.

**4. Better for Evaluating Complex Congenital Heart Disease**

- **CMR** is preferred for assessing **complex congenital heart defects**, vascular anomalies, and post-surgical follow-ups due to its ability to generate 3D reconstructions and visualize blood flow without contrast.

**5. Stress Testing and Myocardial Perfusion**

- **CMR** can assess **ischemia (stress-induced heart disease) and myocardial viability** using stress perfusion imaging and late gadolinium enhancement (LGE).
- **CCT** is not commonly used for stress imaging.

**6. Viability Assessment in CAD**

- **CMR** can detect viable and infarcted myocardium using **late gadolinium enhancement (LGE)**, which is useful in deciding whether a patient might benefit from revascularization (PCI or CABG).
- **CCT** is less effective for assessing viability.

**When Would a Doctor Prefer Cardiac CT Instead?**

- If the **main concern is coronary artery disease (CAD)**, **CCT is often preferred** because it provides **detailed coronary artery imaging** with high spatial resolution.
- If a patient has **metal implants** (e.g., pacemaker, defibrillator) or is **claustrophobic**, **CCT** may be chosen instead.

**Summary: Which is Better?**

- **CMR is preferred** for **soft tissue assessment, functional evaluation, congenital heart disease, and viability studies**.
- **CCT is preferred** for **coronary artery evaluation, detecting calcifications, and rapid assessment of CAD**.

Would you like more information based on your specific situation?

# 9. How do I know if I’m a good candidate for a cardiac CT?

You may be a good candidate for a **cardiac CT (computed tomography) scan** if you meet any of the following criteria:

**1. Assessing Coronary Artery Disease (CAD) Risk**

- You have **intermediate risk** for CAD (based on risk factors like high cholesterol, smoking, hypertension, diabetes, or family history).
- You have **atypical chest pain** or other symptoms suggesting CAD, but not severe enough for invasive testing.
- You need a **coronary artery calcium (CAC) score** to assess your risk of future heart disease, especially if you’re between **40–70 years old** and have risk factors.

**2. Evaluating Known or Suspected CAD**

- You have **chest pain** that may be due to blockages in your coronary arteries.
- Your doctor suspects **coronary artery anomalies** or other structural abnormalities.
- You have **equivocal or inconclusive stress test results** and need further evaluation.

**3. Preoperative Cardiac Assessment**

- You are undergoing **non-cardiac surgery** but have significant cardiovascular risk factors.
- You need a **pre-TAVR (transcatheter aortic valve replacement) assessment** for aortic stenosis.

**4. Assessing Coronary Artery Bypass Grafts (CABG) or Stents**

- You have had **coronary artery bypass surgery**, and your doctor wants to check for graft patency.
- You have a history of **stents** and need non-invasive imaging to assess for restenosis.

**5. Congenital and Structural Heart Disease**

- You have **congenital heart defects** that require non-invasive imaging.
- You need an evaluation of **cardiac tumors, pericardial disease, or cardiac masses**.

**6. Evaluating Cardiac Function and Other Conditions**

- You need an **evaluation of the aorta** (for aneurysm or dissection).
- You need to assess **cardiac chamber sizes and function** in cases where echocardiography or MRI is inconclusive.

**Who May NOT Be a Good Candidate?**

- **High heart rate** (above 65 bpm), as motion artifacts may affect image quality.
- **Severe kidney disease** (due to contrast dye risk).
- **Pregnancy** (unless absolutely necessary).
- **Known severe allergy to contrast dye** (alternative imaging like MRI may be preferred).

If you're unsure, your doctor may use **risk calculators** or order a stress test to determine whether a cardiac CT is right for you. Let me know if you need more details!

# 10. How do I know if I’m a good candidate for a cardiac MRI?

I’m not a medical professional, but I can share some general information that might be helpful. Cardiac MRI (Magnetic Resonance Imaging) is typically used by healthcare providers to get detailed images of the heart’s structure and function. Whether you’re a good candidate often depends on your specific symptoms, medical history, and the diagnostic questions your doctor is trying to answer. Below are some common reasons for and considerations about having a cardiac MRI:

**1. Common Reasons to Consider a Cardiac MRI**

1. **Assessment of Heart Structure and Function**
   - Evaluating the size of the heart’s chambers, the thickness of the heart muscle walls, and blood flow patterns.
   - Checking for heart muscle damage (for example, after a heart attack or in myocarditis).
   - Evaluating cardiac function in conditions such as heart failure or cardiomyopathy.
2. **Checking for Congenital Heart Defects**
   - A cardiac MRI can provide a detailed look at complex congenital heart anomalies in both children and adults.
3. **Evaluating Heart Valve Issues**
   - Getting precise measurements of valve structure, blood flow through the valves, or valve leaks/narrowing.
4. **Detecting Scarring or Inflammation**
   - Visualizing areas of scarred heart tissue (e.g., from past heart attacks) or actively inflamed areas (e.g., in myocarditis).
5. **Further Assessment After Other Tests**
   - Sometimes, if a test such as an echocardiogram or cardiac CT scan shows something unusual, a cardiac MRI might help clarify the findings.

**2. Considerations That Affect Suitability**

1. **Safety Around Metal or Implants**
   - MRI uses powerful magnets. Certain types of metal implants (e.g., pacemakers, older stents, some joint replacements) may be problematic or require special MRI-compatible devices.
   - Always inform your doctor or MRI technician about any implants or metal in your body.
2. **Claustrophobia or Comfort in Enclosed Spaces**
   - MRI machines can feel confining. If you have severe claustrophobia, you might need medication to relax or seek a facility with an “open” MRI (though those are less common for cardiac imaging).
3. **Kidney Function**
   - Contrast agents used in MRI (like gadolinium) are processed by the kidneys. If your kidney function is significantly impaired, your doctor may decide against using certain contrasts or may use a different imaging test.
4. **Allergies**
   - Some people are allergic to contrast agents. Your healthcare provider will review allergies or past reactions to imaging dyes.
5. **Pregnancy**
   - MRI without contrast is generally considered safe in pregnancy, but guidelines vary depending on the trimester. MRI with contrast typically requires more caution.
6. **Physiological Stability**
   - You need to lie still for the duration of the scan. People who have severe difficulty lying down or holding still (e.g., due to back pain, breathing issues) may require special accommodations or sedation.

**3. Deciding if You’re a Good Candidate**

1. **Speak with Your Healthcare Provider**
   - The single most important step is consulting a doctor (cardiologist or primary care) who knows your medical history. They can help you decide whether the detailed information from a cardiac MRI is necessary or if another imaging method—like an echocardiogram, CT scan, or nuclear stress test—would be better.
2. **Review Your Medical Records and History**
   - If you have known heart issues (e.g., prior heart attacks, arrhythmias, unusual symptoms like chest pain or shortness of breath), your doctor might want a more detailed look at the heart muscle or valves using MRI.
3. **Assess the Risk-Benefit Balance**
   - Like all diagnostic tests, a cardiac MRI has potential benefits (better diagnosis, clearer pictures) and certain drawbacks (cost, time, issues with claustrophobia or implants). Your healthcare provider will weigh these before recommending it.

**4. Next Steps**

- **Consult a Cardiologist**
  - They can interpret any existing test results (like ECG, stress tests, echocardiograms) and decide if a cardiac MRI will add helpful information.
- **Communicate Any Concerns**
  - If you have implants, metal fragments, kidney issues, or anxiety about enclosed spaces, tell your doctor. They can plan accommodations or alternative tests.
- **Follow Pre-Scan Instructions**
  - If you proceed with a cardiac MRI, follow instructions about fasting, medication adjustments, or removing jewelry and accessories.

**Important Note**

This information is general and should not replace personalized medical advice. If you suspect you need a cardiac MRI or have heart-related concerns, it’s best to speak with a qualified healthcare professional who can evaluate your individual situation.

# 11. Do I need to fast before my cardiac CT?

**Yes, fasting is generally recommended before a cardiac CT scan, especially if contrast dye (iodinated contrast) is used. Here are the typical fasting guidelines:**

1. **Fasting Duration: You may be asked to fast for 4 to 6 hours before the scan to reduce the risk of nausea or vomiting from the contrast dye.**
2. **Water: You are usually allowed to drink clear water before the test.**
3. **Caffeine & Stimulants: Avoid caffeine and smoking for at least 12 hours before the scan, as they can increase your heart rate, making it harder to get clear images.**
4. **Medications: Continue taking your regular medications unless instructed otherwise by your doctor. Some beta-blockers may be given before the scan to slow your heart rate for better imaging.**

**Special Cases:**

- **Diabetic Patients: If you take metformin, you may need to pause it before the test (check with your doctor).**
- **Renal Patients: If you have kidney issues, hydration might be emphasized to protect kidney function from the contrast dye.**

**Always confirm specific instructions with your radiology department or cardiologist. Would you like help interpreting results if you get a report later?**

# 12. Do I need to fast before my cardiac MRI?

It depends on the type of **cardiac MRI** you are having. Here are the general guidelines:

1. **Standard Cardiac MRI (Without Contrast)**
   - **Fasting is usually not required** unless your doctor or imaging center advises otherwise.
2. **Cardiac MRI With Contrast (Gadolinium-Based Contrast Agent)**
   - **No fasting required in most cases.**
   - Some institutions may ask you to avoid heavy meals before the exam to reduce the risk of nausea.
3. **Cardiac MRI With Stress Test (Adenosine, Dobutamine, or Regadenoson Stress MRI)**
   - **Fasting for at least 4–6 hours before the test is usually required.**
   - Avoid caffeine (coffee, tea, chocolate, soda, energy drinks) for **12–24 hours** before the test, as it can interfere with stress agents.
4. **If You Have Diabetes**
   - Check with your doctor about managing blood sugar levels if fasting is required.

Always confirm with your imaging center, as specific instructions may vary.

# 13. I am undergoing cardiac CT. Can I take my usual medications on the morning of the test?

It depends on the type of **cardiac CT** you're undergoing and the specific medications you're taking. Here are general guidelines:

**1. Routine Medications**

- **Antihypertensives (Blood Pressure Medications)**:
  - **Beta-blockers (e.g., Metoprolol, Atenolol)** → Usually encouraged, especially if needed for heart rate control before a **coronary CT angiography (CCTA)**.
  - **Calcium channel blockers (e.g., Amlodipine, Diltiazem)** → Generally safe to take.
  - **ACE inhibitors (e.g., Lisinopril, Ramipril) or ARBs (e.g., Losartan, Valsartan)** → Sometimes held to avoid interactions with contrast, especially in **kidney disease** patients.
- **Diuretics (e.g., Furosemide, Hydrochlorothiazide)**
  - May be held to **prevent dehydration** and **reduce contrast-induced kidney injury** (especially if contrast is used).
- **Diabetes Medications:**
  - **Metformin** → Sometimes held on the day of the scan and for 48 hours **after contrast-enhanced CT** to prevent lactic acidosis (especially in kidney disease patients).
  - **Insulin** → Adjust dosing if fasting is required to **prevent low blood sugar**.
- **Antiplatelets/Anticoagulants (e.g., Aspirin, Clopidogrel, Warfarin, DOACs like Apixaban, Rivaroxaban)**
  - Typically **not** stopped for a routine **cardiac CT**, but check with your doctor.

**2. If You're Getting a Coronary CT Angiography (CCTA)**

- **Beta-blockers may be prescribed before the test** to lower heart rate for better imaging.
- **Caffeine & Stimulants (e.g., coffee, tea, energy drinks, nicotine, certain decongestants like pseudoephedrine)** should be **avoided for at least 12 hours** before the scan to prevent increased heart rate.

**3. If You Have Contrast Allergy or Kidney Disease**

- If you have a history of **contrast dye allergy**, your doctor may prescribe **steroids & antihistamines** before the scan.
- If you have **chronic kidney disease (CKD)**, you might need **hydration before and after the test** to protect kidney function.

**What You Should Do**

✅ Take most routine medications with **small sips of water**, unless told otherwise.
✅ Avoid **caffeine and stimulants** before a **CCTA**.
✅ If on **Metformin or diuretics**, check with your doctor.
✅ If you have **kidney disease or contrast allergy**, follow any special instructions.

Would you like specific advice based on your medications?

# 14. I am undergoing cardiac MRI. Can I take my usual medications on the morning of the test?

It depends on the type of cardiac MRI you're having and the medications you take. Here are general guidelines:

**1. Routine Cardiac MRI (without stress test)**

- You can **usually take all your medications** as normal on the morning of the test.
- If you have diabetes and take **metformin**, check with your doctor, as some protocols may require stopping it before contrast-enhanced scans.
- Continue blood pressure, cholesterol, and other regular medications unless instructed otherwise.

**2. Cardiac MRI with Stress Testing (e.g., using Dobutamine or Adenosine)**

- You may need to **stop certain medications**, including:
  - **Beta-blockers (e.g., Metoprolol, Atenolol, Propranolol)** – These can interfere with heart rate responses needed for the test.
  - **Calcium channel blockers (e.g., Diltiazem, Verapamil)** – Sometimes discontinued for stress tests.
  - **Caffeine-containing medications (or foods/drinks like coffee, tea, energy drinks, chocolate)** – Usually avoided **24 hours before** adenosine-based stress MRI.
- If you take **asthma medications (e.g., Theophylline)**, discuss with your doctor, as some may interact with adenosine.

**Best Steps:**

- **Check your MRI appointment instructions** – Your doctor or imaging center should provide specific guidance.
- **If unsure, call the MRI facility or your doctor** to confirm whether you should take or skip any medications.

Would you like me to check for any specific medication interactions with your test?

# 15. I am undergoing cardiac CT. Are there any dietary restrictions before the test?

Yes, there are some dietary restrictions before a **cardiac CT**, especially if it involves **coronary CT angiography (CCTA)** or the use of **contrast dye**. Here are the key dietary guidelines:

**1. Avoid Caffeine for 12–24 Hours Before the Test**

- No coffee, tea, soda, energy drinks, or chocolate.
- Caffeine can increase heart rate, making it harder to obtain clear images.

**2. Avoid Heavy or Fatty Meals for 4–6 Hours Before the Test**

- A light meal is okay, but avoid greasy or high-fat foods.
- A heavy meal can affect contrast absorption and cause nausea.

**3. No Eating or Drinking for 2–4 Hours Before the Test**

- This helps reduce the risk of nausea from contrast dye.

**4. Drink Plenty of Water (Unless Instructed Otherwise)**

- Staying hydrated helps with contrast dye clearance from your kidneys.

**5. Avoid Alcohol and Smoking**

- Alcohol and smoking can affect heart rate and blood vessel function.

**6. If You Are Diabetic or on Metformin:**

- You may need to **stop metformin** before and after the scan if contrast is used.
- Consult your doctor about adjusting diabetes medications.

**7. Medication Instructions**

- Take your usual medications unless instructed otherwise.
- **Beta-blockers may be given** to slow heart rate for better images.

Would you like any more specific guidance based on your condition or medications?

# 16. I am undergoing cardiac MRI. Are there any dietary restrictions before the test?

For a **cardiac MRI**, dietary restrictions depend on whether the test includes contrast (gadolinium) or stress imaging:

**General Dietary Guidelines for Cardiac MRI**

1. **If your MRI is WITHOUT contrast**
   - No specific dietary restrictions. You can eat and drink normally.
2. **If your MRI is WITH contrast (Gadolinium-based contrast agent, GBCA)**
   - Generally, no strict fasting is required, but some institutions recommend **avoiding heavy meals 2–3 hours before the scan** to prevent nausea.
   - Stay well-hydrated unless advised otherwise.
3. **If your MRI includes a stress test (Adenosine, Regadenoson, or Dobutamine Stress MRI)**
   - **Avoid caffeine** (coffee, tea, chocolate, sodas, energy drinks, and even decaffeinated beverages) for **12–24 hours before the test**, as caffeine can interfere with vasodilators used in the stress portion.
   - **Avoid heavy meals** at least 3–4 hours before the scan to reduce the risk of nausea during stress induction.
   - Some centers recommend **avoiding alcohol and smoking** for 24 hours before the test.
4. **If you have diabetes**
   - If fasting is required (for stress MRI), follow your physician’s instructions regarding insulin or diabetes medications.
   - Bring a snack for after the test in case your blood sugar drops.
5. **If you have kidney disease**
   - Gadolinium contrast may require pre-test kidney function testing (eGFR), and hydration is encouraged.

Would you like me to check with a specific institution for their guidelines?

# 17. I am undergoing heart CT. Will I need blood tests or other preliminary checks beforehand?

Yes, before undergoing a **cardiac CT (heart CT scan)**, you may need some **preliminary tests** or **preparation steps**, depending on the specific type of CT scan and your medical history. Here’s what to expect:

**1. Blood Tests**

You may need blood tests to check the following:

- **Kidney Function (Creatinine & eGFR)**: If your scan involves **contrast dye (iodinated contrast)**, doctors may require a **serum creatinine test** to assess kidney function. This is particularly important if you have:
  - Diabetes
  - Kidney disease
  - High blood pressure
  - Age > 60 years
- **Thyroid Function (TSH, if indicated)**: If you have a history of thyroid problems, especially **hyperthyroidism**, your doctor may check your thyroid function before contrast administration.

**2. Heart Rate & Blood Pressure Monitoring**

- If your scan requires **coronary CT angiography (CCTA)**, you may need a **low heart rate (usually <65 bpm)** for better image quality.
- A **beta-blocker (e.g., metoprolol)** may be given before the scan to slow your heart rate.
- **Nitroglycerin spray/tablet** might also be used to dilate the coronary arteries for better imaging.

**3. Blood Sugar Check (for Diabetic Patients)**

- If you take **metformin**, you might be advised to **stop it for 24-48 hours** after the contrast-enhanced CT scan to prevent lactic acidosis, especially if kidney function is impaired.
- If fasting is required, you should check how to adjust insulin or diabetes medications.

**4. Allergy History (for Contrast-Enhanced CT)**

- If you have a history of **iodine or contrast dye allergy**, inform your doctor. You may need **steroid premedication** before the scan.

**5. Other Precautions**

- **Fasting**: Some centers require fasting for **2–4 hours** before the scan, especially if contrast dye is used.
- **Hydration**: Drinking plenty of water helps flush out the contrast after the scan.
- **Pregnancy**: If you are female and of childbearing age, you may need to confirm **not being pregnant** before the scan.

Would you like guidance on preparing for your specific type of heart CT (e.g., calcium scoring, coronary CT angiography)?

# 18. I am undergoing heart MRI. Will I need blood tests or other preliminary checks beforehand?

For a **cardiac MRI**, the need for **blood tests** or other **preliminary checks** depends on the type of MRI you are undergoing and your medical history. Here’s what you might expect:

**1. Blood Tests (If Needed)**

You may need **blood tests** before your MRI in certain situations:

- **Kidney Function Tests (Creatinine, eGFR)**
  - If your MRI involves **contrast (gadolinium-based contrast agent)**, a kidney function test may be required, especially if you have **kidney disease, diabetes, hypertension**, or are over **60 years old**.
- **Coagulation Tests (INR, PT, aPTT) or CBC**
  - If you are scheduled for an MRI with **cardiac stress perfusion**, some hospitals check your coagulation profile or platelet count.
- **Iron Studies**
  - If your MRI includes *T2 imaging for iron overload assessment**, ferritin and transferrin saturation might be checked.

**2. Other Preliminary Checks**

- **ECG (Electrocardiogram)**: Some MRI protocols, especially those involving cardiac function assessment, require an ECG before or during the scan to synchronize image acquisition with your heartbeats.
- **Pregnancy Test (if applicable)**: If you are a woman of childbearing age and there is any possibility of pregnancy, some centers may require a test before a contrast-enhanced MRI.
- **Allergy History**: If you have had a previous reaction to MRI contrast, inform your doctor.

**3. Device Screening (If Applicable)**

- If you have **pacemakers, implantable defibrillators, or other metal implants**, additional **safety screening** will be needed.
- Some **MRI-compatible pacemakers** require special programming before the scan.

**4. Fasting Instructions**

- **For contrast-enhanced MRI**: You may be asked to fast for **4–6 hours** before the scan.
- **For stress cardiac MRI** (with adenosine or dobutamine): You may be asked to avoid caffeine for **24 hours** before the test.

Would you like me to check specific hospital protocols based on your location?

# 19. I am undergoing heart CT. What should I wear to the appointment?

For your **heart CT scan**, it's best to wear **comfortable, loose-fitting clothing** and avoid any items that contain metal. Here are some specific recommendations:

**Clothing:**

- Wear a **plain T-shirt** or **loose-fitting top** without metal zippers, snaps, or buttons.
- Opt for **sweatpants, leggings, or athletic shorts** without metal parts.
- Avoid **bras with underwires** (sports bras are a good alternative).

**Accessories:**

- **Remove all jewelry** (necklaces, earrings, rings, bracelets, watches).
- Avoid **metal hairpins, wigs with metal clips, or hair extensions with metal**.
- If you wear **glasses, dentures, or hearing aids**, you may need to remove them.

**Additional Notes:**

- If **contrast dye** is used, you may be asked to **fast for a few hours** before the scan.
- Avoid **caffeine** (including coffee, tea, and energy drinks) **for 12–24 hours** before the test, as it can affect your heart rate.
- If you have **a history of contrast allergies**, let your doctor know beforehand.
- If you're on **beta-blockers or other heart medications**, follow your doctor's instructions.

Would you like any additional details about the procedure?

# 20. I am undergoing heart MRI. What should I wear to the appointment?

For your **heart MRI appointment**, follow these guidelines on what to wear and bring:

**Clothing:**

- Wear **loose, comfortable clothing** without any **metal** (zippers, buttons, snaps, underwire bras).
- **Avoid activewear with metallic fibers** (some sportswear contains metallic threads that interfere with MRI).
- Choose **cotton-based** clothing (sweatpants, T-shirts).
- If unsure, you might be asked to change into a hospital gown.

**Jewelry & Accessories:**

- **Remove all jewelry** (rings, necklaces, earrings, piercings, watches).
- **No hairpins, clips, wigs with metal**.
- If you have a **permanent retainer or dental implants**, inform the MRI team.

**Other Items to Avoid:**

- **No makeup or lotions with metallic components** (some contain fine metallic particles).
- **No nail polish** with glitter.
- Leave **credit cards, keys, phones, and electronics** outside the MRI room.

**Medical Considerations:**

- If you have **metal implants, pacemakers, or prior surgeries**, notify the MRI center in advance.
- If you have **claustrophobia**, you can discuss sedation options with your doctor.
- For **contrast MRIs**, you may need an IV line for gadolinium contrast.

Would you like tips on preparing for the scan or what to expect during the MRI?

# 21. I am undergoing heart CT. Do I need to avoid strenuous activity or exercise before the test?

Yes, you should generally avoid strenuous activity or exercise before a **heart CT scan**, particularly if you are undergoing a **coronary CT angiography (CCTA)** or any CT scan that requires heart rate control. Here’s why:

**Why Avoid Strenuous Exercise Before a Heart CT?**

1. **Heart Rate Consideration:**
   - For **CCTA**, a lower heart rate (ideally below 60 bpm) is required for optimal image quality.
   - Exercise can temporarily **increase heart rate**, making it harder to obtain clear images.
   - Beta-blockers may be given before the scan to slow your heart rate if needed.
2. **Blood Pressure Effects:**
   - Strenuous exercise can temporarily raise blood pressure, which might affect the scan.
3. **Potential for Artifacts:**
   - Increased blood flow and circulation from recent exercise might cause imaging artifacts, leading to less accurate results.

**How Long Should You Avoid Exercise?**

- Most guidelines suggest avoiding **moderate to vigorous exercise for 12–24 hours** before the scan.
- Light activities like walking are usually fine, but avoid activities that significantly elevate your heart rate.

**Other Pre-Test Precautions**

- **Caffeine & Stimulants:** Avoid caffeine (coffee, tea, energy drinks, or chocolate) **at least 12 hours before** the scan, as it can increase heart rate.
- **Medications:** If you're taking beta-blockers or calcium channel blockers, follow your doctor’s instructions regarding dosage before the scan.
- **Fasting:** Some heart CT scans require fasting for **4–6 hours** before the test, especially if contrast dye is used.

Would you like specific advice based on the type of heart CT you’re undergoing?

# 22. I am undergoing heart MRI. Do I need to avoid strenuous activity or exercise before the test?

Yes, you should generally avoid strenuous activity or exercise before a **cardiac MRI**, especially if the test includes certain specialized imaging techniques like **stress MRI** or **perfusion imaging**. Here’s why:

1. **Resting Heart Rate & Blood Flow** – Exercise increases heart rate and blood flow, which may affect imaging results, particularly for stress perfusion MRI or T1/ECV mapping.
2. **Gadolinium Contrast (if used)** – If your MRI involves contrast, avoiding intense exercise ensures your body is in a steady state, preventing transient changes in blood flow that could alter imaging.
3. **Arrhythmias & Artifacts** – Strenuous exercise may lead to temporary arrhythmias or increased respiratory motion, which can degrade image quality.

**General Recommendations:**

- Avoid intense exercise **at least 24 hours before** the scan.
- Stay **hydrated** and follow any fasting instructions (if applicable).
- If your MRI includes **adenosine or regadenoson stress testing**, follow specific instructions about caffeine and food restrictions.

If your test is a **routine cardiac MRI without stress imaging**, avoiding heavy exercise **on the day of the test** is usually sufficient. However, confirm with your radiology center if there are any specific restrictions.

# 23. I am undergoing heart CT. Should I arrive earlier than my scheduled time, and how long will the overall visit take?

For a **heart CT scan**, it's generally recommended to arrive at least **30–60 minutes** before your scheduled appointment time. This allows time for:

- Completing any necessary paperwork
- Checking in and verifying your medical history
- Receiving instructions from the radiology team
- Preparing for the scan (e.g., changing into a gown, IV contrast setup if needed)

**Duration of the Visit**

The actual CT scan typically takes **10–15 minutes**, but the total visit can last **60–90 minutes** due to preparation and post-scan monitoring, especially if contrast dye is used.

If you're having a **coronary CT angiography (CCTA)**, additional time may be needed for:

- **Beta-blockers**: If your heart rate is high, medication may be given to slow it down, which can add **30–60 minutes** to the visit.
- **Post-scan observation**: If contrast is used, you may need to wait for **15–30 minutes** before leaving.

Would you like any preparation tips, such as dietary restrictions or medication instructions?

# 24. I am undergoing heart MRI. Should I arrive earlier than my scheduled time, and how long will the overall visit take?

Yes, for a **cardiac MRI**, you should plan to arrive **at least 30–60 minutes before** your scheduled appointment. This extra time allows for:

- Completing any necessary paperwork.
- Changing into a hospital gown (if required).
- Placing an intravenous (IV) line if contrast (such as gadolinium) is needed.
- A brief consultation with the radiology team.

**Total Visit Duration:**

A **typical cardiac MRI takes 45–90 minutes** depending on:

- Whether contrast is used.
- The complexity of imaging sequences required.
- Your ability to stay still during the scan.

However, including preparation and post-scan observation (if needed), your total time at the facility could range from **1.5 to 3 hours**.

If you have specific questions about your appointment (e.g., fasting requirements or medication adjustments), it's best to check with the imaging center beforehand. Let me know if you need more details!

# 25. I am undergoing heart CT. Will an IV line for contrast dye be necessary, and how is it administered?

Yes, for most **cardiac CT scans**, an **IV line for contrast dye** is necessary. The contrast agent (usually an iodine-based dye) helps enhance the visibility of blood vessels, heart chambers, and coronary arteries, allowing for a more detailed assessment.

**IV Line and Contrast Administration:**

1. **IV Placement:** A small **intravenous (IV) line** will be placed, usually in a vein in your **arm or hand**.
2. **Contrast Injection:** The contrast dye is **injected using an automatic injector**, typically **at a fast rate** (around 3-6 mL/sec) to ensure proper enhancement of the coronary arteries.
3. **Warm Sensation:** You may feel a brief **warm flush** throughout your body when the contrast is injected. This is normal.
4. **Breath-Holding:** You will likely be asked to **hold your breath** for a few seconds during the scan to minimize motion artifacts.
5. **Saline Flush:** After the contrast, a saline flush may be given to clear the IV and improve image quality.

If you have **kidney disease, contrast allergies, or other medical conditions**, inform your doctor beforehand. In some cases, **non-contrast cardiac CT** may be done, such as in coronary artery calcium scoring.

Let me know if you need any more details!

# 26. I am undergoing heart MRI. Will an IV line for contrast dye be necessary, and how is it administered?

Yes, for most **cardiac MRI (CMR) studies**, an **IV line** is required to administer **gadolinium-based contrast dye**. The contrast is used to enhance imaging, especially in studies evaluating **myocardial perfusion, viability (late gadolinium enhancement), fibrosis, or vascular abnormalities**.

**How is the IV Contrast Administered?**

1. **IV Placement**: A healthcare professional will insert a **small intravenous (IV) catheter** into a vein, usually in your **arm or hand**.
2. **Contrast Injection**:
   - The **gadolinium contrast agent** is injected **manually** or using a **power injector**.
   - The injection is typically **a rapid bolus followed by a saline flush**.
3. **Dynamic Imaging**: The contrast circulates through the bloodstream and reaches the heart, helping to highlight **scar tissue, ischemia, or inflammation**.

**Will Contrast Be Necessary for Your Scan?**

- **Contrast is typically used for**:
  - Evaluating **myocardial infarction (scar tissue)**
  - Detecting **fibrosis (e.g., in cardiomyopathies)**
  - Assessing **myocarditis**
  - Studying **perfusion defects (ischemia)**
- **Contrast may not be required for**:
  - **Functional studies** (e.g., looking at cardiac anatomy and motion)
  - Some cases of **congenital heart disease**
  - **Flow imaging (e.g., phase contrast for valvular disease)**

If you have **kidney disease**, your doctor may check kidney function before administering contrast since gadolinium is cleared through the kidneys.

Would you like help understanding any specific aspect of the scan?

# 27. I am undergoing heart CT. Can I drive myself to and from the scan, or do I need someone to accompany me?

Whether you can drive yourself to and from your heart CT scan depends on the type of preparation and medications used during the procedure. Here are some key considerations:

1. **If No Sedation or Beta-Blockers Are Used**
   - If your scan is done without medication that affects your alertness, you should be able to drive yourself without any issues.
2. **If You Are Given Beta-Blockers** (Common for Coronary CT Angiography)
   - Many heart CT scans, particularly **coronary CT angiography (CCTA)**, require beta-blockers to slow your heart rate for better imaging.
   - Beta-blockers can cause dizziness, lightheadedness, or fatigue, which may impair your ability to drive.
   - If you are given beta-blockers, it is recommended that you **arrange for someone to drive you home**.
3. **If You Receive Nitroglycerin**
   - Some heart CT scans use nitroglycerin to dilate blood vessels for better imaging. This can cause low blood pressure, dizziness, or headaches, making it unsafe to drive yourself home.
4. **If Sedation Is Used**
   - If you are given any form of sedation (though not common for standard heart CTs), you **must have someone drive you home**.

**What You Should Do:**

- Check with the imaging center about the medications they plan to use.
- If beta-blockers or nitroglycerin are required, it’s safest to have someone drive you.
- If no medications affecting alertness are given, you can likely drive yourself.

Would you like to check the specific preparation instructions for your scan?

# 28. I am undergoing heart MRI. Can I drive myself to and from the scan, or do I need someone to accompany me?

Whether you can drive yourself to and from your heart MRI depends on the specifics of your scan:

1. **If No Sedation or Contrast Is Used**
   - If your MRI does not involve sedation or contrast (such as gadolinium), you should be able to drive yourself without any issues.
2. **If Gadolinium Contrast Is Used**
   - Gadolinium contrast is commonly used in cardiac MRI to enhance imaging. This does not impair your ability to drive, so you can typically drive yourself home afterward.
3. **If You Receive Sedation**
   - If your MRI requires sedation (e.g., for claustrophobia or difficulty lying still), you **should not** drive. You'll need someone to accompany you or arrange transportation.
4. **If You Are Given a Pharmacologic Stress Test**
   - Some cardiac MRIs involve stress testing with medications like adenosine, regadenoson, or dobutamine. These can cause temporary side effects such as dizziness, nausea, or shortness of breath. If you undergo a stress test, it’s best to have someone drive you home.

**What to Do?**

Check with your radiology center in advance about whether sedation or stress testing will be part of your MRI. If you’re unsure, it’s safest to arrange a ride, especially if you have concerns about dizziness or other temporary effects.

Let me know if you need more details!

# 29. I am undergoing heart CT. Do I need to let my doctor know about allergies?

Yes, you should inform your doctor about any allergies before undergoing a **heart CT scan**, especially if you have a known **iodine or contrast dye allergy**. Many heart CT scans, including **coronary CT angiography (CCTA)**, use **iodinated contrast dye** to enhance the images. If you have a history of **contrast allergy**, your doctor may:

- **Prescribe premedication** (such as steroids and antihistamines) to reduce the risk of an allergic reaction.
- **Consider alternative imaging tests** if necessary.
- **Monitor you more closely** during and after the scan.

Additionally, let your doctor know if you have **asthma, kidney disease, or a history of anaphylaxis**, as these conditions may require special precautions.

Would you like guidance on preparing for your heart CT?

# 30. I am undergoing heart MRI. Do I need to let my doctor know about allergies?

Yes, you should inform your doctor about any allergies before undergoing a **cardiac MRI**, especially if the scan involves contrast material (typically **gadolinium-based contrast agents**). Here’s why:

**1. Contrast Allergy or Previous Reaction**

- If you have had an allergic reaction to gadolinium-based contrast agents (GBCA) in the past, your doctor may take **precautionary measures**, such as prescribing **antihistamines or steroids** before the scan.

**2. Other Allergies (e.g., Food, Drug, or Environmental)**

- While general allergies (like pollen, dust, or food) are **not directly related** to MRI contrast, some individuals with **multiple drug allergies or atopy (asthma, eczema, hay fever)** may have a **higher risk of reaction**.

**3. History of Anaphylaxis**

- If you have a history of severe allergic reactions or anaphylaxis (to any drug, food, or insect stings), your radiology team needs to **be prepared** in case of a reaction.

**4. Kidney Function Concerns**

- If you have **kidney disease** or a history of reduced kidney function, inform your doctor. Some gadolinium-based agents may increase the risk of **nephrogenic systemic fibrosis (NSF)** in patients with severe kidney impairment.

**What You Should Do:**

✔ **Inform your doctor about all allergies**, especially drug allergies.
✔ **Mention any previous reaction to contrast material** (from MRI, CT, or angiography).
✔ **If you have asthma or severe allergies**, discuss whether you need **premedication** before the MRI.
✔ **Check kidney function** (if advised) before getting contrast.

If your MRI is **without contrast**, the risk of an allergic reaction is minimal, but it’s still a good idea to let your doctor know about any allergies.

# 31. What exactly happens during a cardiac CT scan?

A **cardiac CT (computed tomography) scan** is a non-invasive imaging test that uses X-rays to create detailed pictures of the heart and blood vessels. It is often used to assess coronary artery disease, evaluate heart structures, and detect calcium deposits in the coronary arteries.

**Step-by-Step Process of a Cardiac CT Scan:**

**1. Preparation**

- **Fasting:** You may be asked to avoid food and caffeine for a few hours before the scan.
- **Beta-blockers (if needed):** If your heart rate is too high, you may be given beta-blockers to slow it down for better imaging.
- **Nitroglycerin (if needed):** This may be administered to dilate the coronary arteries.
- **Electrocardiogram (ECG) leads:** Small electrodes are attached to your chest to monitor your heart rhythm during the scan.

**2. Positioning**

- You will lie on a narrow table that slides into the CT scanner.
- You may have an IV line inserted into your arm for contrast dye administration.

**3. Contrast Injection (if required)**

- **Iodinated contrast dye** may be injected through an IV to enhance the visibility of coronary arteries and blood flow.
- You may feel a warm sensation or a metallic taste in your mouth for a few seconds.

**4. Image Acquisition**

- The scanner rotates around your chest, taking multiple X-ray images from different angles.
- The ECG synchronizes the images with the heartbeats to minimize motion artifacts.
- You will be asked to hold your breath for a few seconds while images are taken.

**5. Post-Scan**

- If contrast was used, you might need to drink plenty of fluids to flush it out.
- The scan itself is quick (usually 10-15 minutes), but the whole process, including preparation, may take 30-60 minutes.

**Types of Cardiac CT Scans**

- **Coronary Calcium Score (Calcium Scoring CT):** Detects calcium deposits in the coronary arteries, which can indicate early atherosclerosis.
- **CT Coronary Angiography (CTA):** Uses contrast dye to visualize coronary artery blockages.
- **CT for Structural Heart Disease:** Evaluates heart valves, congenital defects, or pericardial disease.
- **Cardiac CT for Planning Procedures:** Used before transcatheter aortic valve replacement (TAVR) or coronary interventions.

Would you like help interpreting cardiac CT findings or understanding its role in a specific condition?

# 32. What exactly happens during a cardiac MRI scan?

A **cardiac MRI (magnetic resonance imaging) scan** is a non-invasive imaging test that provides detailed images of the heart's structure and function using powerful magnets and radio waves. It helps diagnose various heart conditions, such as cardiomyopathies, congenital heart disease, myocardial infarction, and valvular heart disease. Here’s what happens during the scan:

**1. Preparation Before the Scan**

- You may be asked to **change into a hospital gown** and **remove metal objects** (e.g., jewelry, watches, piercings).
- If contrast (usually **gadolinium**) is required, an **IV line** is placed in your arm.
- You will be **positioned on the MRI table** with **electrocardiogram (ECG) leads** placed on your chest to monitor your heart's rhythm.

**2. Entering the MRI Scanner**

- The table slides into the **MRI machine**, which is a large, tube-like structure.
- You will be given **earplugs or headphones** to reduce noise from the machine.
- A **coil** (a special radiofrequency receiver) is placed on your chest to enhance image quality.

**3. During the Scan**

- The machine generates a **strong magnetic field**, which aligns hydrogen atoms in the body.
- **Radiofrequency pulses** send signals to these atoms, and the returning signals are used to create detailed images.
- The scanner makes **loud knocking or thumping noises** as it captures images.
- You will be instructed to **hold your breath for a few seconds** at intervals to minimize motion artifacts.
- If contrast is used, it will be injected **midway through the scan** to enhance blood vessels and tissue differences.

**4. Duration of the Scan**

- A **standard cardiac MRI takes 30 to 90 minutes**, depending on the protocol.
- If additional sequences, stress imaging, or contrast studies are needed, it may take longer.

**5. After the Scan**

- You can **resume normal activities** immediately if no sedation was used.
- If contrast was given, **drinking water** helps clear it from your system.
- The results will be analyzed by a **radiologist or cardiologist**, who will provide a report to your doctor.

Would you like more details on specific aspects, such as contrast use, stress MRI, or common indications?

# 33. I am undergoing heart CT. Do I need to stay completely still or hold my breath at certain times?

Yes, during a **heart CT (cardiac CT)** scan, you will need to follow specific instructions to ensure clear images:

1. **Staying Still:** You should remain as still as possible throughout the scan to prevent motion artifacts, which can blur the images.
2. **Breath Holding:** You will likely be asked to hold your breath for short periods (usually 5–15 seconds) during certain phases of the scan. This helps reduce motion from breathing and heartbeat, leading to sharper images.
3. **Heart Rate Control:** If your heart rate is too high or irregular, you may be given beta-blockers before the scan to slow it down for better image quality.
4. **Contrast Injection:** If you are receiving contrast dye (for **CT coronary angiography**), you may feel a warm sensation or a metallic taste, but you should continue to stay still and follow breath-hold instructions.

Make sure to follow the technologist’s guidance, and let them know if you have any issues holding your breath or staying still.

# 34. I am undergoing heart MRI. Do I need to stay completely still or hold my breath at certain times?

Yes, during a **cardiac MRI**, you will need to stay **completely still** for the entire scan to ensure clear images. Additionally, you will likely be asked to **hold your breath** at specific times, usually for short intervals (about **10-20 seconds**) while certain images are being taken.

Here’s what to expect:

1. **Staying Still** – Any movement can blur the images, so it's important to lie still throughout the scan.
2. **Breath-Holding** – You may be instructed to hold your breath multiple times when imaging the heart and blood vessels. The technician will guide you through this using a speaker inside the MRI machine.
3. **Noise & Comfort** – The MRI machine can be loud, producing knocking or buzzing sounds. You will be given earplugs or headphones.
4. **Contrast Injection (if needed)** – Some cardiac MRIs require a contrast agent (like gadolinium) to enhance the images. This is usually injected through an IV.

If you have any concerns about breath-holding or staying still for long periods, let the MRI technologist know before the scan. They can often adjust the imaging technique to accommodate your comfort.

Would you like any additional tips to prepare for your MRI?

# 35. I am undergoing heart CT. Is the procedure painful or uncomfortable in any way?

A **heart CT scan** (also called a **coronary CT angiography or cardiac CT**) is generally a **painless and non-invasive** procedure. However, there are some aspects that might cause **mild discomfort**:

1. **IV Contrast Injection**
   - If contrast dye is used, it will be injected through an **IV line**, usually in your arm.
   - This can cause a **warm or flushing sensation** throughout your body, which is normal and temporary.
   - Some people may experience a **metallic taste** in their mouth for a few minutes.
2. **Lying Still**
   - You will need to lie **completely still** on the scanner table for a few minutes, which might feel uncomfortable if you have back pain or difficulty staying in one position.
3. **Breath-Holding**
   - The technician will ask you to **hold your breath** for a few seconds while images are taken. This is important to prevent motion artifacts that can blur the images.
4. **Beta-Blockers (If Given)**
   - If your heart rate is too fast, you might receive a **beta-blocker** to slow it down. This can make you feel a bit tired or dizzy.
   - Some people may also receive **nitroglycerin** under the tongue to dilate the coronary arteries, which may cause a mild headache.
5. **Radiation Exposure**
   - Although the radiation dose is **low and generally safe**, it’s something to be aware of, especially if you need multiple scans over time.

**Should You Expect Pain?**

❌ **No significant pain** is expected during the procedure. The most uncomfortable parts may be the **IV placement** and the **warm sensation** from the contrast dye.

**Any Risks?**

- **Allergic reactions to contrast dye** (rare, but let your doctor know if you have allergies).
- **Mild nausea or dizziness** in some cases.
- **Kidney function concerns** in patients with pre-existing kidney disease (contrast dye can affect kidney function in rare cases).

**Final Thought**

A heart CT is a **quick, safe, and well-tolerated procedure** for most people. If you have any concerns about the **contrast dye** or **medications used**, let your doctor or the radiology team know beforehand.

Let me know if you need any **preparation tips** for your scan! 😊

# 36. I am undergoing heart MRI. Is the procedure painful or uncomfortable in any way?

A **heart MRI (cardiac MRI)** is a non-invasive imaging procedure that provides detailed images of your heart and blood vessels. While it is **not painful**, some aspects of the procedure might cause mild discomfort:

**Potential Discomforts:**

1. **Lying Still for a Long Time:** The scan can take **30–90 minutes**, requiring you to stay still, which may cause stiffness.
2. **Enclosed Space (Claustrophobia):** If you are **claustrophobic**, lying in the MRI machine might feel uncomfortable. Some centers offer **open MRI** or mild sedation if needed.
3. **Loud Noises:** The machine makes loud knocking and humming sounds, but you'll be given **earplugs or headphones** to minimize the noise.
4. **Breath-Holding:** You may be asked to **hold your breath** for short periods (10–20 seconds at a time) to improve image quality.
5. **Contrast Injection (Gadolinium-Based):** If contrast is used:
   - It is injected through an **IV in your arm**.
   - Most people tolerate it well, but some might feel a **cool sensation** or mild discomfort.
   - Rarely, it can cause nausea or an allergic reaction.

**Tips to Make It Easier:**

✔ Wear comfortable, metal-free clothing.
✔ Close your eyes and practice deep breathing if feeling anxious.
✔ If you are **claustrophobic**, inform your doctor beforehand.

Would you like me to explain anything in more detail?

# 37. I am undergoing heart CT. Can I listen to music or communicate with the technician during the scan?

During a **heart CT scan**, whether you can listen to music or communicate with the technician depends on the type of scan being performed and the facility’s policies:

1. **Listening to Music**:
   - Some facilities may allow you to wear headphones or play soft music in the background to help you relax, especially if you feel anxious.
   - However, during **coronary CT angiography (CCTA)**, where heart rate control is crucial, they may not allow music to prevent distractions and ensure you follow breathing instructions properly.
2. **Communication with the Technician**:
   - You will **be able to communicate with the technician** via an intercom system before and after the scan.
   - During the scan, the technician may **give you instructions**, such as when to hold your breath or stay still.
   - If you have any discomfort, most CT scanners have an emergency button or microphone system to alert the technician.

Since your **heart CT** may require **breath-holding** and precise timing, distractions like music might not always be allowed. It’s best to ask the staff at your facility beforehand.

# 38. I am undergoing heart MRI. Can I listen to music or communicate with the technician during the scan?

Yes, during a **heart MRI**, you may have some options for listening to music and communicating with the technician, but it depends on the facility and the specific protocol for your scan. Here’s what you can expect:

**Listening to Music:**

- Many MRI centers **offer headphones or earplugs** that allow you to listen to music during the scan.
- However, because cardiac MRI involves **breath-holding instructions**, some facilities may limit music to ensure you can clearly hear the technician’s commands.
- If music is allowed, it is typically **played through an MRI-compatible audio system** or specialized headphones that reduce noise from the scanner.

**Communicating with the Technician:**

- **Intercom System:** You will be able to talk to the MRI technician through an intercom. They will guide you through breath-holding sequences and let you know how long each scan will take.
- **Emergency Button:** Most MRI scanners provide a **call button** that you can press if you feel uncomfortable or need assistance.
- **Visual Monitoring:** The technician will be watching you from the control room and can respond if needed.

**Special Considerations for Cardiac MRI:**

- Since cardiac MRI often requires you to **hold your breath multiple times**, the technician will communicate with you frequently.
- Loud scanner noises may make it difficult to hear, so clear and simple instructions are given before each sequence.
- If contrast (gadolinium) is used, the technician might check in with you after injection.

**Tip:** If you have concerns about music or communication, ask the MRI technologist before the scan starts—they can explain what’s possible based on the equipment they use.

# 39. I am undergoing heart CT. What if I start feeling anxious or need to stop the test?

If you start feeling anxious or need to stop your **heart CT (coronary CT angiography or calcium scoring CT)**, here’s what you should do:

1. **Communicate Immediately** – Let the technician know if you feel anxious, uncomfortable, or need a break. Most imaging centers have a way for you to signal them (such as a button or microphone inside the scanner).
2. **Deep Breathing & Relaxation** – Try slow, deep breathing to help calm yourself. If possible, close your eyes and focus on steady, rhythmic breathing.
3. **Medication Option** – If you have a history of **severe anxiety** during medical tests, you can ask your doctor beforehand about taking a mild sedative (like **lorazepam or diazepam**) before the scan.
4. **Breaks are Possible** – If necessary, the scan can be **paused briefly** to allow you to calm down.
5. **IV Contrast Consideration** – If you’re receiving **contrast dye**, you might feel a temporary warm sensation or a metallic taste. This is normal, but if you feel unwell (e.g., nausea, difficulty breathing), inform the staff immediately.
6. **Know the Test is Quick** – Most heart CT scans **only take a few minutes** (actual scanning may be under a minute), which can be reassuring.

Would you like any tips for preparation before the scan?

# 40. I am undergoing heart MRI. What if I start feeling anxious or need to stop the test?

If you start feeling anxious or need to stop the heart MRI, here’s what you can do:

**1. Use the Emergency Call Button**

- Most MRI machines have a **call button or squeeze ball** that you can press if you feel uncomfortable.
- The MRI technician will stop the scan and communicate with you.

**2. Communicate with the MRI Technologist**

- You can speak with the technician through an intercom.
- If you feel overwhelmed, let them know so they can assist you.

**3. Request a Break if Needed**

- If necessary, the scan can be paused so you can take a moment to relax.
- Some MRI sequences take a few minutes, so it’s best to complete one before stopping.

**4. Try to Stay Calm and Use Breathing Techniques**

- Deep breathing and mindfulness techniques can help you relax.
- Closing your eyes and imagining a peaceful place may reduce anxiety.

**5. Ask About Sedation if You're Extremely Claustrophobic**

- If you’re severely anxious, you can discuss **mild sedation options** with your doctor before the test.
- Some facilities also offer **open MRI machines**, which feel less confining.

**6. Bring Earplugs or Listen to Music**

- MRI machines make loud noises, which can be stressful.
- Many MRI centers provide **earplugs or headphones** with calming music.

**7. Know That You Are Safe**

- MRI scans are non-invasive and supervised.
- The staff is trained to handle anxiety and will help you feel comfortable.

If you anticipate anxiety before the scan, let your doctor or the MRI staff know in advance so they can accommodate your needs. Would you like some tips on preparing for the test?

# 41. I am undergoing Heart CT. Is there any special monitoring or sedation used during this scan?

For a **Heart CT scan (Cardiac CT)**, special monitoring and sedation depend on the type of scan and the patient's condition. Here’s what you can expect:

**1. Monitoring During the Scan:**

- **Heart Rate Monitoring:** Since a cardiac CT, especially **coronary CT angiography (CCTA)**, requires clear images of the coronary arteries, your heart rate may need to be **below 60–70 beats per minute (bpm)** for optimal image quality.
- **Electrocardiogram (ECG) Leads:** Small electrodes may be placed on your chest to monitor your heart rhythm and ensure the scan is timed correctly with your heartbeat.
- **Blood Pressure Monitoring:** If medications are used, your blood pressure may be checked before and after the scan.

**2. Medications That May Be Used:**

- **Beta-Blockers:** If your heart rate is too high, the radiologist may give a **beta-blocker** (e.g., metoprolol) to slow it down for better imaging.
- **Nitroglycerin (Optional):** Some protocols require **sublingual nitroglycerin** to dilate coronary arteries and improve visualization.
- **Sedation (Rarely Needed):** Most patients do not require sedation. However, if you have **claustrophobia or severe anxiety**, mild sedation may be considered.

**3. Use of Contrast Dye:**

- If you are undergoing a **CT coronary angiography (CCTA)** or **cardiac CT with contrast**, you will receive an **iodinated contrast injection** through an IV in your arm.
- You may feel a warm sensation and a metallic taste, which is normal.

**4. Fasting Requirements:**

- You may be asked to **fast for 4–6 hours** before the scan, especially if contrast is used.
- **Caffeine should be avoided** before the scan, as it can increase heart rate.

Let me know if you need more details or have any concerns about your Heart CT! 🚑💙

# 42. I am undergoing Heart MRI. Is there any special monitoring or sedation used during this scan?

A **cardiac MRI (Heart MRI)** is typically a safe and non-invasive imaging study, but certain monitoring or sedation measures may be required depending on the type of scan and your specific condition. Here’s what you can expect:

**Monitoring During a Cardiac MRI**

- **Heart Rate and ECG Monitoring**: Since cardiac MRI sequences are often synchronized with your heartbeat, small ECG leads (electrodes) will be placed on your chest to track your heart’s electrical activity.
- **Breathing Instructions**: You may be asked to hold your breath briefly during certain parts of the scan.
- **Oxygen Saturation and Blood Pressure**: If you have any underlying conditions, these parameters may be monitored.
- **Contrast Injection (Gadolinium-based Contrast Agent)**: If your scan requires contrast to evaluate the heart muscle or vessels, an IV line will be inserted for administration.

**Sedation and Special Cases**

- **Routine Cardiac MRI**: Most patients do **not** need sedation and remain awake throughout the scan.
- **Claustrophobic Patients**: If you feel anxious or claustrophobic inside the MRI machine, mild sedation (such as a benzodiazepine) may be given beforehand.
- **Pediatric Patients or Those with Severe Anxiety**: In some cases, deeper sedation or even anesthesia may be used to keep the patient still.
- **Stress Cardiac MRI**: If you're undergoing a **stress MRI** to assess myocardial perfusion, a medication (such as **adenosine, regadenoson, or dobutamine**) may be administered to simulate the effects of exercise on your heart.

**Precautions**

- If you have any metallic implants, pacemakers, or defibrillators, inform your doctor and MRI technician, as certain devices may not be MRI-compatible.
- If you are **pregnant or have kidney disease**, discuss the risks of contrast agents with your physician.

Would you like specific preparation tips for your cardiac MRI?

# 43. I am undergoing heart CT. Will I need to stay for observation once the scan is done?

In most cases, you will not need to stay for observation after a heart CT (cardiac CT) scan. However, there are a few exceptions depending on the type of scan and any medications or contrast agents used:

1. **If No Contrast is Used:**
   - If your cardiac CT does not require contrast (e.g., a coronary artery calcium scan), you can leave immediately after the scan.
2. **If Contrast is Used (CT Angiography - CTA):**
   - Most cardiac CTs involve an iodine-based contrast agent injected into a vein to visualize the coronary arteries.
   - If contrast is used, you might be monitored for **15–30 minutes** to ensure there are no allergic reactions or adverse effects.
   - Drinking plenty of water afterward is recommended to help flush the contrast from your system.
3. **If Beta-Blockers or Nitroglycerin Were Given:**
   - If your heart rate needed to be slowed down for better imaging, you may have been given a **beta-blocker** or **nitroglycerin** before the scan.
   - Some facilities may monitor you for a short period to ensure there are no side effects (e.g., dizziness, low blood pressure).
4. **If You Have Kidney Disease or Are at Risk of Contrast-Induced Nephropathy:**
   - If you have kidney issues, some facilities may observe you briefly and advise you to stay hydrated to help prevent contrast-induced kidney problems.

**Key Takeaways:**

- If no contrast is used, you can leave immediately.
- If contrast is used, a short observation period (15–30 minutes) may be required.
- If medications (beta-blockers/nitroglycerin) are given, brief monitoring might be needed.
- If you have kidney disease or a history of contrast allergy, additional precautions may apply.

Let me know if you have any specific concerns about your upcoming scan!

# 44. I am undergoing heart MRI. Will I need to stay for observation once the scan is done?

In most cases, after a **heart MRI** (cardiac MRI), you will not need to stay for observation and can go home immediately unless you received **sedation** or **contrast agents (gadolinium-based contrast dye)** that require monitoring for a short period. Here’s what to expect:

**1. If No Contrast or Sedation Was Used:**

- You can leave right after the scan.
- There are no special precautions or recovery time needed.

**2. If You Received Gadolinium Contrast:**

- Most people can leave right away, but some centers may ask you to stay for **15-30 minutes** to monitor for **allergic reactions** (rare).
- If you have **kidney disease**, they may take extra precautions due to the small risk of **nephrogenic systemic fibrosis (NSF)**.

**3. If You Were Given Sedation:**

- If you were sedated due to **claustrophobia or anxiety**, you might need to stay until you are fully awake.
- You should not drive after sedation, so arrange for someone to take you home.

**4. If You Have a Medical Condition That Requires Monitoring:**

- If you have **a history of severe heart disease, arrhythmias, or a pacemaker/ICD (which requires special MRI protocols)**, the medical team may monitor you briefly.

**Final Note:** Since you are doing a meta-analysis on MRI-derived **ECV and T1 mapping**, you may already be familiar with some of these details! If your MRI includes **stress imaging** (e.g., with adenosine or dobutamine), they might observe you longer. Always follow your radiologist's or cardiologist's instructions.

Would you like any advice on interpreting your MRI results afterward?

# 45. I am undergoing Heart CT. Can I drive myself home right afterward?

Whether you can drive yourself home after a **Heart CT (Cardiac CT)** depends on whether **contrast dye** is used and whether you are given **sedation**.

**Key Considerations:**

1. **Without Contrast or Sedation:**
   - If your Heart CT is a **non-contrast scan**, you can **drive yourself home** immediately.
   - You will have no side effects that impair driving.
2. **With Contrast (IV Iodine-based Contrast Dye):**
   - Most patients **can still drive** afterward.
   - However, if you have a history of **contrast allergy** or feel **lightheaded, nauseous, or unwell**, it’s safer to have someone drive you.
3. **With Sedation (Rare for Heart CT):**
   - If you are given **sedatives (e.g., beta-blockers, benzodiazepines)** to slow your heart rate for better imaging, you **should not drive** afterward.
   - In this case, arrange for someone to drive you home.

**Final Advice:**

- **Check with your radiology center** if you’re unsure whether sedation will be used.
- **If you feel completely normal**, you can likely drive yourself.
- **If you feel drowsy or unwell**, wait until you feel better or arrange for a ride.

Would you like me to help you prepare for the scan in any other way?

# 46. I am undergoing Heart MRI. Can I drive myself home right afterward?

In most cases, you **can drive yourself home after a cardiac MRI** if no sedatives or contrast agents that cause drowsiness are used. However, there are a few important factors to consider:

**You Can Drive If:**

- No **sedation** was administered.
- You feel well after the procedure.
- You are not experiencing dizziness, nausea, or other discomfort.

**You Should Not Drive If:**

- **Sedation** (such as benzodiazepines) was used to help you relax during the MRI. This can impair judgment and reflexes.
- You feel **lightheaded, dizzy, or unwell** after the scan.
- You experience any **reaction to the contrast agent** (such as nausea, dizziness, or an allergic reaction).
- Your doctor advises against it based on your medical condition.

**If You Received Gadolinium Contrast:**

- Most people **do not experience side effects** from gadolinium contrast.
- However, some may feel **mild nausea, dizziness, or a metallic taste** for a short time.
- If you feel fine, driving is usually **not restricted**.

**Bottom Line:**

If your MRI is **without sedation**, you should be able to drive yourself home. However, if you feel unwell or were given a sedative, it’s best to arrange for someone to drive you.

# 47. I am undergoing heart CT. Will I feel any immediate side effects like nausea or dizziness?

A **heart CT scan** (coronary CT angiography or calcium scoring CT) is generally safe and well-tolerated. However, depending on the type of scan and whether contrast dye (iodine-based) is used, you might experience some temporary side effects:

**Possible Immediate Side Effects:**

1. **With Contrast Dye:**
   - **Warm sensation:** A feeling of warmth or flushing throughout your body for a few seconds.
   - **Metallic taste:** A temporary metallic or salty taste in your mouth.
   - **Nausea or dizziness:** Some people experience mild nausea or dizziness, but it usually passes quickly.
   - **Allergic reaction:** Rarely, people may develop mild itching, hives, or more severe allergic reactions.
   - **Injection site discomfort:** Mild pain or bruising at the IV site.
2. **Without Contrast Dye:**
   - **No major side effects** are expected since no contrast is injected.
3. **Effects of Beta-Blockers (if given to slow heart rate for better imaging):**
   - **Mild dizziness or fatigue** due to a temporary drop in blood pressure or heart rate.
   - **Cold hands or feet** in some cases.

**What to Do?**

- If you feel **nausea or dizziness**, lying down for a few minutes should help.
- If you develop **itching, swelling, or breathing difficulty**, inform the medical team immediately.
- Stay **hydrated** to help your body flush out the contrast dye.

Most people tolerate heart CT scans without issues, and any mild side effects typically resolve quickly. Let me know if you need any specific guidance!

# 48. I am undergoing heart MRI. Will I feel any immediate side effects like nausea or dizziness?

A **heart MRI (cardiac MRI)** is generally a safe and well-tolerated procedure. However, depending on certain factors, you may experience mild side effects:

**Possible Immediate Side Effects:**

1. **From the MRI Itself:**
   - Most patients feel **no discomfort** during the scan.
   - Some may experience **mild dizziness** or a **floating sensation** due to the loud noises and magnetic field.
   - If you have **claustrophobia**, you might feel anxious inside the machine.
2. **From the Contrast Agent (if used):**
   - If your MRI includes **gadolinium contrast**, mild side effects may occur:
     - **Warm sensation** in the body (normal and temporary).
     - **Nausea** (rare and usually mild).
     - **Dizziness** or **lightheadedness** (uncommon).
     - **Allergic reactions** (very rare, but can include itching, rash, or breathing difficulty).
3. **Lying Still for a Long Time:**
   - The scan can take **30–90 minutes**, so you might feel a bit stiff or uncomfortable afterward.

**How to Minimize Discomfort:**

- **Eat a light meal** before the scan to reduce nausea risk.
- **Stay hydrated**, especially if contrast is used, to help flush it from your system.
- **Tell the technician** if you feel uncomfortable—they can pause or adjust the procedure.

**When to Seek Help:**

- If you experience **severe nausea, vomiting, or breathing difficulty** after the scan, inform your doctor immediately.

Would you like any tips on preparing for your cardiac MRI?

# 49. I did heart CT, for which I received contrast. Do I need to drink extra fluids or take other precautions?

Yes, after receiving contrast for your **cardiac CT**, it is generally recommended to take the following precautions:

**1. Hydration (Most Important)**

- Drink **plenty of fluids** (water is best) to help flush the contrast dye from your system through your kidneys.
- Aim for **at least 2–3 liters** of water over the next 24 hours, unless you have **heart failure, kidney disease, or another condition** where fluid intake needs to be limited.

**2. Monitor for Allergic Reactions**

- Contrast reactions are rare but possible. Seek medical attention if you experience:
  - **Hives, rash, or itching**
  - **Difficulty breathing or swelling of the face, lips, or throat**
  - **Severe nausea or vomiting**
  - **Dizziness or fainting**

**3. Kidney Function (Especially If You Have Risk Factors)**

- If you have **chronic kidney disease (CKD), diabetes, or dehydration**, your kidneys may have difficulty clearing the contrast.
- **Signs of kidney issues** include:
  - Decreased urine output
  - Swelling in legs or face
  - Fatigue
- If you are at risk, your doctor may have ordered a **creatinine test** before or after the scan.

**4. Avoid Nephrotoxic Substances**

- Avoid **NSAIDs (e.g., ibuprofen, naproxen, diclofenac)** for 24–48 hours as they can stress your kidneys.
- Limit **alcohol and caffeine**, as they can contribute to dehydration.

**5. Diabetic Considerations (Metformin Users)**

- If you are on **metformin**, it’s sometimes advised to **pause it for 48 hours** if you have kidney impairment. Your doctor should have given you specific instructions.

**6. Listen to Your Body**

- If you feel **unusually fatigued, have persistent nausea, or reduced urination**, consult your doctor.

For **healthy individuals without kidney issues**, drinking extra water is usually sufficient. Let me know if you have any specific health concerns!

# 50. I did heart MRI, for which I received contrast. Do I need to drink extra fluids or take other precautions?

Since you received **contrast** for your **heart MRI**, it's likely that the contrast agent was **gadolinium-based**. Here are some general recommendations for after the procedure:

**1. Drink Plenty of Fluids**

- Gadolinium is cleared through your **kidneys**, so drinking **extra water** helps flush it out more quickly.
- Aim for at least **2–3 liters (8–12 cups) of water** over the next 24 hours, unless you have a medical condition that limits fluid intake (e.g., heart failure, kidney disease).

**2. Monitor for Side Effects**

- Most people tolerate gadolinium well, but mild side effects may include:
  - Nausea or headache (usually short-lived)
  - A metallic taste in the mouth
  - Mild dizziness
- If you experience **severe symptoms** (e.g., difficulty breathing, swelling, rash, or itching), seek medical attention immediately.

**3. Kidney Function Considerations**

- If you have **kidney disease** or a history of impaired kidney function, gadolinium may not clear as quickly.
- If you were asked to get a **blood test (e.g., creatinine, eGFR) before the MRI**, it’s a sign that your doctor wanted to ensure your kidneys can process the contrast safely.
- If you have **advanced kidney disease**, discuss with your doctor whether follow-up monitoring is needed.

**4. Avoid Alcohol and Excess Caffeine**

- Alcohol and caffeine can be **dehydrating**, which might slow down contrast elimination.

**5. Resume Normal Activities**

- Unless instructed otherwise, you can **eat, exercise, and go about your daily routine** as usual.

Would you like me to check any specific concerns based on your health status?

# 51. I did heart CT, for which I received contrast. How will my body get rid of the contrast?

The contrast agent used in your heart CT scan is likely an **iodinated contrast medium**, which is commonly used for CT imaging. Your body will eliminate the contrast primarily through your **kidneys**, and it will be excreted in your urine.

**How Your Body Clears the Contrast**

1. **Distribution** – After injection, the contrast circulates through your bloodstream and enhances the visibility of heart structures in the CT scan.
2. **Filtration by the Kidneys** – The contrast is **not metabolized** by your liver but is instead filtered out by your kidneys.
3. **Excretion in Urine** – Within a few hours, most of the contrast is eliminated through urination. Typically, **90% of the contrast is excreted within 24 hours** in individuals with normal kidney function.

**Factors That Influence Elimination**

- **Kidney Function** – If you have normal kidney function, contrast clearance is usually fast and uneventful.
- **Hydration** – Drinking plenty of fluids (especially **water**) can help speed up the excretion process and reduce any risk of kidney strain.
- **Pre-existing Kidney Disease** – If you have kidney impairment (e.g., chronic kidney disease), contrast clearance may take longer, and your doctor may monitor your kidney function.

**How to Help Your Body Eliminate the Contrast**

- **Drink Plenty of Water** – This helps flush the contrast out faster.
- **Monitor for Symptoms** – In rare cases, contrast may cause mild reactions like nausea, flushing, or allergic responses. Severe reactions (e.g., contrast-induced nephropathy) are uncommon.
- **Follow-Up if Needed** – If you have a history of kidney disease or diabetes, your doctor may recommend a blood test (serum creatinine) to check kidney function after the scan.

If you have normal kidney function, your body should eliminate the contrast completely within **24 to 48 hours** without any issues. Let me know if you have concerns!

# 52. I did heart MRI, for which I received contrast. How will my body get rid of the contrast?

The contrast agent used in your heart MRI was likely a **gadolinium-based contrast agent (GBCA)**. Gadolinium is a heavy metal, but in contrast agents, it is bound to a chelating molecule that helps prevent toxicity and facilitates its elimination from the body.

**How Your Body Eliminates the Contrast**

1. **Kidney Filtration & Urine Excretion**
   - The majority of the gadolinium contrast is **filtered by your kidneys** and **excreted in the urine** within **24 hours** if you have normal kidney function.
   - More than **90%** of the contrast is typically removed within the **first 6-12 hours**.
2. **Minimal Elimination Through Bile & Feces**
   - A very small amount may be eliminated through the **liver and bile**, but this is negligible in most cases.

**How to Help Your Body Clear the Contrast Faster**

- **Stay well-hydrated**: Drink plenty of water to support kidney function and speed up excretion.
- **Monitor your urine output**: If you have normal urination patterns, this indicates your kidneys are effectively clearing the contrast.
- **Avoid nephrotoxic substances**: Such as NSAIDs (e.g., ibuprofen), which can strain the kidneys.

**Are There Any Risks?**

For most people with **normal kidney function**, gadolinium contrast is cleared safely and does not cause issues. However, if you have **kidney disease**, contrast clearance can be delayed, and there is a small risk of a rare condition called **nephrogenic systemic fibrosis (NSF)**. Since you were given contrast, the radiology team likely confirmed that your kidneys are functioning normally before administration.

**When Should You Be Concerned?**

- If you have **reduced urine output** for more than a day.
- If you experience **persistent nausea, swelling, or skin changes**.
- If you have **known kidney disease**, let your doctor know if you feel unwell after the scan.

Let me know if you need any more clarification!

# 53. I am undergoing heart CT. Will I experience any pain or soreness following the procedure?

A **heart CT scan (cardiac CT)** is a **non-invasive imaging test** that uses X-rays to create detailed images of your heart and blood vessels. It is typically performed to assess coronary artery disease, calcium buildup, or heart structure abnormalities.

**Pain or Soreness After the Procedure?**

- **Minimal to No Pain**: The procedure itself is **painless**. You will lie on a table while the CT scanner takes images.
- **IV Contrast Discomfort (if used)**: If contrast dye is injected, you might feel a **warm sensation** or a **metallic taste** in your mouth for a few seconds.
- **Injection Site Soreness**: If you received IV contrast, you might have **mild soreness or bruising** at the IV insertion site, but it usually resolves quickly.
- **Beta-Blockers or Nitroglycerin (if given)**:
  - If you were given a beta-blocker to slow your heart rate, you might feel a bit **fatigued** or **dizzy**.
  - If **nitroglycerin** was used to dilate your coronary arteries, you might experience a **mild headache**.

**When to Seek Medical Attention?**

If you experience any of the following after the scan, contact your doctor:

- **Persistent pain or swelling at the IV site**
- **Severe allergic reaction** (rash, swelling, difficulty breathing)
- **Dizziness or fainting** that doesn’t improve

**Key Takeaway**

Most people **do not experience any pain or soreness** after a heart CT scan. If you do, it is usually mild and temporary. Let me know if you need further details!

# 54. I am undergoing heart MRI. Will I experience any pain or soreness following the procedure?

A heart MRI (cardiac MRI) is a non-invasive imaging test that uses a strong magnetic field and radio waves to create detailed images of the heart. It is generally painless and does not cause soreness.

However, you might experience the following:

1. **During the Procedure:**
   - You will need to lie still inside the MRI scanner for 30–90 minutes.
   - The machine can be noisy, but earplugs or headphones are usually provided.
   - If contrast dye (gadolinium) is used, you may feel a cold sensation when it is injected.
   - Some people experience mild discomfort from lying still for an extended period.
2. **After the Procedure:**
   - No pain or soreness is expected.
   - If contrast was used, you might have mild nausea or a metallic taste, but this is temporary.
   - If an IV was placed for contrast injection, you may have mild bruising or soreness at the injection site.

If you have any specific medical conditions (e.g., kidney issues or allergies to contrast dye), let your doctor know in advance. Otherwise, recovery is immediate, and you can resume normal activities right after the scan.

# 55. I did heart CT. How soon can I return to my normal activities, such as work or exercise?

If you had a **routine heart CT scan (coronary calcium scoring or coronary CT angiography) with contrast**, you can typically return to normal activities **immediately** after the scan. However, here are a few considerations:

**Returning to Normal Activities After a Heart CT:**

1. **Work:**
   - If your job does not require heavy physical activity, you can return **immediately** after the scan.
   - If sedation was used (rare), you may need a few hours to recover.
2. **Exercise:**
   - If **contrast (iodine-based dye) was used**, drink plenty of water to flush it out.
   - Light exercise can be resumed the **same day** unless advised otherwise.
   - If you had sedation (such as in rare cases for claustrophobia), avoid strenuous exercise for **24 hours**.
3. **Diet & Hydration:**
   - No specific restrictions.
   - If contrast was used, stay hydrated to help your kidneys clear the dye.
4. **Possible Mild Side Effects:**
   - **Contrast-related:** Some may experience mild nausea, warmth, or a metallic taste briefly.
   - **IV site discomfort:** If you had an IV, mild bruising or soreness is normal.
   - **Allergic reaction (rare):** If you develop itching, rash, or breathing difficulty, seek medical help.

**When to Delay Activities?**

- If you feel lightheaded, unwell, or had sedation, take it easy for the rest of the day.
- If you had a **CT-guided procedure (e.g., biopsy, angioplasty planning)**, follow your doctor’s specific recommendations.

If your CT was just for imaging, you should be fine to resume all activities the same day! Let me know if you had a specific concern.

# 56. I did heart MRI. How soon can I return to my normal activities, such as work or exercise?

The time it takes to return to normal activities after a **heart MRI (cardiac MRI)** depends on whether you received **contrast dye (gadolinium-based contrast agent)** and whether you were **sedated** during the scan. Here’s what you need to know:

**1. If No Contrast and No Sedation:**

- You can **resume normal activities immediately**, including **work and exercise**.

**2. If You Received Contrast (Gadolinium):**

- You can still resume normal activities right away, but it's recommended to:
  - **Drink plenty of water** to help flush the contrast out of your system.
  - If you have **kidney disease**, your doctor may give specific instructions regarding contrast clearance.

**3. If You Were Sedated:**

- You may need **a few hours** to fully recover from sedation effects.
- Avoid driving, operating heavy machinery, or making important decisions for the rest of the day.

**4. If You Had a Stress Cardiac MRI:**

- If your MRI included a **pharmacologic stress test** (using medications like adenosine or dobutamine), you may feel temporary side effects such as:
  - Flushing
  - Mild nausea
  - Dizziness
  - Chest discomfort (rare)

These effects typically **resolve within an hour**, but you might feel tired for a few hours. If you feel fine, you can **return to normal activities the same day**.

**When to Be Cautious:**

- If you experience **persistent dizziness, nausea, or allergic reactions** (rash, difficulty breathing) after contrast injection, contact a healthcare provider.

If your doctor hasn’t advised any restrictions, you should be good to go! Let me know if you had a specific concern related to the MRI.

# 57. I did heart CT. Are there any dietary restrictions after the scan?

After a **heart CT scan** (coronary CT angiography or calcium scoring), there are usually **no specific dietary restrictions** unless you received **contrast dye (iodine-based contrast).** Here’s what to keep in mind:

**If No Contrast Was Used:**

- You can **eat and drink as usual** immediately after the scan.

**If Contrast Was Used:**

- **Hydrate well**: Drink plenty of water to help flush the contrast dye from your kidneys.
- **Avoid excessive caffeine**: If you were given a beta-blocker to slow your heart rate for the scan, it’s best to **limit caffeine** for the rest of the day.
- **Watch for allergic reactions**: Rarely, some people may experience nausea, itching, or a rash from the contrast. Seek medical attention if you have severe symptoms.
- **If you have kidney issues**: Your doctor may recommend avoiding high-protein foods and alcohol for a day to reduce strain on your kidneys.

If you were **instructed to fast** before the scan, you can resume eating normally afterward unless advised otherwise. Let me know if you had any specific concerns!

# 58. I did heart MRI. Are there any dietary restrictions after the scan?

For a standard **heart MRI (cardiac MRI)** without contrast, there are **no dietary restrictions** after the scan. You can eat and drink as usual.

However, if your scan included a **contrast agent** (such as gadolinium-based contrast), it's recommended to:

1. **Stay well-hydrated** – Drink plenty of water to help flush the contrast out of your system.
2. **Limit alcohol and caffeine** if they cause dehydration.
3. **If you have kidney issues**, follow your doctor’s guidance, as some people with kidney disease may need additional precautions.

Did your MRI include contrast, or do you have any specific concerns about the scan?

# 59. I did heart CT. Do I need a follow-up visit or phone call to discuss the results?

Whether you need a follow-up visit or phone call depends on a few factors:

1. **Were you told to follow up?** If your doctor or imaging center advised a follow-up, then you should schedule one.
2. **Were results provided to you directly?** If you received the report and understand the findings, you may not need a follow-up unless there are abnormalities that require discussion.
3. **Did the report mention any significant findings?** If your heart CT showed anything concerning (e.g., coronary artery disease, calcium buildup, or other abnormalities), you should follow up with your doctor.
4. **Routine screening vs. diagnostic test:** If this was a routine calcium score test and the results were normal, you may not need a follow-up. If it was done to investigate symptoms (e.g., chest pain, shortness of breath), follow-up is advisable.
5. **Symptoms or concerns:** If you have ongoing symptoms, questions, or concerns about your heart health, a follow-up visit or phone call is recommended.

If you’re unsure, you can call your doctor’s office and ask if a follow-up is necessary. Let me know if you need help interpreting your report!

# 60. I did heart CT. What symptoms after the test should prompt me to call my doctor?

After a **heart CT (coronary CT angiography or calcium scoring CT scan)**, most people do not experience any significant side effects. However, you should contact your doctor if you experience any of the following:

**1. Signs of an Allergic Reaction (if contrast was used)**

- **Hives, rash, or itching**
- **Swelling of the face, lips, tongue, or throat**
- **Difficulty breathing or wheezing**
- **Dizziness or fainting**
- **Severe nausea or vomiting**

**2. Kidney Problems (if contrast was used)**

- **Reduced urination or dark urine**
- **Swelling in the legs or feet**
- **Fatigue or confusion**
- **Shortness of breath**

**3. Signs of a Blood Clot (rare)**

- **Sudden chest pain or pressure**
- **Shortness of breath**
- **Pain or swelling in one leg**
- **Coughing up blood**

**4. Persistent or Worsening Symptoms**

- **Severe headache or dizziness**
- **Unusual weakness or fatigue**
- **Persistent nausea or vomiting**
- **Pain, redness, or swelling at the IV site (possible infection or phlebitis)**

**5. Low Blood Pressure Symptoms (if contrast reaction occurred)**

- **Extreme dizziness**
- **Blurred vision**
- **Fainting or feeling lightheaded**

If you have any of these symptoms, **seek medical help immediately**, especially if you experience difficulty breathing, chest pain, or severe allergic reactions.

If your symptoms are mild but persistent, contact your doctor for further advice.

# 61. I did heart MRI. What symptoms after the test should prompt me to call my doctor?

After a **heart MRI**, most patients experience no side effects, as the test is generally safe and non-invasive. However, you should **contact your doctor** if you experience any of the following symptoms:

**1. Symptoms Related to Contrast Agent (Gadolinium)**

- **Allergic reactions** (itching, rash, hives, or swelling of the face, lips, or throat)
- **Shortness of breath or wheezing**
- **Dizziness or fainting**
- **Nausea, vomiting, or severe headache**
- **Flushing or warmth that persists beyond a few hours**
- **Swelling or pain at the injection site**

*(Note: If you have kidney disease, be alert for worsening kidney function, as gadolinium can sometimes cause complications.)*

**2. Symptoms Related to Implantable Devices**

- If you have a **pacemaker, defibrillator, or metal implant**, and you experience:
  - **Chest pain or palpitations**
  - **Dizziness or lightheadedness**
  - **Unexpected shocks or abnormal heart rhythms**

**3. Unusual Fatigue or Weakness**

- Persistent or **worsening fatigue** beyond normal
- Muscle pain or weakness, which could indicate a rare reaction to gadolinium

**4. Neurological Symptoms**

- Blurred vision
- Difficulty speaking
- Confusion or trouble concentrating

**5. Severe Pain or Swelling**

- Pain at the IV site that worsens
- Swelling in the arms or legs

If you experience any of the above symptoms, especially difficulty breathing, chest pain, or an allergic reaction, seek **immediate medical attention**. If your symptoms are mild but persistent, contact your doctor for further evaluation.

# 62. Can I travel (or fly) shortly after having a cardiac CT?

Yes, in most cases, you can travel or fly shortly after having a **cardiac CT scan**, as it is a non-invasive imaging test. However, there are a few considerations to keep in mind:

**1. Type of Cardiac CT and Travel Considerations**

- **Standard Cardiac CT (without contrast):** No restrictions; you can travel immediately.
- **CT Angiography (CTA) with Contrast:** If iodine-based contrast dye is used, you should stay hydrated to help flush it from your system.
- **Stress CT with Medications:** If your scan involved beta-blockers or vasodilators (e.g., adenosine, regadenoson), you might feel lightheaded or tired for a short period.

**2. Radiation Exposure & Security Scanners**

- The radiation from a CT scan does not make you radioactive, and it will not affect airport security scanners.
- If you had contrast dye, it does not set off security alarms.

**3. Symptoms to Watch For Before Traveling**

- **Allergic Reaction to Contrast:** Rare, but if you develop rash, itching, or breathing difficulty, seek medical help.
- **Kidney Issues:** If you have kidney disease or are at risk, ensure your kidney function is monitored.
- **Side Effects from Medications:** If you were given beta-blockers, check if you feel dizzy before flying.

**4. Long Flights & Blood Clots**

- If you have other **cardiac risk factors** or are prone to **deep vein thrombosis (DVT)**, consider moving your legs during a long flight to reduce clot risks.

**Conclusion**

For most patients, **there is no restriction on travel or flying after a cardiac CT**. If you have specific medical conditions (such as severe kidney disease or contrast allergy), check with your doctor before traveling.

Would you like specific guidance based on your health condition?

# 63. Can I travel (or fly) shortly after having a cardiac MRI?

Yes, you can generally travel or fly shortly after having a **cardiac MRI**, as the procedure itself does not have significant aftereffects that would prevent you from traveling. However, there are a few considerations to keep in mind:

**1. Use of Contrast (Gadolinium)**

- If your cardiac MRI included a contrast agent (gadolinium-based), you might be advised to stay hydrated to help your kidneys eliminate the contrast more effectively.
- Some people experience mild side effects like nausea or dizziness, which could make traveling slightly uncomfortable.

**2. Sedation or Medication**

- If you were given **sedation** (which is rare for most cardiac MRIs), you may need someone to accompany you, and you might be advised to wait a few hours before traveling.
- If you were prescribed **beta-blockers** (to slow your heart rate for better imaging), they could cause lightheadedness or fatigue.

**3. Claustrophobia or Anxiety**

- If you took a mild anxiolytic (like lorazepam) due to claustrophobia, it may impair your alertness temporarily, making immediate travel inadvisable.

**4. Personal Health Condition**

- If your MRI was ordered due to recent **cardiac symptoms (chest pain, shortness of breath, arrhythmia, etc.)**, your doctor may want to review the results before you travel.
- If your condition is stable and the MRI was routine, travel is generally safe.

**5. Flight Considerations**

- Air travel itself poses **no direct risks** after an MRI.
- If you have **severe heart disease**, long flights could increase the risk of deep vein thrombosis (DVT), so wearing compression stockings and staying hydrated is recommended.

**Bottom Line**

If your MRI was done as a routine test and you feel well, there are no absolute restrictions on traveling or flying. However, if you have any concerns, especially regarding your underlying heart condition, contrast reaction, or sedation effects, it's best to consult your doctor before flying.

# 64. Is there a radiation risk with cardiac CT, and how significant is it?

Yes, there is a **radiation risk** associated with **cardiac CT (computed tomography)**, but its significance depends on various factors, including the type of CT scan, patient characteristics, and the scanning protocol used.

**1. Radiation Dose in Cardiac CT**

- **Typical Effective Dose**:
  - **Coronary CT Angiography (CCTA)**: **1 - 12 mSv** (varies based on protocol)
  - **Calcium Scoring CT**: **0.5 - 3 mSv**
  - **Stress Perfusion Cardiac CT**: **Higher doses (~10-15 mSv)**
- **Comparison to Background Radiation**:
  - The **average annual natural background radiation** exposure is about **3 mSv**.
  - A **single cardiac CT scan** can expose a patient to radiation equivalent to a **few months to several years** of natural background radiation.

**2. Risk of Radiation-Induced Cancer**

- The risk is **low but not negligible**, particularly for:
  - **Younger patients** (higher lifetime cancer risk per unit dose)
  - **Females** (higher radiation sensitivity, especially to breast tissue)
  - **Patients requiring repeated scans** over time
- Studies estimate that a **dose of 10 mSv** might be associated with a **1 in 2,000 to 1 in 3,000** risk of developing cancer. However, this risk is small compared to the diagnostic and prognostic benefits in appropriately selected patients.

**3. Dose Reduction Strategies**

To minimize radiation exposure, several techniques are used:

- **Prospective ECG Gating**: Reduces dose compared to retrospective gating by scanning only during specific heartbeats.
- **Lower Tube Voltage (kVp)**: Especially for younger and smaller patients.
- **Iterative Reconstruction Algorithms**: Improve image quality at lower doses.
- **Use of Dual-Energy or Photon-Counting CT**: Advanced techniques with lower radiation dose potential.
- **Limiting Scan Length**: Reducing unnecessary scanned regions.
- **Alternative Imaging Modalities**:
  - **MRI or Echocardiography** if feasible, as they do not involve ionizing radiation.

**4. Clinical Perspective: Balancing Risk vs. Benefit**

- The benefit of a **well-indicated cardiac CT scan** (e.g., ruling out significant coronary artery disease in low-intermediate risk patients) generally **outweighs the small radiation risk**.
- Unnecessary scans should be avoided, and alternative imaging methods should be considered when appropriate.

**Bottom Line**

While there is a **small radiation risk**, modern **dose-reduction techniques** significantly minimize exposure, and the **clinical benefit of cardiac CT**—especially in evaluating coronary artery disease—often outweighs the potential risks when used appropriately.

# 65. Is there a radiation risk with cardiac MRI, and how significant is it?

No, **cardiac MRI (CMR) does not involve ionizing radiation**, making it a **radiation-free imaging modality**. Unlike **CT scans** and **nuclear imaging**, which expose patients to ionizing radiation, MRI relies on **strong magnetic fields and radiofrequency waves** to create detailed images of the heart.

**Why is there no radiation risk?**

- MRI uses **radiofrequency (RF) pulses**, which are **non-ionizing**, meaning they do not have enough energy to remove tightly bound electrons or cause cellular damage.
- The **magnetic field (measured in Tesla, T)** aligns protons in the body, and radio waves cause them to produce signals that are converted into images.

**How does this compare to other cardiac imaging modalities?**

| **Imaging Modality** | **Radiation Exposure (mSv)** | **Risk Level** |
| --- | --- | --- |
| **Cardiac MRI** | **0** | **No radiation** |
| **Echocardiography** | **0** | **No radiation** |
| **Coronary CT Angiography (CTA)** | **1-12 mSv** (varies) | **Low to moderate** |
| **Nuclear Stress Test (MPI)** | **5-25 mSv** (depends on protocol) | **Moderate to high** |
| **Cardiac Catheterization (Angiography)** | **5-15 mSv** | **Moderate** |

**Clinical Implications**

- **Safe for all patients**, including children and pregnant women (except in the first trimester due to theoretical concerns about heating effects).
- **Preferred for repeated imaging** in younger patients to avoid cumulative radiation exposure.
- **Particularly useful in structural heart disease, myocarditis, cardiomyopathies, and viability assessment** without radiation concerns.

**Are there any risks with MRI?**

While there is no radiation risk, **MRI has other safety considerations**, such as:

- **Metallic implants (e.g., pacemakers, defibrillators)** – some are MRI-compatible, but others may pose risks.
- **Claustrophobia** – some patients may need sedation.
- **Gadolinium-based contrast risks** – not radiation-related, but can cause nephrogenic systemic fibrosis (NSF) in patients with severe kidney disease.

**Bottom Line**

**Cardiac MRI is a radiation-free imaging modality**, making it an excellent choice for assessing cardiovascular disease, particularly in younger patients or those requiring serial imaging.

# 66. I have nails in my arm, can I do heart MRI?

It depends on the type of nails in your arm. Here’s what you need to consider before undergoing a **cardiac MRI**:

**1. Material of the Nails**

- **If the nails are stainless steel or any ferromagnetic material** → **MRI is unsafe** as the strong magnetic field could move the nails or cause heating.
- **If the nails are titanium or non-magnetic stainless steel** → **MRI is generally safe**, as these materials are non-ferromagnetic.

**2. MRI Compatibility Check**

- If you had surgery and the nails were implanted, check your **surgical report** or ask your doctor about the implant material.
- You can also ask the radiology department to check MRI safety databases to see if your hardware is **MRI-safe** or **MRI-conditional**.

**3. Risk of Heating & Artifacts**

- Even if the nails are **MRI-safe**, they can still **cause artifacts**, especially in cardiac MRI, which relies on clear imaging.
- If the nails are near the **chest or shoulder**, they might distort the images, making the scan less useful.

**4. Alternatives if MRI is Unsafe**

- **CT scan**: A good alternative if MRI is not possible.
- **Echocardiography**: May be helpful depending on what your doctor needs to assess.

**What You Should Do Now**

- Contact your **radiologist** or **surgeon** and ask about the type of metal.
- If you don’t know the material, an **X-ray** can sometimes help determine if the nails are ferromagnetic.

Would you like help with checking MRI safety for a specific implant brand or type ?

# 67. I have an inserted heart device. Can I do heart MRI?

**It depends on the type of heart device you have. Generally, there are three categories of heart devices concerning MRI safety:**

**1. MRI-Conditional Devices (Safe Under Certain Conditions)**

- **Many modern pacemakers, implantable cardioverter defibrillators (ICDs), and cardiac resynchronization therapy (CRT) devices are MRI-conditional.**
- **This means they can be safely scanned under specific conditions (e.g., certain magnetic field strengths, device programming adjustments, and monitoring during the scan).**
- **Your cardiologist or electrophysiologist should check if your device is MRI-compatible and ensure that safety protocols are followed.**

**2. Non-MRI-Conditional Devices (Potentially Unsafe)**

- **Older pacemakers, ICDs, and other implanted cardiac devices may not be MRI-compatible.**
- **MRI can potentially interfere with their function, causing heating, movement, or unintended stimulation.**
- **In these cases, MRI is usually not recommended, but alternatives such as CT or echocardiography may be considered.**

**3. Other Implanted Cardiac Devices**

- **Loop recorders: Some are MRI-compatible, but conditions vary.**
- **Mechanical/prosthetic valves and stents: Generally safe, but confirmation is necessary.**
- **LVAD (left ventricular assist devices): Typically not MRI-compatible.**

**Next Steps**

1. **Check Your Device Model: Find out if your device is MRI-conditional by looking at your medical records, implant card, or asking your cardiologist.**
2. **Consult Your Cardiologist: They can determine if an MRI is safe for you and whether special protocols need to be followed.**
3. **Inform the Radiologist: If an MRI is needed, provide details about your heart device so they can plan accordingly.**

**Would you like help checking the MRI safety of your specific device?**

# 68. I am pregnant, can I undergo heart CT?

In general, a **heart CT (coronary CT angiography or cardiac CT) is not recommended during pregnancy** unless it is absolutely necessary. This is because CT scans use **ionizing radiation**, which can pose a risk to the developing fetus, especially in the first trimester.

**Considerations for Heart CT in Pregnancy:**

1. **Radiation Exposure:** A heart CT involves a small amount of radiation, but it is typically directed at the chest, not the abdomen. However, scatter radiation can still reach the fetus.
2. **Use of Contrast (Iodinated Contrast):** If a contrast agent is needed, there is limited evidence regarding its safety in pregnancy. Some concerns exist about its effects on fetal thyroid function.
3. **Alternative Imaging:** If possible, doctors usually prefer **echocardiography (ultrasound)** or **MRI** to assess heart conditions during pregnancy, as these do not use radiation.
4. **Urgency of the Condition:** If the CT is **medically necessary** (e.g., life-threatening heart disease), precautions can be taken to minimize fetal exposure.

**What Should You Do?**

- **Consult with your doctor** and radiologist about the necessity of the scan.
- **Consider alternative imaging** like echocardiography or MRI if suitable.
- **If CT is unavoidable**, lead shielding may be used to protect the abdomen.

Would you like help finding alternative imaging options for your specific condition?

# 69. I am pregnant, can I undergo heart MRI?

Yes, you can undergo a **cardiac MRI** while pregnant, but there are some considerations:

**Safety Considerations**

1. **Magnetic Field Exposure**
   - MRI does not use ionizing radiation, so it is generally considered safer than CT scans during pregnancy.
   - Studies suggest that MRI, particularly at **1.5T**, does not harm the developing fetus, especially after the first trimester.
2. **Trimester-Specific Considerations**
   - **First Trimester:** MRI is generally avoided unless absolutely necessary, as organ development occurs during this period.
   - **Second & Third Trimester:** Considered safer, and MRI is more commonly performed if clinically indicated.
3. **Use of Gadolinium Contrast**
   - Gadolinium-based contrast agents **should be avoided** in pregnancy unless absolutely necessary because they can cross the placenta and have been associated with possible risks to the fetus.
   - If contrast is needed, the risks and benefits should be carefully assessed.

**Indications for Cardiac MRI in Pregnancy**

Your doctor may recommend a **heart MRI** if:

- You have congenital or structural heart disease.
- You need evaluation for **cardiomyopathies** (e.g., peripartum cardiomyopathy).
- There is suspicion of cardiac masses or **aortic pathology**.
- Echocardiography or other imaging modalities are insufficient.

**Alternative Imaging Options**

Depending on your condition, your doctor may consider:

- **Echocardiography (TTE or TEE)** – Preferred as the first-line imaging modality since it is safe and does not involve radiation or strong magnetic fields.
- **CT Angiography (CTA)** – Can be considered if absolutely necessary, but it involves radiation, which is generally avoided in pregnancy.

**Conclusion**

A cardiac MRI **without contrast** is generally safe during pregnancy, particularly in the **second and third trimesters**. However, it should only be done when medically necessary. Always discuss with your doctor to weigh the risks and benefits in your specific case.

Would you like help with any other pregnancy-related concerns?

# 70. I have kidney problems, can I do heart CT?

If you have kidney problems, undergoing a **heart CT (cardiac CT)** requires careful consideration, especially regarding the use of **contrast dye (iodinated contrast)**, which can affect kidney function.

**Key Considerations:**

1. **Assessing Kidney Function (eGFR)**
   - Before the CT scan, your doctor will check your **kidney function** using a blood test to measure your **estimated glomerular filtration rate (eGFR)**.
   - If **eGFR is above 60**, contrast is generally safe.
   - If **eGFR is between 30-60**, extra precautions may be taken.
   - If **eGFR is below 30**, contrast is usually avoided unless absolutely necessary.
2. **CT Without Contrast**
   - If your kidney function is poor, a **non-contrast CT** (such as calcium scoring) may still be an option.
3. **Hydration**
   - If contrast is needed, **adequate hydration** before and after the scan helps reduce kidney stress.
   - In some cases, **IV fluids** or medications (like sodium bicarbonate or N-acetylcysteine) might be used to protect the kidneys.
4. **Alternatives**
   - Depending on your condition, your doctor may consider alternatives such as **cardiac MRI**, **stress echocardiography**, or **nuclear stress testing**, which may be safer for your kidneys.

Would you like help interpreting your kidney function test results to determine the safest option?

# 71. I have kidney problems, can I do heart MRI?

Yes, you can undergo a **cardiac MRI**, but with some precautions depending on your **kidney function**.

**Key Considerations:**

1. **Without Contrast (Safe)**
   - If a **contrast agent is not required**, cardiac MRI is generally **safe** for patients with kidney problems.
2. **With Contrast (Gadolinium-Based)**
   - If your doctor recommends **contrast-enhanced MRI** (to evaluate heart structures, perfusion, or scar tissue), they will assess your **kidney function (eGFR - estimated glomerular filtration rate).**
   - **If eGFR > 30 mL/min/1.73m²** → Contrast is **usually safe**.
   - **If eGFR < 30 mL/min/1.73m² or on dialysis** → Gadolinium contrast **may increase the risk of nephrogenic systemic fibrosis (NSF)**, a rare but serious condition. In such cases:
     - Your doctor might choose **a non-contrast protocol** or an **alternative imaging test** like echocardiography or cardiac CT.
     - Some centers use **low-dose or macrocyclic gadolinium agents**, which are safer.

**What Should You Do?**

- **Inform your doctor and radiologist** about your kidney issues.
- **Check your latest kidney function (eGFR)** before scheduling the MRI.
- **Discuss alternative imaging options** if needed.

Would you like help understanding alternative tests if contrast MRI isn't safe for you?

# 72. If I’m claustrophobic, can I do heart MRI?

Yes, you can still undergo a **heart MRI** if you're claustrophobic, but there are several strategies to make the experience more comfortable:

**Options to Manage Claustrophobia During a Heart MRI**

1. **Open or Wide-Bore MRI Machines**
   - Some facilities have **wide-bore MRI scanners** (70 cm diameter) or **open MRI machines** that reduce the feeling of confinement.
2. **Sedation or Anxiolytic Medications**
   - A mild sedative (like **lorazepam or diazepam**) can help relax you if prescribed by your doctor.
   - If severe, some centers may offer **IV sedation** to keep you calm.
3. **Shorter Scans and Fast Sequences**
   - Some centers use **accelerated imaging protocols** to reduce scan time while maintaining quality.
4. **Music, Headphones, or Eye Masks**
   - Many MRI machines allow you to listen to **music or guided relaxation** to distract you.
   - **Eye masks** can prevent you from seeing the enclosed space.
5. **Mirror or Prism Glasses**
   - Some MRI machines have **mirrors or prism glasses** that make it feel like you're looking outside rather than into a tunnel.
6. **Breathing and Relaxation Techniques**
   - Practicing **deep breathing** or **mindfulness** before and during the scan can help manage anxiety.
7. **Having a Companion Nearby**
   - Some centers allow a **friend or family member** to be in the room with you for reassurance.
8. **Talking to the Technician**
   - MRI technicians can **communicate with you** throughout the scan via an intercom, which can be comforting.
9. **Alternative Imaging Modalities** (if MRI is not possible)
   - In some cases, a **cardiac CT scan** may be an alternative for certain heart conditions.

**What You Should Do**

- Inform your doctor and the MRI team about your **claustrophobia** ahead of time.
- Ask if the facility has **wide-bore or open MRI scanners**.
- Discuss the possibility of **sedation** if your anxiety is severe.

Would you like help finding an MRI facility that offers these options?

# 73. My doctor is suggesting heart CT, could a cardiac ultrasound (echo) or stress test replace the CT?

Whether a **cardiac CT** (such as a **coronary CT angiography (CCTA)** or **calcium scoring CT**) can be replaced by an **echocardiogram (echo)** or a **stress test** depends on the reason your doctor is recommending the CT. Each test provides different types of information about your heart:

**When is a Cardiac CT necessary?**

A **cardiac CT** is typically used for:

1. **Coronary Artery Disease (CAD) evaluation**:
   - **Coronary CT Angiography (CCTA):** Detects blockages or narrowing in coronary arteries.
   - **Calcium Score CT:** Measures calcium deposits to assess the risk of heart disease.
2. **Congenital heart disease** or **anomalies** of the coronary arteries.
3. **Aortic disease** (e.g., aortic aneurysm, dissection).
4. **Pre-procedural planning**, such as for transcatheter aortic valve replacement (TAVR) or bypass surgery.

**Can an Echocardiogram Replace a CT?**

A **transthoracic echocardiogram (TTE) or transesophageal echocardiogram (TEE)** is useful for:

- **Assessing heart function** (ejection fraction, heart muscle movement).
- **Valve disease evaluation** (stenosis, regurgitation).
- **Identifying structural abnormalities** (septal defects, pericardial effusion).
- **Detecting large thrombi or tumors** inside the heart.

🔹 **Limitations:** Echo **does not visualize the coronary arteries** well, so it **cannot replace CT** if the goal is to check for **coronary blockages**.

**Can a Stress Test Replace a CT?**

A **stress test (exercise ECG, stress echocardiogram, or nuclear stress test)** helps assess:

- **Blood flow to the heart during exercise** (ischemia).
- **Heart function under stress** (how well the heart pumps when working harder).

🔹 **Limitations:** Stress tests **do not directly visualize coronary arteries**. If your doctor is looking for **plaque buildup, anatomical abnormalities, or non-obstructive CAD**, a **CT would still be needed**.

**When Can You Avoid a CT?**

- If you **only need to assess heart function**, an **echo** may be sufficient.
- If your doctor **only wants to evaluate for significant coronary blockages**, a **stress test** could be an alternative.
- If you have **low risk for CAD** and are asymptomatic, your doctor may suggest **lifestyle changes instead of imaging**.

**Conclusion**

A **cardiac CT is irreplaceable if the goal is to visualize coronary arteries or detect calcium deposits**. If your doctor is primarily evaluating heart function, an **echocardiogram** may be enough. If they are assessing for significant coronary blockages, a **stress test** could be an alternative.

Would you like help understanding your doctor's specific concern and which test would be best for you?

# 74. What is I can't undergo heart MRI, are there any alternatives?

If you cannot undergo a **cardiac MRI**, there are several alternative imaging modalities depending on the clinical question. Here are some alternatives:

**1. Echocardiography (Echo)**

- **Transthoracic Echocardiography (TTE)**: First-line imaging for cardiac structure and function assessment.
- **Transesophageal Echocardiography (TEE)**: Provides better visualization of the left atrium, aorta, and valves.
- **Strain Echocardiography**: Assesses myocardial deformation and fibrosis indirectly.
- **Contrast Echocardiography**: Can enhance visualization in cases with poor acoustic windows.

**2. Cardiac CT (CCT)**

- **Coronary CT Angiography (CCTA)**: A great alternative if MRI is not feasible, especially for coronary artery disease (CAD).
- **CT for Myocardial Characterization**: Dual-energy or delayed iodine enhancement CT can provide information on fibrosis (although not as detailed as MRI).
- **Calcium Scoring**: Helps assess atherosclerosis burden.
- **CT-derived ECV**: Emerging as an alternative to MRI for extracellular volume fraction (ECV) assessment.

**3. Nuclear Imaging**

- **PET Scan (Positron Emission Tomography)**: Useful for viability assessment and inflammatory conditions (e.g., sarcoidosis, myocarditis).
- **SPECT (Single Photon Emission Computed Tomography)**: Used for ischemia evaluation and myocardial perfusion imaging.
- **MUGA Scan (Multi-Gated Acquisition Scan)**: Can assess left ventricular function with high precision.

**4. Cardiac Catheterization**

- **Invasive Coronary Angiography**: Gold standard for assessing coronary artery disease.
- **Endomyocardial Biopsy**: Used for definitive diagnosis of myocarditis, amyloidosis, or rejection after transplantation.

**Choosing the Right Alternative**

The best alternative depends on why the MRI was needed:

- **Structural and functional assessment** → **Echocardiography or CT**
- **Myocardial tissue characterization (fibrosis, edema, inflammation)** → **Nuclear imaging (PET) or contrast-enhanced CT**
- **Coronary artery disease** → **CT angiography or invasive angiography**
- **Myocardial viability** → **PET, nuclear imaging, or contrast-enhanced echocardiography**

Would you like guidance on the best option for a specific condition?

# 75. I am supposed to do Heart CT. However, I have allergy to contrast dye—are there alternative options?

Yes! If you have an allergy to contrast dye (iodinated contrast), there are alternative approaches depending on the purpose of your **Heart CT**:

**1. Pre-Medication for Contrast Allergy**

- If contrast is essential (e.g., for a **Coronary CT Angiography**), many patients with contrast allergies can **safely receive contrast** after taking pre-medication (usually corticosteroids and antihistamines) before the scan.
- Discuss this option with your doctor or radiologist.

**2. Non-Contrast CT Alternatives**

- **Calcium Score CT (Coronary Artery Calcium Scan)**: This is a non-contrast CT that assesses coronary artery calcium buildup. It is useful for risk assessment of coronary artery disease but **cannot evaluate soft plaques or stenosis**.
- **Non-Contrast Cardiac MRI**: If functional or anatomical assessment is needed, a **Cardiac MRI** may be an alternative. Some MRI sequences don’t require contrast and can still provide valuable information.

**3. Nuclear Cardiac Imaging**

- If the CT was meant to evaluate **myocardial perfusion or ischemia**, a **nuclear stress test (SPECT or PET)** may be an alternative, using a radioisotope instead of contrast dye.

**Next Steps:**

- **Confirm the necessity of contrast** with your cardiologist/radiologist.
- **Discuss pre-medication protocols** if contrast is unavoidable.
- **Explore non-contrast imaging alternatives** based on your clinical need.

Would you like help finding specific pre-medication protocols or alternative tests suited to your condition?

# 76. Since there's no radiation risk in heart MRI, why is my doctor recommending heart CT?

Your doctor may be recommending a **heart CT (computed tomography)** instead of a **heart MRI (magnetic resonance imaging)** for several possible reasons, despite the absence of radiation in MRI. Here are some potential explanations:

**1. Better for Coronary Arteries**

- **Coronary CT Angiography (CCTA)** is superior to MRI for **visualizing coronary arteries** and detecting **coronary artery disease (CAD)**, including plaque buildup and blockages.
- MRI is generally **not as effective** for detailed imaging of the coronary arteries due to motion and spatial resolution limitations.

**2. Faster and More Available**

- A **heart CT scan** is much **faster** (usually a few seconds) compared to an MRI, which can take **30-60 minutes**.
- In emergency or urgent cases (e.g., suspected heart attack or aortic dissection), a CT is often the first choice.

**3. Calcification Detection**

- **CT is the gold standard** for detecting **coronary artery calcifications** (Calcium Score Test), which MRI **cannot accurately assess**.

**4. Specific Indications**

- Some heart conditions are better evaluated with CT, such as:
  - **Aortic dissection or aneurysm** (CT is more detailed and faster)
  - **Congenital heart defects**
  - **Pericardial disease** (such as constrictive pericarditis)
  - **Post-surgical anatomy** (e.g., coronary bypass grafts)

**5. Implants or Claustrophobia**

- If you have certain **implants** (e.g., pacemaker, defibrillator) that are **not MRI-compatible**, a CT scan may be safer.
- **Claustrophobia** can also make an MRI challenging.

**6. Functional vs. Structural Imaging**

- **MRI** is excellent for evaluating **heart function, fibrosis, and myocarditis**, while **CT** excels at **structural details**, especially of the **vessels**.

**Should You Ask for an MRI Instead?**

If you are concerned about radiation, you can ask your doctor **if an MRI would be a suitable alternative**. However, if your doctor is specifically looking for **coronary artery disease, calcifications, or urgent findings**, a CT may be the better option.

Would you like me to help interpret your doctor’s reasoning based on your specific case?

# 77. I did heart CT, When and how will I receive my results?

The timeline and method for receiving your **heart CT (coronary CT angiography or calcium scoring CT) results** depend on the facility where you had the scan and the urgency of your case. Here’s what to expect:

**1. When Will You Receive Your Results?**

- **Routine cases**: Usually within **1–3 days**, but it may take up to **a week** if it’s a non-urgent scan.
- **Urgent cases**: If the scan was ordered for an emergency (e.g., suspected acute coronary syndrome), the results may be available **within hours**.
- **If additional review is needed**: Sometimes, radiologists need more time to consult with cardiologists before finalizing the report.

**2. How Will You Receive Your Results?**

- **Online Portal**: Many hospitals or imaging centers upload results to a patient portal where you can access your radiology report.
- **Phone Call from Your Doctor**: If your doctor ordered the test, they may call you to discuss the findings.
- **In-Person Follow-up Appointment**: Some doctors prefer to go over imaging findings in detail during a visit.
- **Direct from the Radiology Center**: Some facilities allow you to pick up a copy of your report and images on a CD or USB drive.

**3. What Should You Do Next?**

- If you **haven’t received your results within a week**, you can call your doctor’s office or the radiology center to check the status.
- If you receive the report **directly from an online portal** but don’t understand the findings, schedule an appointment with your doctor to discuss them.

Would you like help interpreting your report once you receive it?

# 78. I did heart MRI, when and how will I receive my results?

The timing and method of receiving your **heart MRI results** depend on the facility where you had the scan. Generally:

1. **Processing Time:**
   - **Preliminary Review:** The MRI images are first reviewed by a radiologic technologist or imaging specialist, but they do not provide a report.
   - **Radiologist Interpretation:** A specialized cardiac radiologist will analyze your MRI, compare it with clinical history, and generate a formal report. This typically takes **1–3 days** in most hospitals but can take up to **1–2 weeks** in some settings.
2. **Receiving the Results:**
   - **Through Your Doctor:** Your ordering physician (cardiologist or primary care doctor) will receive the report and discuss the findings with you. They may schedule a follow-up appointment or call you.
   - **Patient Portal (If Available):** Some hospitals upload MRI reports to an online patient portal, allowing you to view them directly.
   - **Direct Request:** If you need the results urgently, you can contact the radiology department or your doctor’s office.
3. **Next Steps:**
   - If the MRI shows normal findings, no further action may be needed.
   - If abnormalities are detected, your doctor may suggest further testing or treatment.

Since you have a **medical background**, do you want guidance on interpreting the MRI report once you receive it?

# 79. Will I need additional testing after heart CT?

Whether you need additional testing after a **heart CT (coronary CT angiography or calcium scoring CT)** depends on the findings of the scan and your clinical situation. Here’s a breakdown:

**1. If the Heart CT is Normal**

- If **no significant plaque or narrowing** is found, further testing is usually **not necessary**.
- Your doctor may still focus on **preventive measures**, such as managing risk factors (cholesterol, blood pressure, diabetes, lifestyle changes).

**2. If Mild to Moderate Coronary Artery Disease (CAD) is Found**

- If your scan shows **mild or moderate plaque buildup**, additional testing may be needed, such as:
  - **Stress testing (exercise or pharmacologic stress test)** – To assess if the plaques are restricting blood flow.
  - **Blood tests (cholesterol, inflammatory markers like hs-CRP, or cardiac enzymes if symptoms suggest ischemia)**.
  - **Advanced lipid panel** (if high-risk plaques are found).

**3. If Significant Blockages are Found**

- If your heart CT shows **severe narrowing (>70% stenosis) or signs of high-risk plaque**, your doctor may recommend:
  - **Invasive coronary angiography (cardiac catheterization)** – To directly assess blockages and possibly place a stent.
  - **Nuclear stress test or cardiac MRI** – To check if the heart muscle is getting enough blood supply.

**4. If Incidental Findings Are Present**

- **Lung nodules or aortic disease** seen on the CT may require additional imaging (chest CT, echocardiography).
- If calcifications are seen in the **aorta or valves**, further echocardiography or follow-up imaging might be needed.

**5. If You Have Symptoms Despite a Normal or Mildly Abnormal CT**

- If you continue to have **chest pain, shortness of breath, or other cardiac symptoms**, your doctor may still recommend additional functional testing (like a stress test or Holter monitor) to rule out microvascular disease or arrhythmias.

Would you like me to help interpret a specific result or explain any findings further?

# 80. I am undergoing heart MRI. Can I obtain copies of the images or medical reports for my personal records?

Yes, you can obtain copies of your **cardiac MRI images and reports** for your personal records. Here’s how:

**1. Ask the Imaging Center or Hospital**

- Most radiology departments allow patients to request copies of their images and reports.
- You may need to contact the **Medical Records Department** or the **Radiology Department** directly.

**2. Request Format Options**

- **DICOM files**: These are standard medical imaging files that can be viewed using specialized software.
- **CD/DVD or USB**: Many facilities provide images on a disc or USB with a built-in viewer.
- **Printed or PDF Reports**: Your radiology report can be provided in print or electronically.

**3. Authorization & ID**

- You may need to fill out a **medical release form** and present a valid **ID**.
- Some hospitals allow online requests via patient portals.

**4. Check for Any Fees**

- Some institutions provide copies for free, while others may charge a fee for CDs, USBs, or printed reports.

**5. Use for Second Opinions or Personal Records**

- If you are applying for a **research position** or keeping records for future medical visits, having these images is beneficial.

Would you like help understanding the MRI report when you receive it?

# 81. If my heart CT turns to be normal, does that guarantee I’m free from heart disease?

A normal **coronary CT angiography (CCTA)** or **calcium score** significantly lowers the likelihood of significant coronary artery disease (CAD), but it does **not** completely guarantee that you are free from heart disease. Here’s why:

**What a Normal Heart CT Means:**

1. **Coronary Calcium Score = 0**: This means no detectable calcified plaque, making significant obstructive coronary artery disease (CAD) **very unlikely**.
2. **Normal Coronary CT Angiography (CCTA)**: If your CCTA is normal, it suggests **no significant blockages or narrowing in the coronary arteries**, which makes a heart attack from plaque rupture very unlikely in the near future.

**Limitations of a Normal Heart CT:**

1. **Non-calcified Plaques May Be Missed**
   - A calcium score of 0 does **not** rule out **soft (non-calcified) plaques**, which may still be present and potentially rupture.
   - A CCTA can detect soft plaques, but very early-stage plaques may still be present.
2. **Microvascular Disease or Spasm**
   - **Coronary microvascular disease** affects the small arteries of the heart and does **not** show up on standard CT scans.
   - **Coronary artery spasm (Prinzmetal's angina)** can cause chest pain even if large arteries are clear.
3. **Other Types of Heart Disease**
   - **Valvular heart disease**, **arrhythmias**, or **myocardial diseases (e.g., cardiomyopathy, myocarditis, amyloidosis)** are **not** ruled out by a coronary CT.
4. **Acute vs. Long-Term Risk**
   - A normal heart CT gives **low short-term risk** (usually 5–10 years), but it does not prevent future plaque buildup due to lifestyle, genetics, or other factors.

**What Should You Do If Your Heart CT Is Normal?**

- If you have **no symptoms** and no major risk factors, your risk of a heart event is likely **very low**.
- If you **still have symptoms (e.g., chest pain, shortness of breath, palpitations)**, further testing (e.g., stress test, echocardiogram, MRI) may be needed.
- Maintain **heart-healthy habits** (exercise, diet, blood pressure, cholesterol control).

**Bottom Line**

A normal heart CT is **a very reassuring result**, especially for ruling out obstructive CAD, but it is **not an absolute guarantee** that you are completely free from heart disease. Always consider symptoms, risk factors, and clinical history in decision-making.

# 82. If my heart MRI turns to be normal, does that guarantee I’m free from heart disease?

A normal heart MRI is a reassuring result, but it **does not completely rule out all forms of heart disease**. While MRI is one of the most advanced imaging techniques for assessing cardiac structure and function, it has limitations. Here’s what a normal heart MRI does and does not mean:

**What a Normal Heart MRI Means:**

✅ **No Structural Abnormalities** – Your heart chambers, valves, and walls appear normal.
✅ **Normal Heart Function** – Your heart’s pumping ability (ejection fraction) is within a healthy range.
✅ **No Obvious Fibrosis or Scarring** – Late gadolinium enhancement (LGE) can detect scar tissue from past heart attacks or myocarditis, and a normal MRI suggests no significant fibrosis.
✅ **No Myocardial Edema or Inflammation** – If done with T2-weighted imaging, it can rule out conditions like myocarditis.
✅ **No Large Vessel Disease** – It can assess for congenital abnormalities and some large vessel diseases.

**What a Normal MRI Does *Not* Rule Out:**

🚫 **Coronary Artery Disease (CAD) with Mild or Non-obstructive Plaque** – MRI does not assess coronary arteries directly. A normal MRI does not exclude early atherosclerosis or microvascular dysfunction.
🚫 **Ischemia (Blood Flow Issues)** – If your MRI did not include stress perfusion imaging, it cannot evaluate how well your heart responds to stress or detect ischemia.
🚫 **Early or Subclinical Heart Disease** – Some conditions, like early-stage cardiomyopathy or metabolic/inflammatory diseases, may not yet show clear MRI findings.
🚫 **Arrhythmias and Electrical Heart Disease** – Conditions like long QT syndrome, Brugada syndrome, or conduction disorders do not show up on MRI.
🚫 **Microvascular Dysfunction** – Small vessel disease (common in diabetes or hypertension) may not be visible on MRI.
🚫 **Valve or Functional Abnormalities Under Certain Conditions** – Some valve diseases only manifest with stress or exercise and might not be detected at rest.

**Next Steps if You’re Concerned About Heart Disease:**

- If you have **risk factors** (family history, high cholesterol, smoking, diabetes, hypertension), additional tests may be needed (e.g., coronary CT angiography, stress test, lipid panel).
- If you have **persistent symptoms** (chest pain, shortness of breath, palpitations), further workup may be necessary despite a normal MRI.
- If needed, a **coronary calcium score** or **stress imaging** (stress MRI, stress echo, or nuclear perfusion scan) can assess ischemia risk.

**Bottom Line:**

A normal heart MRI is **a very good sign** that your heart is structurally healthy, but it does not guarantee you are free from all heart diseases, particularly coronary artery disease, electrical abnormalities, or microvascular dysfunction. If you have ongoing symptoms or risk factors, further evaluation may be necessary.

# 83. My doctor says that my heart CT is positive, how certain is that?

A "positive" heart CT result typically means that some abnormality was detected, such as coronary artery calcifications (in a calcium score test) or narrowing of the coronary arteries (in a coronary CT angiography). The certainty of this result depends on several factors:

**1. Type of Heart CT You Had**

- **Coronary Artery Calcium (CAC) Score**: Measures calcium buildup in your coronary arteries. A positive result means some level of calcium is present, which indicates a higher risk of coronary artery disease (CAD).
- **Coronary CT Angiography (CCTA)**: Uses contrast dye to assess the degree of narrowing in your coronary arteries. A positive result suggests blockages, but false positives can occur due to artifacts or overestimation of stenosis.
- **CT for Structural Heart Disease**: Used for conditions like aortic stenosis, congenital defects, or cardiac masses. A positive result means something abnormal was found, but further evaluation is often needed.

**2. False Positives and Certainty**

- CT is highly sensitive, meaning it is good at detecting abnormalities, but it may sometimes overestimate the severity of disease.
- **Motion artifacts**, poor contrast timing, or calcium "blooming" can affect accuracy.
- If your doctor is uncertain or if the findings don’t match your symptoms, they may recommend additional tests like a **stress test, invasive coronary angiography, or cardiac MRI**.

**3. Next Steps**

- If your CT was a **calcium score**, your doctor will assess your risk factors and possibly recommend lifestyle changes or medication.
- If it was a **CCTA** showing blockages, your doctor may order a **stress test or invasive angiogram** to confirm the findings.
- If you're asymptomatic and have mild abnormalities, monitoring over time may be advised.

Would you like help interpreting your specific results? If you have the report, I can break it down for you.

# 84. My doctor says that my heart MRI is positive, how certain is that?

The certainty of your **heart MRI** being "positive" depends on multiple factors:

**1. What Does "Positive" Mean?**

- Your doctor likely means that the MRI has shown some abnormalities related to your heart structure, function, or tissue characteristics.
- MRI findings are interpreted based on **quantitative and qualitative** measures, such as:
  - **T1 Mapping and ECV (Extracellular Volume Fraction):** These can indicate myocardial fibrosis or inflammation.
  - **LGE (Late Gadolinium Enhancement):** Can suggest scar tissue, ischemia, or other pathologies.
  - **Cine Imaging:** Evaluates heart function and wall motion.
  - **Perfusion Imaging:** Assesses blood flow to the heart muscle.

**2. Certainty of the Result**

- MRI is one of the **most accurate imaging tests** for heart disease, but **false positives and interpretation variability** exist.
- Certainty depends on:
  - **The expertise of the radiologist interpreting the scan.**
  - **Quality of the images** (motion artifacts, poor contrast uptake, etc.).
  - **Clinical correlation** (MRI findings should match symptoms, lab tests, and other imaging).
  - **Use of Contrast** (Certain conditions are better seen with contrast-enhanced scans).

**3. What to Do Next?**

- Ask your doctor **what specific abnormality was found** on MRI.
- Consider a **second opinion** if the findings are unclear or you need further confirmation.
- Your doctor may recommend additional **tests (e.g., blood work, echocardiogram, cardiac catheterization)** to confirm or rule out disease.
- If the MRI suggests a serious condition, treatment options will depend on the specific diagnosis.

Would you like help interpreting specific terms from your MRI report?
